# A Spectrochemical Series for Electron Spin Relaxation

**DOI:** 10.1021/jacs.4c16571

**Published:** 2025-01-08

**Authors:** Nathanael P. Kazmierczak, Kay T. Xia, Erica Sutcliffe, Jonathan P. Aalto, Ryan G. Hadt

**Affiliations:** Division of Chemistry and Chemical Engineering, Arthur Amos Noyes Laboratory of Chemical Physics, California Institute of Technology, Pasadena, California 91125, United States

## Abstract

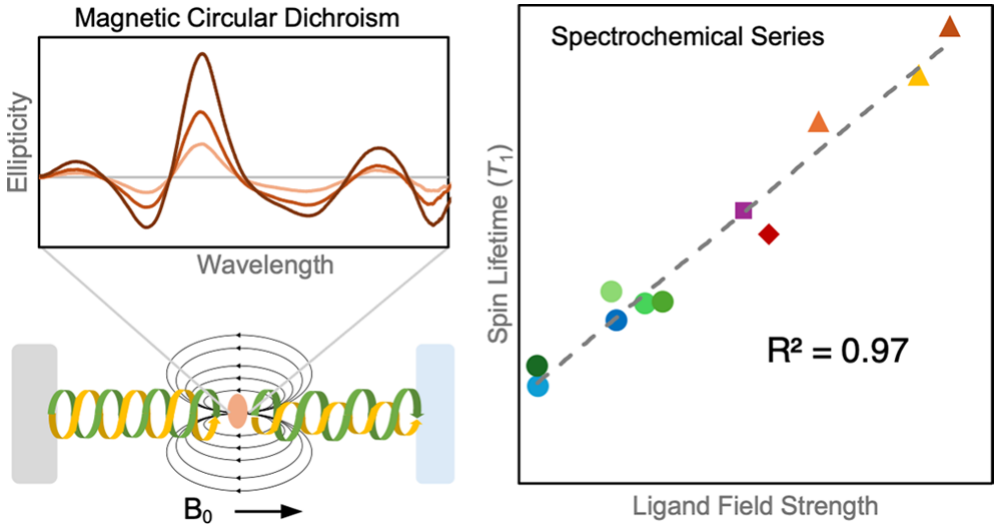

Controlling the rate of electron spin relaxation in paramagnetic molecules is essential for contemporary applications in molecular magnetism and quantum information science. However, the physical mechanisms of spin relaxation remain incompletely understood, and new spectroscopic observables play an important role in evaluating spin dynamics mechanisms and structure–property relationships. Here, we use cryogenic magnetic circular dichroism (MCD) spectroscopy and pulse electron paramagnetic resonance (EPR) in tandem to examine the impact of ligand field (d–d) excited states on spin relaxation rates. We employ a broad scope of square-planar Cu(II) compounds with varying ligand field strength, including CuS_4_, CuN_4_, CuN_2_O_2_, and CuO_4_ first coordination spheres. An unexpectedly strong correlation exists between spin relaxation rates and the average d–d excitation energy (*R*^2^ = 0.97). The relaxation rate trends as the inverse 11th power of the excited-state energies, whereas simplified theoretical models predict only an inverse second power dependence. These experimental results directly implicate ligand field excited states as playing a critical role in the ground-state spin relaxation mechanism. Furthermore, ligand field strength is revealed to be a particularly powerful design principle for spin dynamics, enabling formation of a spectrochemical series for spin relaxation.

## 1 Introduction

The spin dynamics properties of paramagnetic transition metal complexes have been studied since the earliest days of molecular magnetism,^1,2^ but a resurgence of interest has accompanied the recent rise of molecular quantum information science.^3,4^*S* = 1/2 molecular complexes constitute a convenient two-level quantum system, fulfilling the requirements for a quantum bit (qubit). Molecular qubits possess the advantage of extreme miniaturization relative to other qubit platforms, though generation of large entangled arrays remains challenging.^5^ Thus, molecular qubits are believed to possess advantages for quantum sensing applications in chemical microenvironments.^6−9^

**Figure 1:**
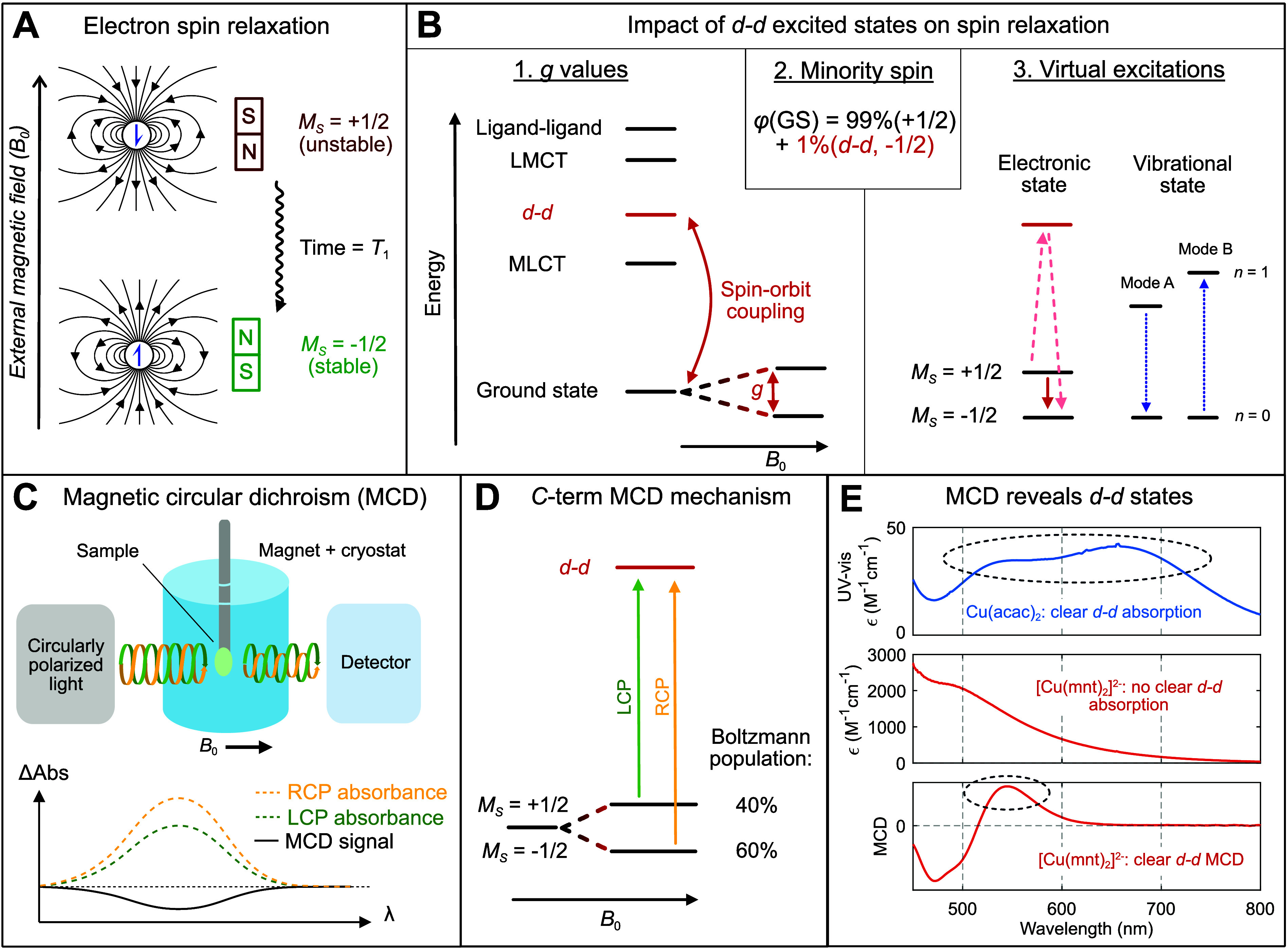
MCD as a useful spectroscopic probe for spin relaxation. (A) Electron spin relaxation arises from the reorientation of spin magnetic dipoles to align with an external magnetic field. (B) Excited states produced by transferring an electron between two d orbitals (d–d transitions) play a key role in spin relaxation under multiple theoretical paradigms. (C) Schematic of the MCD instrument, which produces a signal based on differential absorption of left-handed and right-handed circularly polarized light (LCP/RCP) in the presence of a magnetic field. (D) Paramagnetic complexes produce an MCD signal through differential Boltzmann population of Zeeman sublevels, referred to as the C-term intensity mechanism. (E) The d–d transitions can be invisible in UV–vis–NIR absorption spectroscopy when buried under intense charge transfer transitions. MCD reveals ligand field transitions hidden in UV–vis–NIR absorption spectra (example data shown for Cu(acac)_2_ and (PPh_4_)_2_[Cu(mnt)_2_]).

To enact any quantum information protocol using a molecular qubit, it is necessary that a prepared spin state must retain its orientation and phase with high fidelity over a period of time. These spin states are typically generated in the presence of an applied magnetic field (*B*_0_), such as in a pulse electron paramagnetic resonance (EPR) spectrometer.^10^ However, electron spins possess an intrinsic magnetic dipole, causing them to interact with *B*_0_. An electron spin placed in an antiparallel state to *B*_0_ is out of equilibrium and experiences an energetically unfavorable repulsion (Figure 1A). Over time, such an electron will reorient its spin so that the magnetic dipole realigns with *B*_0_. This process, referred to as spin–lattice relaxation^11^ and given by the time constant *T*_1_, destroys the quantum information stored in the original state. Spin–lattice relaxation occurs through thermalizing interactions between the spin and vibrational modes and thus proceeds much faster at elevated temperatures.^12^ However, the mechanistic details of the spin–vibration coupling remain the subject of theoretical debate.^13^ To realize the full potential of molecular quantum sensing, it is imperative to develop a more robust understanding of chemical factors affecting *T*_1_.

Though spin relaxation occurs between the ground-state *M*_S_ sublevels, it has been suggested that electronic excited states may play an important role. Three distinct classes of spin relaxation models each predict a correlation between relaxation rates and the energy of the d–d (i.e., ligand field) excited states in transition metal complexes (Figure 1B). First, the popular spin Hamiltonian approaches model spin relaxation through the impact of vibrational modes on the *g* value, which controls the energy splitting between the ground-state *M*_S_ sublevels.^14−19^ It is well-known that an empirical correlation often exists between the orbital shift of the *g* value and the rate of spin relaxation,^20^ with studies in both organic nitroxide radicals^21^ and transition metal complexes.^14,22,23^ Dynamic vibrational impacts on *g* are often roughly proportional to the static orbital contribution to *g* itself, so spin Hamiltonian models correctly predict faster relaxation for compounds with greater orbital angular momentum. Crucially, orbital contributions to *g* are produced by out-of-state spin–orbit coupling (SOC) between the ground state and excited d–d states.^10^ The magnitude of this coupling is inversely proportional to the energy gap between the relevant d–d excited state and the ground state, so larger d–d excitation energies should lead to slower relaxation.^14^ Second, it has been shown that shortcomings in the spin Hamiltonian model can be remedied by a wave function theory of spin relaxation that models the amount of ground-state minority spin.^24^ The minority spin is produced by the same SOC mechanism as before, so the wave function theory predicts a similar relationship between d–d excitation energies and *T*_1_. The d–d excitation energies may also be used to explain features of *T*_1_ anisotropy.^24^ Third, a recent approach has invoked virtual excitations to the d–d excited states as the primary driver of spin relaxation; these excitations become more feasible with reduced d–d excitation energy.^25^

Despite these predictions, there does not exist strong, direct experimental evidence for the impact of d–d excitation energies on *T*_1_. The d–d transitions are weak in intensity because of the Laporte selection rule (ε = 10–100 M^–1^ cm^–1^). If a compound has no spectral congestion from transitions involving ligands, then the d–d transitions can be observed through UV–vis–NIR absorption spectroscopy (Figure 1E, top). However, many highly coherent molecules possess extended π-conjugation and significant ligand–metal covalency. This induces intense charge transfer transitions (ε > 1000 M^–1^cm^–1^) across the visible spectrum, effectively masking the locations of the d–d states (Figure 1E, middle).^24^ UV–vis–NIR absorption spectroscopy alone is thus insufficient to reliably quantitate d–d excitation energies across a broad scope of *S* = 1/2 molecules.

Magnetic circular dichroism (MCD) spectroscopy overcomes these limitations by selectively enhancing the strength of the d–d transitions. MCD is superficially related to the more familiar circular dichroism (CD) measurement; in both cases, a signal is produced from differential absorption of left- and right-handed circularly polarized light (LCP/RCP) (Figure 1C).^26^ However, the mechanism of dichroism is fundamentally distinct. In CD spectroscopy, signals can only arise when the molecule is chiral. MCD, however, does not require a chiral structure, and signals can arise even for achiral molecules, such as square-planar Cu(II) complexes^27−30^ and related VO(IV) complexes.^31^ Dichroism is instead produced by the interaction of an applied magnetic field with the molecule’s electronic structure and magnetic moment. A variety of books and reviews have covered the mathematical theory and experimental history of MCD,^26,32−34^ with notable applications to bioinorganic metal active sites.^35,36^ Of relevance here, a major MCD intensity mechanism for paramagnetic molecules (referred to as the *C*-term mechanism) arises from unequal Boltzmann population of the ground-state Zeeman sublevels (Figure 1D). The population inequality increases as the temperature is decreased, so *C*-term MCD spectra are best acquired at cryogenic temperatures (2–20 K). In the presence of SOC, a transition from a particular Zeeman sublevel (say, *M*_S_ = −1/2) to a given excited-state *J* will exhibit preferential absorption for RCP or LCP light. When the ground state is energetically well-separated from the excited states, the degree of this preference is often dominated by the strength of SOC in the excited-state *J*.^37,38^ Crucially, d–d excited states have much stronger SOC than charge transfer or ligand-based excited states, as the metal-centered SOC constant is typically up to an order of magnitude larger than on the ligand. Thus, d–d transitions intrinsically possess an amplified *C*-term MCD signal. MCD spectra can therefore resolve d–d transitions that are hidden beneath charge transfer transitions in the UV–vis–NIR absorption spectrum (Figure 1E, bottom).^39^

**Figure 2:**
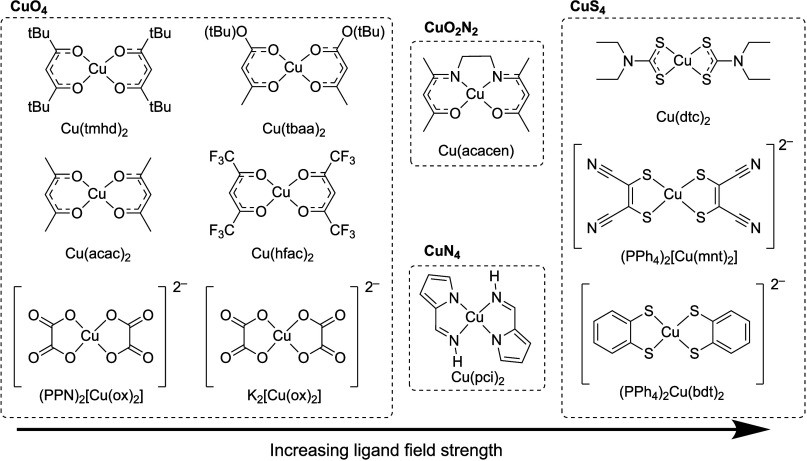
Compound scope for correlating MCD to spin relaxation rates. All compounds possess an approximately square-planar first coordination sphere in the absence of axial ligation. Ligand abbreviations: tmhd^–^ = 2,2,6,6-tetramethylheptanedionate, tbaa^–^ = *tert*-butylacetoacetate; acac^–^ = acetylacetonate, hfac^–^ = hexafluoroacetylacetonate, ox^2–^ = oxalate, acacen^2–^ = bis(acetylacetonato)ethylenediamine, pci^–^ = pyrrolylcarbaldimine, dtc^–^ = diethyldithiocarbamate, mnt^2–^ = maleonitriledithiolate, and bdt^2–^ = benzenedithiolate.

In this work, we leverage cryogenic MCD spectroscopy to accurately determine ligand field energies across a broad scope of square-planar Cu(II) complexes (Figure 2).^13,24,40−45^ The series includes molecules known to have long-lived spin lifetimes (e.g., [Cu(mnt)_2_]^2–^), as well as reference compounds not previously studied for their spin relaxation properties (e.g., [Cu(ox)_2_]^2–^). *T*_1_ measurements at 100 K are subsequently acquired for each member of the series using matrix preparations identical or comparable to the MCD samples. This study provides the first direct experimental correlation between ligand field strength and spin relaxation rates. The unexpectedly strong correlation provides new insights into spin relaxation mechanisms and suggests that ligand field excited states dictate the spin dynamics behavior of transition metal complexes more than previously realized.

## 2 Results

### 2.1 Assigning d–d Transitions

To assign d–d bands, the first step is to acquire low-temperature MCD and UV–vis–NIR absorption spectra, which necessitates immobilization of the molecule in an optically transparent matrix. Three sample preparation techniques were used in this work (Supporting Information Section 1.3). First, the analyte can be dissolved into a polymer film, such as polystyrene (PS), poly(methyl methacrylate) (PMMA), or poly(vinyl alcohol) (PVA), and drop-cast onto a quartz disc.^46,47^ Polymer film samples generally have excellent optical properties permitting measurement of both MCD and absorption, but solubility can be limited, and the geometry of the compound is not crystallographically known. Second, the analyte can be dissolved into an optically glassing solvent and frozen in a home-built cell. This method can allow high solubility and good optical quality, but only a few solvents are optically transparent when frozen (such as butyronitrile, 2-methyl THF, and 3:7 glycerol:water).^48^ Third, a solid crystalline powder of the analyte can be finely ground and suspended in fluorolube, referred to as a mull. Mull samples have a crystallographically known geometry but typically possess inferior optical quality, making it challenging to reliably measure absorption spectra. The sample preparation methods used for all compounds are tabulated in Table S1 in the Supporting Information.

**Figure 3:**
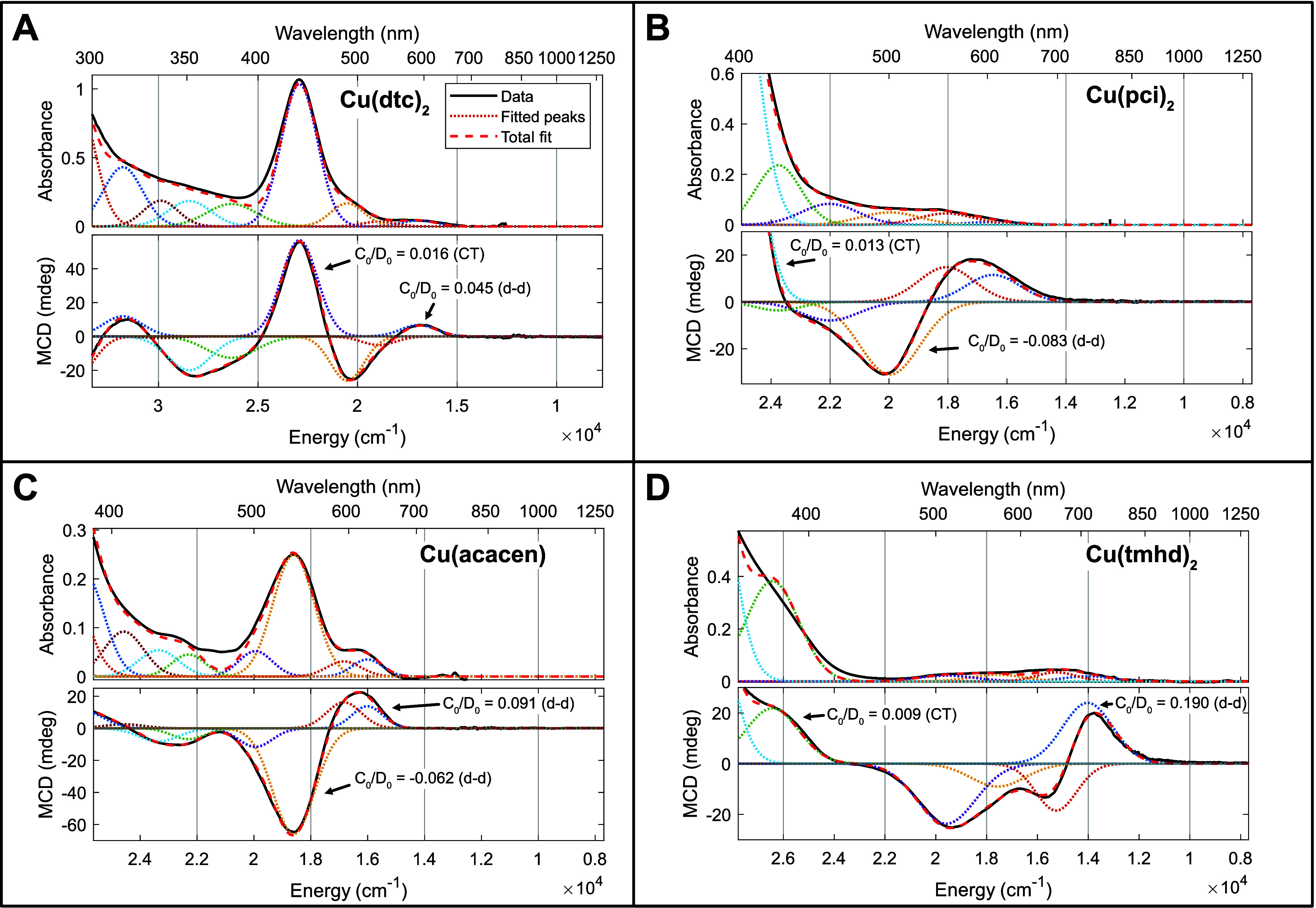
Assignment of electronic transitions through Gaussian band fitting of representative MCD and UV–vis–NIR absorption spectra. The relative intensity of transitions in MCD vs absorbance is denoted by the *C*_0_/*D*_0_ ratio; a magnitude of ∼0.01 is indicative of charge transfer transitions, while ∼0.1 is indicative of d–d transitions. (A) Cu(dtc)_2_ MCD collected in PS film at ±2 T and 5.5–10.0 K. (B) Cu(pci)_2_ MCD in PS film at ±4 T and 5.0–10.0 K. (C) Cu(acacen) MCD collected in PS film at ±2 T and 5.0–10.0 K. (D) Cu(tmhd)_2_ MCD collected in PMMA film at ±2 T and 5.0–20.0 K. All UV–vis–NIR absorption spectra are collected at the lowest temperature for which MCD data were measured. Two-point temperature subtractions eliminate temperature-independent features, yielding the pure C-term spectrum.

Initially, we employed polymer films to simultaneously acquire *C*-term MCD and absorption spectra at cryogenic temperatures (2–20 K). Representative spectra for complexes from each class of coordinating ligand (e.g., CuS_4_, CuN_4_, CuN_2_O_2_, and CuO_4_) are displayed in Figure 3, and full fitting information is provided in Supporting Information Sections 5.2–5.3. While a few intense peaks are displayed in the absorption spectra, such as Cu(dtc)_2_ at 430 nm (Figure 3A), many areas in the visible absorption spectra are comparatively flat and featureless, such as Cu(pci)_2_ from 450 to 650 nm (Figure 3B). However, all compounds display a clear structure in the MCD spectra. The flat absorption tail for Cu(pci)_2_ is resolved into multiple signed bands in the MCD (Figure 3B). Similarly, multiple peaks are discernible in the Cu(tmhd)_2_ MCD despite very low absorption (Figure 3D). These observations already suggest that the MCD spectra are successfully detecting d–d states that are not directly visible in the absorption spectra.

To quantitatively assign the d–d transitions, we performed Gaussian peak resolution to identify the spectral transitions (Figure 3).^39^ Because MCD and absorption both arise from the same electronic states, we modeled Gaussian peaks as having the same energetic position and width in both spectra. The ratio of the MCD *C*-term and absorption transition moments (the latter is traditionally denoted as “*D*_0_” in the MCD literature) can then be directly compared to give information about the nature of the excited state. In the linear limit, the *C*_0_/*D*_0_ ratio may be calculated according to eq 1 (see also Supporting Information Section 5.1).^26,33,49^ Here, ε represents the molar absorptivity, and Δε gives the MCD spectrum in units of differential molar absorptivity:^26^
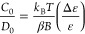
1

It has been previously shown that a *C*_0_/*D*_0_ ratio around 0.1 is diagnostic of a d–d excited state, while a *C*_0_/*D*_0_ ratio around 0.01 is diagnostic of a charge transfer state.^50^ In other words, d–d states have more intrinsic magnetic response per unit light absorption, owing to the enhanced metal-centered SOC.

Examination of the Figure 3*C*_0_/*D*_0_ fits reveals important commonalities across all four compounds. Cu(dtc)_2_ (Figure 3A) exhibits three bands from 21,000 to 16,000 cm^–1^ with *C*_0_/*D*_0_ ratios between 0.04 and 0.05; while somewhat small, such *C*_0_/*D*_0_ ratios are best assigned to d–d transitions. By contrast, the intense transition at 23,000 cm^–1^ possesses a *C*_0_/*D*_0_ ratio of only 0.016, and the higher-energy transitions have similar values. Thus, the 23,000 cm^–1^ band may be assigned to charge transfer, consistent with its intense extinction coefficient. The lowest-energy d–d band has a positive MCD sign, while the highest-energy assigned d–d band has a negative MCD sign. Cu(pci)_2_ (Figure 3B) possesses three strong d–d bands from 20,000–16,000 cm^–1^ with *C*_0_/*D*_0_ ratios around 0.08. The negative MCD peak at 19,970 cm^–1^ is especially prominent, despite not being resolved in the absorption spectrum. Cu(acacen) (Figure 3C) possesses a strong absorption peak at 18,600 cm^–1^ that is similar in appearance to the Cu(dtc)_2_ charge transfer. However, the *C*_0_/*D*_0_ ratio is much larger at 0.062, indicating that this is a d–d transition in Cu(acacen). Furthermore, the transition has a prominent negative MCD sign, similar to the highest-energy d–d transition in Cu(pci)_2_. Finally, Cu(tmhd)_2_ (Figure 3D) displays *C*_0_/*D*_0_ ratios at or above 0.1 for four d–d bands in the visible region, with a prominent negative MCD peak at 19,590 cm^–1^. Note that in all four compounds, the highest-energy d–d band has a strong negative MCD signal, while the lowest-energy d–d band has a positive MCD signal. This spectral characteristic is conserved across the entire compound scope, enabling assignment of d–d transitions even when the UV–vis–NIR absorption spectrum cannot be obtained.

### 2.2 Comparing Ligand Field Strengths

**Figure 4:**
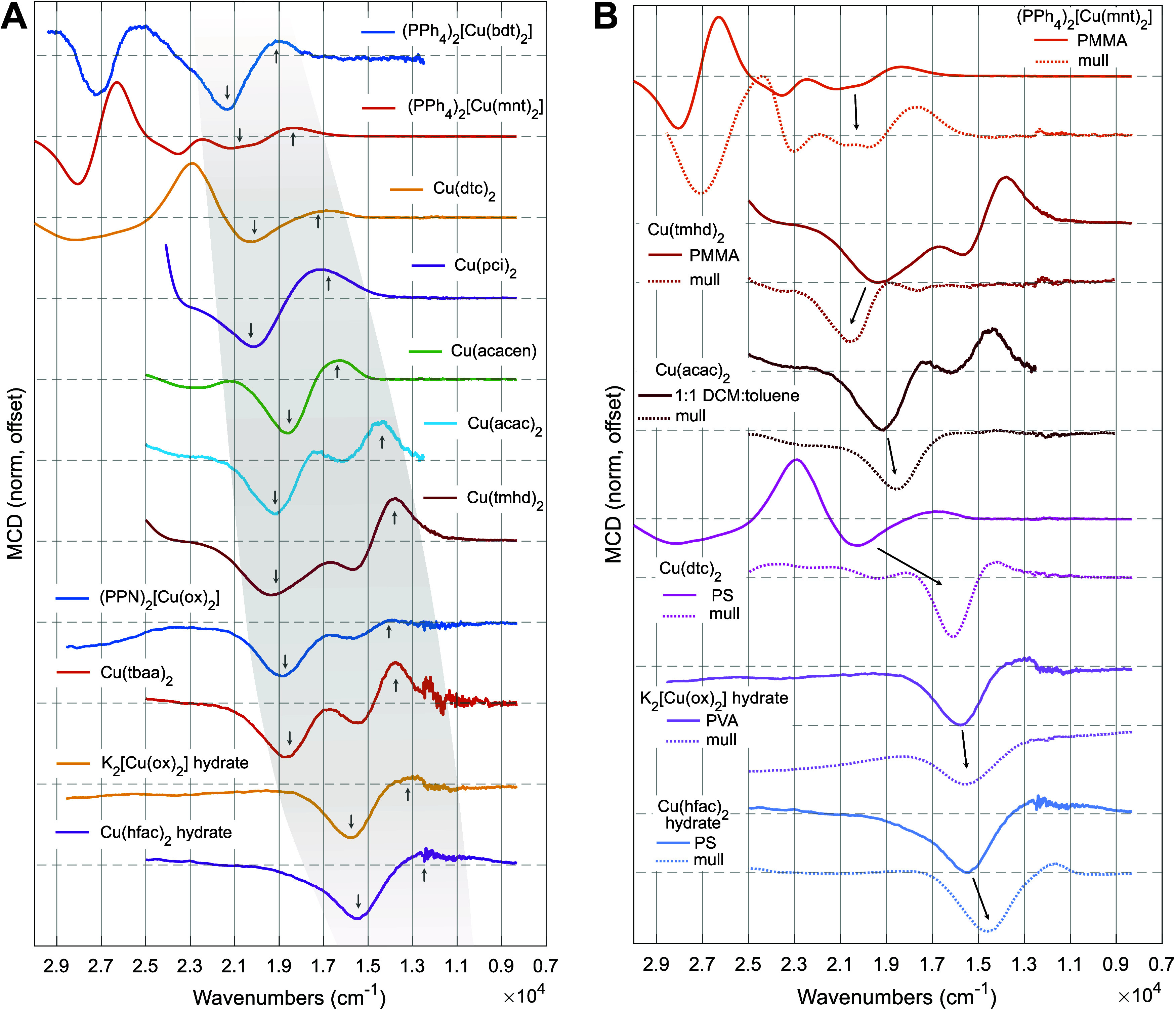
MCD spectral scope. (A) Comparison of MCD spectra across all compounds in disordered matrices (polymer film, solvent glass). The gray swath indicates the region assigned to d–d transitions. Gray arrows indicate negative and positive features common to all spectra that bracket the d–d transitions. Matrices: (PPh_4_)_2_[Cu(bdt)_2_] in 1:1 butyronitrile:DCM, (PPh_4_)_2_[Cu(mnt)_2_] in PMMA, Cu(dtc)_2_ in PS, Cu(pci)_2_ in PS, Cu(acacen) in PS, Cu(acac)_2_ in 1:1 DCM:toluene, Cu(tmhd)_2_ in PMMA, (PPN)_2_[Cu(ox)_2_] in PS, Cu(tbaa)_2_ in PS, K_2_[Cu(ox)_2_] in PVA, and Cu(hfac)_2_ in PS. (B) Comparison of matrix effects on MCD spectra for selected compounds. Polymer film or solution spectra are replicated from panel A. Arrows denote the shift in the prominent negative d–d MCD signal. Temperatures, fields, and matrices are given for each spectrum in Supporting Information Section 5.1.1.

Having identified the d–d transitions from *C*_0_/*D*_0_ fitting, the positions of the d–d bands across the compound scope may be compared (Figure 4). First, we examined the samples in randomly oriented matrices, either polymer films or frozen solutions (Figure 4A). In all 11 spectra, the d–d region is bookended by a negative MCD transition at higher energy and a positive MCD transition at lower energy. A total of four d–d transitions are expected for a d^9^ Cu(II) complex. For some compounds, such as [Cu(bdt)_2_]^2–^, Cu(pci)_2_, and Cu(hfac)_2_, no extra resolved d–d peaks are observed in an intermediate energy range relative to the bookend transitions, though band asymmetry hints at extra transitions for Cu(pci)_2_. These spectra also contain comparatively small energy gaps between the bookends, suggesting closely spaced d–d manifolds. For other CuO_4_ derivatives (Cu(acac)_2_, Cu(tmhd)_2_, and Cu(tbaa)_2_), however, two additional prominent peaks are found between the bookend transitions. These observations account for all four d–d states in the MCD. Note that while the CuO_4_ compounds do not have charge transfer transitions obscuring the d–d region, the four d–d states are not all individually resolved in the UV–vis–NIR absorption spectra. These MCD spectra thus provide enhanced ligand field information on all classes of compounds studied.

The sign of the MCD alone does not necessarily give an unambiguous indication of the precise d–d transitions, so calculations are valuable for conducting specific state assignments. In previous studies of rhombically distorted *C*_3*v*_ Cu(II) metalloprotein active sites, an intense negative MCD feature at the highest d–d excitation energy was assigned to an *xz*/*yz* → *x*^2^–*y*^2^ transition,^39^ while a negative transition at the highest d–d excitation energy arose from *z*^2^ → *x*^2^–*y*^2^ in *D*_4*h*_ CuCl_4_^2–^.^30^ However, the sign of MCD transitions can remain invariant in low-symmetry systems even for multiple orderings of the excited states.^38^ The expected band signs and energy orderings must be independently evaluated for the present *D*_2*h*_ complexes. Time-dependent density functional theory (TDDFT) calculations consistently assign the highest-energy state to the *x*^2^–*y*^2^ → *xy* transition in *D*_2*h*_ symmetry (Supporting Information Section 7.1), while the lowest-energy state is often (but not always) assigned to the *xz* → *xy* state. This analysis is consistent with the expected ordering for a nonbonding *x*^2^–*y*^2^ orbital and π-donating ligands.

A substantial shift in ligand field strength was observed across the series (Figure 4A). The dithiolenes [Cu(bdt)_2_]^2–^ and [Cu(mnt)_2_]^2–^ possess the highest-energy d–d bands at averages of 20,460 and 20,070 cm^–1^, while the weakest ligand fields are displayed by K_2_[Cu(ox)_2_] in PVA and Cu(hfac)_2_ at 15,250 and 15,230 cm^–1^. This constitutes a 5000 cm^–1^ range, giving a 34% change in ligand field strength relative to the Cu(hfac)_2_ endmember. The average d–d excitation energies are reliably ordered by the type of first coordination sphere: all CuS_4_ > all CuN_4_ > all CuN_2_O_2_ > all CuO_4_. This is in good agreement with expectations from fundamental ligand field theory and with the observed CW EPR *g* values (Table S25). The O_4_ acetylacetonate and oxalate donors possess lone pairs with facile mixing into the metal *xz* and *yz* orbitals, producing antibonding character, raising the orbital energy, and decreasing the gap to the *xy* acceptor. The antibonding character is visible in the *xz* donor NTOs from TDDFT (for example, Figure S122). Upon transitioning to N_2_O_2_ and N_4_, the lone pairs are progressively removed, removing the π antibonding character and increasing the average transition energy. When moving to S_4_, strong σ-donation leads to a high-lying σ* *xy* acceptor orbital. The σ strength of the dithiolene ligands arises both from excellent orbital overlap of the diffuse S ligand and also close energetic matching of the S and metal orbitals, which can in some cases (such as for [Cu(mnt)_2_]^2–^) produce an inverted bonding regime.^51−55^ Note that a weak MCD transition was detected at 8250 cm^–1^ for [Cu(mnt)_2_]^2–^ with a *C*_0_/*D*_0_ ratio of only 0.02 (Figures S26–S29). Though this donor orbital has the symmetry of the *xz* orbital, the low *C*_0_/*D*_0_ ratio indicates a predominantly charge transfer character. This assignment is in agreement with recent S K-edge 1s3p RIXS analysis, which concluded that a primarily LMCT character is the best description of the state.^56^ Additionally, the TDDFT NTO donor orbital also displays primarily ligand character. Therefore, we do not include this transition in the calculation of the average d–d excitation energy for [Cu(mnt)_2_]^2–^. In sum, MCD spectroscopy assigns a ligand field strength ordering of CuS_4_ > CuN_4_ > CuN_2_O_2_ > CuO_4_.

### 2.3 Impact of Axial Coordination

Close inspection of Figure 4A reveals that two different sample preparations of [Cu(ox)_2_]^2–^ possess significantly different d–d excitation energies. When prepared with the comparatively nonpolar PPN^+^ counterion and dissolved in a noncoordinating PS film, (PPN)_2_[Cu(ox)_2_] displayed the *x*^2^–*y*^2^ → *xy* transition with a strong negative MCD signal at 18,940 cm^–1^. However, when prepared with the K^+^ counterion and dissolved in the water-soluble PVA polymer, K_2_[Cu(ox)_2_] displayed a significant shift of the *x*^2^–*y*^2^ → *xy* transition to 15,640 cm^–1^. A concomitant shift in *g*_*z*_ from 2.255 to 2.322 was observed from (PPN)_2_[Cu(ox)_2_] in PS to K_2_[Cu(ox)_2_] in 3:7 glycerol:water (a solution phase model of the PVA environment), consistent with an increase in ground-state orbital angular momentum from a weakened ligand field (Table S25). We posited that this shift could be explained by axial coordination of the alcohol groups in the PVA film, leading to a six-coordinate Cu(II) site with expanded equatorial bond lengths and a weakened σ* interaction. Explicit solvation TDDFT calculations using the ORCA SOLVATOR^57^ module support this interpretation (Supporting Information Sections 7.4–7.5). In the absence of axial ligands, TDDFT predicts an *x*^2^–*y*^2^ → *xy* energy of 19,150 cm^–1^ for [Cu(ox)_2_]^2–^ (Table S29). Addition of explicit water solvation predicted a single axially coordinated H_2_O molecule (Table S77), from which TDDFT predicted an *x*^2^–*y*^2^ → *xy* energy of 16,480 cm^–1^ (Table S90). Addition of explicit methanol solvation predicted two axial alcohol coordination sites (Table S77), and TDDFT predicted an *x*^2^–*y*^2^ → *xy* energy of 16,520 cm^–1^ (Table S94). The alcohol groups model the environment of the PVA matrix. Both explicit solvation approaches predict a band shift of about 2650 cm^–1^, which is in good agreement with the experimental shift between the two sample matrices (3300 cm^–1^). Note that the secondary peak near 16,000 cm^–1^ in the (PPN)_2_[Cu(ox)_2_] PS film may arise from an axially coordinated species due to residual water, as this aligns with the PVA film. Additionally, a hydrated Cu(hfac)_2_ PS film possesses the weakest ligand field of all compounds studied; this compound commonly crystallizes as a hydrate with 1–2 axial waters ligated to the metal.^58,59^ The axial ligation may be retained in the PS film and contribute to a weaker ligand field. These observations motivated further investigation of the role of axial coordination and the sample matrix in determining the measured ligand field strength.

MCD spectra in fluorolube mulls were acquired for six compounds and compared to the corresponding polymer film or solution spectra (Figure 4B). For (PPh_4_)_2_[Cu(mnt)_2_], a slight overall redshift was observed in the mull, which may be attributed to the dielectric change in a crystalline powder. The band shape of the d–d transitions remained consistent, suggesting no major changes in compound geometry. Hydrated K_2_[Cu(ox)_2_] and Cu(hfac)_2_, which have crystallographic axial coordination, display minimal changes between the films and the mulls, suggesting that the samples are axially coordinated in both sample preparations. Cu(tmhd)_2_ displays a blueshift of the *x*^2^–*y*^2^ → *xy* transition, unique among the mull samples. Both Cu(tmhd)_2_ and Cu(acac)_2_ display a significant reduction in intensity of the lowest-energy positive MCD band, which is assigned to the *xz* → *xy* transition. The *x*^2^–*y*^2^ → *xy* transition remains prominent and negative. The origin of this reduction is unclear but may arise from increased conformational flexibility in the polymer/solution imparting enhanced electric dipole intensity to this transition. Finally, Cu(dtc)_2_ displays the most prominent change of all the compounds. The negative *x*^2^–*y*^2^ → *xy* band shifts dramatically from over 20,000 cm^–1^ in the PS polymer film to just above 16,000 cm^–1^ in the mull, and the mull spectrum is more similar in appearance to the CuO_4_ samples. This shift likely arises because Cu(dtc)_2_ crystallizes as a staggered dimer, where the in-plane dtc ligand for one molecular unit provides out-of-plane axial coordination for the other molecular unit.^60^ Cu(dtc)_2_ likely dissociates into free square-planar monomers in the PS polymer film, supported by the lack of propensity to axial coordination in the explicit solvation DFT calculations (Table S77) and the observation of a strong *S* = 1/2 EPR signal. Thus, the strong mull MCD redshift for Cu(dtc)_2_ is also explained by axial coordination.

### 2.4 Correlation to Spin Relaxation Rates

**Figure 5:**
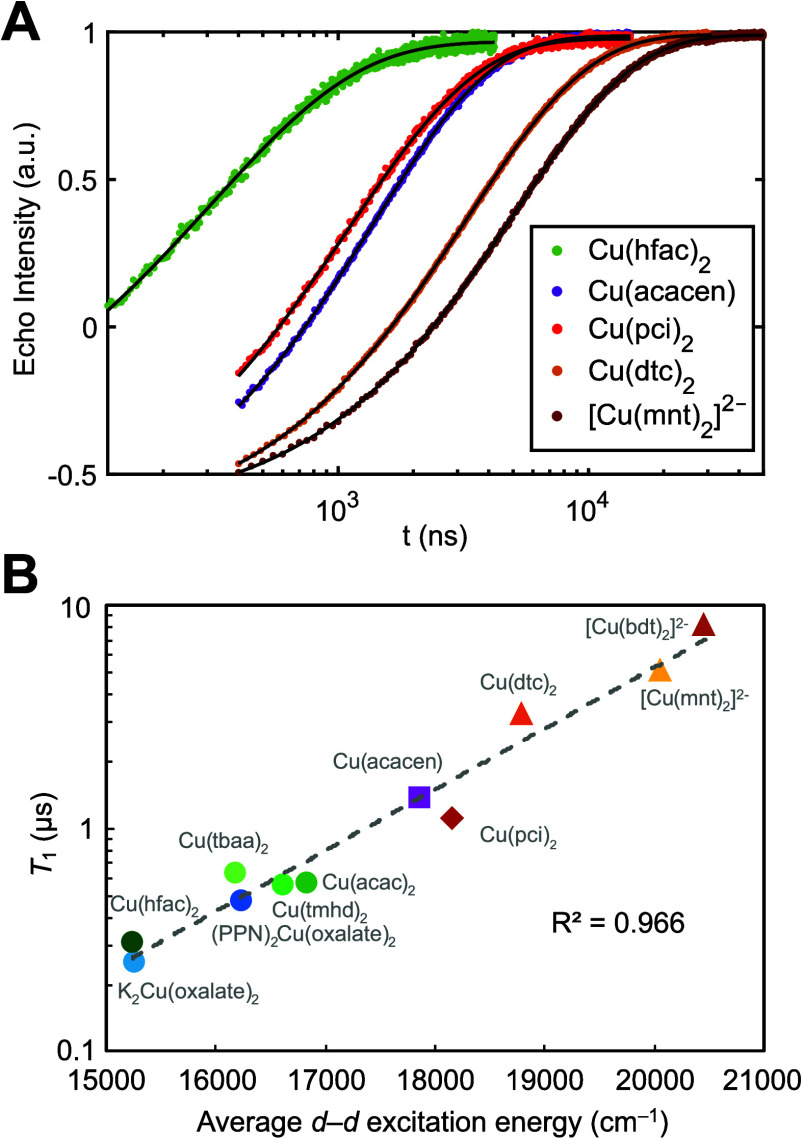
Correlation between MCD and pulse EPR spin relaxation rates. (A) Inversion recovery traces for selected compounds, acquired in the polymer matrix used for MCD (Cu(hfac)_2_ in PS, Cu(acacen) in PS, Cu(pci)_2_ in PS, Cu(dtc)_2_ in PS, and [Cu(mnt)_2_]^2–^ in PMMA). Stretched exponential fits yield the value of *T*_1_. (B) Strong linear correlation between log(*T*_1_) and the average d–d excited-state energy determined from the MCD spectra. Error bars are smaller than the markers; tabulated in Table S26.

We next sought to correlate the observed MCD ligand field strengths to the rates of spin relaxation. Pulse EPR X-band inversion recovery measurements were conducted at 100 K and fit to stretched exponentials to extract *T*_1_ (Figure 5A). The temperature was chosen to ensure that molecular vibrations localized to the first coordination sphere constituted the dominant driving force for spin relaxation, as opposed to low-energy phonons.^24^ The correlation between d–d excited-state energies and spin relaxation rates has been theoretically predicted under a dominant two-phonon Raman relaxation mechanism with molecular vibrations at elevated temperatures (Figure 1B).^10,15^ Spectral diffusion^10^ is not a major factor at this temperature, so saturation recovery measurements are not needed. For most of the polymer film MCD samples, the films could be simply cut into strips and placed in an EPR tube. Strong CW and pulse EPR signals validated the dominant presence of magnetically dilute sites, with spin Hamiltonian parameters consistent with known molecular values in other matrices (Table S25). All *T*_1_ measurements were collected at the most intense microwave absorption feature (powder line) to remove orientation effects, which is most appropriate for conducting the correlation analysis with the average d–d excitation energy.

To ensure that sample matrix differences did not affect the correlation analysis, EPR sample preparation was kept as close to the MCD sample preparation as possible (Table S1). For seven of the 11 samples, the MCD polymer film was reused for the EPR measurements, ensuring direct comparability. For [Cu(ox)_2_]^2–^, the coordinating PVA film was modeled by a 3:7 glycerol:water solvent mixture, while the noncoordinating PS film was modeled by a toluene solvent system. [Cu(bdt)_2_]^2–^ and Cu(acac)_2_ were run in glassing solvent mixtures for both MCD and EPR. Mull MCD samples were necessarily excluded from the correlation analysis, as these paramagnetically concentrated powders do not display a spin echo in pulse EPR. There is some evidence that changing the matrix affects both the d–d excitation energies and the *T*_1_ values in the same way (Figure 5B). For [Cu(ox)_2_]^2–^, preparation in an axially coordinating matrix (K^+^ counterion hydrate) led to both a reduced d–d excitation energy (15,250 cm^–1^) and a reduced *T*_1_ (254 ns) as compared to the preparation in a noncoordinating PPN^+^ matrix (d–d = 16,240 cm^–1^; *T*_1_ = 480 ns). Therefore, while changes in the sample matrix can alter both the electronic structure and spin relaxation rates, these effects have been accounted for in the experimental design such that direct comparisons may be made.

A very strong correlation between the spin–lattice relaxation rates and the excited-state energies was observed (Figure 5B). A correlation plot of log(1/*T*_1_) versus the average d–d excitation energy yields a linear fit with *R*^2^ = 0.966. Notably, measured *T*_1_ values at 100 K range from 8.15 μs ([Cu(bdt)_2_]^2–^) to 308 ns (Cu(hfac)_2_), a change by a factor of 26.5 over the smaller value. However, the average d–d excitation energies for these two compounds are 20,460 and 15,230 cm^–1^, respectively, which only constitutes a change by a factor of 1.34. The remarkable dependence of *T*_1_ on comparatively small changes in excited-state energies is discussed below.

## 3 Discussion

**Figure 6:**
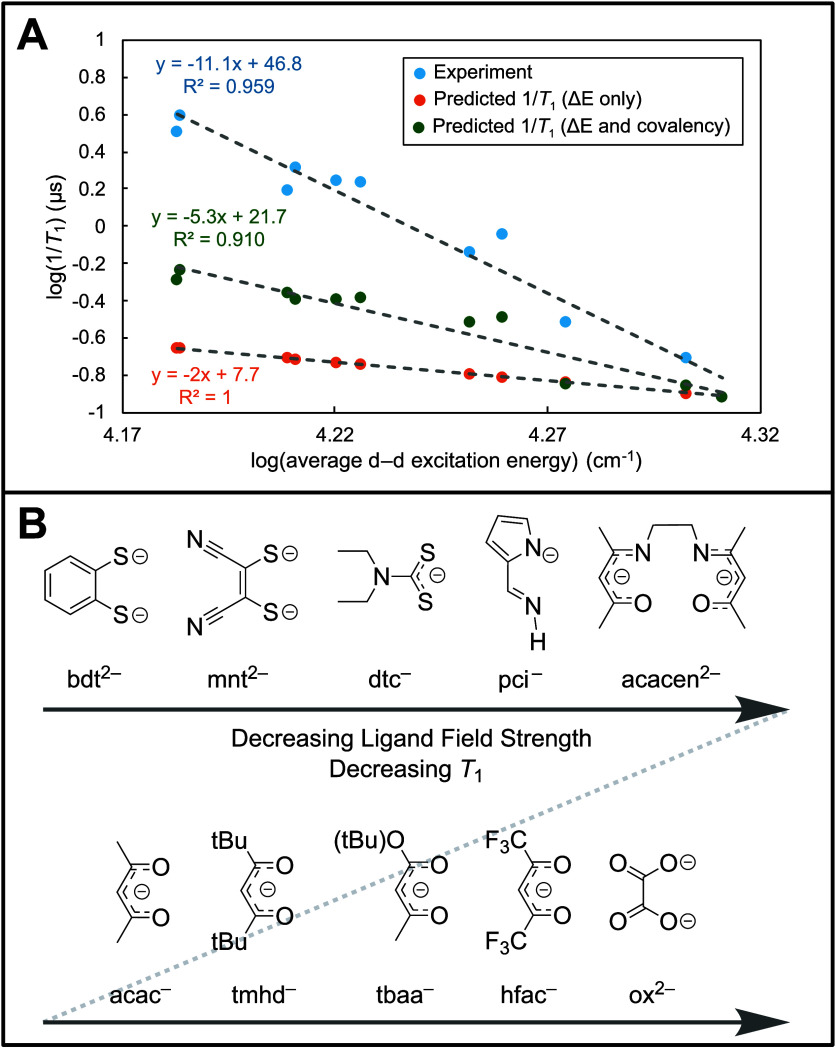
(A) Log–log plot of predicted *T*_1_ scaling with d–d excitation energies (1/*T*_1_ ∝ Δ*E*^–2^ or ≈ Δ*E*^–5^) compared to experimental scaling (1/*T*_1_ ∝ Δ*E*^–11^). (B) Spectrochemical series for spin relaxation.

We set out to quantify the surprisingly steep changes in *T*_1_ with d–d excited-state energies and compare the experimental correlation to contemporary theoretical predictions. Denoting the average d–d excited-state energy as Δ*E*, we correlated 1/*T*_1_ and Δ*E* on a double logarithm plot to extract the effective power law scaling between the two variables. A linear fit to log(1/*T*_1_) vs log(Δ*E*) gives a slope of approximately −11 (Figure 6A), implying 1/*T*_1_ ∝ Δ*E*^–11^. This remarkable scaling is substantially stronger than would be naïvely predicted by examination of contemporary spin relaxation models.

To illustrate this unexpected result, we consider three main classes of spin relaxation models (Figure 1B): (1) spin Hamiltonian, (2) minority spin, and (3) virtual excitations. In the first class, the spin Hamiltonian *g* value itself scales as Δ*E*^–1^ according to a well-established relationship from second-order perturbation theory (eq 2, where *E*_*e*_ – *E*_*g*_ = Δ*E*).^15,61^
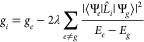
2

To connect the equilibrium *g* value to spin relaxation, it is common to differentiate eq 2 with respect to a vibrational mode *Q*. The derivative, d*g*/d*Q*, is referred to as the spin–phonon coupling coefficient and predicts the intrinsic propensity of a vibrational mode to induce spin relaxation. The leading order term in d*g*/d*Q* scales as Δ*E*^–2^.^14^ Models using d*g*/d*Q* as the spin relaxation coefficient will thus predict that 1/*T*_1_ scales somewhere between Δ*E*^–2^ or Δ*E*^–4^, depending whether the d*g*/d*Q* coefficient is squared or not.^14,15,19^ The unclarity in coefficient squaring arises because d*g*/d*Q* is a proxy for spin relaxation, rather than a true spin–flip matrix element amenable to Fermi’s golden rule treatment.^24^ For the second class of model, spin relaxation is proportional to the minority spin^24^ in the ground-state wave function, which in turn is proportional to the out-of-state SOC. The minority spin in the ground-state wave function (Figure 1B) also scales^24^ as Δ*E*^–1^, so squaring the matrix element in Fermi’s golden rule predicts 1/*T*_1_ ∝ Δ*E*^–2^. In the third class, the Γ_II_ virtual transition mechanism (Figure 1B) similarly contains a Δ*E*^–2^ dependence from the virtual transitions,^25^ though the scaling of the matrix elements is unclear. The Δ*E*^–11^ scaling of 1/*T*_1_ is therefore substantially stronger than would be naïvely predicted by examination of any of these models. Thus, contemporary theory only incompletely describes the impact of excited states on spin relaxation.

One possible explanation arises from ligand field theory analysis of inorganic bonding. Ligand field spin dynamics predicts^10,14,15^ four separate factors impacting *T*_1_: (1) the d–d excited-state energy, (2) the ligand–metal covalency, (3) the thermal population of the coupling vibrations, and (4) the magnitude of the excited-state vibronic coupling. Minimizing spin relaxation thus effectively constitutes an optimization problem in four dimensions. However, if multiple dimensions are tightly related in a series of molecules, then it may be possible to obtain exceptionally steep apparent correlations. The Cu(II)–S compounds probed here are known to have highly covalent ligand–metal bonds, which produce an orbital reduction factor^62^ that reduces the effective orbital angular momentum available for spin–vibration coupling.^51^ Using the experimental EPR *g* values, we extracted effective orbital reduction factors for each compound (Supporting Information Section 6.3) that model the effects of bond covalency. Inclusion of covalency leads to a predicted scaling of 1/*T*_1_ ∝ Δ*E*^–5^. While closer to experiment, this prediction still substantially underestimates the scaling. Alterations of the vibrational mode frequencies for different ligand frameworks may provide an additional contributing factor, though a full spin–phonon coupling analysis for all compounds is beyond the scope of the present study.

Irrespective of the theoretical details, this work demonstrates that ligand field strength can be an exceptionally powerful predictor for spin–lattice relaxation. Changing the d–d excitation energies by only 5000 cm^–1^ can be sufficient to alter *T*_1_ by over a factor of 25. The logic of ligand field strength is furthermore a commonly employed synthetic design principle. For spin-based technological applications, great dividends may be produced by engineering compounds with the strongest possible ligand field strength. This can be accomplished in square-planar Cu(II) complexes both through strong-field ligands and through avoiding axial solvent coordination, which tends to weaken the ligand field. A similar approach should be applicable to square-pyramidal V(IV)O complexes, which are known to have long *T*_1_ values.^63^ On the basis of the MCD spectra, a spectrochemical series for spin relaxation can be formulated (Figure 6B).

## Conclusions

By leveraging the selectivity of MCD spectroscopy, this study reports the first experimental demonstration of a strong correlation between ligand field strength and spin relaxation rates. This trend validates a general prediction of the ligand field approach to spin dynamics, showing that analysis of the static electronic structure can explain many spin dynamics phenomena.^10,14,15,24^ The use of MCD spectroscopy enables quantification of ligand field strength even when the requisite bands cannot be detected in UV–vis–NIR absorption spectroscopy. As such, MCD is a valuable addition to the spectroscopic toolkit for studying spin relaxation mechanisms.

We emphasize that while spin relaxation is a ground-state process, the mechanism of spin relaxation is controlled by excited states. These electronic states are never populated during the process of spin relaxation, but they influence the motion of the electron spin through out-of-state SOC interactions and/or virtual excitations. MCD bands correlate to spin relaxation rates because MCD probes these relevant excited states. As such, it is essential to consider the full electronic state diagram when assessing spin relaxation mechanisms. Ligand field theory provides an indispensable tool for understanding the connection of spin properties to electronic structure design.

In the quest to produce molecules with long coherence times, formulation of reliable and practical synthetic guidelines for spin dynamics properties has been highly desired. Theories of spin relaxation have implicated multiple factors, including vibrational energy,^64,65^ ligand–metal covalency,^45^ coordination geometry,^63^ and excited-state energy.^24,25^ However, the very strong correlation demonstrated herein suggests that ligand field strength may be capable of predicting much, if not most, of the *T*_1_ variation in planar Cu(II) compounds. Only a very small change in d–d excitation energy is required for a significant impact on the rate of spin relaxation. Furthermore, ligand field strength is more readily translated into practical synthetic strategies than theoretical concepts like spin–phonon coupling. By leveraging the ligand field strength design principle, further elongation of *T*_1_ may likely be obtained across a range of paramagnetic complexes.

## Supporting Information Available

Synthesis and characterization methods, powder X-ray diffraction, X-ray crystallography, UV–vis spectroscopy, MCD methods, MCD analysis, EPR methods, and computational methods (PDF)
