# Supplementary material for: A Spectrochemical Series for Electron Spin Relaxation
Source: J Am Chem Soc. 2025 Jan 8;147(3):2849–59. doi: 10.1021/jacs.4c16571 (PMC11760167; doi:10.1021/jacs.4c16571)
Supplement: Supplementary file 1 — ja4c16571_si_001.pdf [file ja4c16571_si_001.pdf]

Supporting Information

For

A Spectrochemical Series for Electron Spin Relaxation

Nathanael P. Kazmierczak,† Kay T. Xia, † Erica Sutcliffe, Jonathan P. Aalto, Ryan G. Hadt*

Division of Chemistry and Chemical Engineering, Arthur Amos Noyes Laboratory of Chemical Physics, California Institute of Technology, Pasadena, California 91125, United States

†Authors contributed equally

*corresponding author: rghadt@caltech.edu

Table of Contents:

1. Synthesis and Characterization Methods 15

1.1 General Methods 15

1.2 Synthetic Methods 16

1.2.1 Synthesis of $K_2[Cu(ox)_2]$ 16

1.2.2 Synthesis of $(PPN)_2[Cu(ox)_2]$ 16

1.2.3 Synthesis of Cu(acacen) 17

1.2.4 Synthesis of $Cu(pci)_2$ 18

1.2.5 Synthesis of $(PPh_4)_2[Cu(mnt)_2]$ 18

1.2.6 Synthesis of $Cu(dtc)_2$ 19

1.2.7 Synthesis of $(PPh_4)_2[Cu(bdt)_2]$ 20

1.3 Sample Preparation for EPR and MCD 21

2. Powder X-Ray Diffraction Data and Refinement 23

3. X-Ray Crystallography Data for $(PPN)_2[Cu(ox)_2]$ 27

4. UV-vis Spectroscopy 29

5. MCD Spectroscopy 33

5.1 MCD Methods 33

5.1.1 Parameters for MCD spectra 40

5.2 MCD Analysis 41

5.3 Polymer Film MCD 45

5.3.1 $(PPh_4)_2[Cu(mnt)_2]$ in PMMA film 45

5.3.2 $Cu(dtc)_2$ in PS film 49

5.3.3 $Cu(pci)_2$ in PS film 52

5.3.4 Cu(acacen) in PS film 55

5.3.5 $Cu(tbaa)_2$ in PS film 58

5.3.6 $Cu(tmhd)_2$ in PMMA film 61

5.3.7 $(PPN)_2[Cu(ox)_2]$ in PS film 65

5.3.8 $Cu(hfac)_2$ hydrate in PS film 66

5.3.9 $K_2[Cu(ox)_2]$ in PVA film 69

5.4 Frozen Solution MCD 70

5.4.1 $(PPh_4)_2[Cu(bdt)_2]$ in 1:1 butyronitrile:DCM frozen solution 70

5.4.2 $Cu(acac)_2$ in 1:1 DCM:toluene frozen solution 72

5.5 Fluorolube Mull MCD 73

5.5.1 $(PPh_4)_2[Cu(mnt)_2]$ Fluorolube mull 73

5.5.2 $Cu(dtc)_2$ in Fluorolube mull 75

5.5.3 $Cu(tmhd)_2$ in Fluorolube mull 77

5.5.4 $Cu(acac)_2$ in Fluorolube mull 79

5.5.5 $Cu(hfac)_2$ hydrate in Fluorolube mull 81

5.5.6 $K_2[Cu(ox)_2]$ hydrate in Fluorolube mull 83

6. EPR Methods **84**
6.1 Spin Hamiltonian Fitting of EPR Spectra 85
6.2 Spin Relaxation EPR Data and Fitting 105
6.3 Orbital Reduction Factors for T_1 Scaling 117
7. Computational Methods **119**
7.1 Analysis of Excited State Energies 119
7.1.1 $K_2[Cu(ox)_2]$ *122*
7.1.2 $K_2[Cu(ox)_2]•2H_2O$ *124*
7.1.3 $(PPN)_2[Cu(ox)_2]$ *126*
7.1.4 $Cu(acac)_2$ *128*
7.1.5 $Cu(tmhd)_2$ *130*
7.1.6 $Cu(hfac)_2$ *132*
7.1.7 $Cu(hfac)_2•H_2O$ *134*
7.1.8 $Cu(hfac)_2•2H_2O$ *136*
7.1.9 $Cu(tbaa)_2$ *138*
7.1.10 $Cu(tbaa)_2•H_2O$ *140*
7.1.11 Cu(acacen) *142*
7.1.12 Cu(acacen) (Constrained Optimization) *144*
7.1.13 $Cu(pci)_2$ *146*
7.1.14 $(PPh_4)_2[Cu(mnt)_2]$ *148*
7.1.15 $(PPh_4)_2[Cu(mnt)_2]$ (Constrained Optimization) *151*
7.1.16 $Cu(dtc)_2$ *154*
7.1.17 $Cu(dtc)_2$ (Constrained Optimization) *157*
7.1.18 $(PPh_4)_2[Cu(bdt)_2]$ *159*
7.1.19 $(PPh_4)_2[Cu(bdt)_2]$ (Constrained Optimization) *162*
7.2 Optimized Coordinates Used for TDDFT Calculations 165
7.3 Example TDDFT Input File 177
7.4 Explicit Solvation Computations 178
7.4.1 Computed g values and Free Energies *180*
7.4.2 Comparison of Methods of Explicit Solvation *182*
7.4.3 Changes in g_z vs. Solvent Coordination *183*
7.5 Computed Excited State Energies and Absorbance Spectra 184
7.5.1 $Cu(acac)_2 + 7H_2O$ *184*
7.5.2 $Cu(hfac)_2 + 7H_2O$ *186*
7.5.3 $Cu(tmhd)_2 + 7H_2O$ *188*
7.5.4 $Cu(acacen) + 7H_2O$ *190*
7.5.5 $Cu(dtc)_2 + 7H_2O$ *192*
7.5.6 $[Cu(mnt)_2]^{2-} + 7H_2O$ *194*
7.5.7 $[Cu(ox)_2]^{2-} + 7H_2O$ *197*
7.5.8 $Cu(hfac)_2 + 7MeOH$ *199*
7.5.9 $[Cu(ox)_2]^{2-} + 7MeOH$ *202*
7.5.10 $Cu(hfac)_2 + 4Toluene$ *204*
7.5.11 $[Cu(ox)_2]^{2-} + 4Toluene$ *207*

7.6 Optimized Geometries 210
8. References 213

List of Figures

Figure S1: PXRD data and Rietveld refinement for $Cu(dtc)_2$ 24
Figure S2: PXRD data and Rietveld refinement for $(PPh_4)_2[Cu(bdt)_2]$. 25
Figure S3: PXRD data and Rietveld refinement for $K_2[Cu(ox)_2]$ 26
Figure S4: Asymmetric unit in the single crystal structure of $(PPN)_2[Cu(ox)_2]$. Thermal ellipsoids are drawn with 50% probability. 28
Figure S5: UV-vis spectrum of $K_2[Cu(ox)_2]$ in water. 29
Figure S6: UV-vis spectrum of $(PPN)_2[Cu(ox)_2]$ in chloroform. 29
Figure S7: UV-vis spectrum of $Cu(acac)_2$ in chloroform. 29
Figure S8: UV-vis spectrum of $Cu(tmhd)_2$ in chloroform. 30
Figure S9: UV-vis spectrum of $Cu(hfac)_2$ in chloroform. 30
Figure S10: UV-vis spectrum of $Cu(tbaa)_2$ in chloroform. 30
Figure S11: UV-vis spectrum of Cu(acacen) in chloroform. 31
Figure S12: UV-vis spectrum of $Cu(pci)_2$ in chloroform. 31
Figure S13: UV-vis spectrum of $(PPh_4)_2[Cu(mnt)_2]$ in chloroform. 31
Figure S14: UV-vis spectrum of $Cu(dtc)_2$ in chloroform. 32
Figure S15: UV-vis spectrum of $(PPh_4)_2[Cu(bdt)_2]$ in chloroform. 32
Figure S16: Block diagram of the homebuilt MCD spectrometer. 33
Figure S17: Nickel tartrate depolarization tests for a PMMA sample. 36
Figure S18: Nickel tartrate depolarization tests for a Fluorolube mull ($K_2[Cu(ox)_2]$ hydrate) with small optical scattering. 37
Figure S19: Nickel tartrate depolarization tests for a Fluorolube mull ($Cu(acac)_2$) with large optical scattering. 37
Figure S20: Plot of $1/T_1$ vs. average d–d transition energy. 43
Figure S21: Log-log plot of $1/T_1$ vs. average d–d transition energy. 43
Figure S22: Plot of $\log(1/T_1)$ vs. average d–d transition energy. 43
Figure S23: Plot of $1/T_1$ vs. $d(x^2–y^2)$ energy. 44
Figure S24: Log-log plot of $1/T_1$ vs. $d(x^2–y^2)$ energy. 44
Figure S25: Plot of $\log(1/T_1)$ vs. $d(x^2–y^2)$ energy. 44
Figure S26: MCD spectra of $(PPh_4)_2[Cu(mnt)_2]$ in PMMA film at 2.0 K (UV-visible region of spectrum). 45
Figure S27: MCD spectra of $(PPh_4)_2[Cu(mnt)_2]$ in PMMA film at 2.0 K (NIR region of spectrum; zoomed in). 45
Figure S28: Gaussian band fits to $(PPh_4)_2[Cu(mnt)_2]$ in PMMA film. MCD is obtained at 2.0 K and +/-0.5 T. 46
Figure S29: Gaussian band fits to $(PPh_4)_2[Cu(mnt)_2]$ in PMMA film, zoomed on NIR region to emphasize band 1. MCD is obtained at 2.0 K and +/-0.5 T. 47
Figure S30: MCD spectra of $Cu(dtc)_2$ in PS film at 5.5 K. 49
Figure S31: MCD spectra of $Cu(dtc)_2$ in PS film at 10.0 K. 49
Figure S32: Pure C-term MCD spectra of $Cu(dtc)_2$ in PS film obtained by subtracting 5.5 K spectra from 10.0 K spectra. 50
Figure S33: Gaussian band fits to $Cu(dtc)_2$ in PS film. MCD is the pure C-term spectrum obtained at +/-2 T by subtracting the 5.5 K spectrum from the 10.0 K spectrum. 50
Figure S34: MCD spectra of $Cu(pci)_2$ in PS film at 5.0 K. 52
Figure S35: MCD spectra of $Cu(pci)_2$ in PS film at 10.0 K. 52

Figure S36: Pure C-term MCD spectra of $Cu(pci)_2$ in PS film obtained by subtracting 5.0 K spectra from 10.0 K spectra. 53
Figure S37: Gaussian band fits to $Cu(pci)_2$ in PS film. MCD is the pure C-term spectrum obtained at +/-4 T by subtracting the 5.0 K spectrum from the 10.0 K spectrum. 53
Figure S38: Gaussian band fits to $Cu(pci)_2$ in PS film (zoomed-in on y-axis). MCD is the pure C-term spectrum obtained at +/-4 T by subtracting the 5.0 K spectrum from the 10.0 K spectrum. 54
Figure S39: MCD spectra of Cu(acacen) in PS film at 5.0 K. 55
Figure S40: MCD spectra of Cu(acacen) in PS film at 10.0 K. 55
Figure S41: Pure C-term MCD spectra of Cu(acacen) in PS film obtained by subtracting 5.0 K spectra from 10.0 K spectra. 56
Figure S42: Gaussian band fits to Cu(acacen) in PS film. MCD is the pure C-term spectrum obtained at +/-2 T by subtracting the 5.0 K spectrum from the 10.0 K spectrum. 56
Figure S43: MCD spectra of $Cu(tbaa)_2$ in PS film at 5.0 K. 58
Figure S44: MCD spectra of $Cu(tbaa)_2$ in PS film at 10.0 K. 58
Figure S45: Pure C-term MCD spectra of $Cu(tbaa)_2$ in PS film obtained by subtracting 5.0 K spectra from 10.0 K spectra. 59
Figure S46: Gaussian band fits to $Cu(tbaa)_2$ in PS film. MCD is the pure C-term spectrum obtained at +/-2 T by subtracting the 5.0 K spectrum from the 10.0 K spectrum. 59
Figure S47: MCD spectra of $Cu(tmhd)_2$ in PMMA film at 5.0 K. 61
Figure S48: MCD spectra of $Cu(tmhd)_2$ in PMMA film at 10.0 K. 61
Figure S49: MCD spectra of $Cu(tmhd)_2$ in PMMA film at 20.0 K. 62
Figure S50: Comparison of MCD spectra of $Cu(tmhd)_2$ in PMMA film at +/-2T. 62
Figure S51: Pure C-term MCD spectra of $Cu(tmhd)_2$ in PMMA film obtained by subtracting 5.0 K spectra from 20.0 K spectra. 63
Figure S52: Gaussian band fits to $Cu(tmhd)_2$ in PMMA film. MCD is the pure C-term spectrum obtained at +/-2 T by subtracting the 5.0 K spectrum from the 20.0 K spectrum. 63
Figure S53: Pure C-term MCD spectra of $(PPN)_2[Cu(ox)_2]$ in PS film obtained by subtracting 5.0 K spectra from 10.0 K spectra. 65
Figure S54: Gaussian band fits to $(PPN)_2[Cu(ox)_2]$ in PS film. MCD is the pure C-term spectrum obtained at +/-4 T by subtracting the 5.0 K spectrum from the 10.0 K spectrum. 65
Figure S55: MCD spectra of $Cu(hfac)_2$ hydrate in PS film at 5.0 K. 66
Figure S56: MCD spectra of $Cu(hfac)_2$ hydrate in PS film at 10.0 K. 66
Figure S57: Pure C-term MCD spectra of $Cu(hfac)_2$ in PS film obtained by subtracting 5.0 K spectra from 10.0 K spectra. 67
Figure S58: Pure C-term MCD spectra of $Cu(hfac)_2$ in PS film obtained by subtracting 5.0 K spectra from 10.0 K spectra (zoomed axes). 67
Figure S59: Gaussian band fits to $Cu(hfac)_2$ in PS film. MCD is the pure C-term spectrum obtained at +/-4 T by subtracting the 5.0 K spectrum from the 10.0 K spectrum. 68
Figure S60: Pure C-term MCD spectra of $K_2[Cu(ox)_2]$ in PVA film obtained by subtracting 5.0 K spectra from 10.0 K spectra. 69
Figure S61: Gaussian band fits to $K_2[Cu(ox)_2]$ in PVA film. MCD is the pure C-term spectrum obtained at +/-4 T by subtracting the 5.0 K spectrum from the 10.0 K spectrum. 69
Figure S62: MCD spectra of $(PPh_4)_2[Cu(bdt)_2]$ in 1:1 butyronitrile:DCM glass at 6.5 K and 20.0 K. 70
Figure S63: Pure C-term MCD spectra of $(PPh_4)_2[Cu(bdt)_2]$ in 1:1 butyronitrile:DCM glass obtained by subtracting 6.5 K spectra from 20.0 K spectra. 70

Figure S64: Comparison of scaled MCD spectra for $(PPh_4)_2[Cu(mnt)_2]$ in PMMA and $(PPh_4)_2[Cu(bdt)_2]$ in 1:1 butyronitrile:DCM glass. 71
Figure S65: Gaussian band fits to $(PPh_4)_2[Cu(bdt)_2]$ in 1:1 butyronitrile:DCM frozen solution. MCD is the pure C-term spectrum obtained at +/-6 T by subtracting the 6.5 K spectrum from the 20.0 K spectrum. 71
Figure S66: MCD spectra of $Cu(acac)_2$ in 1:1 DCM:toluene frozen solution at 5.0 K. 72
Figure S67: Gaussian band fits to $Cu(acac)_2$ in 1:1 DCM:toluene frozen solution. MCD is obtained at 5.0 K and +/-1 T. 72
Figure S68: Comparison of scaled MCD spectra for $(PPh_4)_2[Cu(mnt)_2]$ in PMMA and $(PPh_4)_2[Cu(mnt)_2]$ in Fluorolube mull. Spectrum in PMMA acquired at 2.0 K and +/-0.5 T, while spectrum in Fluorolube mull acquired at 5.0 K and +/-7 T (polynomial baseline subtracted). 73
Figure S69: Gaussian band fits to $(PPh_4)_2[Cu(mnt)_2]$ in Fluorolube mull. MCD acquired at 5.0 K and +/-7 T (polynomial baseline subtracted). 73
Figure S70: Pure C-term MCD spectra of $Cu(dtc)_2$ in Fluorolube mull obtained by subtracting 5.0 K spectra from 20.0 K spectra. 75
Figure S71: Gaussian band fits to $Cu(dtc)_2$ in Fluorolube mull. MCD is the pure C-term spectrum obtained at +/-2 T by subtracting the 5.0 K spectrum from the 20.0 K spectrum. 75
Figure S72: Pure C-term MCD spectra of $Cu(tmhd)_2$ in Fluorolube mull obtained by subtracting 5.0 K spectra from 10.0 K spectra. 77
Figure S73: Gaussian band fits to $Cu(tmhd)_2$ in Fluorolube mull. MCD is the pure C-term spectrum obtained at +/-4 T by subtracting the 5.0 K spectrum from the 10.0 K spectrum. 77
Figure S74: Gaussian band fits to $Cu(tmhd)_2$ in Fluorolube mull (zoomed in). MCD is the pure C-term spectrum obtained at +/-4 T by subtracting the 5.0 K spectrum from the 10.0 K spectrum. 78
Figure S75: MCD spectra of $Cu(acac)_2$ in Fluorolube mull at 2.0 K. 79
Figure S76: Gaussian band fits to $Cu(acac)_2$ in Fluorolube mull. MCD obtained at +/-0.5 T and 2.0 K. 79
Figure S77: Gaussian band fits to $Cu(acac)_2$ in Fluorolube mull (zoomed in). MCD obtained at +/-0.5 T and 2.0 K. 80
Figure S78: Gaussian band fits to $Cu(acac)_2$ in Fluorolube mull (zoomed in further). MCD obtained at +/-0.5 T and 1.8 K. 80
Figure S79: Pure C-term MCD spectra of $Cu(hfac)_2$ hydrate in Fluorolube mull obtained by subtracting 5.0 K spectra from 10.0 K spectra. 81
Figure S80: Gaussian band fits to $Cu(hfac)_2$ hydrate in Fluorolube mull. MCD is the pure C-term spectrum obtained at +/-4 T by subtracting the 5.0 K spectrum from the 10.0 K spectrum. 81
Figure S81: Pure C-term MCD spectra of $K_2[Cu(ox)_2]$ hydrate in Fluorolube mull obtained by subtracting 5.0 K spectra from 20.0 K spectra. 83
Figure S82: Gaussian band fits to $K_2[Cu(ox)_2]$ hydrate in Fluorolube mull. MCD is the pure C-term spectrum obtained at +/-7 T by subtracting the 5.0 K spectrum from the 20.0 K spectrum. 83
Figure S83: X-band CW EPR spectrum and fit for $Cu(dtc)_2$ in PS film. (ν = 9.4148 GHz, 4 G modulation amplitude, 77 K). 87
Figure S84: X-band pulsed EDFS spectrum and fit for $Cu(dtc)_2$ in PS film. (ν = 9.7280 GHz, 100 K). 88
Figure S85: X-band CW EPR spectrum and fit for $Cu(pci)_2$ in PS film. (ν = 9.4148 GHz, 4 G modulation amplitude, 77 K). 89

Figure S86: X-band pulsed EDFS spectrum and fit for $Cu(pci)_2$ in PS film. (ν = 9.7284 GHz, 100 K). 90
Figure S87: X-band CW EPR spectrum and fit for Cu(acacen) in PMMA film. (ν = 9.4101 GHz, 4 G modulation amplitude, 77 K). 91
Figure S88: X-band pulsed EDFS spectrum and fit for Cu(acacen) in PMMA film. (ν = 9.7284 GHz, 100 K). 92
Figure S89: X-band CW EPR spectrum and fit for $Cu(tmhd)_2$ in PMMA film. (ν = 9.3968 GHz, 4 G modulation amplitude, 77 K). 93
Figure S90: X-band pulsed EDFS spectrum and fit for $Cu(tmhd)_2$ in PMMA film. (ν = 9.7284 GHz, 100 K). 94
Figure S91: X-band CW EPR spectrum and fit for $Cu(tbaa)_2$ in PS film. (ν = 9.4007 GHz, 4 G modulation amplitude, 77 K). 95
Figure S92: X-band pulsed EDFS spectrum and fit for $Cu(tbaa)_2$ in PS film. (ν = 9.7162 GHz, 100 K). 96
Figure S93: X-band CW EPR spectrum and fit for $Cu(hfac)_2$ in PS film. (ν = 9.4014 GHz, 4 G modulation amplitude, 77 K). 97
Figure S94: X-band pulsed EDFS spectrum and fit for $Cu(hfac)_2$ in PS film. (ν = 9.7165 GHz, 100 K). 98
Figure S95: X-band CW EPR spectrum and fit for $(PPN)_2[Cu(ox)_2]$ in PS film. (ν = 9.4011 GHz, 4 G modulation amplitude, 77 K). 99
Figure S96: X-band pulsed EDFS spectrum and fit for $(PPN)_2[Cu(ox)_2]$ in PS film. (ν = 9.7161 GHz, 100 K). 100
Figure S97: X-band pulsed EDFS spectrum and fit for $(PPh_4)_2[Cu(mnt)_2]$ in PMMA film. (ν = 9.7161 GHz, 100 K). 101
Figure S98: X-band CW EPR spectrum and fit for $K_2[Cu(ox)_2]$in 30%:70% glycerol:water. (ν = 9.3927 GHz, 4 G modulation amplitude, 77 K). 102
Figure S99: X-band CW EPR spectrum and fit for $(PPh_4)_2[Cu(bdt)_2]$ in 2:1 MeCN:toluene. (ν = 9.3927 GHz, 4 G modulation amplitude, 77 K). 103
Figure S100: X-band pulsed EDFS spectrum and fit for $Cu(acac)_2$ in 3:1 DCM:benzene. (ν = 9.7257 GHz, 100 K). 104
Figure S101: T_1 by inversion recovery for $(PPh_4)_2[Cu(mnt)_2]$ in PMMA film (9.7161 GHz, 3444 G, 100 K). 106
Figure S102: T_m by Hahn-echo decay for $(PPh_4)_2[Cu(mnt)_2]$ in PMMA film (9.7161 GHz, 3444 G, 100 K). 106
Figure S103: T_1 by inversion recovery for $Cu(dtc)_2$ in PS film (9.7280 GHz, 3451 G, 100 K). 107
Figure S104: T_m by Hahn-echo decay for $Cu(dtc)_2$ in PS film (9.7280 GHz, 3451 G, 100 K). . 107
Figure S105: T_1 by inversion recovery for Cu(acacen) in PMMA film (9.7284 GHz, 3410 G, 100 K). 108
Figure S106: T_m by Hahn-echo decay for Cu(acacen) in PMMA film (9.7284 GHz, 3410 G, 100 K). 108
Figure S107: T_1 by inversion recovery for $Cu(pci)_2$ in PS film (9.7284 GHz, 3396 G, 100 K). 109
Figure S108: T_m by Hahn-echo decay for $Cu(pci)_2$ in PS film (9.7284 GHz, 3396 G, 100 K). . 109
Figure S109: T_1 by inversion recovery for $Cu(tbaa)_2$ in PS film (9.7162 GHz, 3382 G, 100 K). 110
Figure S110: T_m by Hahn-echo decay for $Cu(tbaa)_2$ in PS film (9.7162 GHz, 3382 G, 100 K). 110

Figure S111: T_1 by Hahn-echo decay for $Cu(acac)_2$ in 3:1 DCM:benzene frozen glass (9.7257 GHz, 3390 G, 100 K). ... 111
Figure S112: T_1 by inversion recovery for $Cu(tmhd)_2$ in PMMA film (9.7284 GHz, 3392 G, 100 K). ... 112
Figure S113: T_m by Hahn-echo decay for $Cu(tmhd)_2$ in PMMA film (9.7284 GHz, 3392 G, 100 K). ... 112
Figure S114: T_1 by inversion recovery for $(PPN)_2[Cu(ox)_2]$ in PS film (9.7161 GHz, 3386 G, 100 K). ... 113
Figure S115: T_m by Hahn-echo decay for $(PPN)_2[Cu(ox)_2]$ in PS film (9.7161 GHz, 3386 G, 100 K). ... 113
Figure S116: T_1 by inversion recovery for $Cu(hfac)_2$ in PS film (9.7165 GHz, 3372 G, 100 K). ... 114
Figure S117: T_m by Hahn-echo decay for $Cu(hfac)_2$ in PS film (9.7165 GHz, 3372 G, 100 K). 114
Figure S118: T_1 by inversion recovery for $K_2[Cu(ox)_2]$ in 3:7 glycerol:water (9.7174 GHz, 3352 G, 100 K). ... 115
Figure S119: T_m by Hahn-echo decay for $K_2[Cu(ox)_2]$ in 3:7 glycerol:water (9.7174 GHz, 3352 G, 100 K). ... 115
Figure S120: T_1 by inversion recovery for $(PPh_4)_2[Cu(mnt)_2]$ in 2:1 MeCN:toluene (9.7204 GHz, 3448 G, 100 K). ... 116
Figure S121: T_m by Hahn-echo decay for $(PPh_4)_2[Cu(mnt)_2]$ in 2:1 MeCN:toluene (9.7204 GHz, 3448 G, 100 K). ... 116
Figure S122: TDDFT natural transition orbitals for $K_2[Cu(ox)_2]$. ... 122
Figure S123: TDDFT natural transition orbitals for $K_2[Cu(ox)_2]$•$2H_2O$. ... 124
Figure S124: TDDFT natural transition orbitals for $(PPN)_2[Cu(ox)_2]$. ... 126
Figure S125: TDDFT natural transition orbitals for $Cu(acac)_2$. ... 128
Figure S126: TDDFT natural transition orbitals for $Cu(tmhd)_2$. ... 130
Figure S127: TDDFT natural transition orbitals for $Cu(hfac)_2$. ... 132
Figure S128: TDDFT natural transition orbitals for $Cu(hfac)_2$•H_2O. ... 134
Figure S129: TDDFT natural transition orbitals for $Cu(hfac)_2$•$2H_2O$. ... 136
Figure S130: TDDFT natural transition orbitals for $Cu(tbaa)_2$. ... 138
Figure S131: TDDFT natural transition orbitals for $Cu(tbaa)_2$•H_2O. ... 140
Figure S132: TDDFT natural transition orbitals for Cu(acacen). ... 142
Figure S133: TDDFT natural transition orbitals for Cu(acacen) (constrained optimization). ... 144
Figure S134: TDDFT natural transition orbitals for $Cu(pci)_2$. ... 146
Figure S135: TDDFT natural transition orbitals for $(PPh_4)_2[Cu(mnt)_2]$. ... 148
Figure S136: TDDFT natural transition orbitals for $(PPh_4)_2[Cu(mnt)_2]$ (Constrained Optimization). ... 151
Figure S137: TDDFT natural transition orbitals for $Cu(dtc)_2$. ... 154
Figure S138: TDDFT natural transition orbitals for $Cu(dtc)_2$ (Constrained Optimization). ... 157
Figure S139: TDDFT natural transition orbitals for $(PPh_4)_2[Cu(bdt)_2]$. ... 159
Figure S140: TDDFT natural transition orbitals for $(PPh_4)_2[Cu(bdt)_2]$ (Constrained Optimization). ... 162
Figure S141: Sequence of DFT and SOLVATOR calculations for each copper complex. ... 179
Figure S142: Set of copper complexes analyzed under explicit solvation conditions: H_2O → All seven complexes; MeOH → $Cu(hfac)_2$ and $[Cu(ox)_2]^{2-}$; Toluene → $Cu(hfac)_2$ and $[Cu(ox)_2]^{2-}$. ... 179

Figure S143: Change in g_z vs. number of coordinated water molecules. 183
Figure S144: Change in g_z vs. sum of Cu–OH_2 bond lengths. 183
Figure S145: TDDFT natural transition orbitals for [Cu(acac)$_2$ + 7H_2O]. 184
Figure S146: TDDFT natural transition orbitals for [Cu(hfac)$_2$ + 7H_2O]. 186
Figure S147: TDDFT natural transition orbitals for [Cu(tmhd)$_2$ + 7H_2O]. 188
Figure S148: TDDFT natural transition orbitals for [Cu(acacen) + 7H_2O]. 190
Figure S149: TDDFT natural transition orbitals for [Cu(dtc)$_2$ + 7H_2O]. 192
Figure S150: TDDFT natural transition orbitals for [[Cu(mnt)$_2$]$^{2-}$ + 7H_2O]. 194
Figure S151: TDDFT natural transition orbitals for [[Cu(ox)$_2$]$^{2-}$ + 7H_2O]. 197
Figure S152: TDDFT natural transition orbitals for [Cu(hfac)$_2$ + 7MeOH]. 199
Figure S153: TDDFT natural transition orbitals for [[Cu(ox)$_2$]$^{2-}$ + 7MeOH]. 202
Figure S154: TDDFT natural transition orbitals for [Cu(hfac)$_2$ + 4Toluene]. 204
Figure S155: TDDFT natural transition orbitals for [[Cu(ox)$_2$]$^{2-}$ + 4Toluene]. 207

List of Tables

Table S1: Summary of sample preparation methods for all compounds. 21
Table S2: Starting and optimized parameters from Rietveld refinement. 23
Table S3: Crystal data and structure refinement for $(PPN)_2[Cu(ox)_2]$. 27
Table S4: Wavelength-dependent depolarization values obtained from nickel tartrate measurements. 38
Table S5: Temperature and field strengths for MCD spectra corresponding to Figure 4 of the main text. 40
Table S6: Tabulation of fitted ligand field transition energies from MCD spectra for all samples in this study, together with associated spin relaxation rates. 41
Table S7: Tabulation of fitted ligand field C/D ratios from MCD spectra, together with associated spin relaxation rates. 42
Table S8: MCD fit parameters for $(PPh_4)_2[Cu(mnt)_2]$ in PMMA film. Assigned ligand field transitions given in bold. 48
Table S9: MCD fit parameters for $Cu(dtc)_2$ in PS film (see above). Assigned ligand field transitions given in bold. 51
Table S10: MCD fit parameters for $Cu(pci)_2$ in PS film (see above). Assigned ligand field transitions given in bold. 54
Table S11: MCD fit parameters for Cu(acacen) in PS film (see above). Assigned ligand field transitions given in bold. 57
Table S12: MCD fit parameters for $Cu(tbaa)_2$ in PS film (see above). Assigned ligand field transitions given in bold. 60
Table S13: MCD fit parameters for $Cu(tmhd)_2$ in PMMA film (see above). Assigned ligand field transitions given in bold. 64
Table S14: MCD fit parameters for $(PPN)_2[Cu(ox)_2]$ in PS film (see above). Assigned ligand field transitions given in bold. 65
Table S15: MCD fit parameters for $Cu(hfac)_2$ in PS film (see above). Assigned ligand field transitions given in bold. 68
Table S16: MCD fit parameters for $K_2[Cu(ox)_2]$ in PVA film (see above). Assigned ligand field transitions given in bold. 69
Table S17: MCD fit parameters for $(PPh_4)_2[Cu(bdt)_2]$ in 1:1 butyronitrile:DCM frozen solution (see above). Assigned ligand field transitions given in bold. 71
Table S18: MCD fit parameters for $Cu(acac)_2$ in 1:1 DCM:toluene frozen solution (see above). Assigned ligand field transitions given in bold. 72
Table S19: MCD fit parameters for $(PPh_4)_2[Cu(mnt)_2]$ in Fluorolube mull (see above). Assigned ligand field transitions given in bold. 74
Table S20: MCD fit parameters for $Cu(dtc)_2$ in Fluorolube mull (see above). Assigned ligand field transitions given in bold. 76
Table S21: MCD fit parameters for $Cu(tmhd)_2$ in Fluorolube mull (see above). Assigned ligand field transitions given in bold. 78
Table S22: MCD fit parameters for $Cu(acac)_2$ in Fluorolube mull (see above). Assigned ligand field transitions given in bold. 80
Table S23: MCD fit parameters for $Cu(hfac)_2$ hydrate in Fluorolube mull. Assigned ligand field transitions given in bold. 82

Table S24: MCD fit parameters for $K_2[Cu(ox)_2]$ hydrate in Fluorolube mull (see above). Assigned ligand field transitions given in bold ... 83
Table S25: Simulated spin Hamiltonian parameters for CW and pulsed EDFS EPR spectra. ... 85
Table S26: Fitted time constants for pulse EPR samples. T_1 and T_m values reported for measurements at the powder line (peak of maximum intensity), which averages over all orientations of the molecule. T_1 recorded by inversion recovery, while T_m recorded by Hahn echo decay at X-band, 100 K. ... 105
Table S27: Tabulation of orbital reduction factors extracted from EPR g values and MCD excited state energies, according to Equation S11. ... 118
Table S28: Crystal structures used as starting geometries. ... 119
Table S29: TDDFT-calculated excited state d–d transition energies for all compounds. ... 120
Table S30: Comparison of the average d–d transition energies as calculated by TDDFT and as measured by MCD spectroscopy. ... 121
Table S31: Properties used to evaluate covalency. Tabulated average copper–ligand bond lengths in optimized geometries and Loewdin density of excited state acceptor d(xy) orbital. ... 121
Table S32: TDDFT-calculated $K_2[Cu(ox)_2]$ excited states assigned to d-d transitions. ... 122
Table S33: TDDFT-calculated $K_2[Cu(ox)_2]$ UV-vis-NIR absorption spectrum ... 123
Table S34: TDDFT-calculated $K_2[Cu(ox)_2]$•$2H_2O$ excited states assigned to d-d transitions. ... 124
Table S35: TDDFT-calculated $K_2[Cu(ox)_2]$•$2H_2O$ UV-vis-NIR absorption spectrum. ... 125
Table S36: TDDFT-calculated $(PPN)_2[Cu(ox)_2]$ excited states assigned to d-d transitions. ... 126
Table S37: TDDFT-calculated $(PPN)_2[Cu(ox)_2]$ UV-vis-NIR absorption spectrum. ... 127
Table S38: TDDFT-calculated $Cu(acac)_2$ excited states assigned to d-d transitions ... 128
Table S39: TDDFT-calculated $Cu(acac)_2$ UV-vis-NIR absorption spectrum. ... 129
Table S40: TDDFT-calculated $Cu(tmhd)_2$ excited states assigned to d-d transitions. ... 130
Table S41: TDDFT-calculated $Cu(tmhd)_2$ UV-vis-NIR absorption spectrum. ... 131
Table S42: TDDFT-calculated $Cu(hfac)_2$ excited states assigned to d-d transitions. ... 132
Table S43: TDDFT-calculated $Cu(hfac)_2$ UV-vis-NIR absorption spectrum ... 133
Table S44: TDDFT-calculated $Cu(hfac)_2$•H_2O excited states assigned to d-d transitions. ... 134
Table S45: TDDFT-calculated $Cu(hfac)_2$•H_2O UV-vis-NIR absorption spectrum. ... 135
Table S46: TDDFT-calculated $Cu(hfac)_2$•$2H_2O$ excited states assigned to d-d transitions. ... 136
Table S47: TDDFT-calculated $Cu(hfac)_2$•$2H_2O$ UV-vis-NIR absorption spectrum. ... 137
Table S48: TDDFT-calculated $Cu(tbaa)_2$ excited states assigned to d-d transitions. ... 138
Table S49: TDDFT-calculated $Cu(tbaa)_2$ UV-vis-NIR absorption spectrum. ... 139
Table S50: TDDFT-calculated $Cu(tbaa)_2$•H_2O excited states assigned to d-d transitions. ... 140
Table S51: TDDFT-calculated $Cu(tbaa)_2$•H_2O UV-vis-NIR absorption spectrum. ... 141
Table S52: TDDFT-calculated Cu(acacen) excited states assigned to d-d transitions. ... 142
Table S53: TDDFT-calculated Cu(acacen) UV-vis-NIR absorption spectrum. ... 143
Table S54: TDDFT-calculated Cu(acacen) (constrained optimization) excited states assigned to d-d transitions ... 144
Table S55: TDDFT-calculated Cu(acacen) (constrained optimization) UV-vis-NIR absorption spectrum. ... 145
Table S56: TDDFT-calculated $Cu(pci)_2$ excited states assigned to d-d transitions. ... 146
Table S57: TDDFT-calculated $Cu(pci)_2$ UV-vis-NIR absorption spectrum ... 147
Table S58: TDDFT-calculated $(PPh_4)_2[Cu(mnt)_2]$ excited states assigned to d-d transitions. ... 148
Table S59: TDDFT-calculated $(PPh_4)_2[Cu(mnt)_2]$ UV-vis-NIR absorption spectrum. ... 149

Table S60: TDDFT-calculated $(PPh_4)_2[Cu(mnt)_2]$ (Constrained Optimization) excited states assigned to d-d transitions. 151
Table S61: TDDFT-calculated $(PPh_4)_2[Cu(mnt)_2]$ (Constrained Optimization) UV-vis-NIR absorption spectrum. 152
Table S62: TDDFT-calculated $Cu(dtc)_2$ excited states assigned to d-d transitions. 154
Table S63: TDDFT-calculated $Cu(dtc)_2$ UV-vis-NIR absorption spectrum. 155
Table S64: TDDFT-calculated $Cu(dtc)_2$ (Constrained Optimization) excited states assigned to d-d transitions. 157
Table S65: TDDFT-calculated $Cu(dtc)_2$ (Constrained Optimization) UV-vis-NIR absorption spectrum. 158
Table S66: TDDFT-calculated $(PPh_4)_2[Cu(bdt)_2]$ excited states assigned to d-d transitions. 159
Table S67: TDDFT-calculated $(PPh_4)_2[Cu(bdt)_2]$ UV-vis-NIR absorption spectrum. 160
Table S68: TDDFT-calculated $(PPh_4)_2[Cu(bdt)_2]$ (Constrained Optimization) excited states assigned to d-d transitions. 162
Table S69: TDDFT-calculated $(PPh_4)_2[Cu(bdt)_2]$ (Constrained Optimization) UV-vis-NIR absorption spectrum. 163
Table S70: g values, Gibbs free energies (GFE), and solvent coordination values for $Cu(acac)_2$. 180
Table S71: g values, Gibbs free energies (GFE), and solvent coordination values for $Cu(hfac)_2$. 180
Table S72: g values, Gibbs free energies (GFE), and solvent coordination values for $Cu(tmhd)_2$. 180
Table S73: g values, Gibbs free energies (GFE), and solvent coordination values for $Cu(acacen)_2$. 180
Table S74: g values, Gibbs free energies (GFE), and solvent coordination values for $Cu(dtc)_2$. 181
Table S75: g values, Gibbs free energies (GFE), and solvent coordination values for $[Cu(mnt)_2]^{2-}$. 181
Table S76: g values, Gibbs free energies (GFE), and solvent coordination values for $[Cu(ox)_2]^{2-}$. 181
Table S77: Solvent coordination comparison for methods 1 (D) and 2 (F). 182
Table S78: TDDFT-calculated $[Cu(acac)_2 + 7H_2O]$ excited states assigned to d-d transitions. 184
Table S79: TDDFT-calculated $[Cu(acac)_2 + 7H_2O]$ UV-vis-NIR absorption spectrum. 185
Table S80: TDDFT-calculated $[Cu(hfac)_2 + 7H_2O]$ excited states assigned to d-d transitions. 186
Table S81: TDDFT-calculated $[Cu(hfac)_2 + 7H_2O]$ UV-vis-NIR absorption spectrum. 187
Table S82: TDDFT-calculated $[Cu(tmhd)_2 + 7H_2O]$ excited states assigned to d-d transitions. 188
Table S83: TDDFT-calculated $[Cu(tmhd)_2 + 7H_2O]$ UV-vis-NIR absorption spectrum. 189
Table S84: TDDFT-calculated $[Cu(acacen) + 7H_2O]$ excited states assigned to d-d transitions. 190
Table S85: TDDFT-calculated $[Cu(acacen) + 7H_2O]$ UV-vis-NIR absorption spectrum. 191
Table S86: TDDFT-calculated $[Cu(dtc)_2 +7H_2O]$ excited states assigned to d-d transitions. 192
Table S87: TDDFT-calculated $[Cu(dtc)_2 + 7H_2O]$ UV-vis-NIR absorption spectrum. 193
Table S88: TDDFT-calculated $[[Cu(mnt)_2]^{2-} + 7H_2O]$ excited states assigned to d-d transitions. 194
Table S89: TDDFT-calculated $[[Cu(mnt)_2]^{2-} + 7H_2O]$ UV-vis-NIR absorption spectrum. 195
Table S90: TDDFT-calculated $[[Cu(ox)_2]^{2-} + 7H_2O]$ excited states assigned to d-d transitions. 197

Table S91: TDDFT-calculated [[Cu(ox)$_2$]$^{2-}$ + 7H_2O] UV-vis-NIR absorption spectrum 198
Table S92: TDDFT-calculated [Cu(hfac)$_2$ + 7MeOH] excited states assigned to d-d transitions. 199
Table S93: TDDFT-calculated [Cu(hfac)$_2$ + 7MeOH] UV-vis-NIR absorption spectrum......... 200
Table S94: TDDFT-calculated [[Cu(ox)$_2$]$^{2-}$ + 7MeOH] excited states assigned to d-d transitions. 202
Table S95: TDDFT-calculated [[Cu(ox)$_2$]$^{2-}$ + 7MeOH] UV-vis-NIR absorption spectrum. 203
Table S96: TDDFT-calculated [Cu(hfac)$_2$ +4Toluene] excited states assigned to d-d transitions. 204
Table S97: TDDFT-calculated [Cu(hfac)$_2$ + 4Toluene] UV-vis-NIR absorption spectrum. 205
Table S98: TDDFT-calculated [[Cu(ox)$_2$]$^{2-}$ + 4Toluene] excited states assigned to d-d transitions. 207
Table S99: TDDFT-calculated [[Cu(ox)$_2$]$^{2-}$ + 4Toluene] UV-vis-NIR absorption spectrum. ... 208
Table S100: Optimized DFT structures for copper complexes, from methods 1 (**D**) and 2 (**F**). 210

1. Synthesis and Characterization Methods

1.1 General Methods

Unless otherwise noted, all reactions were carried out in oven-dried glassware sealed with rubber septa under a nitrogen atmosphere with Teflon-coated magnetic stir bars. Reagents were purchased from Sigma Aldrich and Strem Chemicals and used without further purification unless otherwise noted. Ultraviolet-visible (UV-vis) spectroscopy was conducted using an Agilent 8453 UV-vis spectrophotometer in the Beckman Institute Laser Resource Center. Electron paramagnetic resonance (EPR) experiments were conducted using the Caltech Beckman Institute EPR facility. Continuous wave (CW) EPR spectroscopy was conducted using a Bruker EMX X-band CW EPR spectrometer at 77 K in a liquid nitrogen immersion dewar. Pulse EPR spectroscopy was conducted using a Bruker ELEXSYS E580 pulse EPR spectrometer equipped with a liquid nitrogen flow cryostat system. X-ray crystallography was conducted at the Caltech Beckman Institute X-Ray Crystallography Facility. Single crystal X-Ray diffraction data were collected using a Bruker AXS D8 Venture four-circle X-ray diffractometer with a photon II detector. Powder X-ray diffraction (PXRD) data were collected using a Rigaku SmartLab diffractometer. PXRD data were fit to reported data using Argonne National Laboratory's General Structure Analysis System-II (GSAS-II).

Methods and procedures for magnetic circular dichroism (MCD) and electron paramagnetic resonance (EPR) are respectively described in detail in the MCD methods and EPR methods sections of the supporting information below.

1.2 Synthetic Methods

The following compounds were purchased from Strem Chemicals:

- Copper (II) bis(acetylacetonate) [Cu(acac)$_2$]
- Copper (II) bis(hexafluoroacetylacetonate) [Cu(hfac)$_2$]
- Copper (II) bis(2,2,6,6-tetramethylheptane-3,5-dione) [Cu(tmhd)$_2$]
- Copper (II) bis(tert-butylacetoacetate) [Cu(tbaa)$_2$]

Copper salts used as reagents in the syntheses below were purchased from Strem Chemicals. All other chemical reagents were purchased from Sigma Aldrich.

1.2.1 Synthesis of $K_2[Cu(ox)_2]$

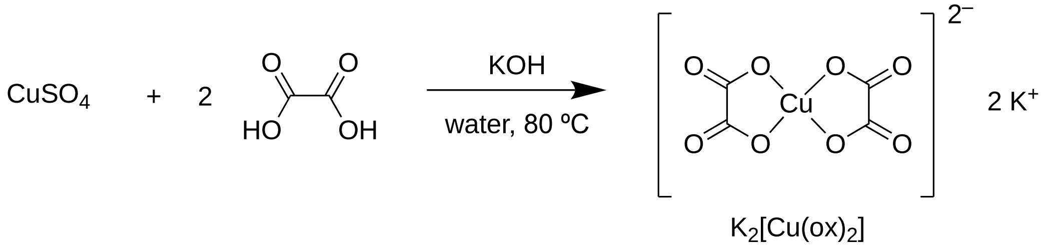

The synthesis of potassium copper (II) bisoxalate [$K_2[Cu(ox)_2]$] was adapted from a reported procedure.[1] In a 20-mL scintillation vial equipped with a magnetic stir bar, 1.41 g potassium hydroxide (25.1 mmol., 8 equiv.) and 1.13 g oxalic acid (12.5 mmol., 4 equiv.) were dissolved in 5 mL water. Once the solution was homogeneous, 500 mg copper (II) sulfate (3.13 mmol., 1 equiv.) was added to the vial. The reaction solution was heated to 80 ºC and stirred overnight for 18 hrs, then cooled to room temperature and filtered over a glass frit to obtain insoluble $K_2[Cu(ox)_2]{\bullet}x(H_2O)$ as a blue crystalline solid (833 mg 84% yield). The product was characterized by PXRD and CW EPR in comparison to literature reports (see **SI Sections 2** and **6.1** below). According to the PXRD data fitting, a mixture of $K_2[Cu(ox)_2]{\bullet}2(H_2O)$ (CCDC identifier KCUOXD02) and $K_2[Cu(ox)_2]{\bullet}4(H_2O)$ (CCDC identifier RIZQIA) were present in the product.

1.2.2 Synthesis of $(PPN)_2[Cu(ox)_2]$

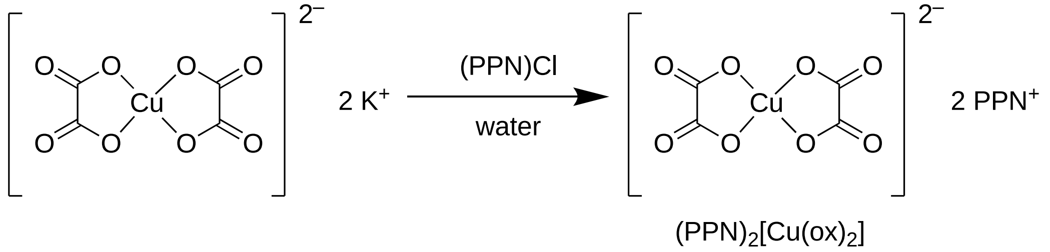

Bis(triphenylphosphine)iminium copper (II) bisoxalate [$(PPN)_2[Cu(ox)_2]$] was synthesized from $K_2[Cu(ox)_2]$ using an ion exchange procedure. 100 mg $K_2[Cu(ox)_2]$ (0.28 mmol., 1 equiv.) and 341 mg bis(triphenylphosphine)iminium (PPN) chloride (0.59 mmol., 2.1 equiv.) were dissolved

in 10 mL water in a 20-mL scintillation vial equipped with a magnetic stir bar. A blue precipitate formed. After 1 hr., the solution was extracted with 10 mL dichloromethane. The blue organic layer was separated and layered with hexanes. After crystallization overnight at room temperature, blue block crystals suitable for single crystal X-ray diffraction were obtained (238 mg, 64% yield). Structural data for this compound is included in **SI Section 3** below. CW EPR data for this compound is included in **SI Section 6.1**.

1.2.3 Synthesis of Cu(acacen)

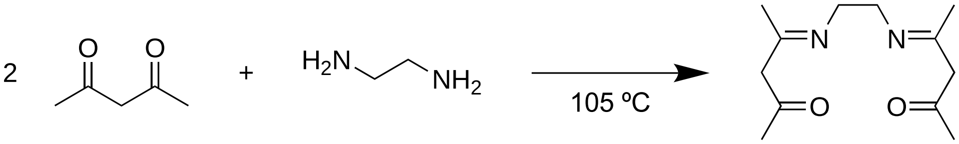

acacenH$_2$

The synthesis of *N,N'*-bis(acetylacetone)-1,2-ethylenediimine (acacenH$_2$) was adapted from a reported procedure.[2] 2.00 g acetylacetone (2.04 mL, 20 mmol., 2 equiv.) was added to a 50-mL round bottom flask equipped with a magnetic stir bar. 600 mg ethylenediamine (0.67 mL, 10 mmol., 1 equiv.) was added dropwise to the flask. The reaction mixture was heated in an oil bath at 105 ºC overnight for 18 hrs, then cooled to room temperature. The red-orange mixture was filtered over a glass frit to collect a light-yellow crystalline precipitate. The precipitate was dissolved in 20 mL dichloromethane and dried over magnesium sulfate. The solvent was then removed by rotary evaporation. The crystalline solids were then recrystallized by redissolving in 10 mL dichloromethane and layering with hexanes. After crystallization overnight at room temperature, the light-yellow crystalline product acacenH$_2$ was dried and used in the next step without further purification (1.856 g, 83% yield).

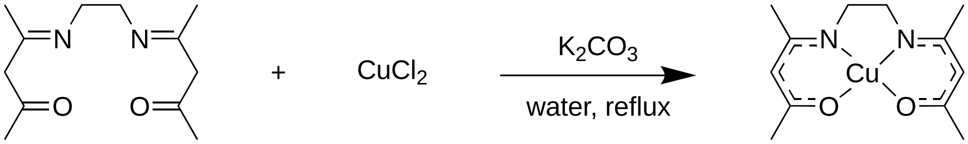

Cu(acacen)

The synthesis of Cu(acacen) was adapted from a reported procedure.[3] 100 mg acacenH$_2$ (0.45 mmol., 1 equiv.) and 203 mg potassium carbonate were dissolved in 5 mL water in a 2-necked 25-mL round bottom flask equipped with a magnetic stir bar and a reflux condenser and heated to reflux. Then, a solution of 60 mg copper (II) chloride in 1 mL water was added. A purple solid precipitated. The mixture was stirred at reflux for 1 hr, then allowed to cool to room temperature and filtered through a glass frit. The purple powdered precipitate was dissolved in 5 mL acetone and recrystallized by slow addition of water to yield a purple microcrystalline powder (98 mg, 77% yield). The product was characterized by CW EPR in comparison to literature reports (see **SI Section 6.1** below).

1.2.4 Synthesis of Cu(pci)₂

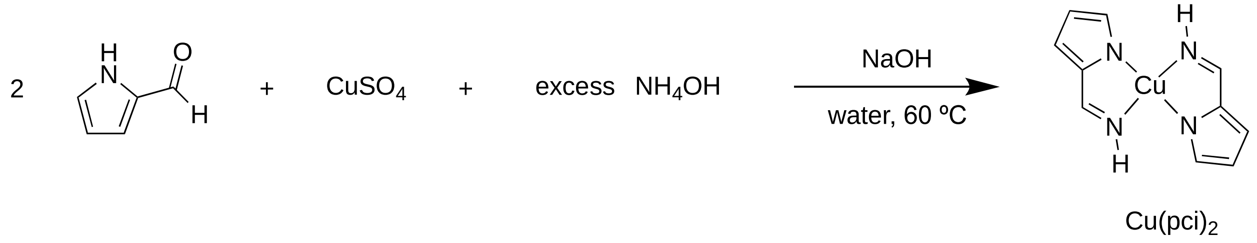

The synthesis of copper (II) bis(pyrrole-2-iminaldehyde) [Cu(pci)$_2$] was adapted from a reported procedure.[4] 400 mg pyrrole-2-carboxaldehyde (4.20 mmol., 2 equiv.) was dissolved in 6 mL water in a 25-mL round bottom flask equipped with a magnetic stir bar, and heated to 60 ºC in an oil bath. 336 mg copper (II) sulfate (2.10 mmol., 1 equiv.) was dissolved in 5 mL of a 10% v/v solution of ammonium hydroxide in water. This blue solution was added to the warm pyrrole solution. The mixture was allowed to stand for 10 minutes. A solution of 170 mg sodium hydroxide (4.20 mmol., 2 equiv.) dissolved in 6 mL water was added dropwise. The solution was allowed to stand for 1hr. A sparkly crystalline precipitate formed. The solution was filtered over a glass frit to collect the solids, which were then washed with water. The red solids were recrystallized from ~20 mL hot methanol (848 mg, 81% yield). The product was characterized by CW EPR in comparison to literature reports (see **SI Section 6.1** below).

1.2.5 Synthesis of (PPh$_4$)$_2$[Cu(mnt)$_2$]

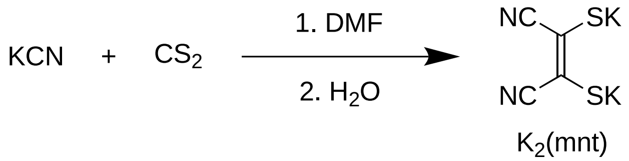

<u>WARNING</u>: this procedure uses potassium cyanide, and all cyanide-containing waste should be disposed of in a separate container. Care should be taken to avoid acidification of any cyanide-containing solutions or waste.

The synthesis of dipotassium maleonitriledithiolate [K$_2$(mnt)] was adapted from a reported procedure.[5] 13.33 g potassium cyanide (205 mmol., 1.03 equiv.) and 60 mL dimethylformamide were added to a 500-mL round bottom flask equipped with a magnetic stir bar and cooled in an ice bath. Under nitrogen on a Schlenk line, 12 mL carbon disulfide (200 mmol., 1 equiv.) was added dropwise using a syringe. The mixture turned a dark reddish brown color and was stirred for 2 hours. The flask was opened to air and 150 mL isopropanol was added. The mixture was heated to 75 ºC in an oil bath while stirring, then filtered while hot to remove residual undissolved potassium cyanide. The filtrate was cooled in a dry ice and acetone bath, and then filtered while cold and washed with diethyl ether to obtain a pale yellow powder. These solids were then dissolved in 400 mL water and stirred for 18 hrs overnight. The resulting dark yellow solution was then filtered to remove elemental sulfur. The filtrate was dried under rotary evaporation. The residual solids were redissolved in 200 mL hot ethanol (~75 ºC), which was then filtered to obtain a bright yellow crystalline powder. 80 mL diethyl ether was added to the filtrate, which was then

cooled in an ice bath. The precipitate was collected by filtration, and then combined with the bright yellow precipitate from the previous filtration. These solids were redissolved in 150 mL hot ethanol, and then filtered again to obtain a yellow crystalline powder. 60 mL diethyl ether was added to the filtrate, which was again cooled in an ice bath and then filtered again. The yellow precipitates from both filtration steps were combined and dried under vacuum to obtain the product (12.535 g, 28% yield).

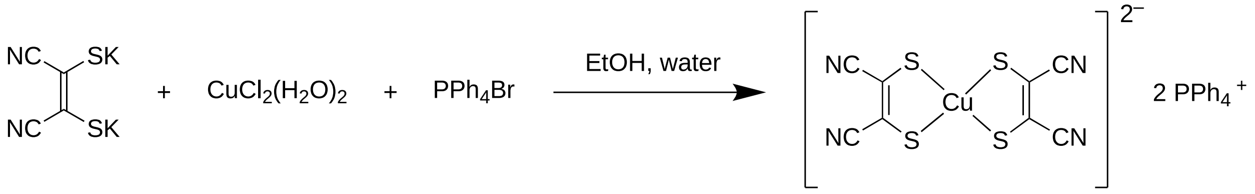

$(PPh_4)_2[Cu(mnt)_2]$

The synthesis of bis(tetraphenylphosphonium) copper (II) bis(maleonitriledithiolate) $[(PPh_4)_2[Cu(mnt)_2]]$ was adapted from a reported procedure.[6] 100 mg $[K_2(mnt)]$ (0.46 mmol., 2 equiv.) was dissolved in a mixture of 2 mL ethanol and 1 mL water in a 20-mL scintillation vial equipped with a magnetic stir bar. 39 mg copper (II) chloride dihydrate (0.23 mmol., 1 equiv.) was dissolved in 2 mL ethanol and then added to the vial as a solution. After stirring for 15 minutes, 192 mg tetraphenylphosphonium bromide (0.46 mmol., 2 equiv.) was dissolved in 6 mL ethanol and added to the vial as a solution. The reaction mixture was stirred for another 5 minutes, during which a precipitate formed. The precipitate was collected by filtration, washed with ethanol, and dried under vacuum to obtain the product as a dark red powder (250 mg, 91% yield). The product was characterized by EPR in comparison to literature reports (see **SI Section 6.1** below).

1.2.6 Synthesis of $Cu(dtc)_2$

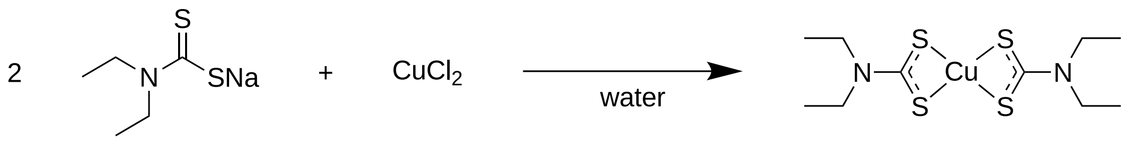

$Cu(dtc)_2$

The synthesis of copper (II) bis(diethyldithiocarbamate) $[Cu(dtc)_2]$ was adapted from a reported procedure.[7] 100 mg sodium diethyldithiocarbamate [Na(dtc)] (0.58 mmol., 2 equiv.) was dissolved in 2 mL water in a 20-mL scintillation vial equipped with a magnetic stir bar. A solution of 39 mg copper (II) chloride (0.29 mmol., 1 equiv.) in 2 mL water was added and the solution was stirred for 1 hr. at room temperature (22 ºC), and then filtered over a glass frit. The dark precipitate was dissolved in 5 mL dichloromethane and crystallized by layering with hexanes to obtain the product as a black crystalline solid (95 mg, 90% yield). The product was characterized by PXRD and CW EPR in comparison to literature reports (see **SI Sections 2** and **6.1** below).

1.2.7 Synthesis of $(PPh_4)_2[Cu(bdt)_2]$

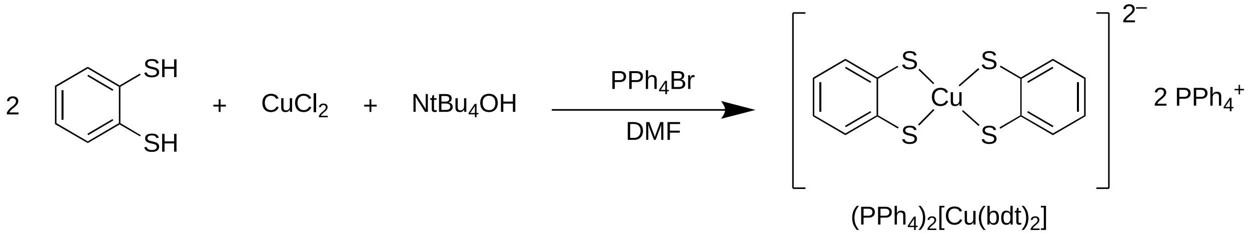

The synthesis of bis(tetraphenylphosphonium) copper (II) bis(benzene-dithiolate) $[(PPh_4)_2[Cu(bdt)_2]]$ was adapted from a reported procedure.[8] $[(PPh_4)_2[Cu(bdt)_2]]$ is air sensitive and was synthesized using Schlenk techniques and subsequently stored and handled under a nitrogen atmosphere inside a glovebox. 65 mg copper (II) chloride (0.48 mmol., 1 equiv.) and 145 mg dithiobenzene (1.02 mmol., 2.1 equiv.) were dissolved in 15 mL dimethylformamide in a 20-mL scintillation vial equipped with a magnetic stir bar. 2 mL of a 1.0 M solution of tetrabutylammonium hydroxide in methanol was added dropwise. The solution turned dark red. The mixture was stirred for 30 minutes at room temperature (22 ºC). 420 mg tetraphenylphosphonium bromide (1.00 mmol., 2.1 equiv.) was added to the solution, which was then stirred for another 2 hrs before being cooled in a –35 ºC freezer overnight for 18 hrs. The cold solution was then filtered over a glass frit inside a glovebox and washed with diethyl ether to obtain a dark red microcrystalline solid (445 mg, 92% yield). The product was characterized by PXRD and CW EPR in comparison to literature reports (see **SI Sections 2** and **6.1** below).

1.3 Sample Preparation for EPR and MCD

Table S1 shows the sample preparation method used for each compound for EPR and MCD.

Table S1: Summary of sample preparation methods for all compounds.

**Compound**	**EPR sample**	**MCD sample**
$K_2[Cu(ox)_2]$	--	Mull
$K_2[Cu(ox)_2]$	Frozen solution	PVA film
$(PPN)_2[Cu(ox)_2]$	PS film	PS film
$Cu(acac)_2$	--	Mull
$Cu(acac)_2$	Frozen solution	Frozen solution
$Cu(tmhd)_2$	--	Mull
$Cu(tmhd)_2$	PMMA film	PMMA film
$Cu(hfac)_2$	PS film	PS film
$Cu(tbaa)_2$	PS film	PS film
Cu(acacen)	PS film	PS film
$Cu(pci)_2$	PS film	PS film
$(PPh_4)_2[Cu(mnt)_2]$	--	Mull
$(PPh_4)_2[Cu(mnt)_2]$	PMMA film	PMMA film
$Cu(dtc)_2$	--	Mull
$Cu(dtc)_2$	--	PMMA film
$Cu(dtc)_2$	PS film	PS film
$(PPh_4)_2[Cu(bdt)_2]$	Frozen solution	Frozen solution

Preparation of mull samples

10 mg of polycrystalline $Cu(acac)_2$ powder was ground using a small agate mortar and pestle for about 10 minutes. Then, three drops (~0.2 mL) Fluorolube oil was added to the mortar, and further ground with the $Cu(acac)_2$ for another 10 minutes until the mixture looked like a homogenous paste, consisting of very small particles of $Cu(acac)_2$ suspended in the Fluorolube. The paste was spread onto a 15 mm diameter quartz disc (1 mm thick) using a spatula, and then another 15 mm quartz disc was placed on top. The "sandwiched" mull could then be used for measurement by MCD. The same procedure was repeated for $K_2[Cu(ox)_2]$, $Cu(tmhd)_2$, $(PPh_4)_2[Cu(mnt)_2]$, and $Cu(dtc)_2$.

Preparation of PVA film sample

2 g polyvinylalcohol (PVA) pellets was mixed with 10 mL water in a 20-mL scintillation vial and heated at 80 ºC for 10 hrs to form a homogenous solution. Unused polymer solution was discarded after 3 days, as older polymer solutions would thicken and develop a gel-like film on the surface. 5 mg $K_2[Cu(ox)_2]$ was dissolved in 1 mL water, and then mixed thoroughly with 3 mL PVA solution. A pipette was used to drop-cast approximately 0.2 mL of this mixture onto a 15 mm diameter quartz disc. The disc was covered by a tent made out of aluminum foil and allowed to dry slowly. After approximately 2 hrs, the film was dry and ready for measurement by MCD.

Preparation of PMMA film samples

2 g polymethylmethacrylate (PMMA) powder was mixed with 10 mL chloroform in a 20-mL scintillation vial and allowed to sit for 12 hrs to form a homogenous solution. Unused polymer solution was discarded after 3 days, as older polymer solutions would thicken and develop a gel-like film on the surface. 5 mg of $Cu(tmhd)_2$, $(PPh_4)_2[Cu(mnt)_2]$ or $Cu(dtc)_2$ respectively was dissolved in 1 mL chloroform, and then mixed thoroughly with 3 mL PMMA solution. A pipette was used to drop-cast approximately 0.2 mL of this mixture onto a 15 mm diameter quartz disc. The disc was covered by a tent made out of aluminum foil and allowed to dry slowly. After approximately 2 hrs, the film was dry and ready for measurement by MCD. For solutions that were strongly absorptive, dilutions were performed by adding more PMMA solution to the copper compound PMMA solution described above, and then recasting the diluted solution on a quartz disc.

Preparation of PS film samples

2 g polystyrene (PS) pellets was mixed with 10 mL chloroform in a 20-mL scintillation vial and allowed to sit for 12 hrs to form a homogenous solution. Unused polymer solution was discarded after 3 days, as older polymer solutions would thicken and develop a gel-like film on the surface. 5 mg of $(PPN)_2[Cu(ox)_2]$, $Cu(hfac)_2$, $Cu(tbaa)_2$ Cu(acacen), $Cu(pci)_2$, or $Cu(dtc)_2$ respectively was dissolved in 1 mL chloroform, and then mixed thoroughly with 3 mL PS solution. A pipette was used to drop-cast approximately 0.2 mL of this mixture onto a 15 mm diameter quartz disc. The disc was covered by a tent made out of aluminum foil and allowed to dry slowly. After approximately 2 hrs, the film was dry and ready for measurement by MCD. For solutions that were strongly absorptive, dilutions were performed by adding more PS solution to the copper compound PS solution described above, and then recasting the diluted solution on a quartz disc.

General method for preparation of polymer film EPR samples

Once dried, the polymer films could be removed in one piece from the supporting quartz disc by sliding a razor blade between the film and the disc. The polymer film was then sliced into strips of approximately 2 mm in width, which were slid into a quartz X-band EPR tube. The tube was tapped gently on the benchtop to settle all the polymer strips at the bottom of the tube. A small amount of Kimwipe tissue was inserted into the tube opening as a permeable stopper. CW and pulse EPR experiments were performed directly on the polymer film strips, which behave similarly to a frozen solution environment.

2. Powder X-Ray Diffraction Data and Refinement

Powder X-ray diffraction (PXRD) was collected on samples in air using a Rigaku SmartLab diffractometer. The diffraction patterns were fit with the Rietveld method using the General Structure Analysis System II (GSAS-II).[9,10] Line broadening was accounted for by domain size, and orientation selection was accounted for by spherical harmonic preferred orientation model. Sample displacement was used to account for spectrum shifts.

$Cu(dtc)_2$ and $(PPh_4)_2[Cu(bdt)_2]$ refined well to reported literature structures. $K_2[Cu(ox)_2]$ contained a mix of hydration states, corresponding to two different reported structures. The KCUOXD02 structure corresponds to the formula $K_2[Cu(ox)_2]•2H_2O$, and the two waters are both axially bound to the copper. The RIZQIA structure corresponds to the formula $K_2[Cu(ox)_2]•4H_2O$, but none of the waters in the structure are bound to the copper.

Table S2: Starting and optimized parameters from Rietveld refinement.

Compound (CCDC identifier)	Space Group		a(Å)	b(Å)	c(Å)	α(°)	β(°)	γ(°)	wR (%)
$Cu(dtc)_2$ (CETCAM01)	P21/c	initial	9.90	10.61	15.50	90.00	102.00	90.00	10.66
		refined	9.90	10.64	16.59	90.00	113.83	90.00	
$(PPh_4)_2[Cu(bdt)_2]$ (QOXKEV)	P21/n	initial	11.14	14.78	14.95	90.00	95.01	90.00	12.80
		refined	11.15	14.77	14.98	90.00	94.98	90.00	
$K_2[Cu(ox)_2]$ (KCUOXD02, RIZQIA)	P-1	initial	6.94	8.70	9.02	108.25	99.96	97.19	12.09
		refined	6.94	8.71	9.01	108.25	99.98	97.24	
	P21/n	initial	3.78	14.82	10.76	90.00	93.18	90.00	
		refined	3.78	14.82	10.76	90.00	93.14	90.00	

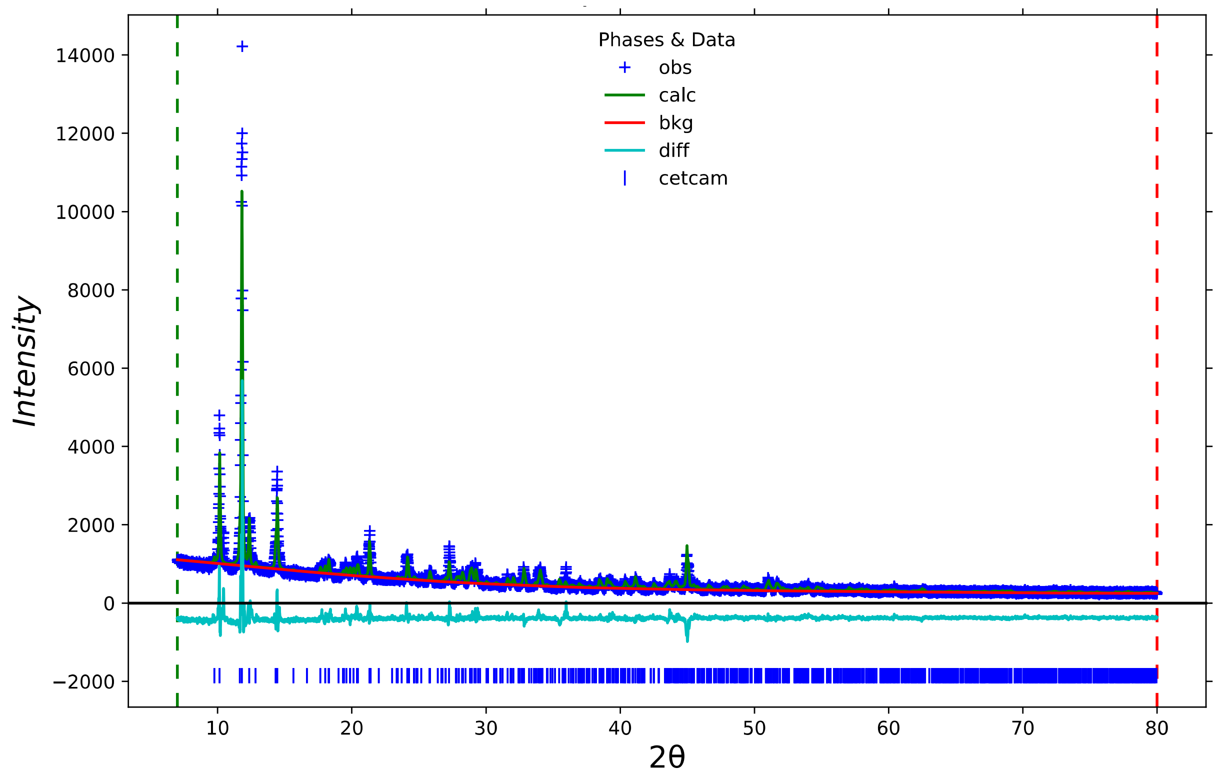

Figure S1: PXRD data and Rietveld refinement for $Cu(dtc)_2$.

Dark blue = experimental data; green = simulation; teal = residuals; red = baseline.

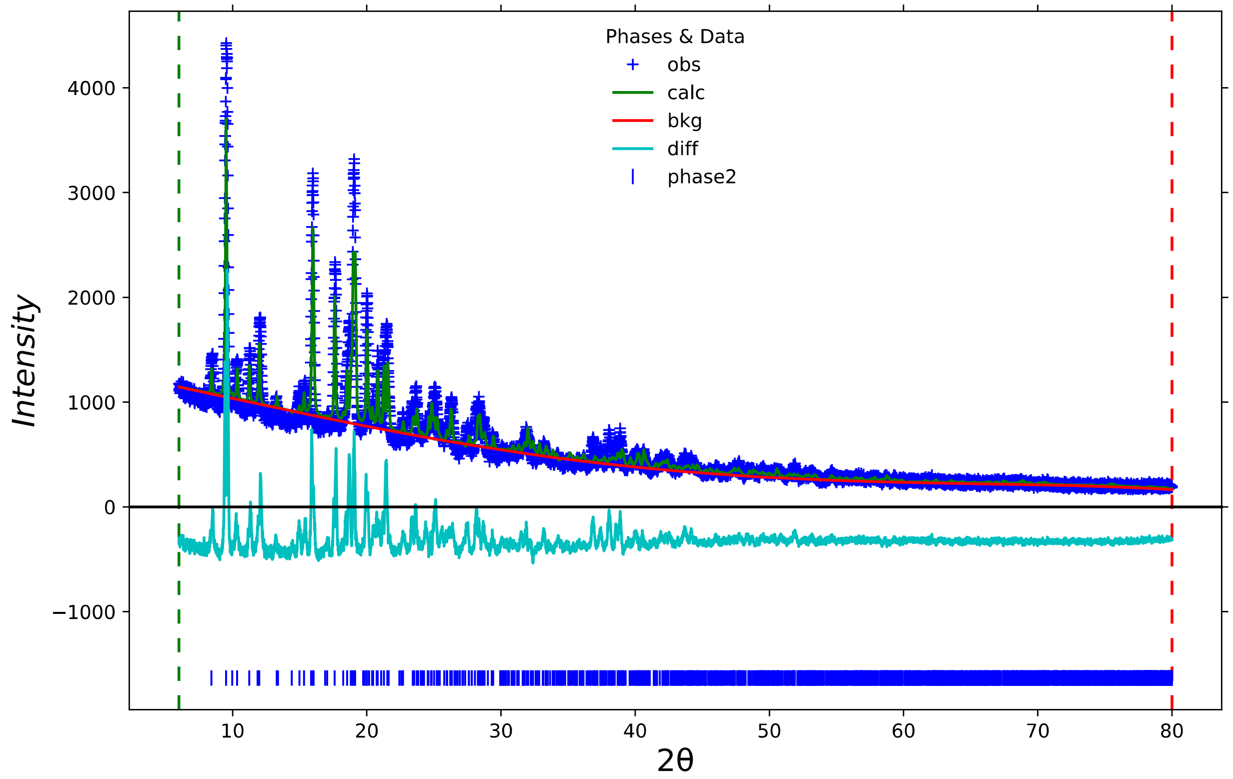

Figure S2: PXRD data and Rietveld refinement for $(PPh_4)_2[Cu(bdt)_2]$.

Dark blue = experimental data; green = simulation; teal = residuals; red = baseline.

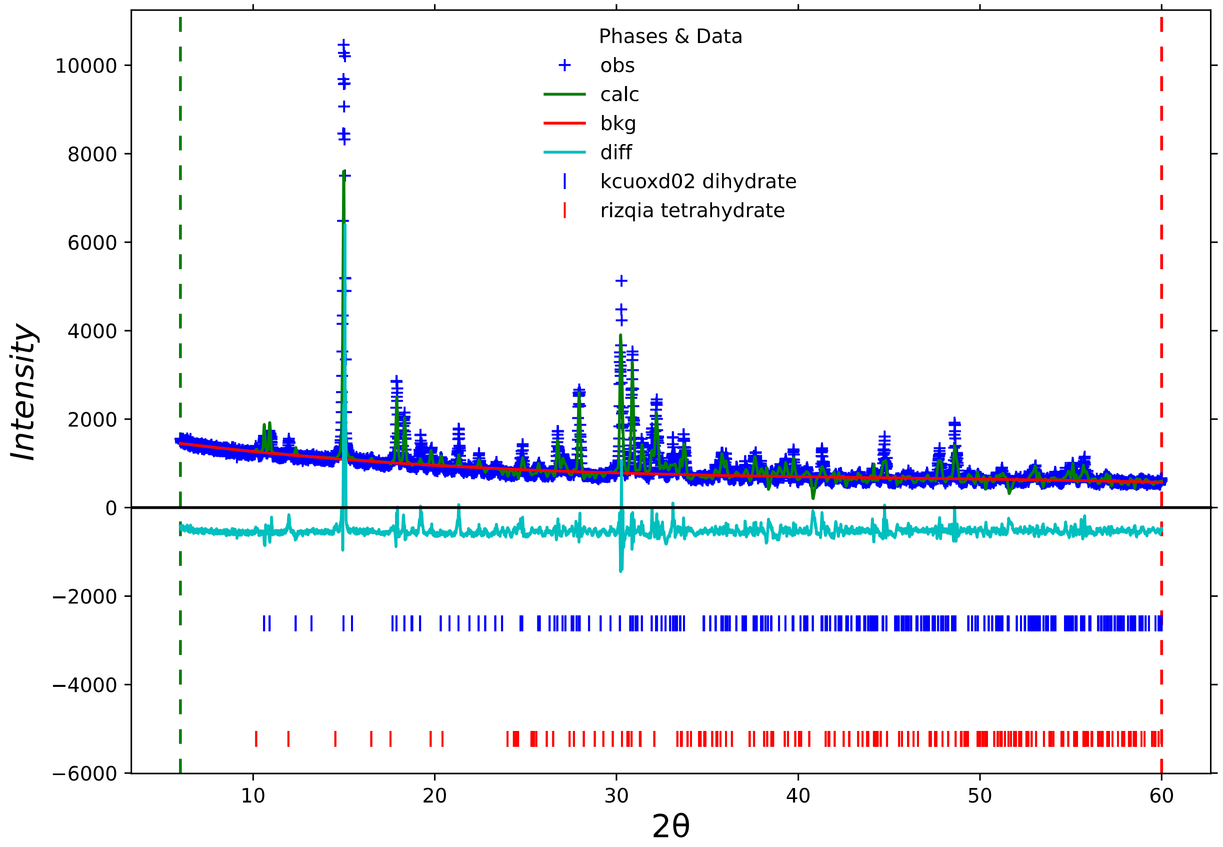

Figure S3: PXRD data and Rietveld refinement for $K_2[Cu(ox)_2]$.

Dark blue = experimental data; green = simulation; teal = residuals; red = baseline. Blue dashes correspond to predicted peak positions for reference structure RIZQIA and red dashes correspond to predicted peak positions for reference structure KCUOXD02.

3. X-Ray Crystallography Data for $(PPN)_2[Cu(ox)_2]$

Table S3: Crystal data and structure refinement for $(PPN)_2[Cu(ox)_2]$.

CCDC Deposition Number	2383406	
Empirical formula	C76 H60.93 Cu N2 O8.47 P4	
Formula weight	1325.05	
Temperature	100(2) K	
Wavelength	0.71073 Å	
Crystal system	Monoclinic	
Space group	$P2_1/n$	
Unit cell dimensions	a = 9.2811(19) Å	a= 90°.
	b = 15.656(3) Å	b= 101.791(8)°.
	c = 21.830(5) Å	g = 90°.
Volume	3105.0(11) $Å^3$	
Z	2	
Density (calculated)	1.417 Mg/m^3	
Absorption coefficient	0.518 mm^{-1}	
F(000)	1375	
Crystal size	0.300 x 0.300 x 0.300 mm^3	
Theta range for data collection	1.906 to 36.339°.	
Index ranges	-15<=h<=15, -26<=k<=26, -36<=l<=36	
Reflections collected	147526	
Independent reflections	15076 [R(int) = 0.0485]	
Completeness to theta = 25.242°	99.9 %	
Absorption correction	Semi-empirical from equivalents	
Max. and min. transmission	0.4977 and 0.4535	
Refinement method	Full-matrix least-squares on F^2	
Data / restraints / parameters	15076 / 2 / 428	
Goodness-of-fit on F^2	1.026	
Final R indices [I>2sigma(I)]	R1 = 0.0334, wR2 = 0.0899	
R indices (all data)	R1 = 0.0440, wR2 = 0.0954	
Extinction coefficient	n/a	
Largest diff. peak and hole	0.717 and -0.840 e.$Å^{-3}$	

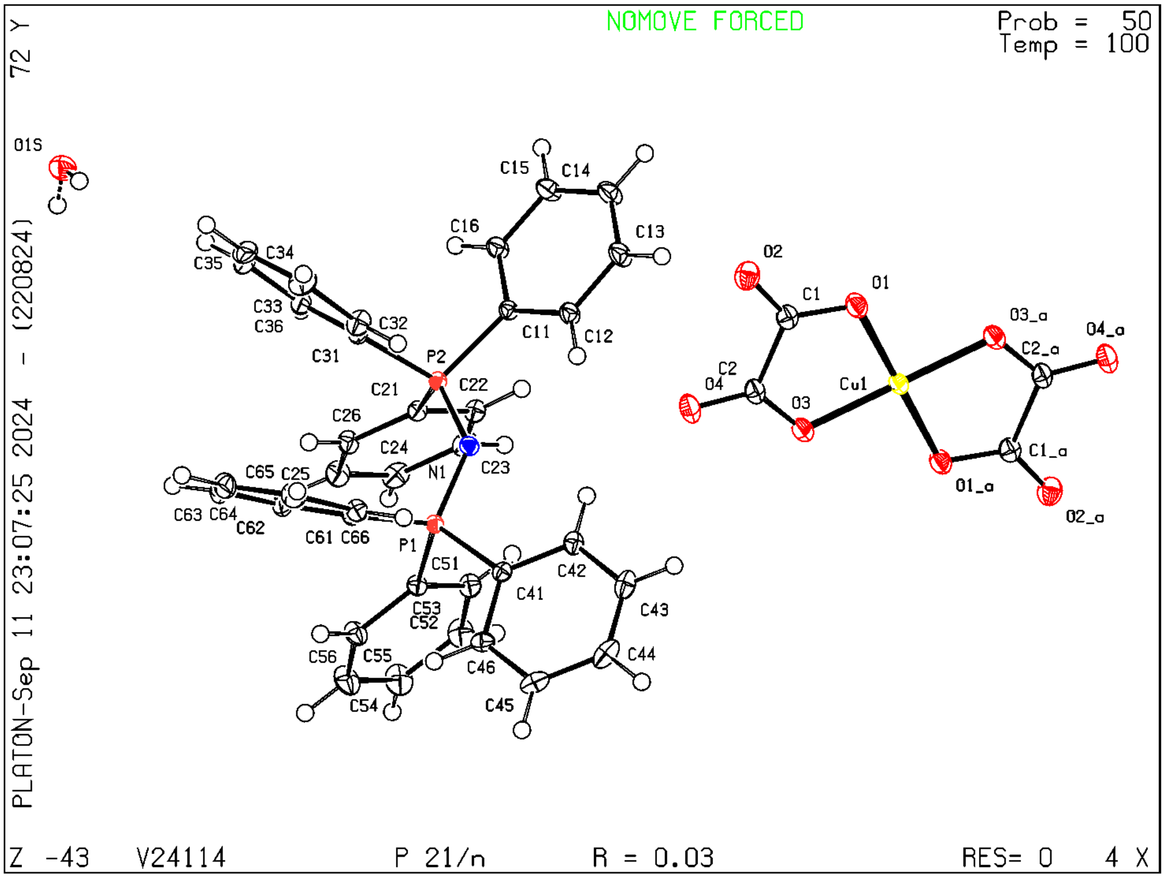

Figure S4: Asymmetric unit in the single crystal structure of (PPN)$_2$[Cu(ox)$_2$]. Thermal ellipsoids are drawn with 50% probability.

4. UV-vis Spectroscopy

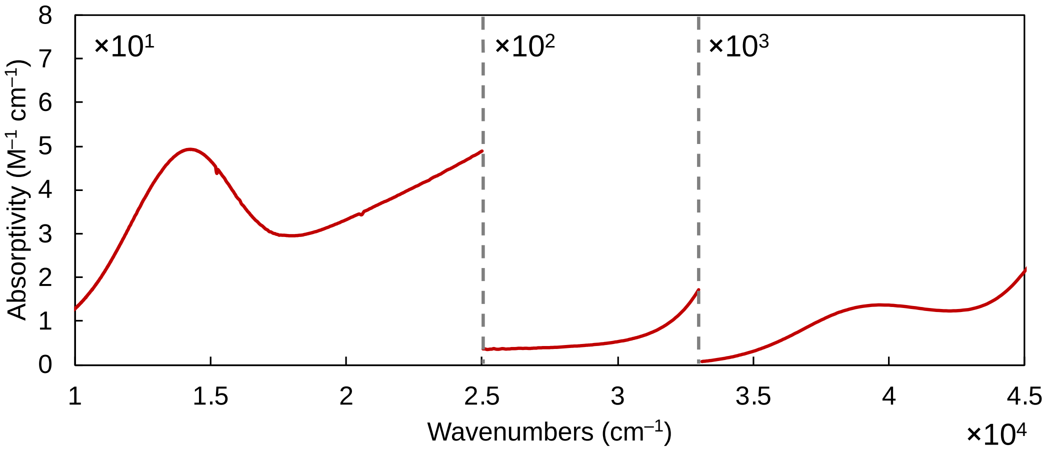

Figure S5: UV-vis spectrum of $K_2[Cu(ox)_2]$ in water.

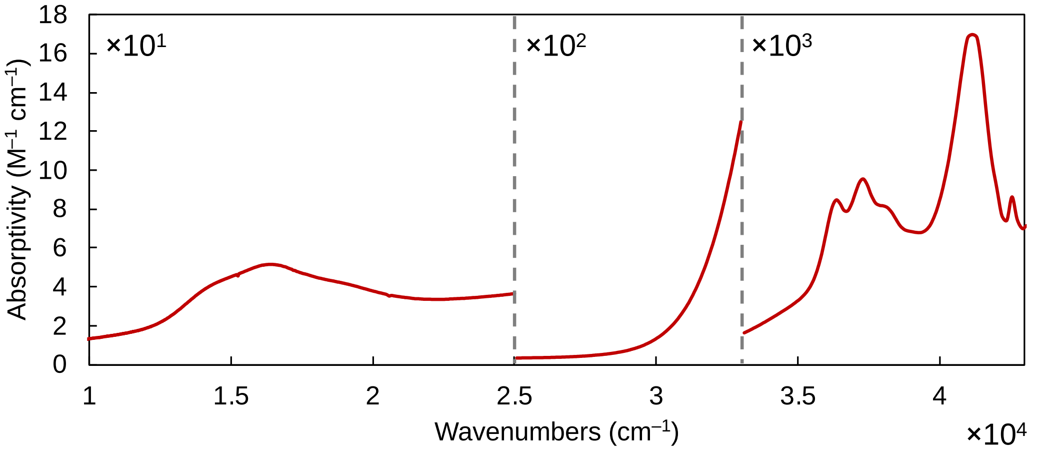

Figure S6: UV-vis spectrum of $(PPN)_2[Cu(ox)_2]$ in chloroform.

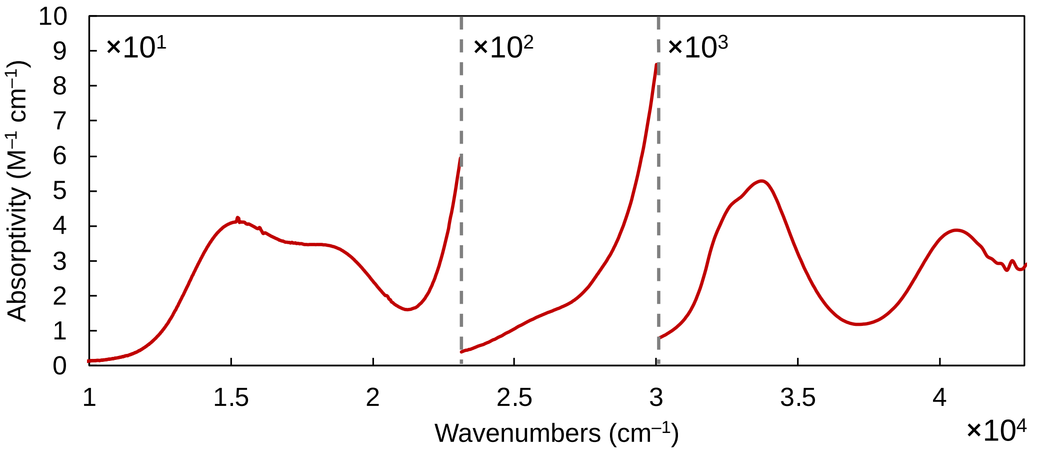

Figure S7: UV-vis spectrum of $Cu(acac)_2$ in chloroform.

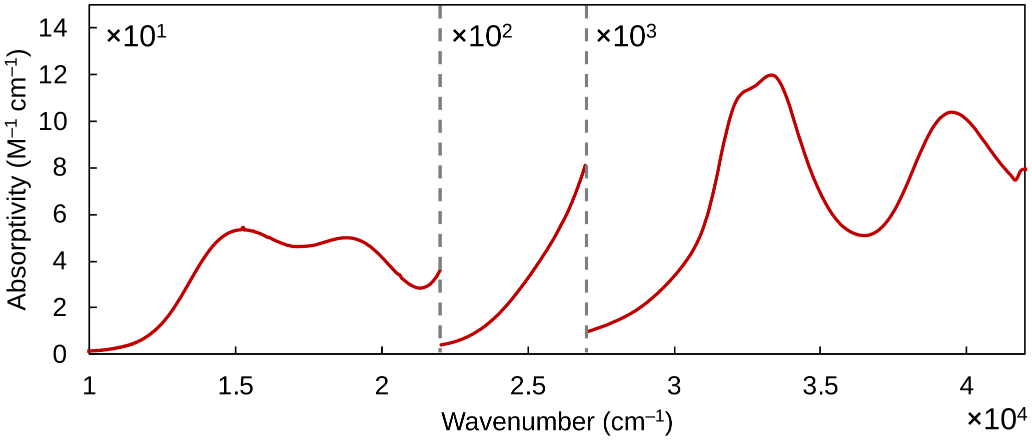

Figure S8: UV-vis spectrum of $Cu(tmhd)_2$ in chloroform.

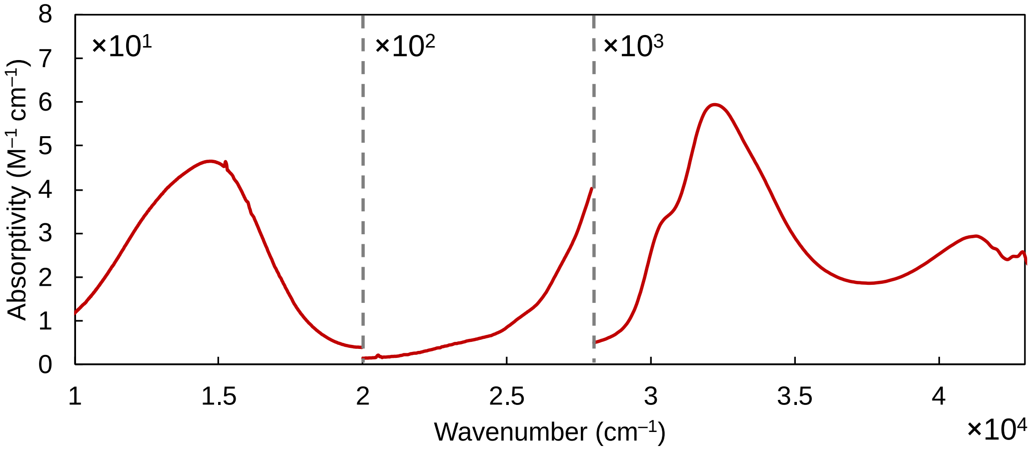

Figure S9: UV-vis spectrum of $Cu(hfac)_2$ in chloroform.

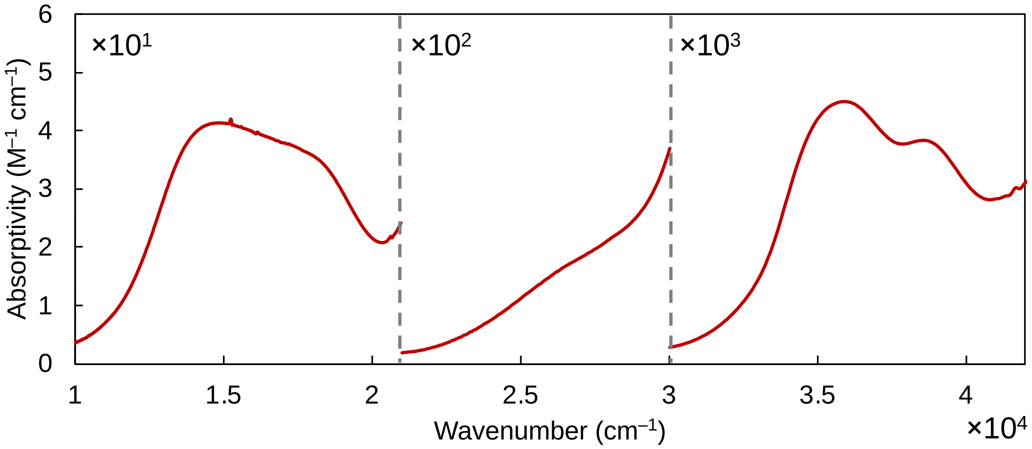

Figure S10: UV-vis spectrum of $Cu(tbaa)_2$ in chloroform.

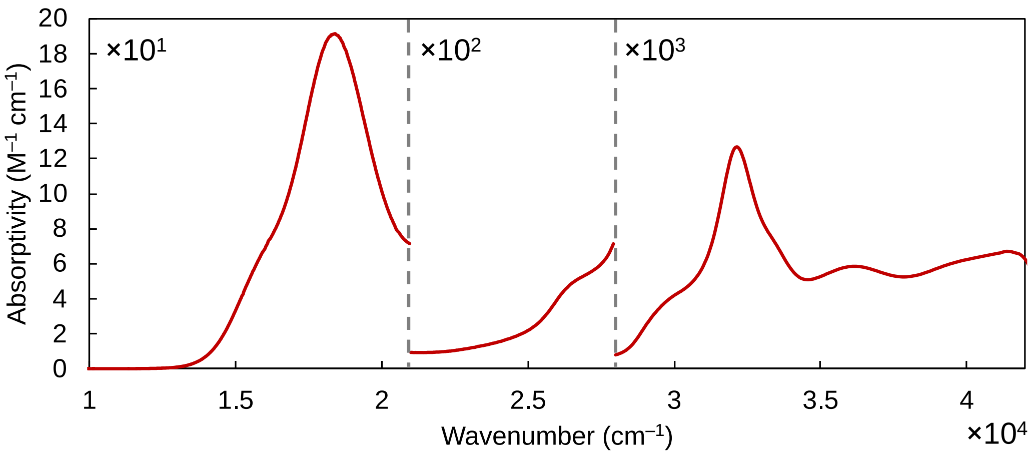

Figure S11: UV-vis spectrum of Cu(acacen) in chloroform.

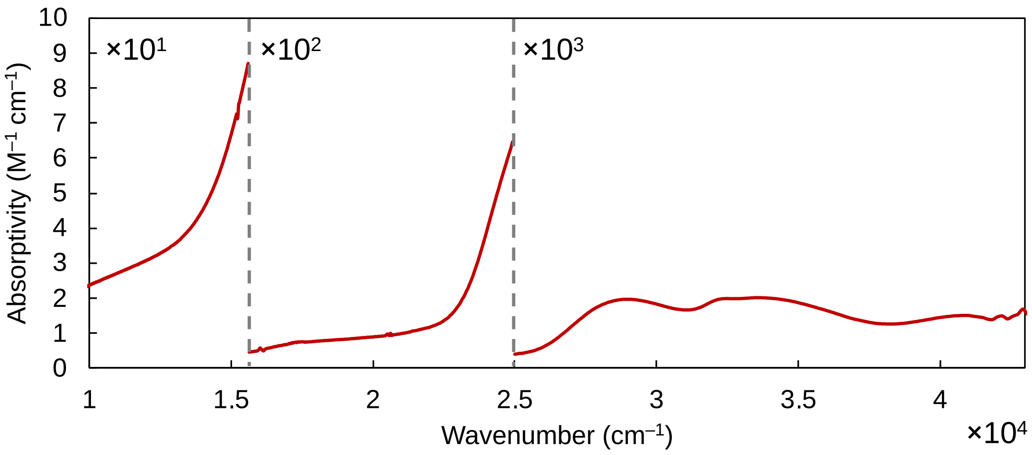

Figure S12: UV-vis spectrum of Cu(pci)$_2$ in chloroform.

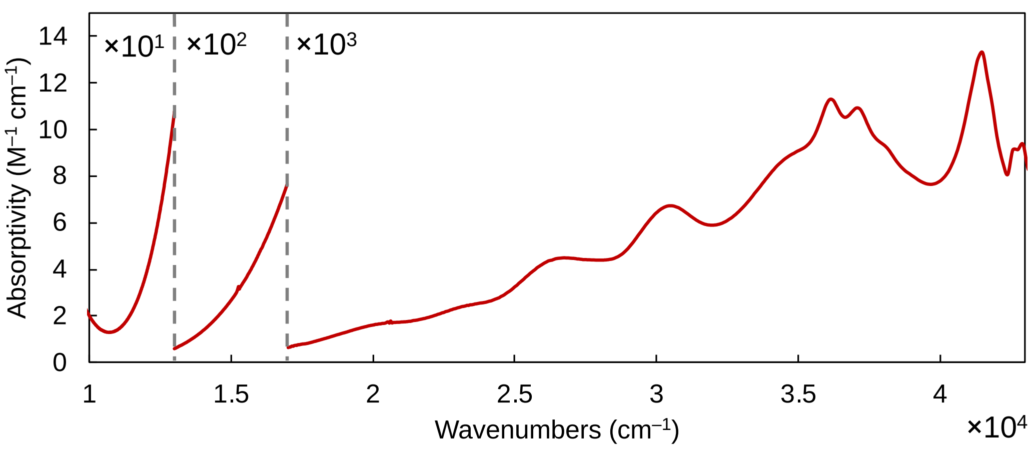

Figure S13: UV-vis spectrum of $(PPh_4)_2[Cu(mnt)_2]$ in chloroform.

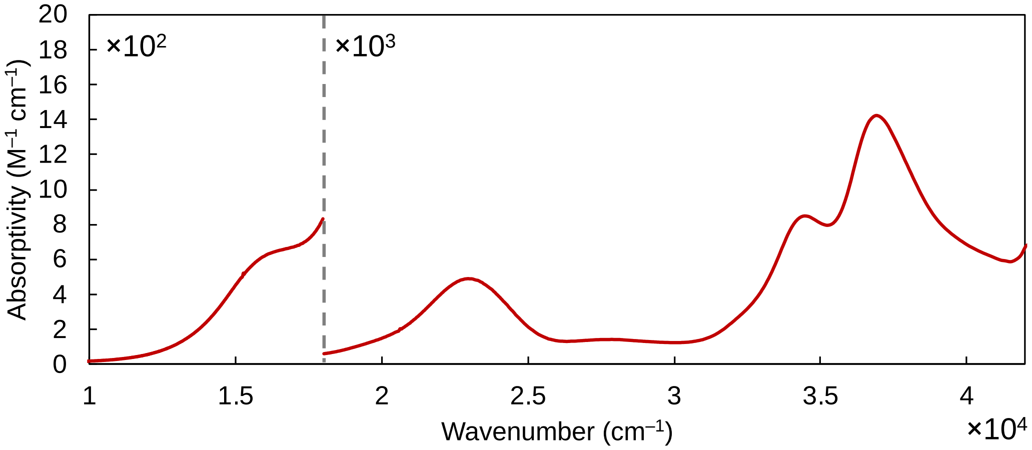

Figure S14: UV-vis spectrum of $Cu(dtc)_2$ in chloroform.

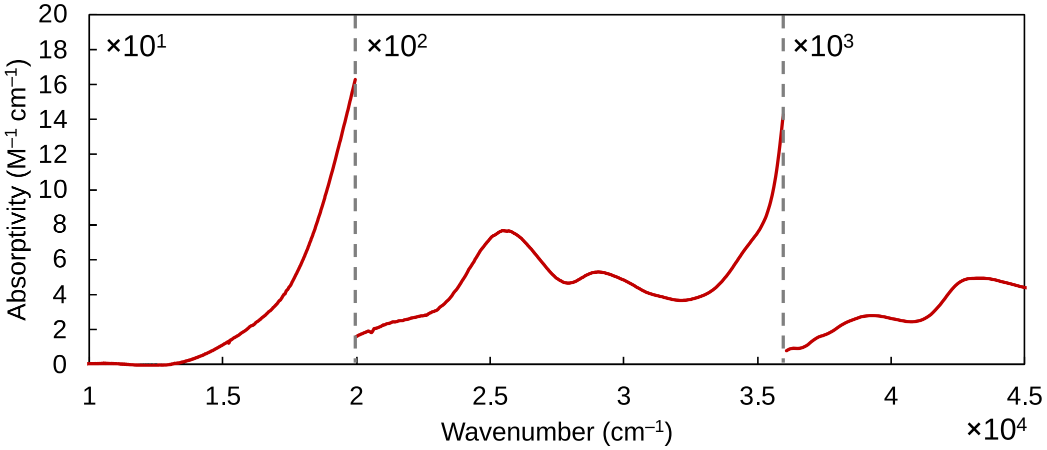

Figure S15: UV-vis spectrum of $(PPh_4)_2[Cu(bdt)_2]$ in chloroform.

5. MCD Spectroscopy

5.1 MCD Methods

Magnetic circular dichroism (MCD) spectra were collected using a homebuilt setup employing commercial spectrometer components (**Figure S16**). A commercial J1700 circular dichroism spectrometer (Jasco Inc., Tokyo, Japan) generates the circularly polarized light from either a xenon lamp (250-800 nm) or a tungsten lamp (800-2500 nm), which is made monochromatic through a prism (250-1600 nm) or a grating (1600-2500 nm) and subsequently passes through a photoelastic modulator (PEM) in the spectrometer source compartment. The monochromators are separately calibrated *via* a third mercury lamp in the source compartment, which is not used during measurements. The sample chamber is left empty, and the detector compartment is detached, such that the incoherent beam exits the spectrometer into free space. The beam was found to possess a small vertical divergence, so a cylindrical lens was employed for collimation along the vertical axis. All lenses used are made from uncoated quartz (Thorlabs, Inc., Newton, NJ, USA), permitting broadband wavelength transmission. The beam is focused into the windows of a cryogen-free C-Mag Vari-7T magneto-optical cryostat (Cryomagnetics, Inc., Oak Ridge, TN, USA), with a maximum magnetic field of 7 T and a minimum sample temperature of 1.6 K. The sample is mounted between two copper plates screwed on the end of a sample rod (see below) and sits in a helium exchange gas. Vertical sample alignment is accomplished by raising and lowering the sample rod. Horizontal sample alignment is accomplished *via* a screw jack apparatus bolted to the optical table. The magneto-optical cryostat sits on a Teflon pad, enabling the screw jack to push and pull the entire system from side to side. Once the beam exits the sample compartment, it is collimated, and subsequently focused into the J1700 detector compartment. The automatic detector changer employs a photomultiplier tube (PMT) from 250-800 nm and two InGaAs photodiode detectors from 800-1600 nm and 1600-2500 nm. To mitigate magnetic field interference on the PMT, a sheet of MuMetal magnetic shielding (Magnetic Shield Corp., Bensenville, IL, USA) was placed in front of the detector compartment, with a small hole punched through to allow passage of the optical beam.

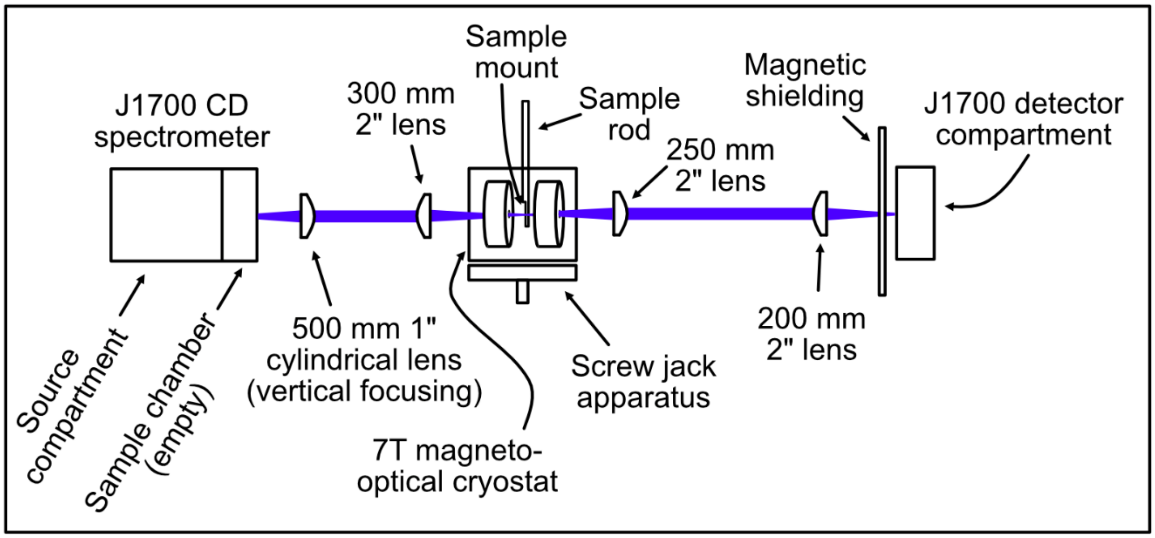

Figure S16: Block diagram of the homebuilt MCD spectrometer.

The copper sample holder consists of two plates with circular insets that can be tightened through countersunk screws. This permits a variety of options for sample mounting. Polymer films (**SI**

Section 1.3) deposited on a circular quartz disk were covered with a Viton chemical-resistant fluoroelastomer O-ring and a second quartz disk as a coverslip; the assemblage of two disks and an O-ring is tightened by the countersunk screws. Mull samples were mounted between two quartz disks without an intermediate O-ring; the pressure from the disks smooths the Fluorolube mull, improving the optical quality. Frozen solution samples were prepared by pre-assembling two quartz disks with a Viton gasket spacer. Two needles were inserted into the gasket. Through one needle, a syringe injects a liquid solution sample; the other needle serves as a vent port. $(PPh_4)_2[Cu(bdt)_2]$ was found to be air sensitive, so the solution cells for were assembled in an inert nitrogen atmosphere glovebox, removed through an antechamber, and immediately immersed in liquid nitrogen for rapid flash freezing. When prepared with a suitable optically-glassing solvent, the resulting frozen solution cells are suitable for optical transmission. Adjustment of the sample position enables mitigation of scattering from cracks that form during the freezing process. Apart from $(PPh_4)_2[Cu(bdt)_2]$, other samples were mounted in the copper holder under air. This typically entails a 1-2 minute exposure to air. Nearly all of the compounds reported here are stable under air for at least short periods of time (see **SI Section 1.2**).

The spectrometer enables *in situ* acquisition of UV-vis-NIR absorption spectra under the same cryogenic conditions as the MCD spectra. These spectra were acquired at the conclusion of an MCD measurement, but before altering the sample position, ensuring direct comparability to the MCD spectra. To acquire an absorption spectrum, the magnetic field was discharged to 0 T. The high voltage gain on the PMT was set to a fixed value, being careful not to overload the detector, and a scan was taken. The J1700 CD spectrometer records both the CD signal (produced from lock-in amplification at the PEM frequency) and also the direct-current (DC) voltage from the detector. The latter channel quantifies the total light intensity with the sample present, I. The sample rod was then removed from the cryostat, and another scan was taken without changing the fixed detector gains. The DC voltage from this measurement serves as the reference light intensity, I_0. When necessary, a third scan was collected with the source blocked, given the dark intensity I_d. The absorbance was then calculated by the textbook relationship (**Equation S1**). This protocol effectively operates the J1700 as a single-beam absorption spectrometer.

$$A = -\log_{10}\left(\frac{I - I_d}{I_0 - I_d}\right) \tag{S1}$$

The absorption spectra acquired in this manner were compared with UV-vis-NIR absorption spectra acquired at room temperature on a Cary 500 dual-beam absorbance spectrometer. Good agreement was generally found, though the single-beam J1700 absorption spectra were significantly noisier. Additionally, the J1700 absorption spectra display nonzero baseline offsets, frequently with a quasi-linear slope in the spectral region of interest. This arises due to scattering off of the materials in the sample holder, as the quartz disks tend to acquire a thin film of frozen atmosphere, which increases the apparent optical density of the sample. Owing to detector drift during the long cooldown times of the sample rod (1-2 hours), as well as the effect of slightly different sample rod positioning, it was not possible to employ a second assembled cell with a blank polymer film as an effective reference blank. Thus, the baseline offset was manually subtracted prior to conducting C_0/D_0 ratio fits, so that the absorbance comes only from the MCD-active compound.

The MCD spectral shapes were validated by acquiring a spectrum of $K_3[Fe(CN)_6]$ prepared in a polyvinyl alcohol film, according to a literature standard.[11] Very good agreement was found over the optical transmission range of the instrument. The MCD spectrum at a given field strength B is calculated by acquiring the ellipticity θ at positive (parallel) field (+B) and negative (antiparallel) field (-B). Then, the MCD is calculated by:

$$MCD = \frac{\theta(+B) - \theta(-B)}{2} \tag{S2}$$

A baseline scan was always acquired by discharging the field to 0 T and acquiring the ellipticity spectrum. Application of the 0 T baseline correction does affect the calculated MCD signal, as the correction cancels in the subtraction. It does, however, enable validation of the spectrum quality. It should be the case that the $\theta(+B)$ and $\theta(-B)$ are mirror images about the 0 T baseline; if not, artifacts may be present, e.g. from field interference on the detectors. We ensured the reliability of these spectra through the 0 T baselines.

For samples with good optical quality (scattering $\leq$ 0.1 Abs, which was satisfied for most of the polymer films), the above procedure could be followed to obtain a high-quality MCD spectrum with no further modifications. For samples with more optical scattering (particularly, several of the Fluorolube mull samples), it was observed that broad field-dependent ellipticity signals could arise, producing an effective baseline shift in the ellipticity as a function of field. A significant component of this shift displayed odd parity in the field, so the subtraction in **S2** does not remove it. However, it was also observed that the scattering signal was temperature invariant over the 5 – 20 K range, while the molecular C-term intensity desired varies as 1/kT. Therefore, to remove scattering artifacts, we typically subtracted two MCD spectra at different temperatures (for example, 5 K and 10 K) to obtain the pure molecular C-term spectrum. This subtraction procedure also removes any temperature-independent B-term contributions to the molecular MCD spectra, which was desirable for accurately calculating C_0/D_0 ratios. Therefore, we generally applied this two-point temperature correction even for samples with good optical quality, with a couple exceptions noted in the description of each sample.

The sample temperature was chosen to be as low as feasible within the base temperature of the cryostat at the time of the measurements. Thus, some samples were able to be run in recirculating evaporative cooling (1.6 – 5 K), while others were run with gas recirculation (≥5 K). This does not affect the interpretation of the spectra, as temperature variations are accounted for in the calculation of the C_0/D_0 ratios (*vide infra*). Temperature control at the sample was actively maintained through a sample heater connected to a PID loop. Fidelity to the setpoints indicated was always better than 0.1 K, but generally better than 0.01 K. When changing the setpoint, a waiting time was given for sample thermal re-equilibration before measuring a new spectrum.

Depolarization tests were conducted by comparing the CD signal produced by a solution of nickel tartrate placed (1) in the J1700 sample compartment, before the cryostat or the sample and (2) immediately before the magnetic shielding, after the cryostat and sample. This enables determination of the loss of circular polarization due to the optics and sample in the cryostat by taking the ratio of the two measurements. Nickel tartrate was prepared by producing a solution of 0.12 M $Ni(SO_4) \cdot 6H_2O$ and 0.18 M Rochelle salt (sodium potassium L(+)-tartrate tetrahydrate) in

deionized water in a volumetric flask. The solution was measured in a 1-cm pathlength quartz cuvette before and after the sample. Small baseline shifts arose, particularly in the UV, from mounting the cuvette outside a sample holder in front of the magnetic shielding. Depolarization tests were conducted with three samples: (a) a PMMA film sample (**Figure S17**), (b) a low-scattering Fluorolube mull ($K_2[Cu(ox)_2]$ hydrate, **Figure S18**) and (c) a high-scattering Fluorolube mull ($Cu(acac)_2$, **Figure S19**).

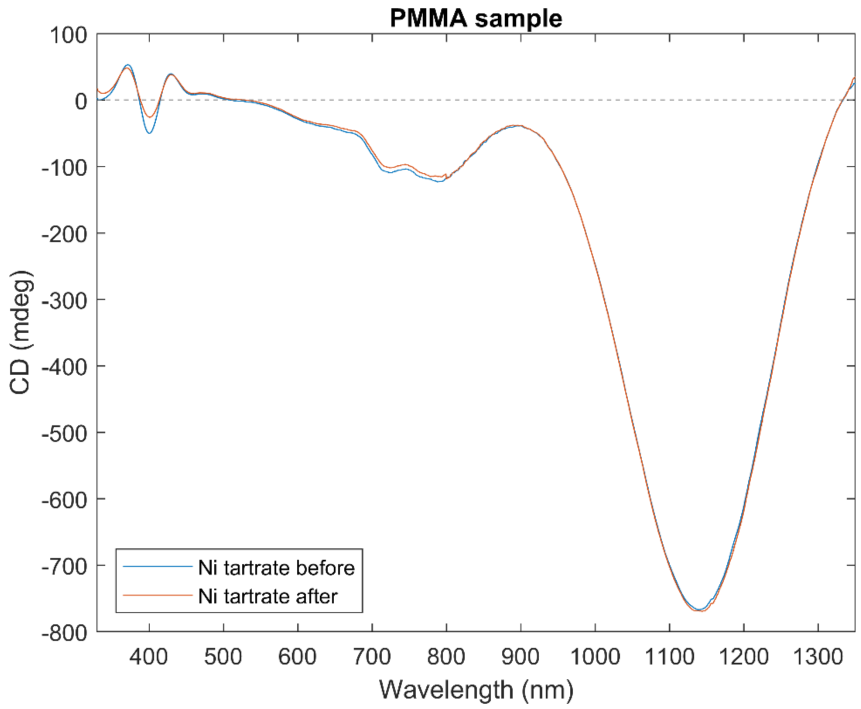

Figure S17: Nickel tartrate depolarization tests for a PMMA sample.

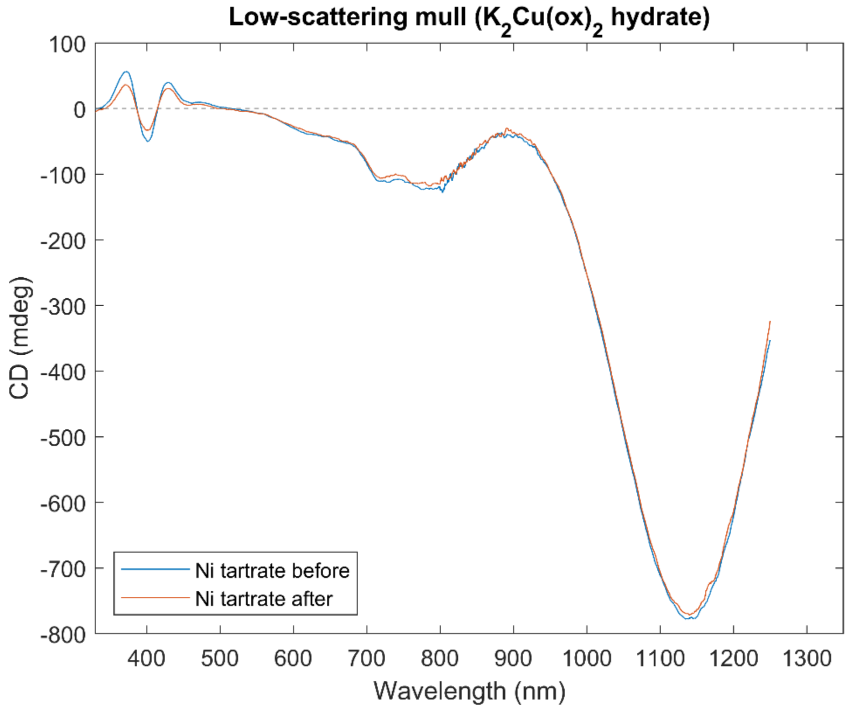

Figure S18: Nickel tartrate depolarization tests for a Fluorolube mull ($K_2[Cu(ox)_2]$ hydrate) with small optical scattering.

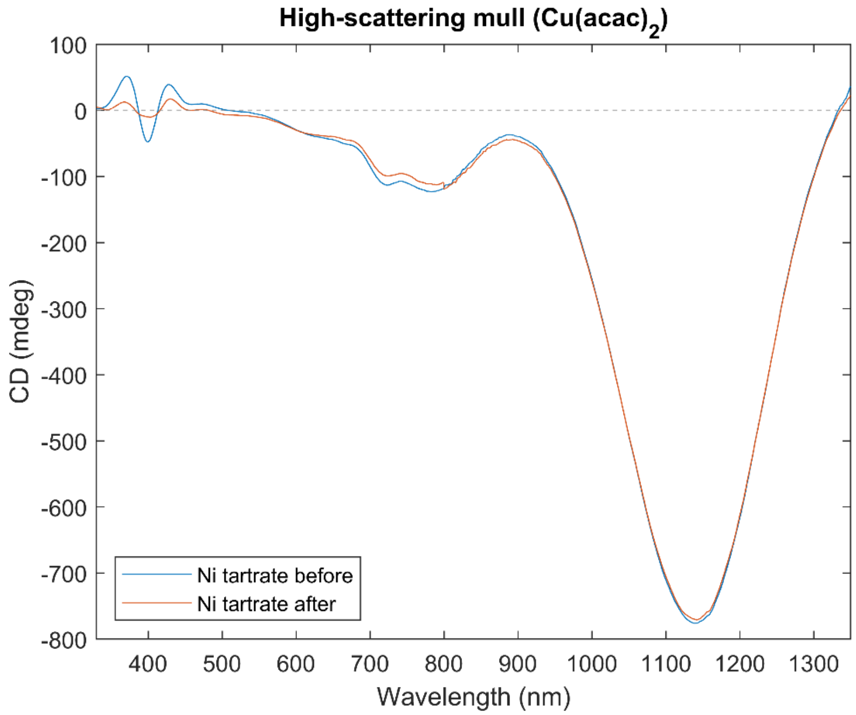

Figure S19: Nickel tartrate depolarization tests for a Fluorolube mull ($Cu(acac)_2$) with large optical scattering.

Table S4: Wavelength-dependent depolarization values obtained from nickel tartrate measurements.

*Values are calculated as (after)/(before)*100%, thereby quantifying the percentage of polarization that is retained through the sample at the given wavelength. "NIR" values obtained from 1140 nm, "Red vis" values averaged over 790 nm and 725 nm, and "Blue vis / UV" values averaged over 429, 400, and 371 nm.*

Wavelength (nm)	PMMA sample (%)	Low-scattering mull (%)	High-scattering mull (%)
1140	100	99	99
790	94	94	92
725	93	95	88
429	96	77	44
400	51	67	21
371	90	65	23
NIR	100	99	99
Red vis	94	94	90
Blue vis / UV	79	70	29

For the polymer film samples, no more than ~20% circular polarization is lost across the spectrum. The depolarization is maximized in the UV and blue region of the visible spectrum due to the wavelength dependence of scattering; negligible depolarization arises in the NIR. The high-quality mull displays a similar good fidelity of polarization, with 30% loss in the blue/UV. By contrast, the low-quality mull displayed intense scattering, causing a loss of 70% of the polarization in the blue/UV, though depolarization was still small at longer wavelengths. Samples in this work which report C_0/D_0 ratios and acquisition of cryogenic UV-vis spectra have optical qualities most similar to the PMMA sample, so we do not expect depolarization effects to significantly perturb the C/D ratios. Mull samples with poor optical quality may have some depolarization effects in the blue/UV, rendering these spectra most reliable in the visible and NIR. As the ligand field bands analyzed fall in the region without depolarization, this potential for some UV depolarization is not a source of concern.

To extract C_0/D_0 ratios, the MCD and corresponding cryogenic UV-vis absorption spectra (*vide supra*) were jointly fit, where possible, to Gaussian bands using a Matlab package developed in-house. (Note that for some samples, low molar absorptivity and/or optical scattering prevented acquisition of a reliable UV-vis absorption spectrum, so the MCD spectrum was fit by itself.) For any given transition, a Gaussian peak was placed at the same center wavelength in both the MCD and the UV-vis absorption spectra. The full-width-at-half-max (FWHM) for the transition was additionally constrained to be identical in both the MCD and the absorption spectra. These constraints can slightly worsen the quality of the fit, but greatly improve chemical interpretability and allow for direct C/D calculation from the relative heights of the peaks. The peak height was allowed to vary independently in the MCD and absorption spectra; in the former case, negative heights are allowed. All parameters were fit by manually adjusting the peaks to provide a plausible representation of the data, as least-squares fits were frequently found to be unphysical (for example, very large positive and negative intensities that cancel out). When fitting a bisignate pseudo-A term to Gaussians, it is frequently possible to choose many intensity values for the positive and negative bands, allowing the overlap between the bands to cause cancellations and adjust the steepness of the transition through zero. We took the approach of using the smallest absolute

intensities that could plausibly model the observed spectra. Note that symmetry-enforced orbital degeneracies are not present, owing to the twofold rotational symmetry (at most) of the ligand frameworks employed. It is therefore appropriate to fit all bisignate terms to Gaussians under the assumption that they are pseudo-A terms. Throughout, these fitting choices are motivated by chemical interpretability.

The J1700 readout is given in units of ellipticity (mdeg). The conversion from millidegrees to differential LCP and RCP absorption is given by the standard equation:

$$\Delta Abs = \frac{\theta(mdeg)}{32980} \tag{S3}$$

When the MCD is in units of ΔAbs and the UV-vis absorption is in units of Abs, the C_0/D_0 ratio may be calculated. Denoting the band integral of the UV-vis absorption peak as Q(Abs) and the band integral of the MCD peak as Q(MCD), we have in the linear (non-saturated) limit:

$$C_0/D_0 = \frac{Q(MCD)}{Q(Abs)} \times \frac{k_B T}{\beta B} \tag{S4}$$

Here k_B is the Boltzmann constant and β is the Bohr magneton. If a pure C-term MCD spectrum is being employed through the temperature subtraction process, then the equation becomes:

$$C_0/D_0 = \frac{Q_2(MCD) - Q_1(MCD)}{Q(Abs)} \times \frac{1}{\left(\frac{\beta B_1}{k_B T_1} - \frac{\beta B_2}{k_B T_2}\right)} \tag{S5}$$

If the magnetization is partially in the saturating regime, then the C_0/D_0 ratio may still be calculated for a S = ½ system provided that the g value is approximately known. The resulting, most general equation becomes:

$$C_0/D_0 = \frac{Q_2(MCD) - Q_1(MCD)}{Q(Abs)} \times \frac{g}{2\left(tanh\left(\frac{g\beta B_1}{2k_B T_1}\right) - tanh\left(\frac{g\beta B_2}{2k_B T_2}\right)\right)} \tag{S6}$$

Here the hyperbolic tangent arises from the S = ½ Brillouin function. We use this latter equation to calculate the C_0/D_0 values in the general case, assuming g ≈ 2 for a S = ½ system. Deviations from the linear limit calculation are no greater than 17% ($Cu(pci)_2$), and generally much less than that.

A summary of all MCD spectra collected is presented in the following sections, organized by the sample preparation matrix (polymer film, frozen solution, or Fluorolube mull).

5.1.1 Parameters for MCD spectra

The temperature and magnetic field strengths corresponding to each MCD spectrum in Figure 4 of the main text are given below. Temperatures and field strengths were optimized for each compound to efficiently acquire high-quality C-term spectra. Spectra at different fields and temperatures are directly comparable through the C-term intensity values. All spectra are calculated from positive and negative field values (parallel and antiparallel directions) as described in **Equation S2**. All field strengths are nominally accurate to 1 mT. All temperatures are nominally accurate to 0.01 K.

Table S5: Temperature and field strengths for MCD spectra corresponding to Figure 4 of the main text.

Compound	Matrix	Temperature	Field
$(PPh_4)_2[Cu(mnt)_2]$	PMMA	2.0 K	0.5 T
$Cu(dtc)_2$	PS	5.5 K – 10.0 K	2 T
$Cu(pci)_2$	PS	5.0 K – 10.0 K	4 T
Cu(acacen)	PS	5.0 K – 10.0 K	2 T
$Cu(tbaa)_2$	PS	5.0 K – 10.0 K	2 T
$Cu(tmhd)_2$	PMMA	5.0 K – 20.0 K	2 T
$(PPN_2)[Cu(ox)_2]$	PS	5.0 K – 10.0 K	4 T
$K_2[Cu(ox)_2]$	PVA	5.0 K – 10.0 K	4 T
$Cu(hfac)_2$	PS	5.0 K – 10.0 K	4 T
$(PPh_4)_2[Cu(bdt)_2]$	1:1 butyronitrile:DCM	6.5 K – 20.0 K	6 T
$Cu(acac)_2$	1:1 DCM:toluene	5.0 K	1 T
$(PPh_4)_2[Cu(mnt)_2]$	mull	5.0 K	7 T
$Cu(dtc)_2$	mull	5.0 K – 20.0 K	2 T
$Cu(tmhd)_2$	mull	5.0 K – 10.0 K	4 T
$Cu(acac)_2$	mull	2.0 K	0.5 T
$K_2[Cu(ox)_2]$	mull	5.0 K – 20.0 K	7 T
$Cu(hfac)_2$ hydrate	mull	5.0 K – 10.0 K	4 T

5.2 MCD Analysis

Many of the MCD spectra display a pronounced negative band on the high-energy side of the ligand field region that constitutes the most prominent common feature of the spectrum; it is therefore of particular interest to assign this transition. TDDFT calculations predict that the $x^2–y^2 \rightarrow xy$ transition should generally fall at the highest energy of the four ligand field transitions (**Table S29**). The exceptions to this occur for the acetylacetonate ligands, where the $x^2–y^2 \rightarrow xy$ and $yz \rightarrow xy$ transitions are approximately degenerate. We therefore assign the highest-energy ligand field band (which is always negative and prominent) to the $x^2–y^2 \rightarrow xy$ transition, and tabulate these along with the remainder of the ligand field bands.

Tabulation of all fitted ligand field energies is given in **Table S6**, and C_0/D_0 ratios in **Table S7**. The spin relaxation rates used for the correlation are also included, copied from **SI Section 6.2**. As all T_1 measurements were conducted in disordered matrices (polymer films or frozen solutions), these values are correlated with MCD spectra on polymer films or frozen solutions for direct comparability. Note that a C_0/D_0 ratio around 0.1 is characteristic of a ligand field band, while a ratio around 0.01 is more characteristic of a charge transfer transition.[12]

Table S6: Tabulation of fitted ligand field transition energies from MCD spectra for all samples in this study, together with associated spin relaxation rates.

Assigned $x^2–y^2$ excited state presents the most negative d-d MCD intensity; all assigned d-d transitions are listed in ascending order. Lowest energy transition omitted from the average sum for $(PPh_4)_2[Cu(mnt)_2]$ owing to substantial charge transfer character (see Section 2.2 in the main text). All values in wavenumbers (cm^{-1}).

Compound	Matrix	*d–d* ($x^2–y^2$)	*d–d* (1)	*d–d* (2)	*d–d* (3)	*d–d* (4)	Average	$1/T_1$ (μs^{-1})
$(PPh_4)_2[Cu(mnt)_2]$	PMMA	21443	8249*	18622	20145	21443	20070	0.196
$Cu(dtc)_2$	PS	20517	16935	18933	20517		18795	0.305
$Cu(pci)_2$	PS	19970	16472	18056	19970		18166	0.912
Cu(acacen)	PS	18061	16013	16837	18601	19946	17849	0.724
$Cu(tbaa)_2$	PS	18637	13977	15171	16919	18637	16176	1.573
$Cu(tmhd)_2$	PMMA	19586	14021	15262	17561	19586	16607	1.769
$(PPN_2)[Cu(ox)_2]$	PS	18938	14079	15690	18938		16235	2.081
$K_2[Cu(ox)_2]$	PVA	15639	13166	15639	16953		15252	3.930
$Cu(hfac)_2$	PS	15401	12664	15401	17638		15234	3.240
$(PPh_4)_2[Cu(bdt)_2]$	1:1 butyronitrile:DCM	21228	19698	21228			20463	0.122
$Cu(acac)_2$	1:1 DCM:toluene	19088	14472	16260	17474	19088	16823	1.75
$(PPh_4)_2[Cu(mnt)_2]$	mull	20796	17655	19599	20796		19350	
$Cu(dtc)_2$	mull	16077	12646	14581	16077		14434	
$Cu(tmhd)_2$	mull	20388	20388	21484			20936	
$Cu(acac)_2$	mull	18589	14006	18589			16297	
$K_2[Cu(ox)_2]$	mull	15457	15457				15457	
$Cu(hfac)_2$ hydrate	mull	14419	11941	12639	14419	15683	13670	

Table S7: Tabulation of fitted ligand field C/D ratios from MCD spectra, together with associated spin relaxation rates.

Numbering of transitions corresponds to Table S6. C/D averages are conducted on the absolute value.

Compound	Matrix	C/D (x^2–y^2)	C/D (1)	C/D (2)	C/D (3)	C/D (4)	C/D (average)	1/T_1 (μs^{-1})
$(PPh_4)_2[Cu(mnt)_2]$	PMMA	-0.034	-0.013*	0.052	-0.025	-0.034	0.037	0.196
$Cu(dtc)_2$	PS	-0.045	0.045	-0.042	-0.045		0.044	0.305
$Cu(pci)_2$	PS	-0.084	0.146	0.044	-0.084		0.091	0.912
Cu(acacen)	PS	-0.062	0.091	0.120	-0.062	-0.052	0.091	0.724
$Cu(tbaa)_2$	PS	-0.190	0.180	-0.120	0.044	-0.190	0.115	1.573
$Cu(tmhd)_2$	PMMA	-0.164	0.190	-0.083	-0.055	-0.164	0.109	1.769

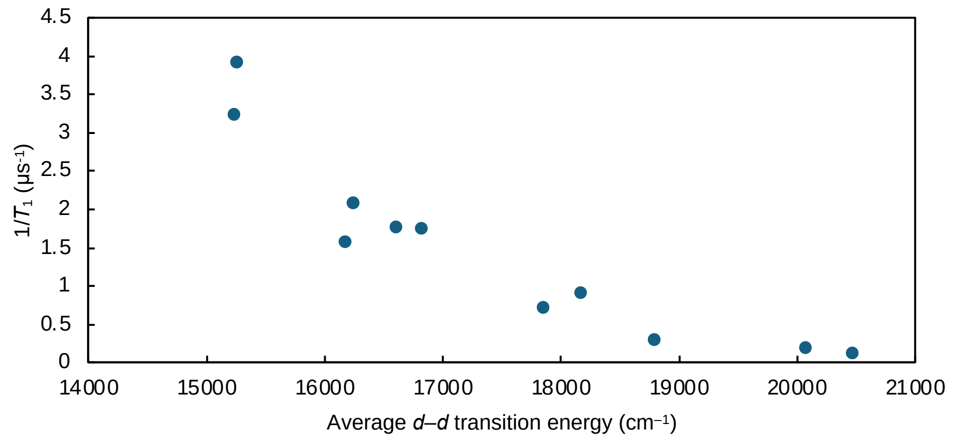

Figure S20: Plot of $1/T_1$ vs. average d–d transition energy.

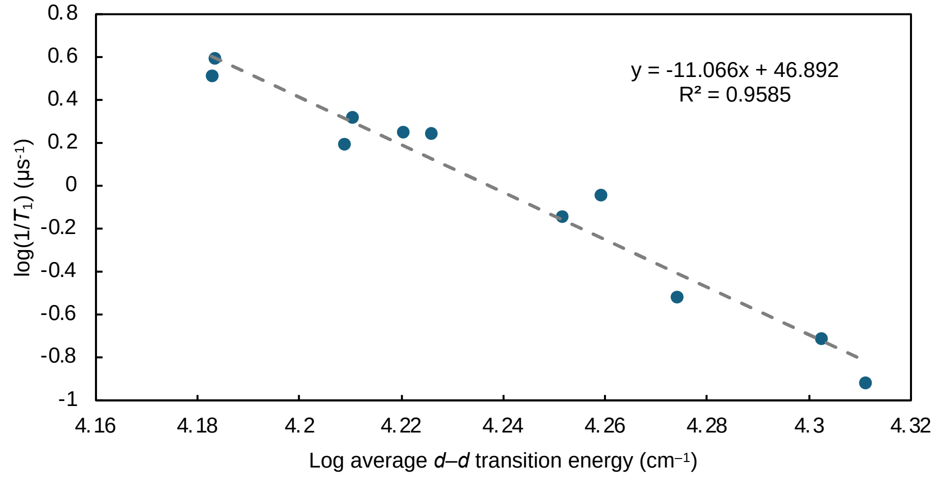

Figure S21: Log-log plot of $1/T_1$ vs. average d–d transition energy.

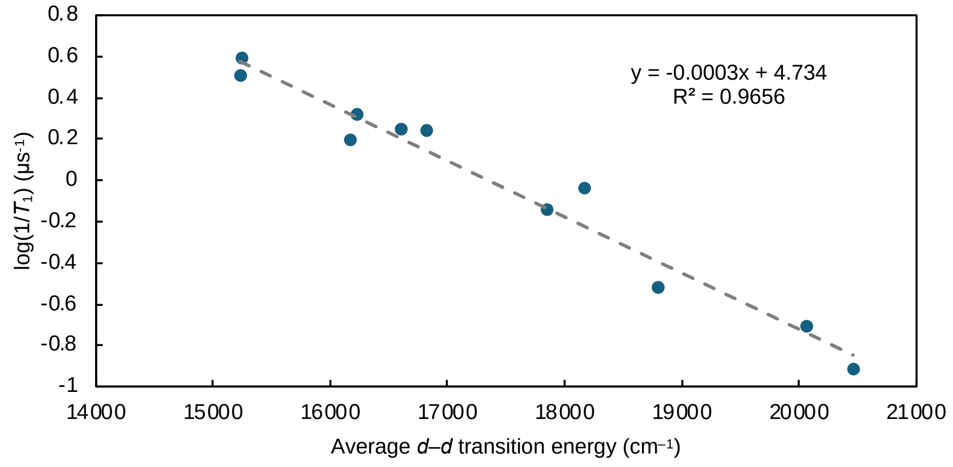

Figure S22: Plot of $\log(1/T_1)$ vs. average d–d transition energy.

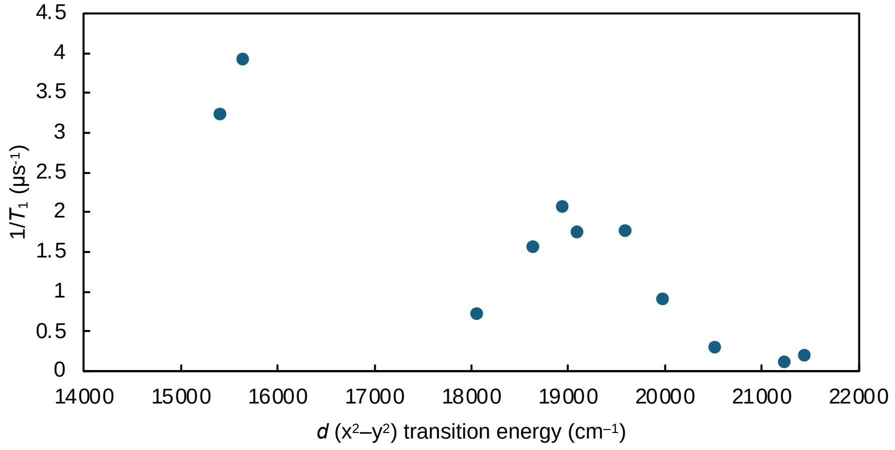

Figure S23: Plot of $1/T_1$ vs. $d(x^2–y^2)$ energy.

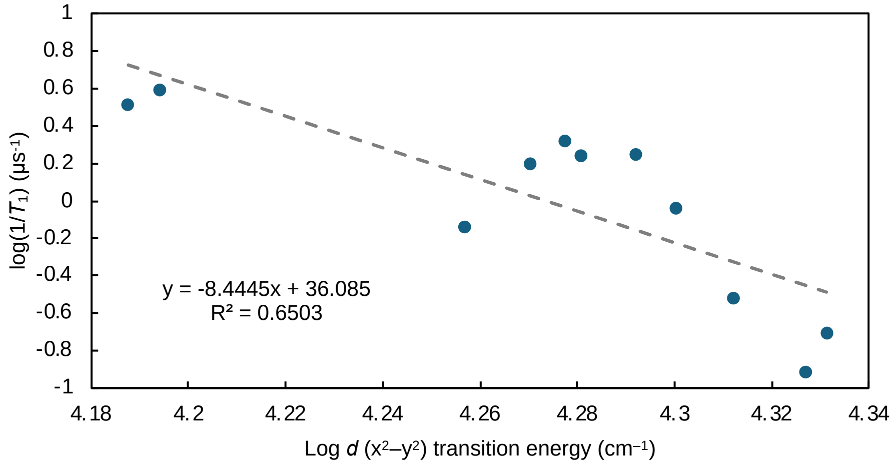

Figure S24: Log-log plot of $1/T_1$ vs. $d(x^2–y^2)$ energy.

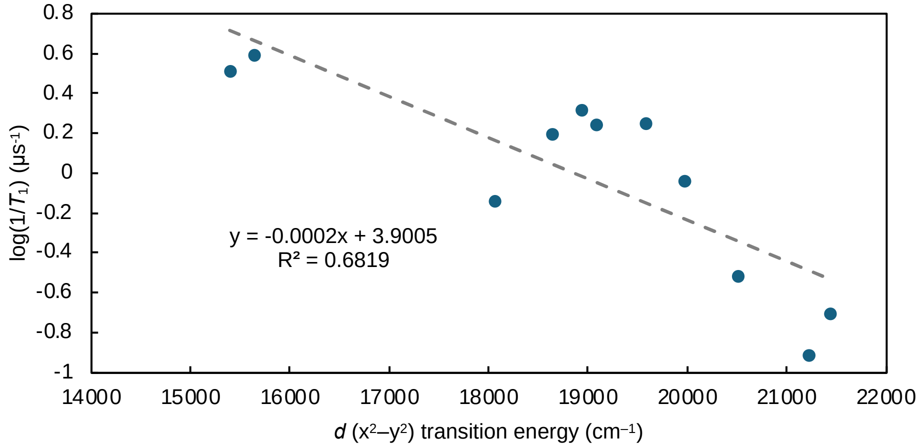

Figure S25: Plot of $\log(1/T_1)$ vs. $d(x^2–y^2)$ energy.

5.3 Polymer Film MCD

5.3.1 $(PPh_4)_2[Cu(mnt)_2]$ in PMMA film

The $(PPh_4)_2[Cu(mnt)_2]$ film was run at 2.0 K, where C-term intensity is expected to completely dominate the spectrum, and the sample displayed excellent optical quality. We therefore use the 2.0 K spectra without a temperature-dependent correction.

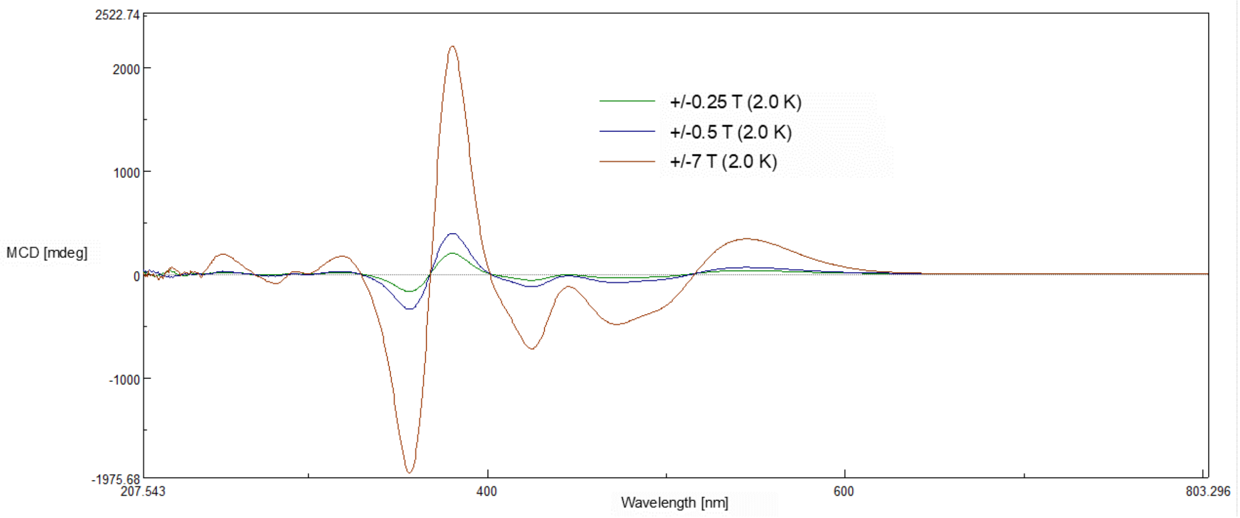

Figure S26: MCD spectra of $(PPh_4)_2[Cu(mnt)_2]$ in PMMA film at 2.0 K (UV-visible region of spectrum).

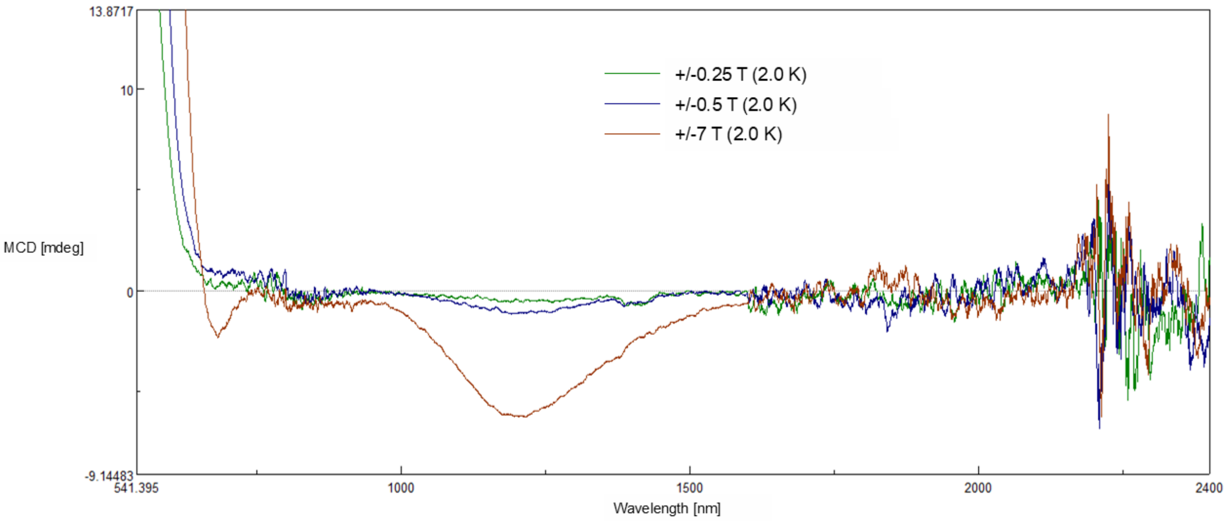

Figure S27: MCD spectra of $(PPh_4)_2[Cu(mnt)_2]$ in PMMA film at 2.0 K (NIR region of spectrum; zoomed in).

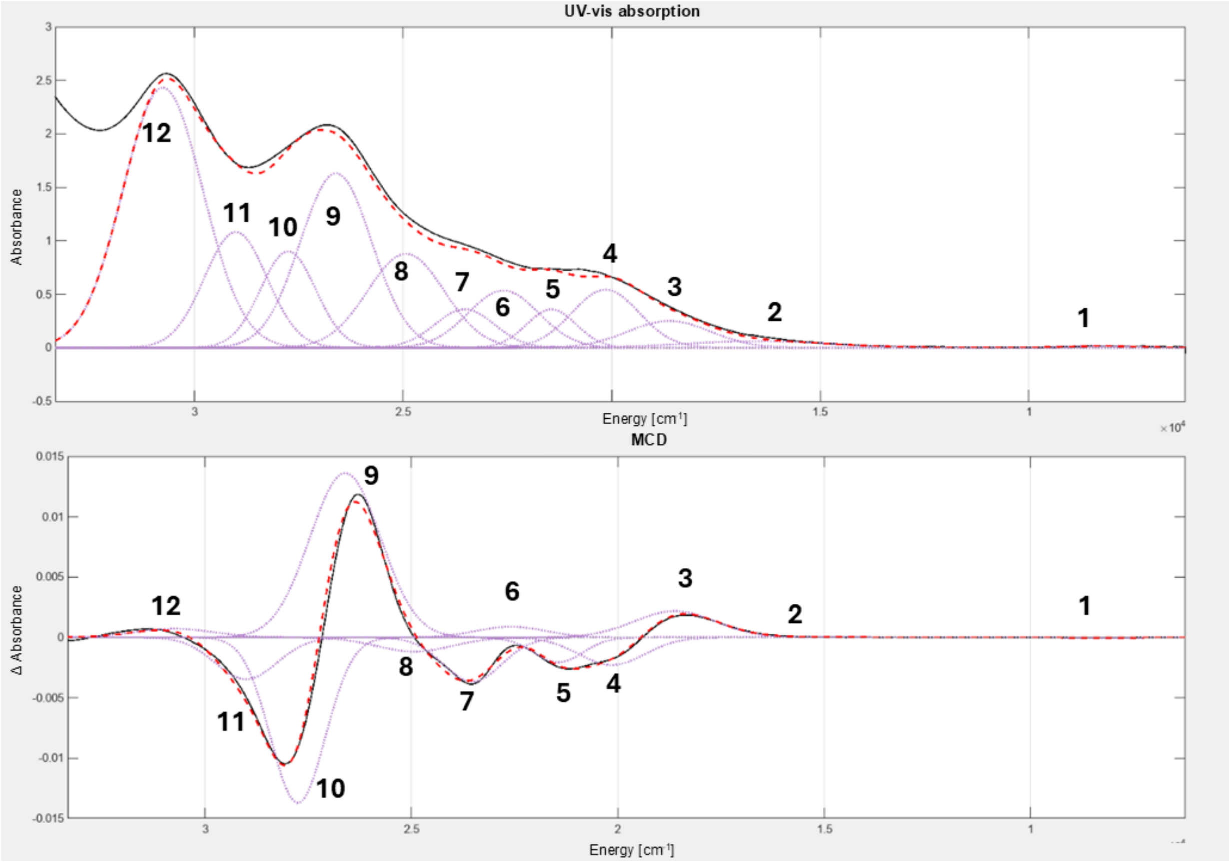

Figure S28: Gaussian band fits to $(PPh_4)_2[Cu(mnt)_2]$ in PMMA film. MCD is obtained at 2.0 K and +/-0.5 T.

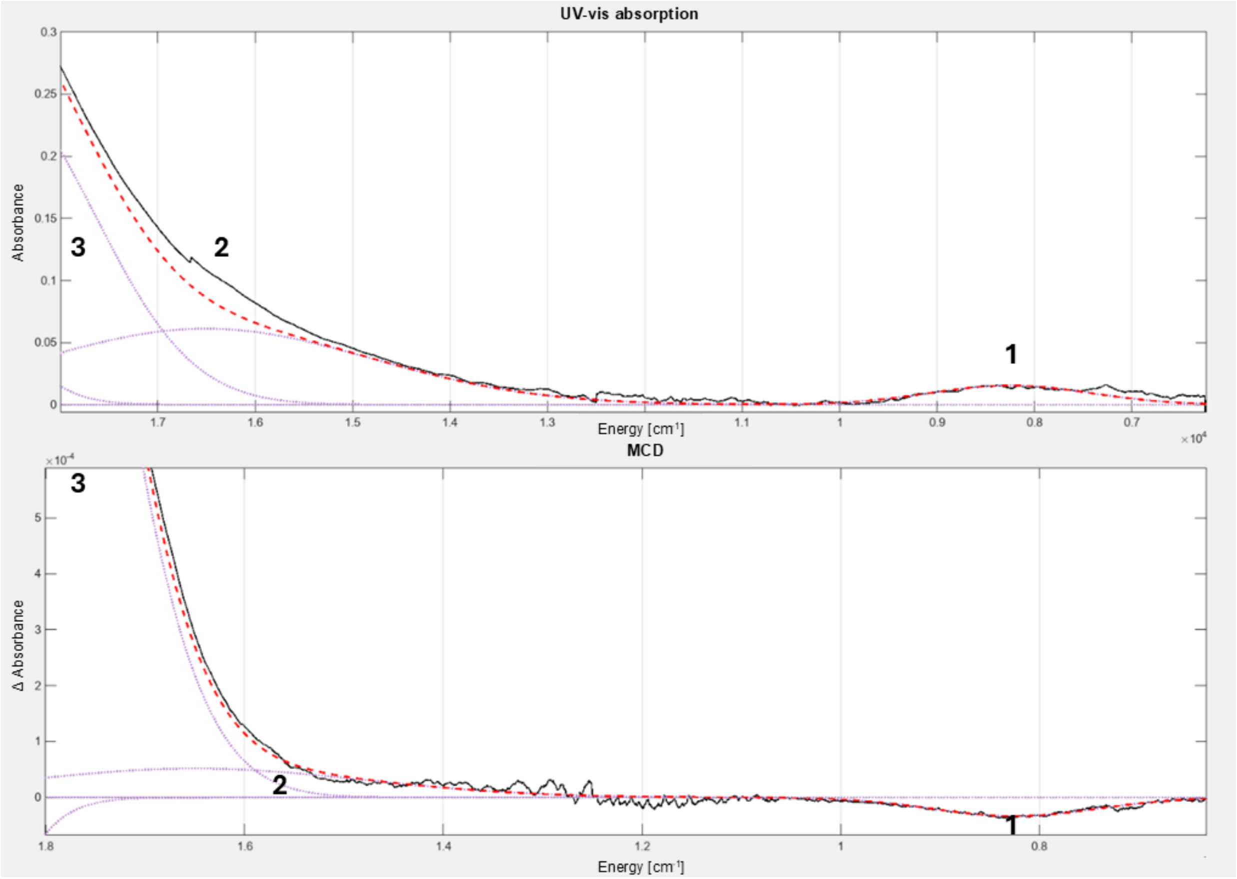

Figure S29: Gaussian band fits to $(PPh_4)_2[Cu(mnt)_2]$ in PMMA film, zoomed on NIR region to emphasize band 1. MCD is obtained at 2.0 K and +/-0.5 T.

Table S8: MCD fit parameters for $(PPh_4)_2[Cu(mnt)_2]$ in PMMA film. Assigned ligand field transitions given in bold.

Peak number	Center (cm^{-1})	FWHM (cm^{-1})	Absorption height (Abs)	MCD height (ΔAbs)	C/D @ 2.0K, 0.5T	Assignment
**1**	**8249**	**1983**	**0.016**	**-3.33E-05**	**-1.28E-02**	**d-d / MLCT (d_{xz})**
2	16506	4107	0.061	5.18E-05	5.04E-03	bandshape
**3**	**18622**	**2376**	**0.249**	**2.18E-03**	**5.21E-02**	**d-d**
**4**	**20145**	**1929**	**0.542**	**-2.28E-03**	**-2.50E-02**	**d-d / CT (mixed)**
**5**	**21443**	**1435**	**0.358**	**-2.06E-03**	**-3.43E-02**	**d-d (x^2–y^2 → xy) / CT (mixed)**
6	22592	2032	0.534	8.90E-04	9.92E-03	
7	23523	1708	0.359	-3.65E-03	**-6.07E-02**	CT / d-d (mixed)
8	24931	2199	0.878	-1.17E-03	-7.96E-03	
9	26606	2060	1.630	1.36E-02	**4.97E-02**	CT / d-d (mixed)
10	27753	1588	0.900	-1.37E-02	**-9.06E-02**	CT / d-d (mixed)
11	29009	1799	1.081	-3.47E-03	-1.91E-02	
12	30755	2303	2.431	7.18E-04	1.76E-03	

5.3.2 Cu(dtc)$_2$ in PS film

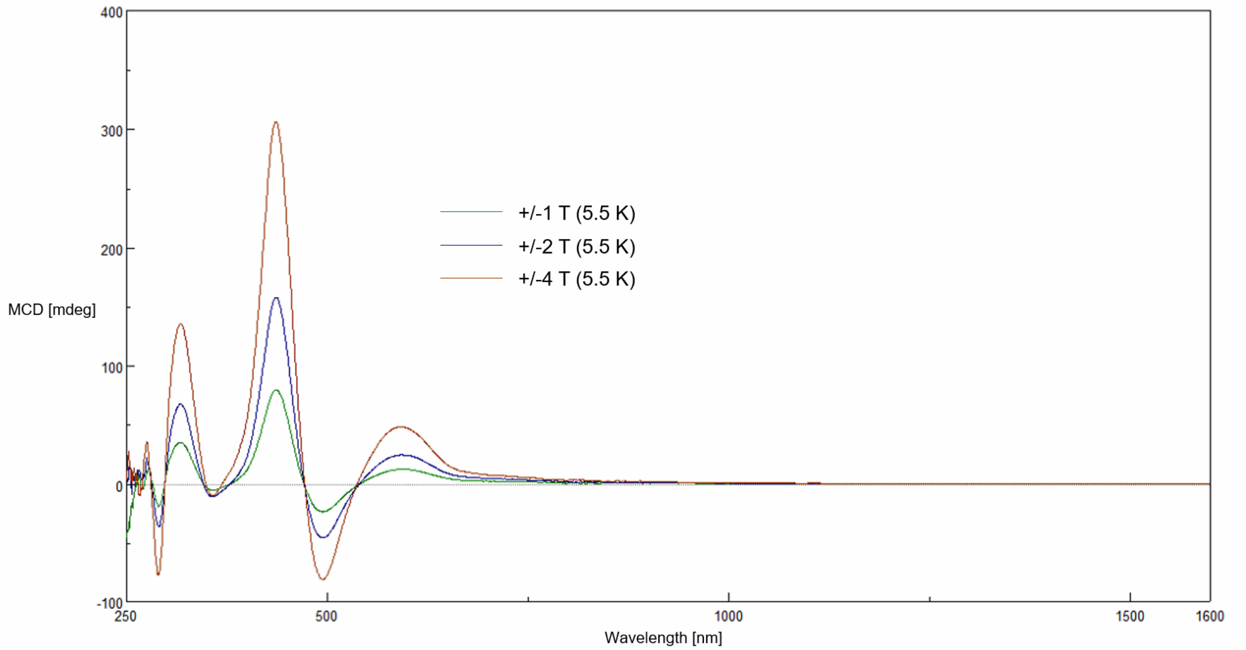

Figure S30: MCD spectra of Cu(dtc)$_2$ in PS film at 5.5 K.

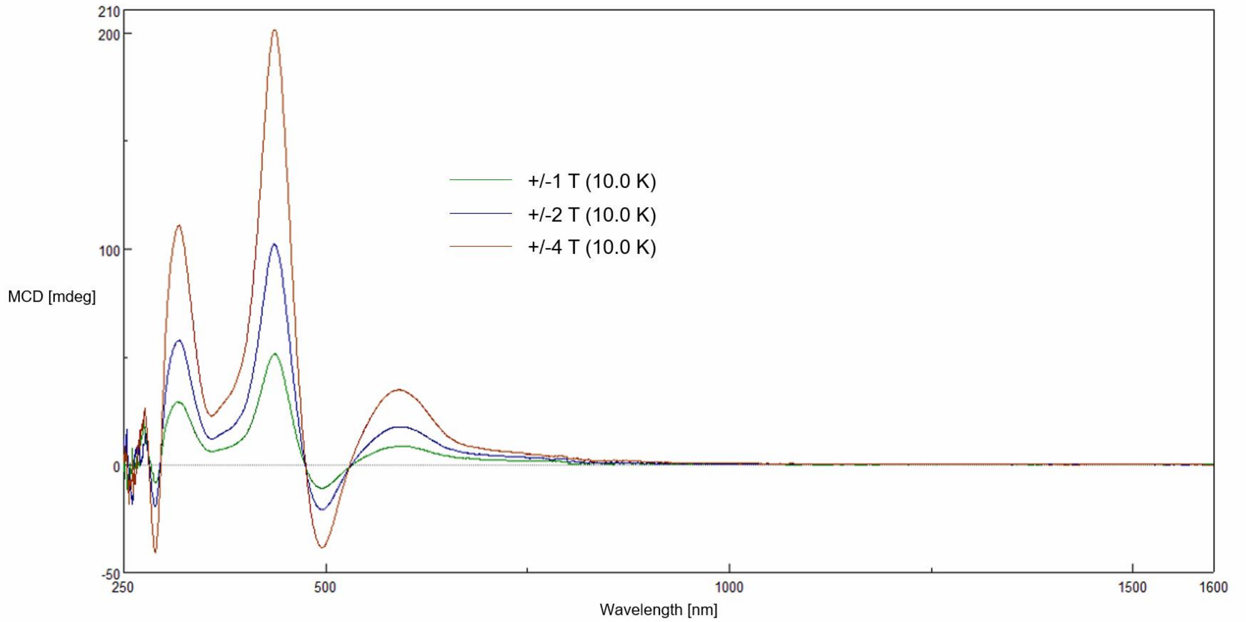

Figure S31: MCD spectra of Cu(dtc)$_2$ in PS film at 10.0 K.

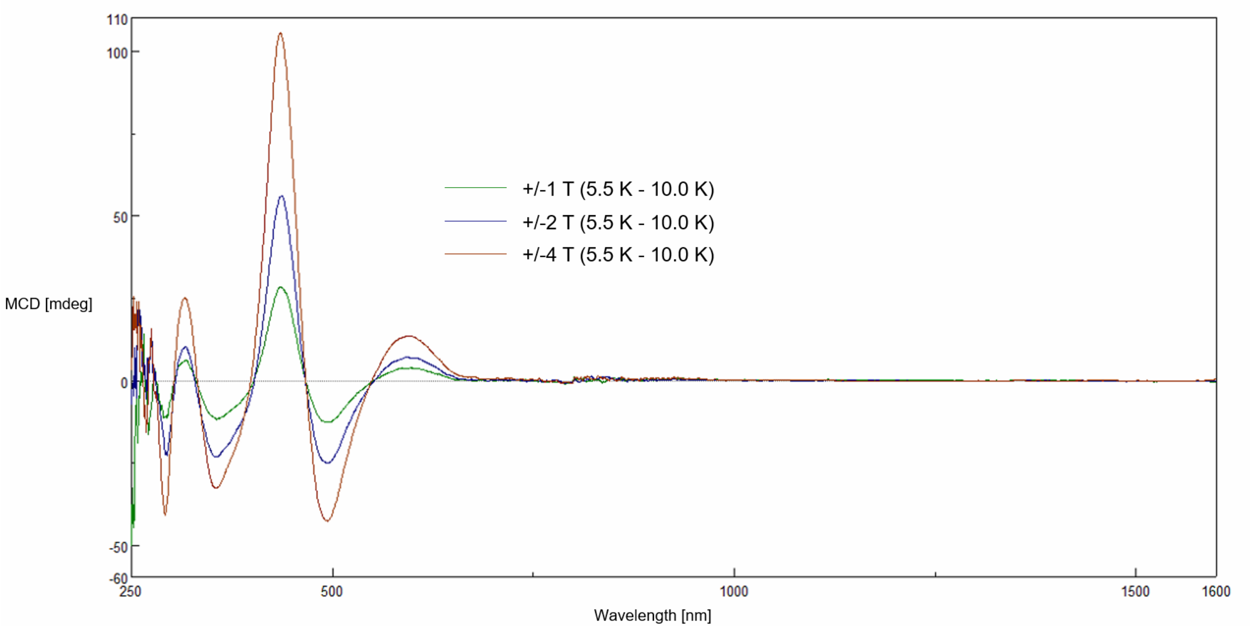

Figure S32: Pure C-term MCD spectra of $Cu(dtc)_2$ in PS film obtained by subtracting 5.5 K spectra from 10.0 K spectra.

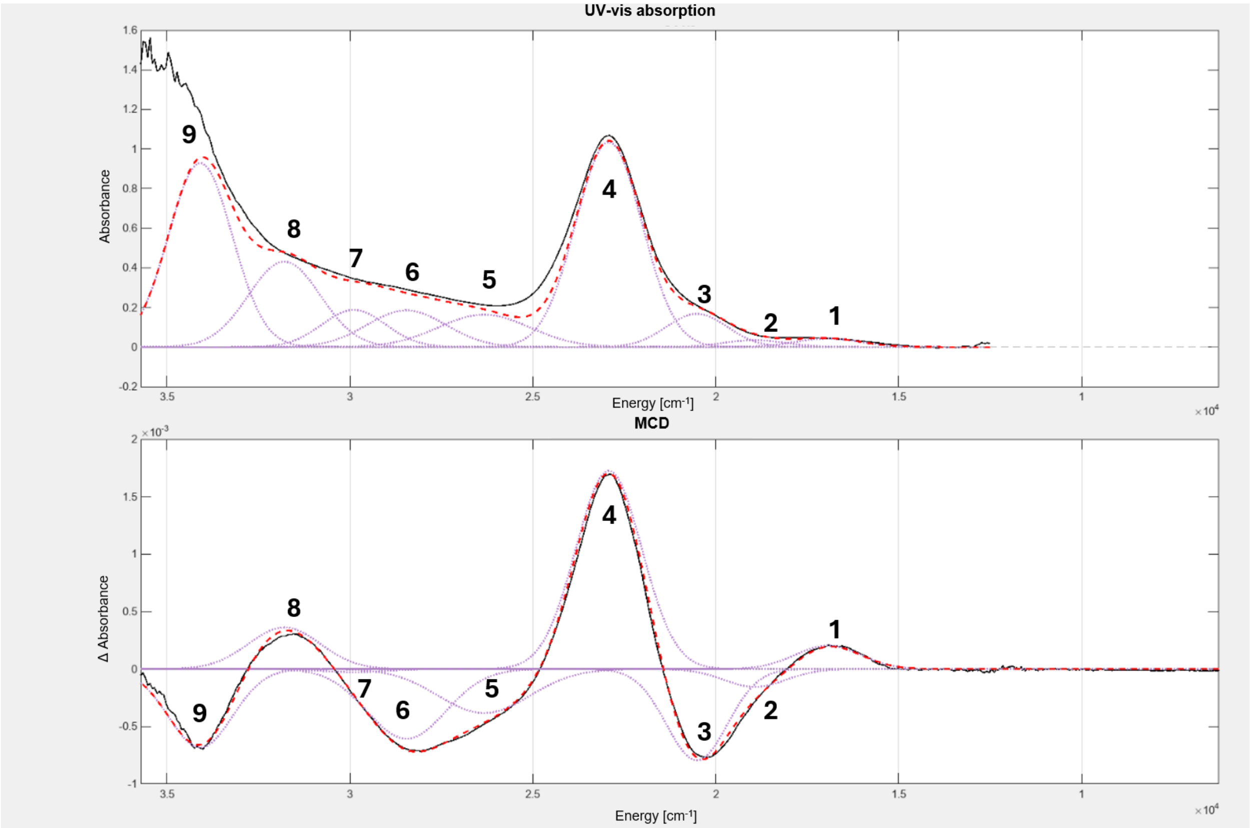

Figure S33: Gaussian band fits to $Cu(dtc)_2$ in PS film. MCD is the pure C-term spectrum obtained at +/-2 T by subtracting the 5.5 K spectrum from the 10.0 K spectrum.

Table S9: MCD fit parameters for $Cu(dtc)_2$ in PS film (see above). Assigned ligand field transitions given in bold.

Peak number	Center (cm^{-1})	FWHM (cm^{-1})	Absorption height (Abs)	MCD height (ΔAbs)	C/D @ 5.5K-10K, 2T	Assignment
**1**	**16935**	**2140**	**0.043**	**2.04E-04**	**0.045**	**d-d**
**2**	**18933**	**2037**	**0.035**	**-1.55E-04**	**-0.042**	**d-d**
**3**	**20517**	**2004**	**0.168**	**-7.93E-04**	**-0.045**	**d-d (x^2–y^2 → xy)**
4	22923	2172	1.035	1.72E-03	0.016	
5	26334	3003	0.163	-3.83E-04	-0.022	CT (mixed)
6	28454	2584	0.185	-6.07E-04	-0.031	CT (mixed)
7	29891	2094	0.187	-2.68E-05	-0.001	
8	31791	2357	0.431	3.62E-04	0.008	
9	34086	2098	0.929	-6.82E-04	-0.007	

5.3.3 Cu(pci)₂ in PS film

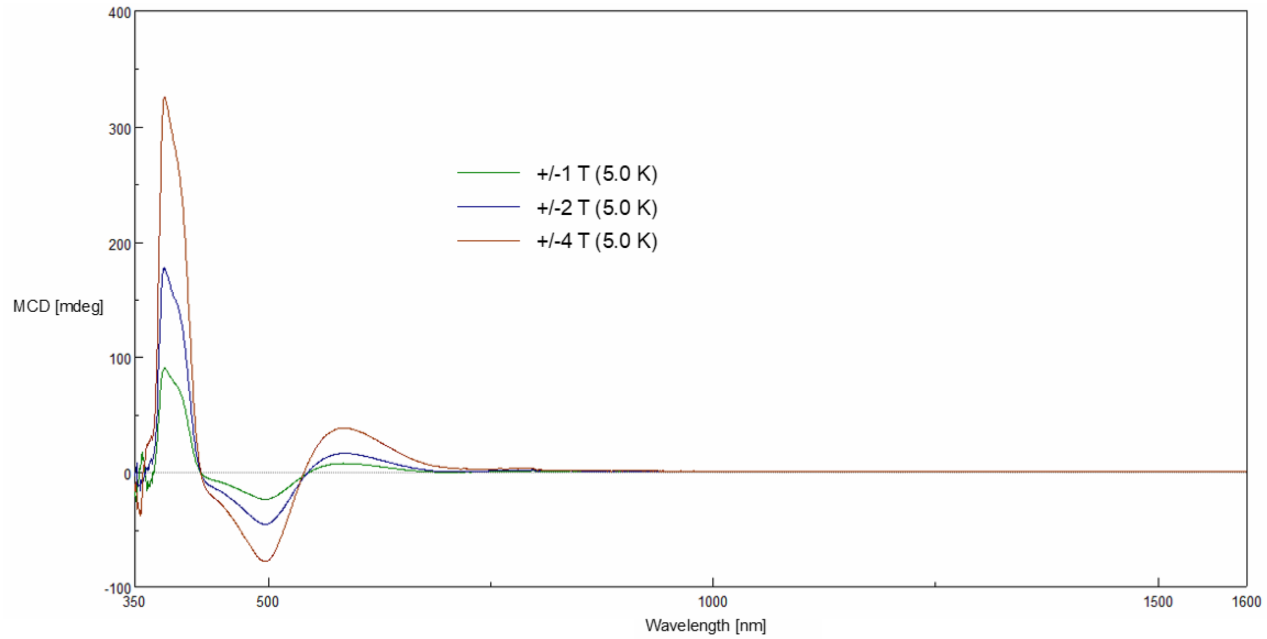

Figure S34: MCD spectra of $Cu(pci)_2$ in PS film at 5.0 K.

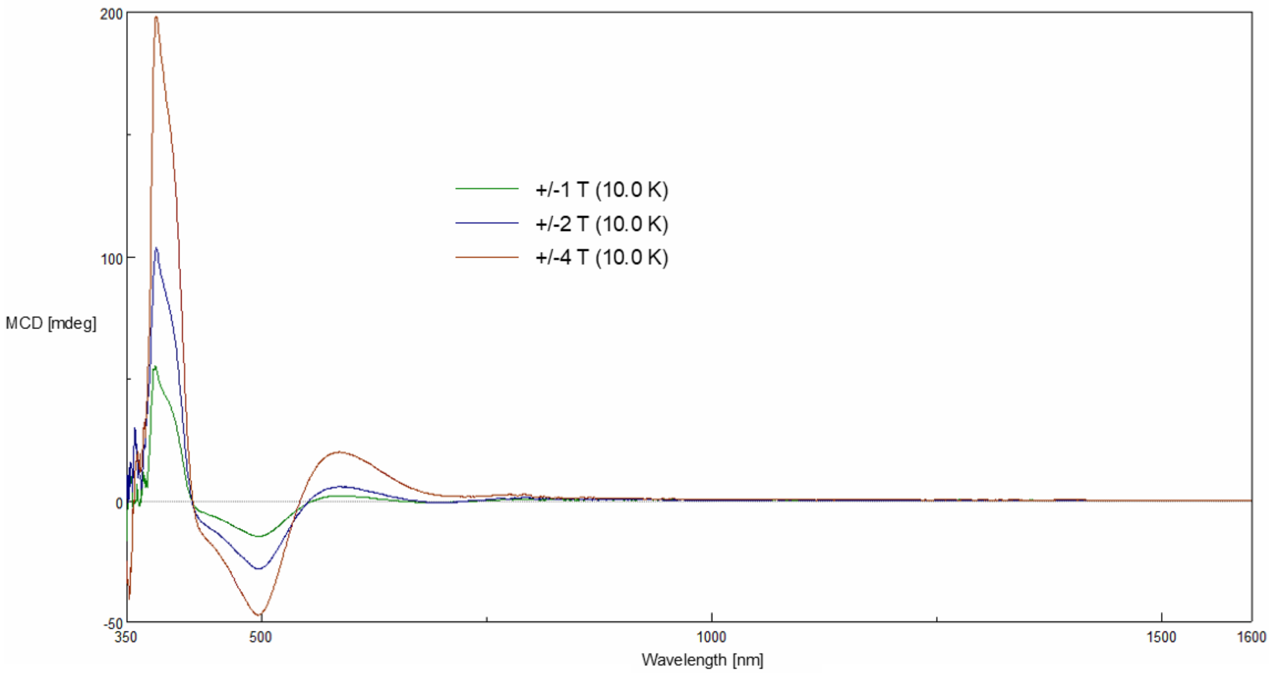

Figure S35: MCD spectra of $Cu(pci)_2$ in PS film at 10.0 K.

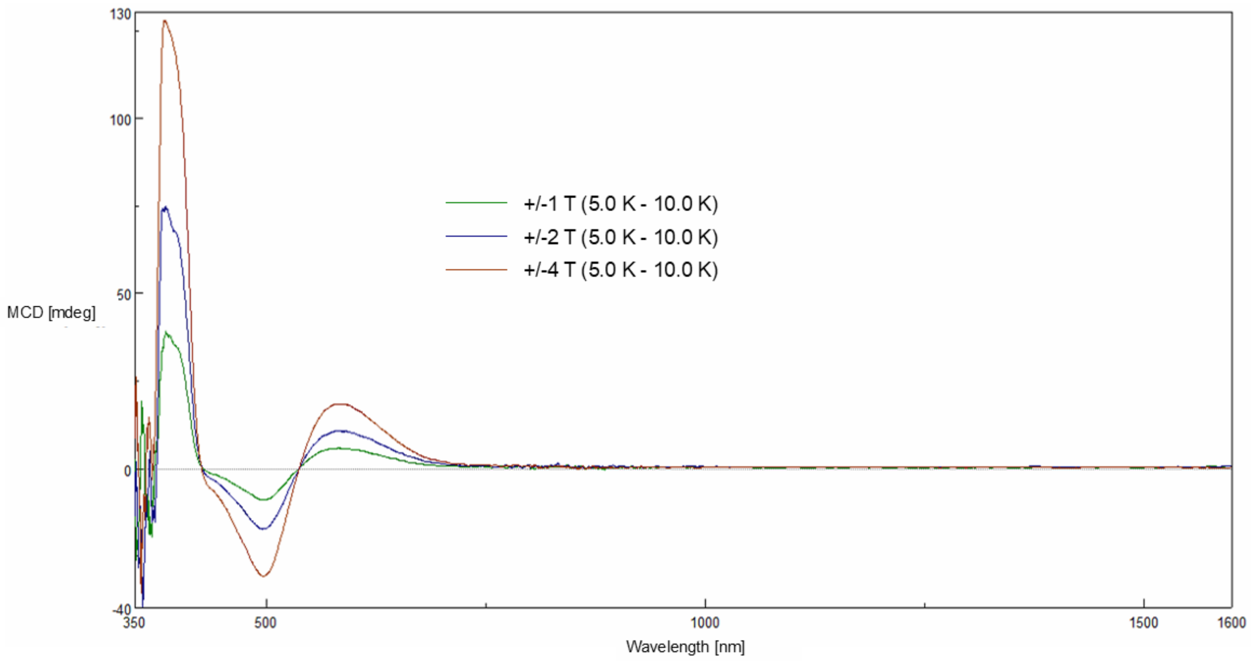

Figure S36: Pure C-term MCD spectra of Cu(pci)$_2$ in PS film obtained by subtracting 5.0 K spectra from 10.0 K spectra.

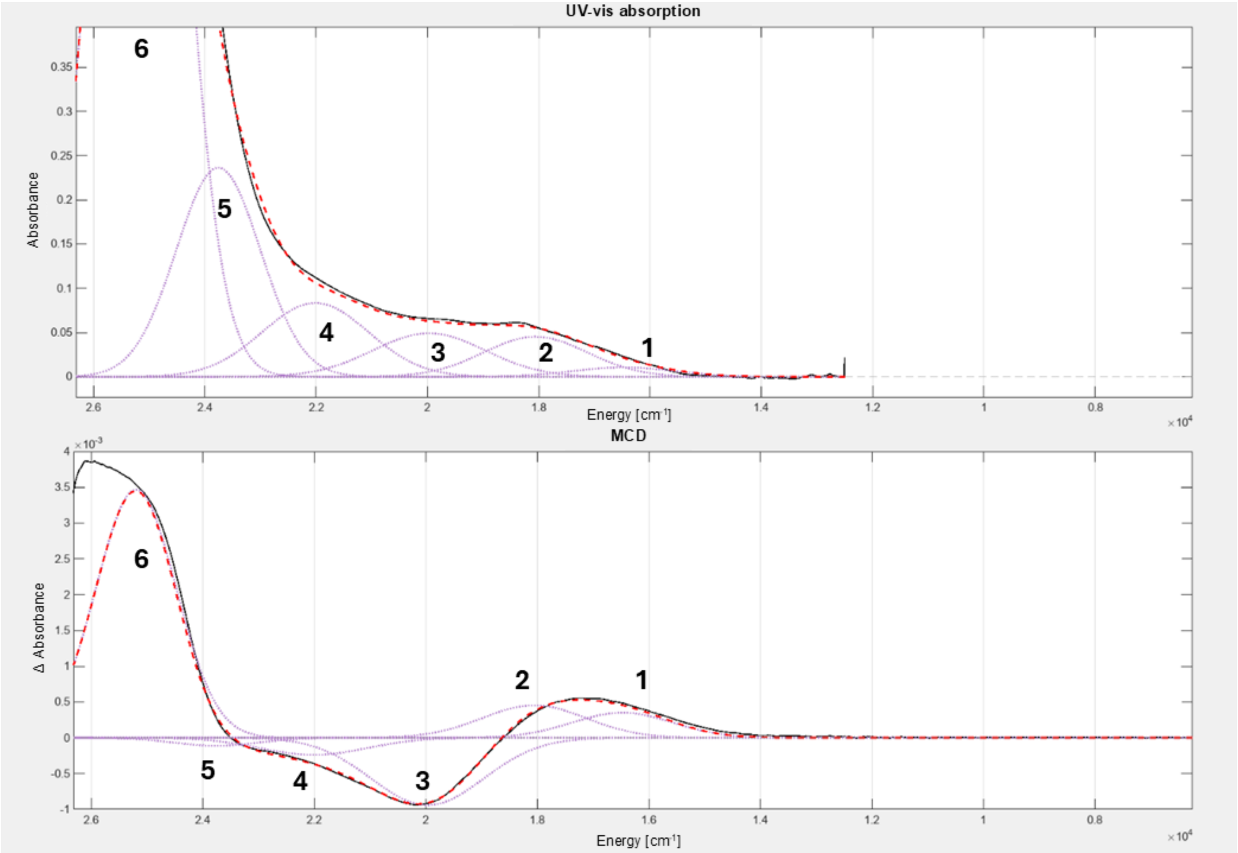

Figure S37: Gaussian band fits to Cu(pci)$_2$ in PS film. MCD is the pure C-term spectrum obtained at +/-4 T by subtracting the 5.0 K spectrum from the 10.0 K spectrum.

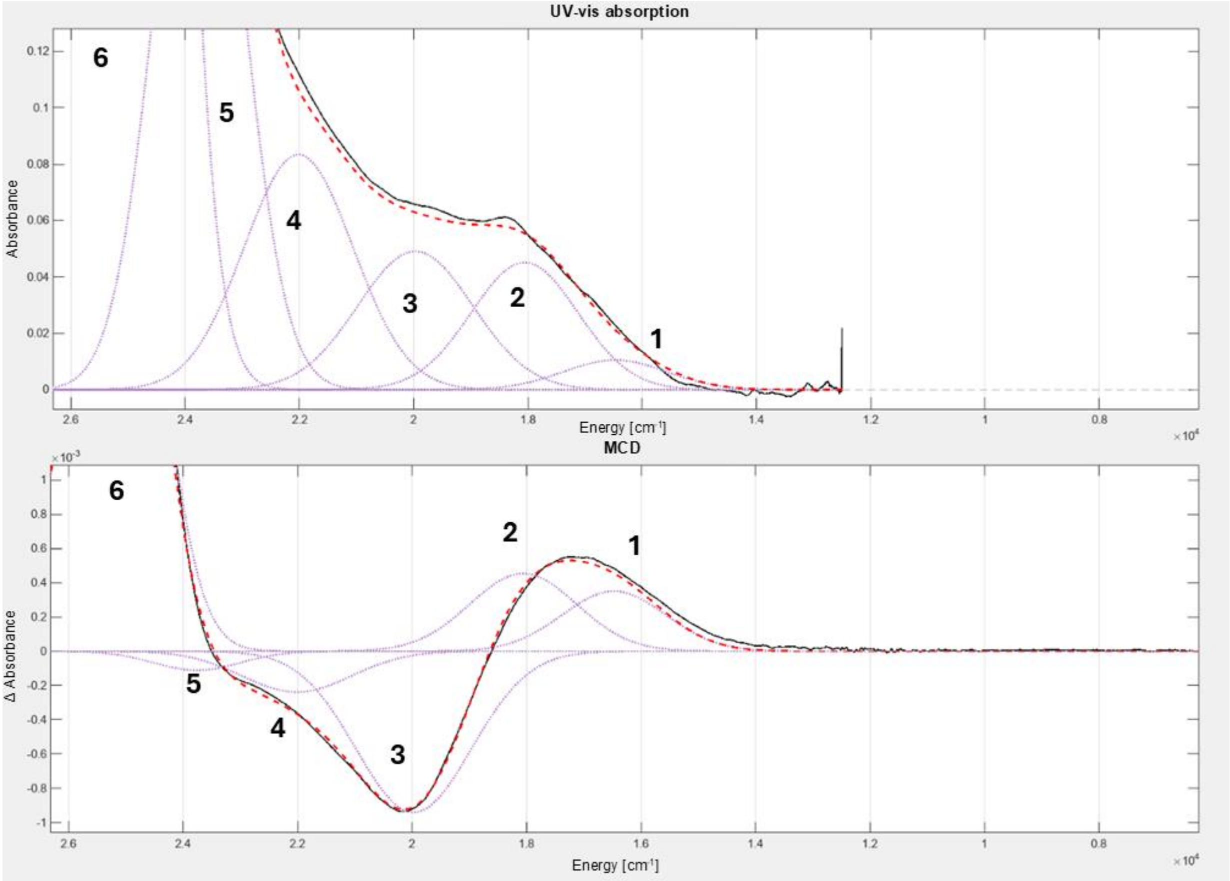

Figure S38: Gaussian band fits to Cu(pci)$_2$ in PS film (zoomed-in on y-axis). MCD is the pure C-term spectrum obtained at +/-4 T by subtracting the 5.0 K spectrum from the 10.0 K spectrum.

Table S10: MCD fit parameters for Cu(pci)$_2$ in PS film (see above). Assigned ligand field transitions given in bold.

Peak number	Center (cm^{-1})	FWHM (cm^{-1})	Absorption height (Abs)	MCD height (ΔAbs)	C/D @ 5.0K-10K, 4T	Assignment
**1**	**16472**	**2169**	**0.011**	**3.5E-04**	**0.146**	**d-d**
**2**	**18056**	**2242**	**0.045**	**4.5E-04**	**0.044**	**d-d**
**3**	**19970**	**2419**	**0.049**	**-9.4E-04**	**-0.084**	**d-d (x^2–y^2 → xy)**
4	22013	2288	0.083	-2.4E-04	-0.013	
5	23755	1816	0.236	-1.1E-04	-0.002	
6	25195	1723	1.138	3.5E-03	0.013	

5.3.4 Cu(acacen) in PS film

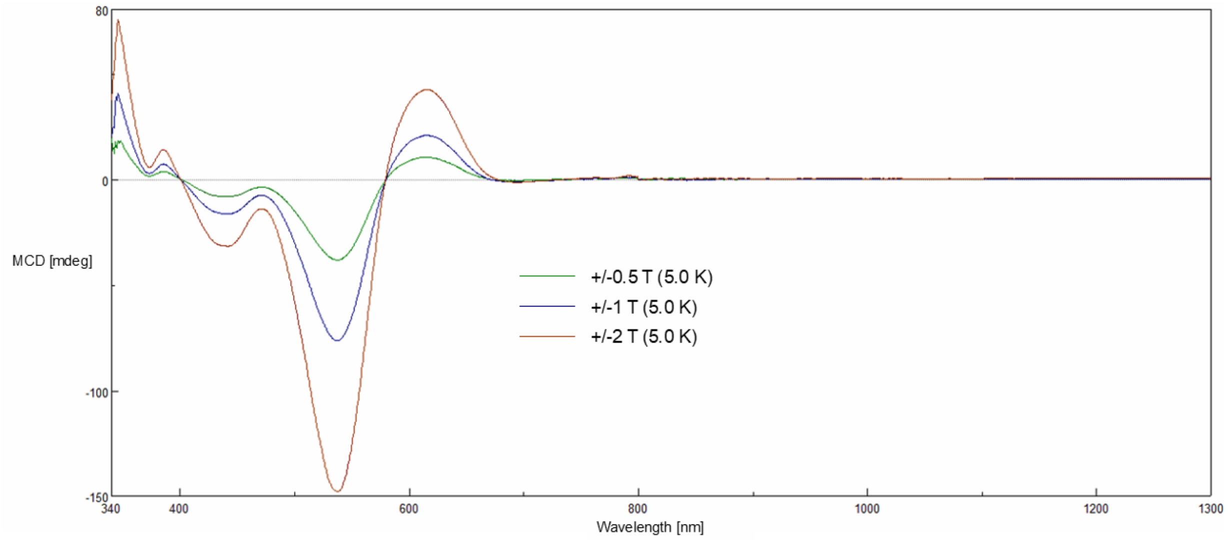

Figure S39: MCD spectra of Cu(acacen) in PS film at 5.0 K.

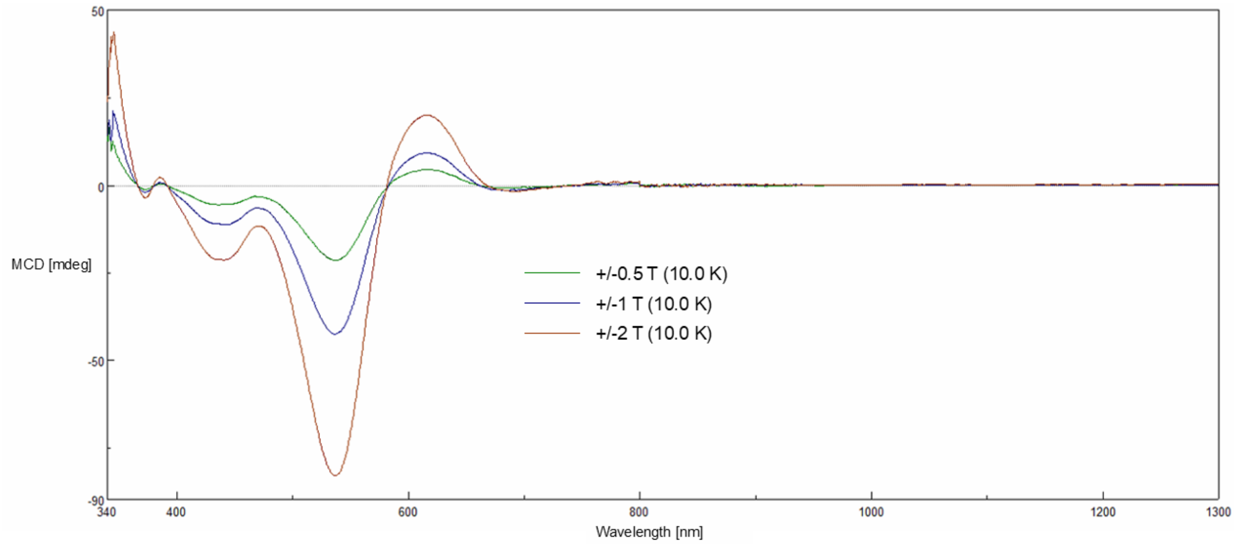

Figure S40: MCD spectra of Cu(acacen) in PS film at 10.0 K.

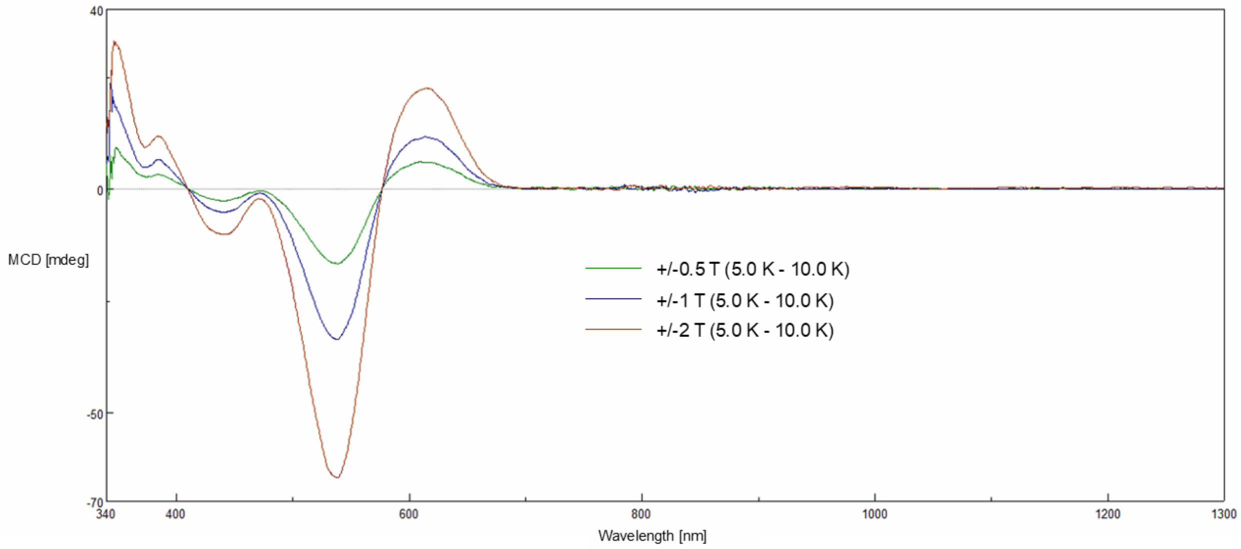

Figure S41: Pure C-term MCD spectra of Cu(acacen) in PS film obtained by subtracting 5.0 K spectra from 10.0 K spectra.

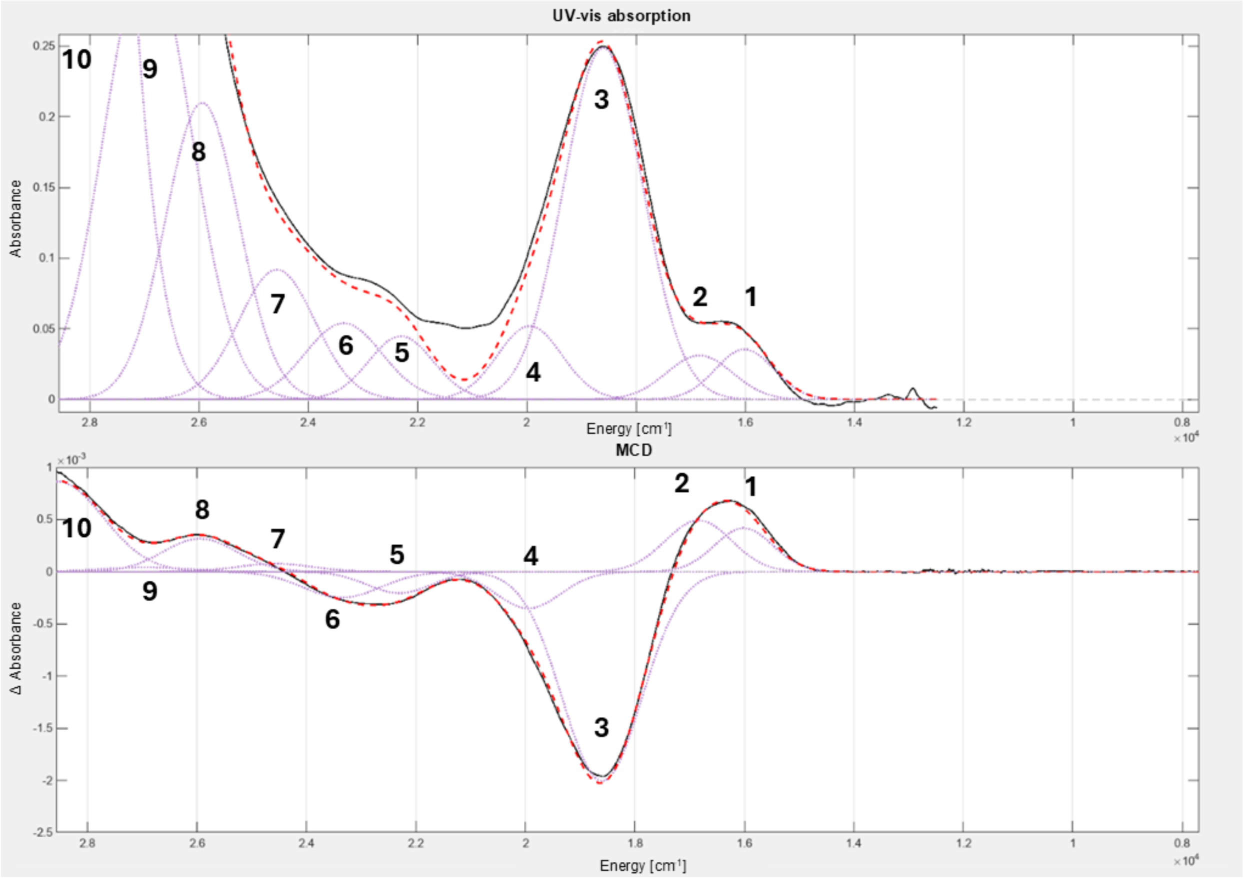

Figure S42: Gaussian band fits to Cu(acacen) in PS film. MCD is the pure C-term spectrum obtained at +/-2 T by subtracting the 5.0 K spectrum from the 10.0 K spectrum.

Table S11: MCD fit parameters for Cu(acacen) in PS film (see above). Assigned ligand field transitions given in bold.

Peak number	Center (cm^{-1})	FWHM (cm^{-1})	Absorption height (Abs)	MCD height (ΔAbs)	C/D @ 5.0K-10K, 2T	Assignment
**1**	**16013**	**1280**	**0.035**	**4.17E-04**	**9.1E-02**	**d-d**
**2**	**16837**	**1451**	**0.031**	**4.92E-04**	**1.2E-01**	**d-d**
**3**	**18601**	**1743**	**0.249**	**-2.00E-03**	**-6.2E-02**	**d-d (x^2–y^2 → xy)**
**4**	**19946**	**1416**	**0.052**	**-3.51E-04**	**-5.2E-02**	**d-d**
5	22303	1416	0.045	-2.05E-04	-3.5E-02	
6	23355	1681	0.054	-2.48E-04	-3.6E-02	
7	24576	1673	0.092	7.63E-05	6.4E-03	
8	25944	1614	0.210	3.17E-04	1.2E-02	
9	26938	1897	0.317	4.28E-05	1.0E-03	
10	28542	1982	1.157	8.63E-04	5.8E-03	

5.3.5 $Cu(tbaa)_2$ in PS film

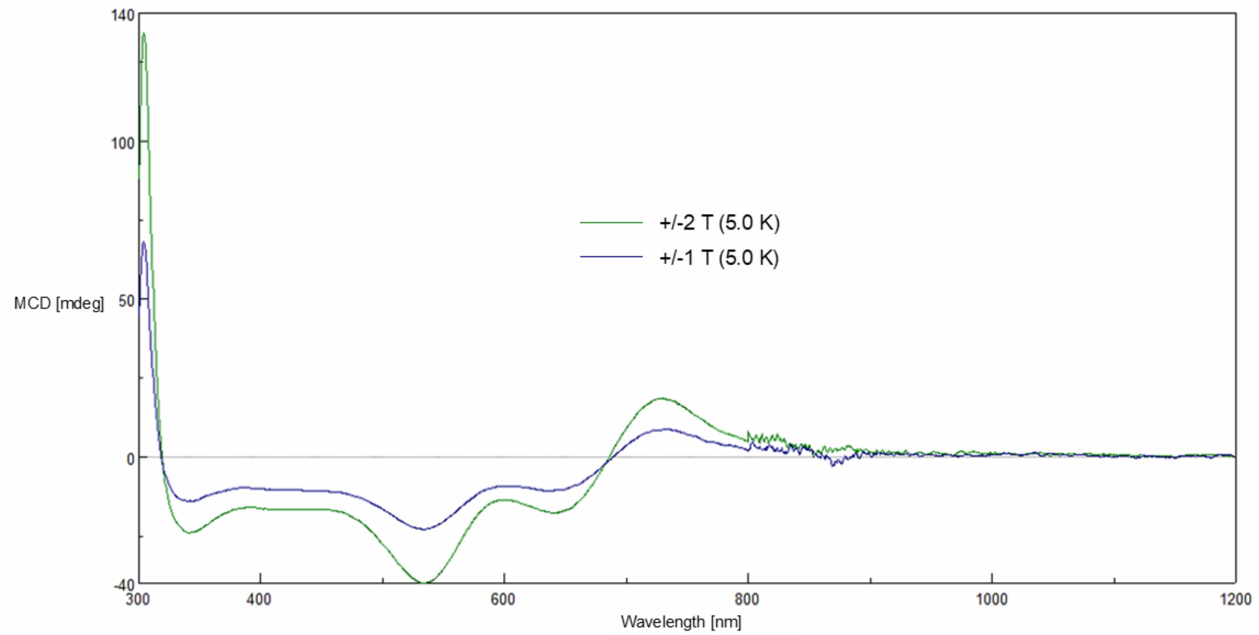

Figure S43: MCD spectra of $Cu(tbaa)_2$ in PS film at 5.0 K.

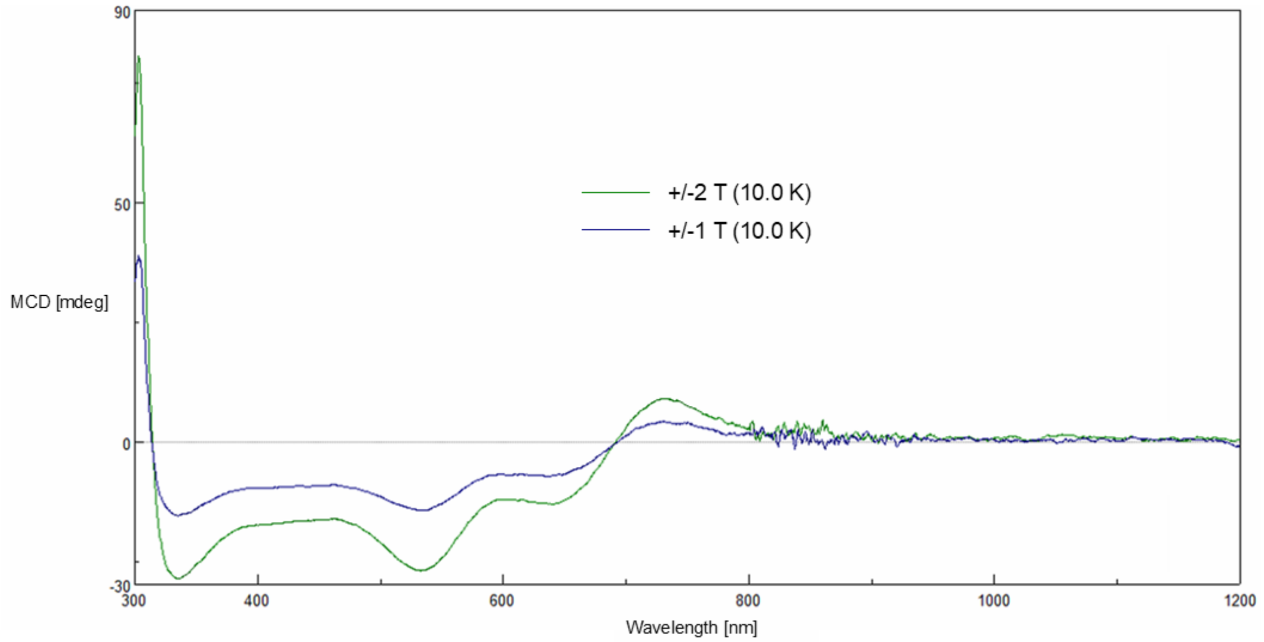

Figure S44: MCD spectra of $Cu(tbaa)_2$ in PS film at 10.0 K.

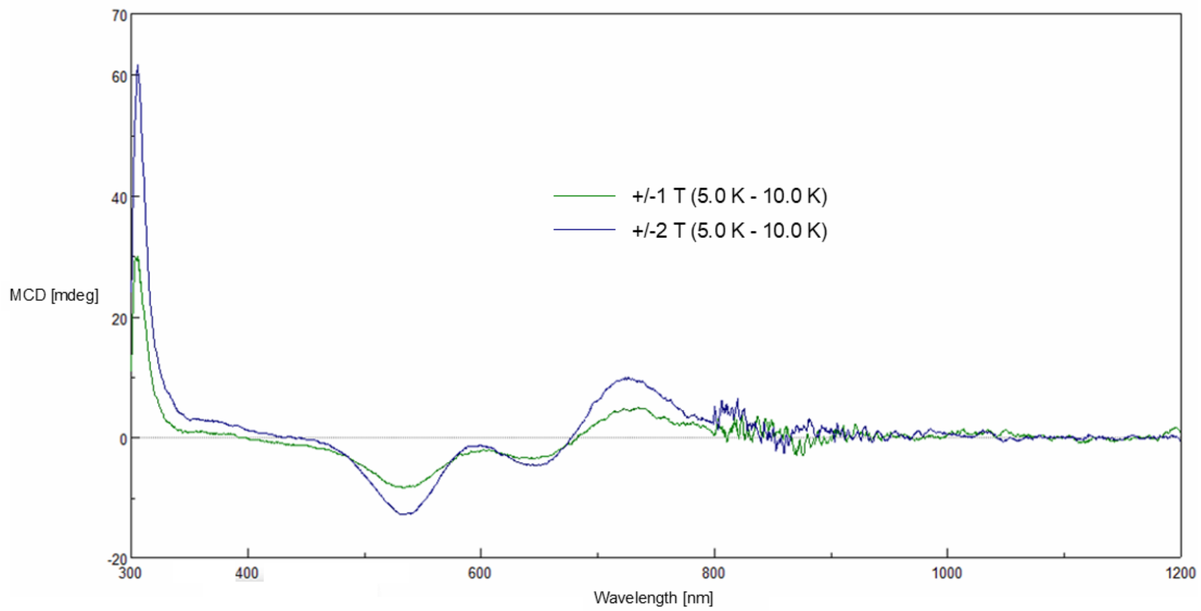

Figure S45: Pure C-term MCD spectra of Cu(tbaa)₂ in PS film obtained by subtracting 5.0 K spectra from 10.0 K spectra.

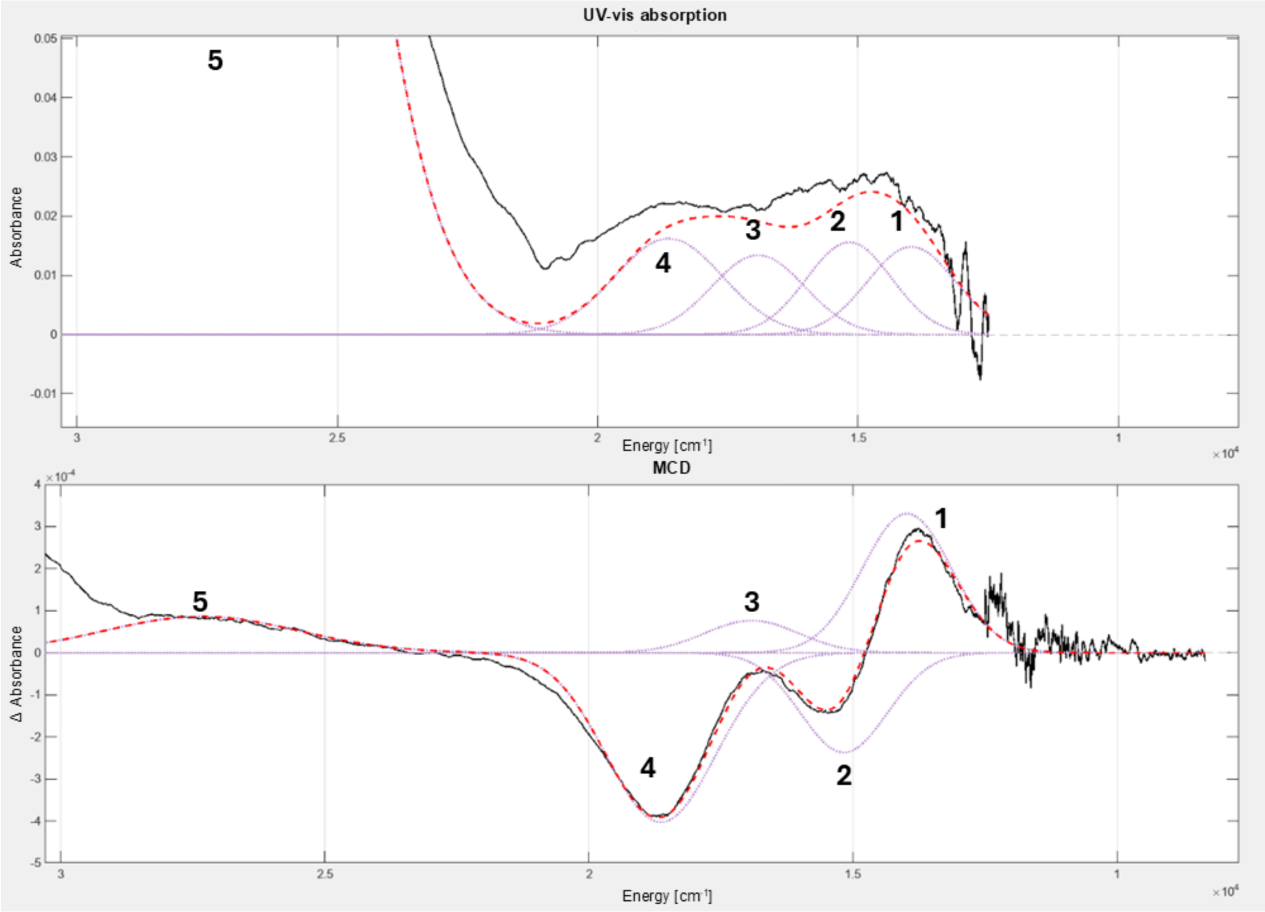

Figure S46: Gaussian band fits to Cu(tbaa)₂ in PS film. MCD is the pure C-term spectrum obtained at +/-2 T by subtracting the 5.0 K spectrum from the 10.0 K spectrum.

Table S12: MCD fit parameters for Cu(tbaa)$_2$ in PS film (see above). Assigned ligand field transitions given in bold.

Peak number	Center (cm^{-1})	FWHM (cm^{-1})	Absorption height (Abs)	MCD height (ΔAbs)	C/D @ 5.0K-10K, 2T	Assignment
**1**	**13977**	**2031**	**0.015**	**3.31E-04**	**1.8E-01**	**d-d**
**2**	**15171**	**1971**	**0.016**	**-2.37E-04**	**-1.2E-01**	**d-d**
**3**	**16919**	**2136**	**0.013**	**7.63E-05**	**4.4E-02**	**d-d**
**4**	**18637**	**2484**	**0.016**	**-4.03E-04**	**-1.9E-01**	**d-d (x^2–y^2 → xy)**
5	27337	4422	0.298	8.60E-05	2.2E-03	

5.3.6 Cu(tmhd)$_2$ in PMMA film

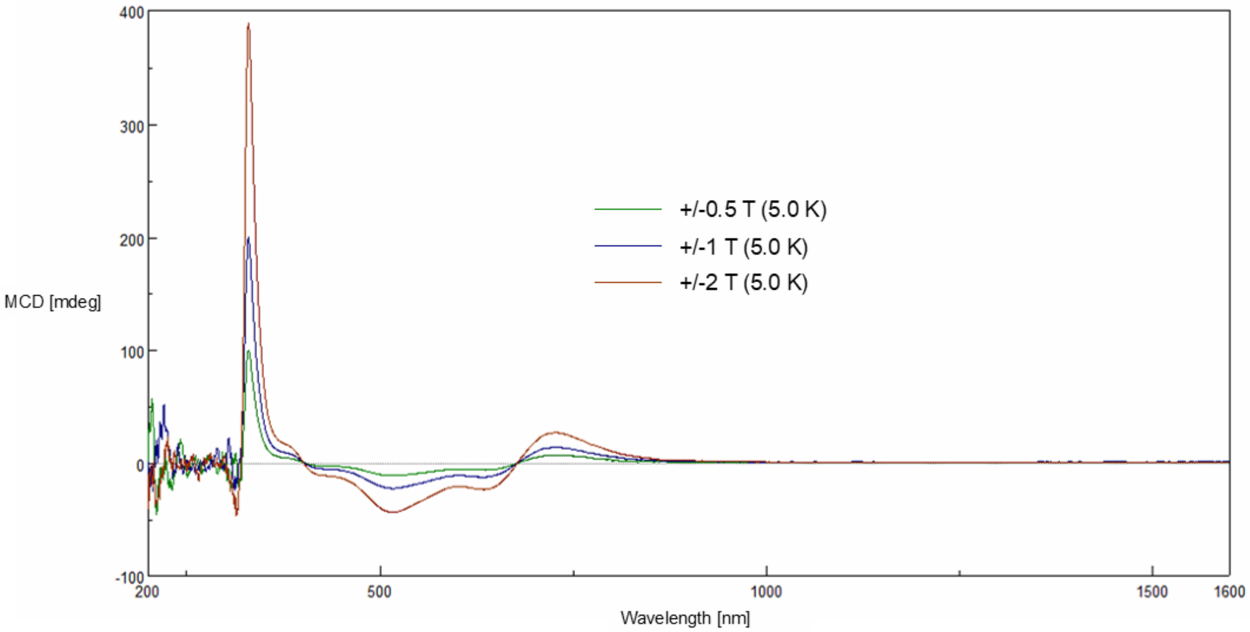

Figure S47: MCD spectra of Cu(tmhd)$_2$ in PMMA film at 5.0 K.

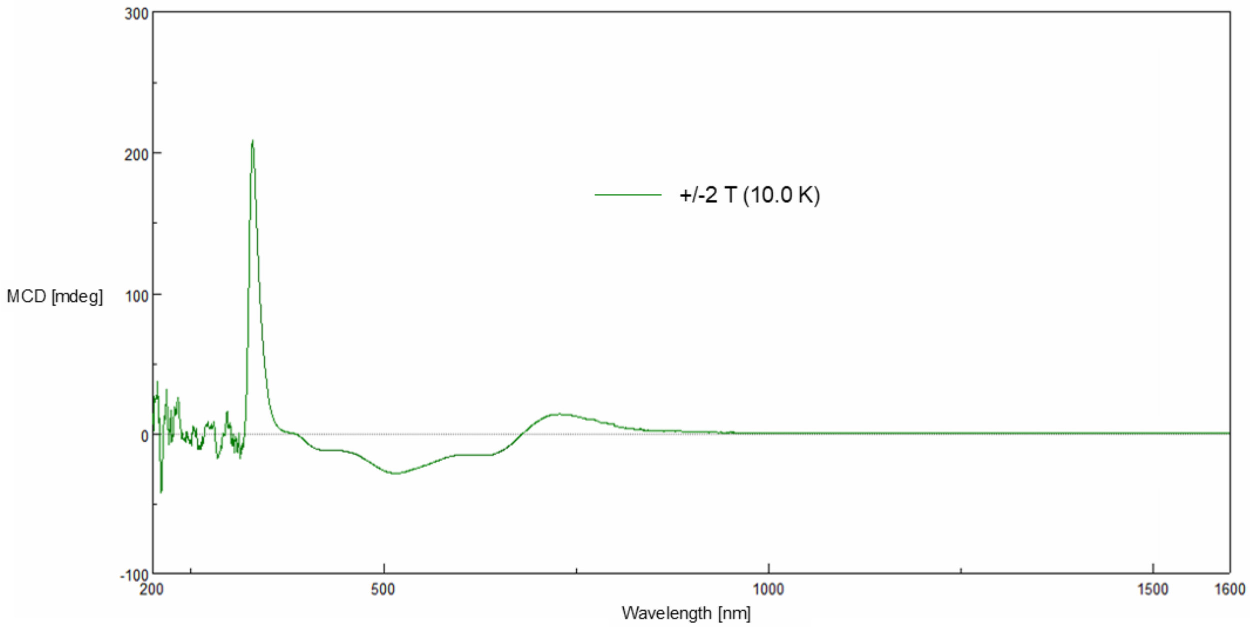

Figure S48: MCD spectra of Cu(tmhd)$_2$ in PMMA film at 10.0 K.

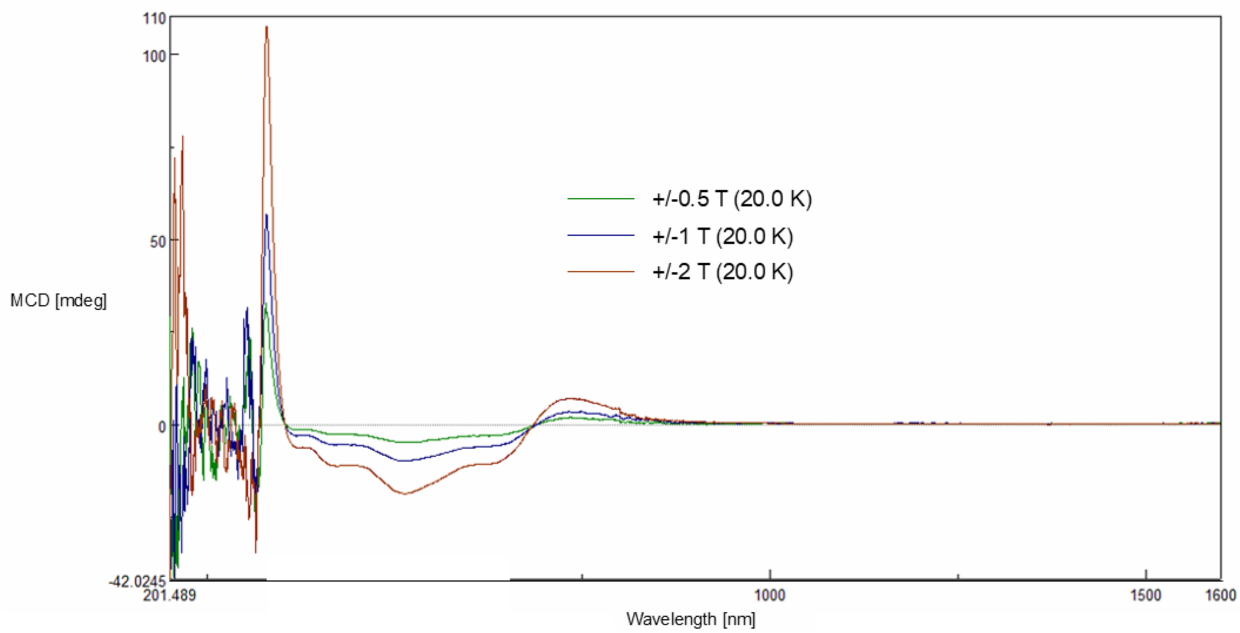

Figure S49: MCD spectra of $Cu(tmhd)_2$ in PMMA film at 20.0 K.

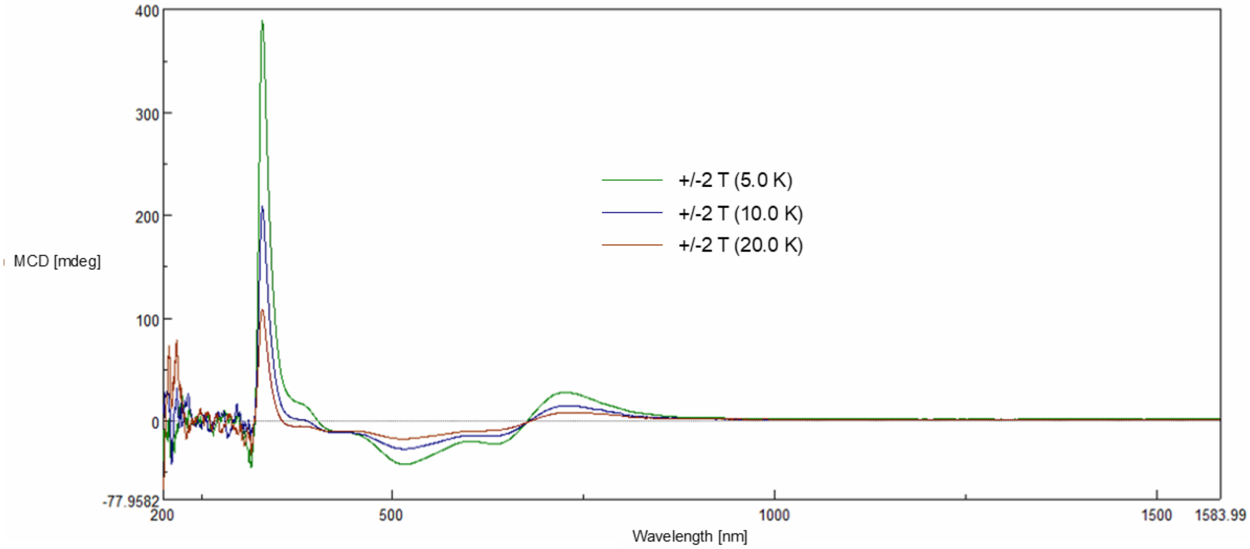

Figure S50: Comparison of MCD spectra of $Cu(tmhd)_2$ in PMMA film at +/-2T.

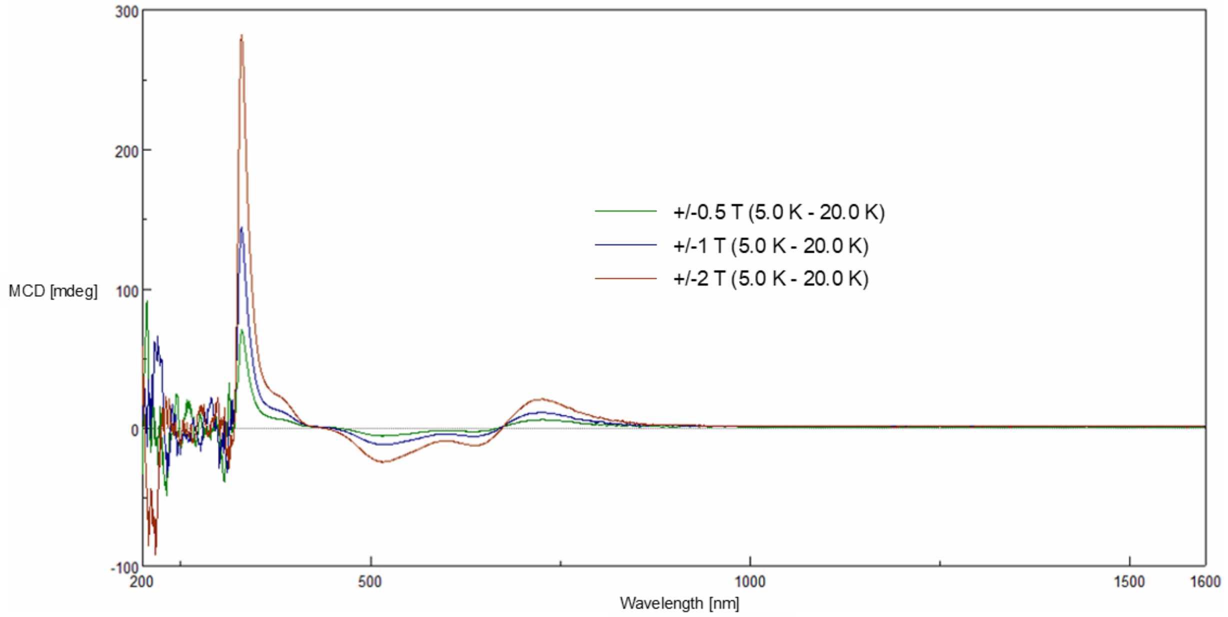

Figure S51: Pure C-term MCD spectra of Cu(tmhd)₂ in PMMA film obtained by subtracting 5.0 K spectra from 20.0 K spectra.

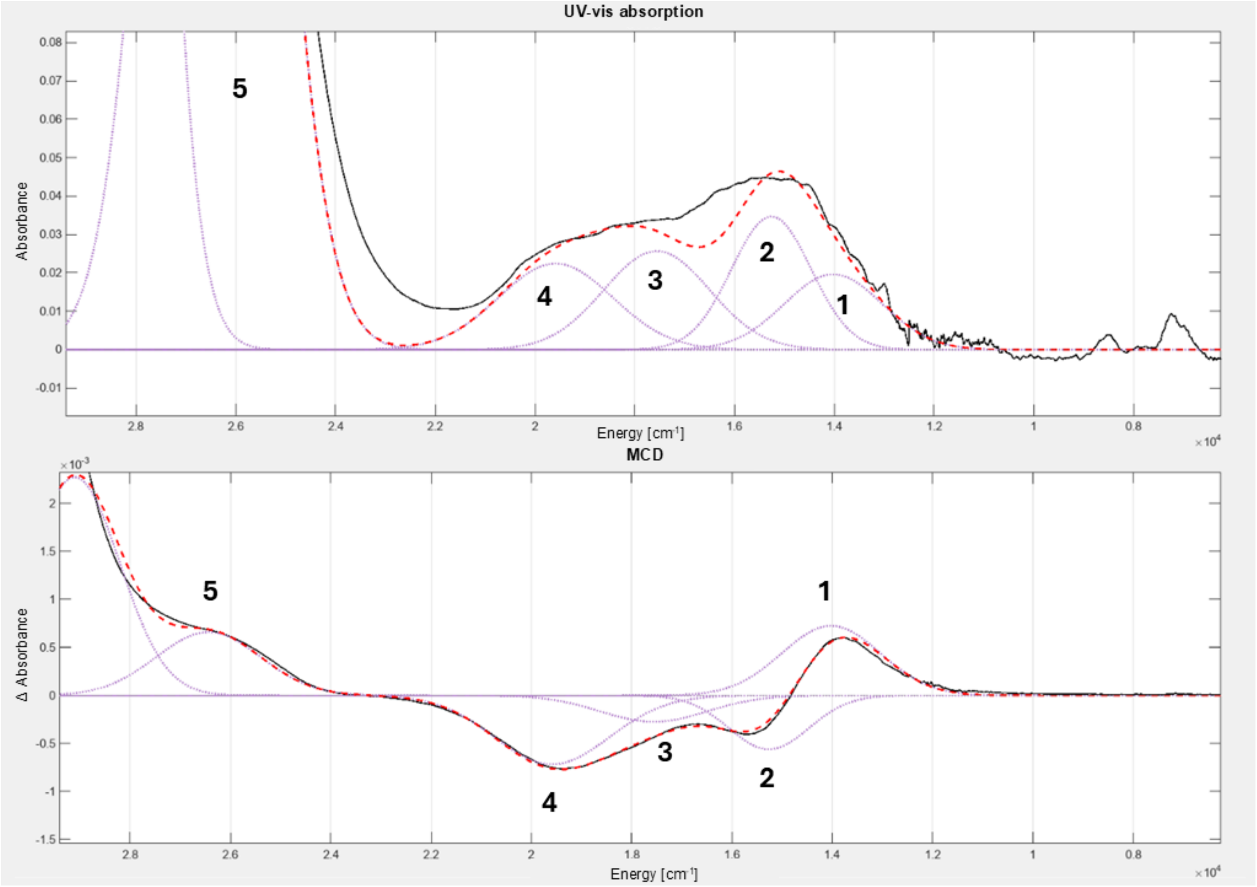

Figure S52: Gaussian band fits to Cu(tmhd)₂ in PMMA film. MCD is the pure C-term spectrum obtained at +/-2 T by subtracting the 5.0 K spectrum from the 20.0 K spectrum.

Table S13: MCD fit parameters for Cu(tmhd)$_2$ in PMMA film (see above). Assigned ligand field transitions given in bold.

Peak number	Center (cm^{-1})	FWHM (cm^{-1})	Absorption height (Abs)	MCD height (ΔAbs)	C/D @ 5.0K-20K, 2T	Assignment
**1**	**14021**	**2362**	**0.020**	**7.26E-04**	**1.9E-01**	**d-d**
**2**	**15262**	**1917**	**0.035**	**-5.61E-04**	**-8.3E-02**	**d-d**
**3**	**17561**	**2549**	**0.026**	**-2.74E-04**	**-5.5E-02**	**d-d**
**4**	**19586**	**2748**	**0.022**	**-7.18E-04**	**-1.6E-01**	**d-d (x^2–y^2 → xy)**
5	26437	2501	0.382	6.61E-04	8.9E-03	

5.3.7 $(PPN)_2[Cu(ox)_2]$ in PS film

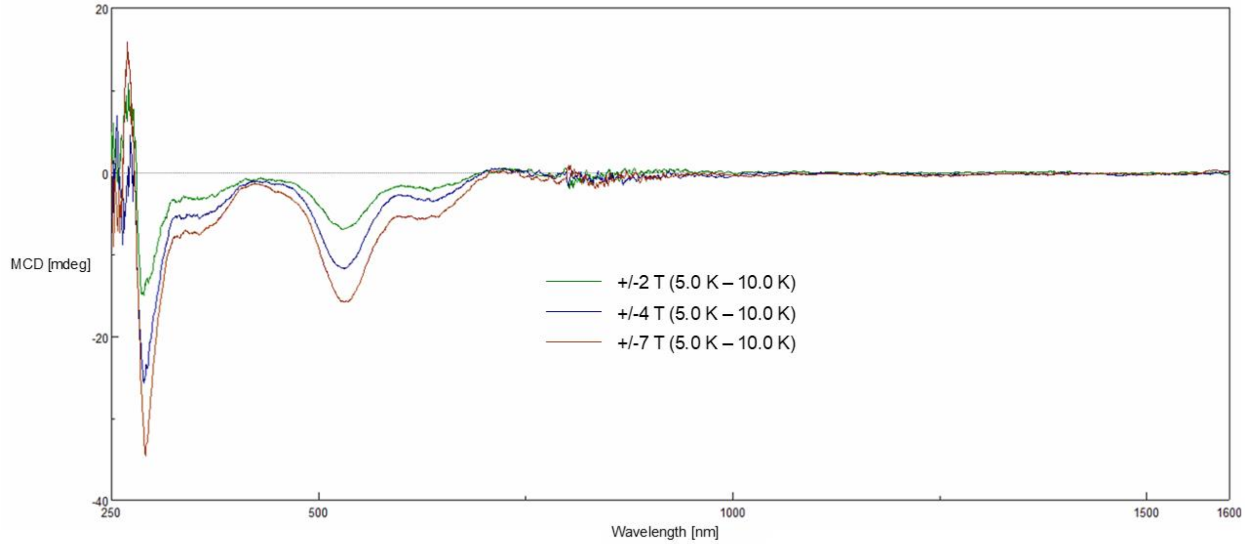

Figure S53: Pure C-term MCD spectra of $(PPN)_2[Cu(ox)_2]$ in PS film obtained by subtracting 5.0 K spectra from 10.0 K spectra.

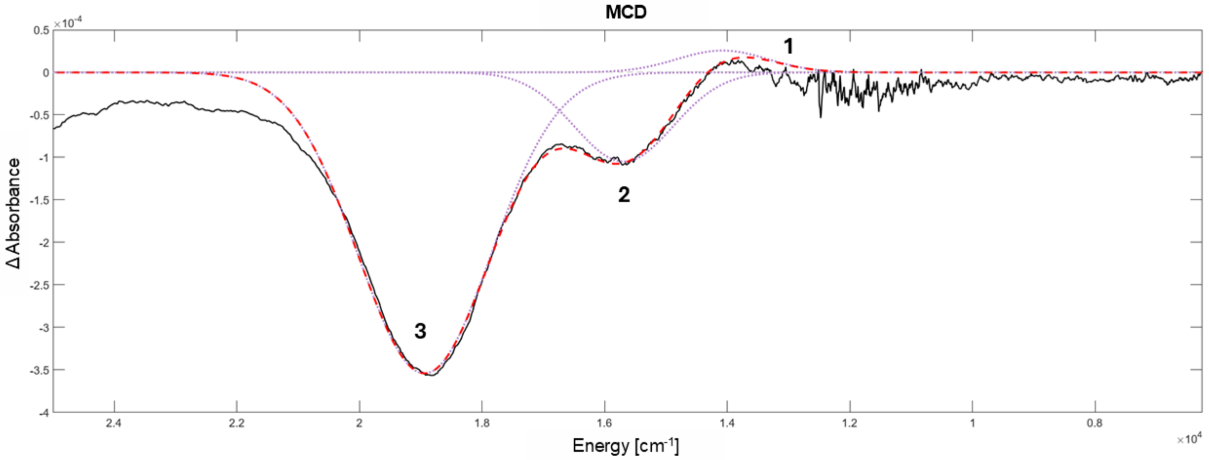

Figure S54: Gaussian band fits to $(PPN)_2[Cu(ox)_2]$ in PS film. MCD is the pure C-term spectrum obtained at +/-4 T by subtracting the 5.0 K spectrum from the 10.0 K spectrum.

Table S14: MCD fit parameters for $(PPN)_2[Cu(ox)_2]$ in PS film (see above). Assigned ligand field transitions given in bold.

Peak number	Center (cm^{-1})	FWHM (cm^{-1})	MCD height (ΔAbs)	Assignment
**1**	**14079**	**1777**	**2.57E-05**	**d-d**
**2**	**15690**	**1932**	**-1.05E-04**	**d-d**
**3**	**18938**	**2605**	**-3.54E-04**	**d-d (x^2–y^2 → xy)**

5.3.8 Cu(hfac)$_2$ hydrate in PS film

Owing to optical scattering, the MCD spectra at a fixed temperature contain significant temperature-independent artifacts. The temperature-subtracted MCD spectra provide the most reliable picture of the molecular MCD spectrum.

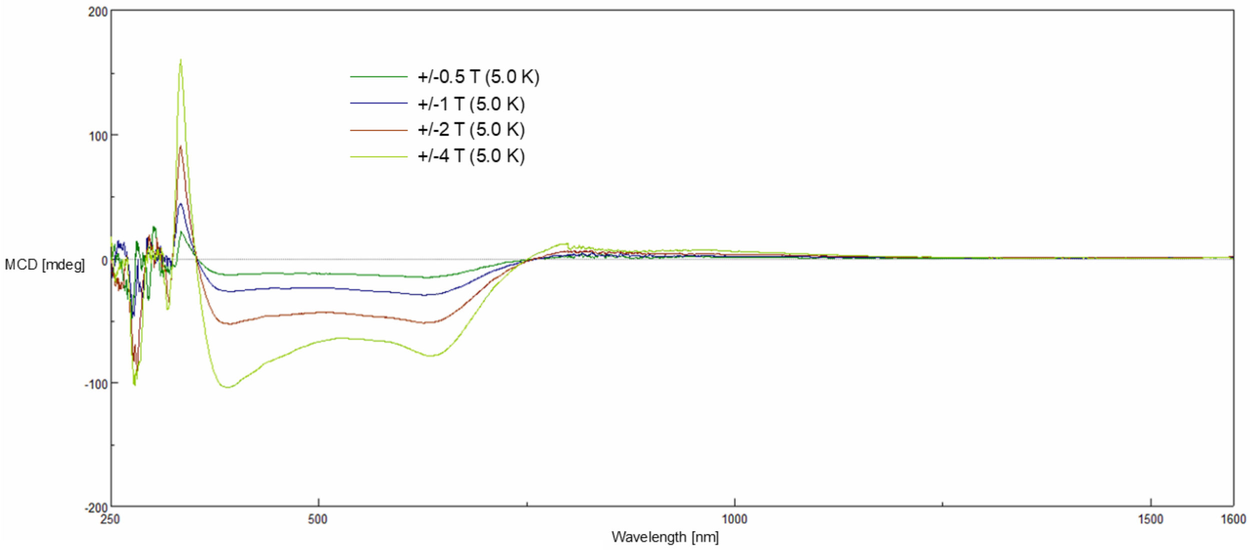

Figure S55: MCD spectra of Cu(hfac)$_2$ hydrate in PS film at 5.0 K.

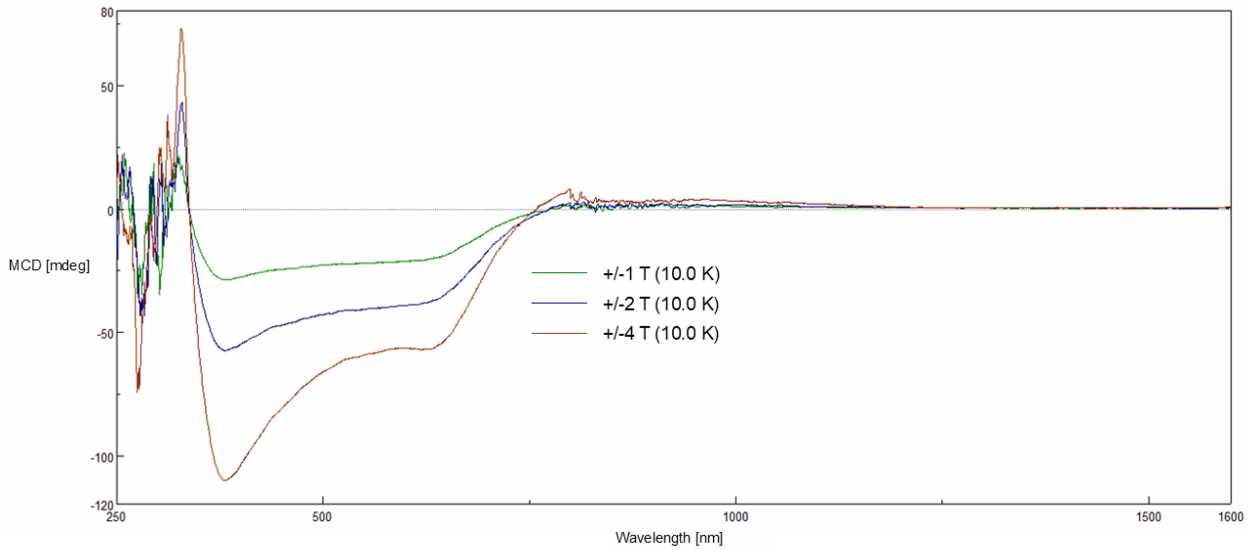

Figure S56: MCD spectra of Cu(hfac)$_2$ hydrate in PS film at 10.0 K.

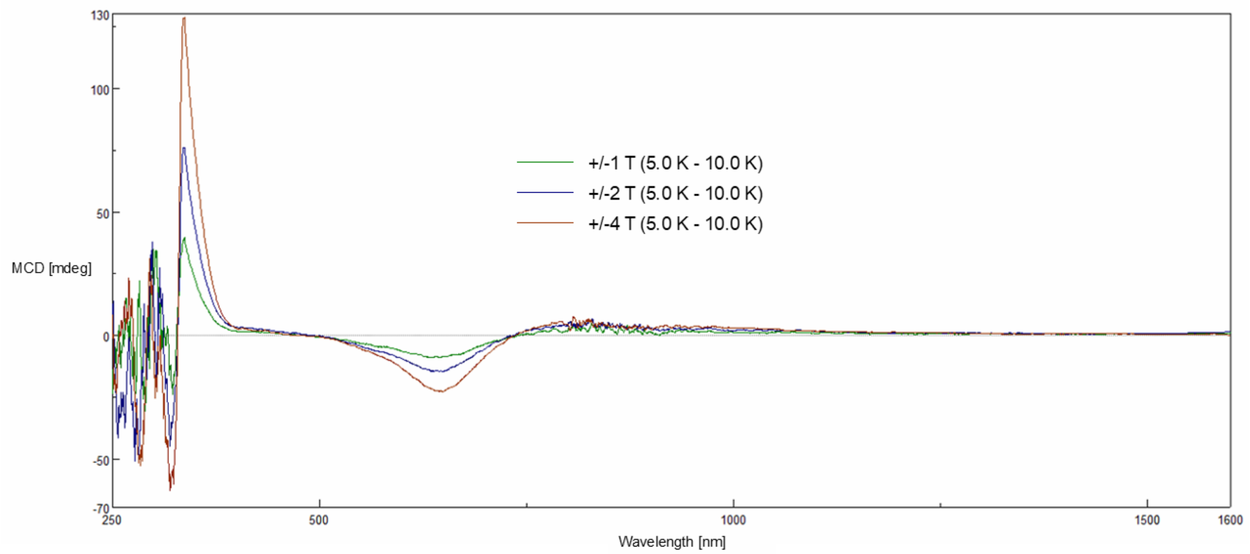

Figure S57: Pure C-term MCD spectra of $Cu(hfac)_2$ in PS film obtained by subtracting 5.0 K spectra from 10.0 K spectra.

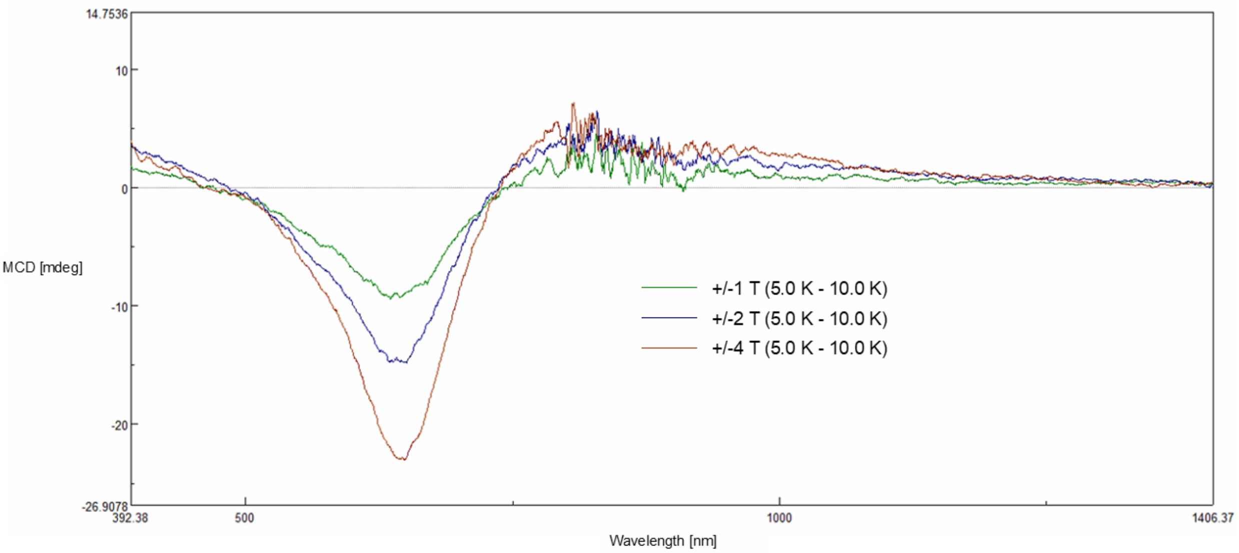

Figure S58: Pure C-term MCD spectra of $Cu(hfac)_2$ in PS film obtained by subtracting 5.0 K spectra from 10.0 K spectra (zoomed axes).

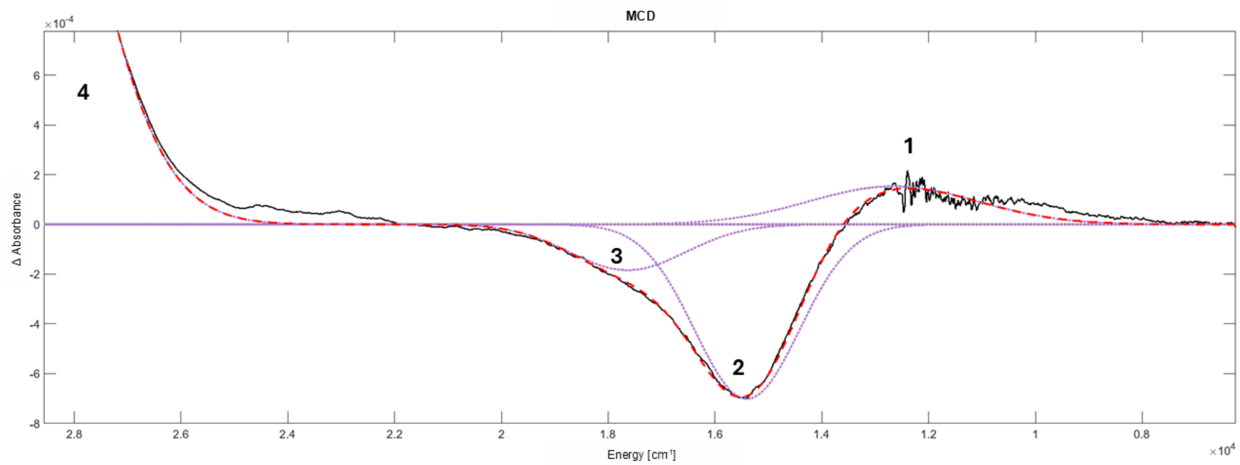

Figure S59: Gaussian band fits to Cu(hfac)$_2$ in PS film. MCD is the pure C-term spectrum obtained at +/-4 T by subtracting the 5.0 K spectrum from the 10.0 K spectrum.

Table S15: MCD fit parameters for Cu(hfac)$_2$ in PS film (see above). Assigned ligand field transitions given in bold.

Peak number	Center (cm^{-1})	FWHM (cm^{-1})	MCD height (ΔAbs)	Assignment
**1**	**12664**	**3785**	**1.53E-04**	**d-d**
**2**	**15401**	**2355**	**-7.01E-04**	**d-d (x^2–y^2 → xy)**
**3**	**17638**	**2615**	**-1.84E-04**	**d-d**
4	29710	3753	2.87E-03	

5.3.9 $K_2[Cu(ox)_2]$ in PVA film

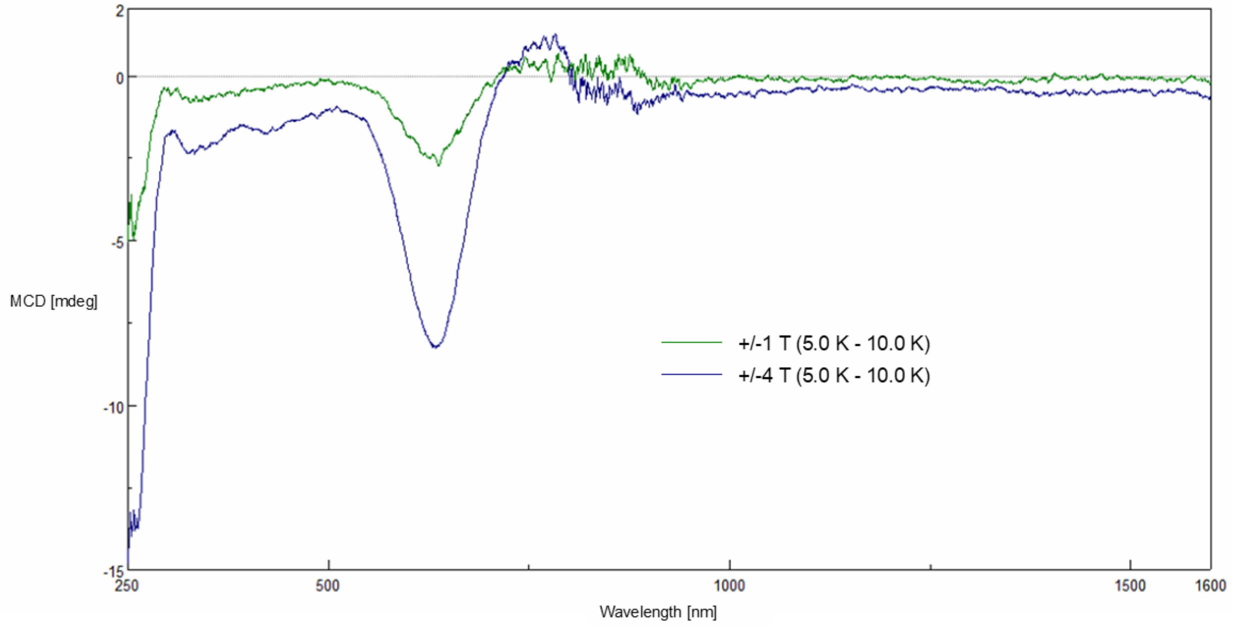

Figure S60: Pure C-term MCD spectra of $K_2[Cu(ox)_2]$ in PVA film obtained by subtracting 5.0 K spectra from 10.0 K spectra.

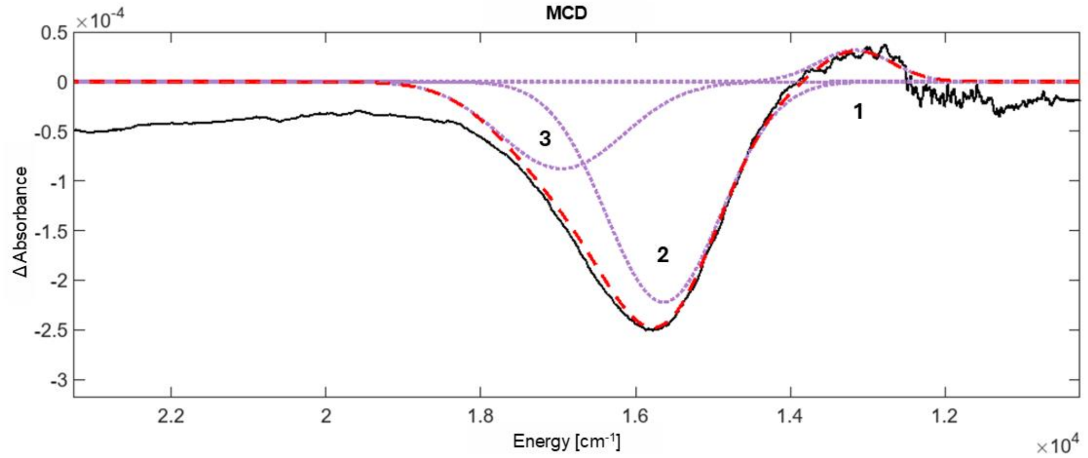

Figure S61: Gaussian band fits to $K_2[Cu(ox)_2]$ in PVA film. MCD is the pure C-term spectrum obtained at +/-4 T by subtracting the 5.0 K spectrum from the 10.0 K spectrum.

Table S16: MCD fit parameters for $K_2[Cu(ox)_2]$ in PVA film (see above). Assigned ligand field transitions given in bold.

Peak number	Center (cm^{-1})	FWHM (cm^{-1})	MCD height (ΔAbs)	Assignment
**1**	**13166**	**1142**	**3.18E-05**	**d-d**
**2**	**15639**	**1766**	**-2.2E-04**	**d-d (x^2–y^2 → xy)**
**3**	**16953**	**1917**	**-8.76E-05**	**d-d**

5.4 Frozen Solution MCD

5.4.1 $(PPh_4)_2[Cu(bdt)_2]$ in 1:1 butyronitrile:DCM frozen solution

Owing to solubility issues, the spectrum of $(PPh_4)_2[Cu(bdt)_2]$ was by far the weakest of those obtained in this study. However, comparison to the spectrum for $(PPh_4)_2[Cu(mnt)_2]$ reveals a similar spectral profile for the two compounds (**Figure S64**). This enabled identification and extraction of the analogous ligand field transitions for $(PPh_4)_2[Cu(bdt)_2]$ despite the low signal strength.

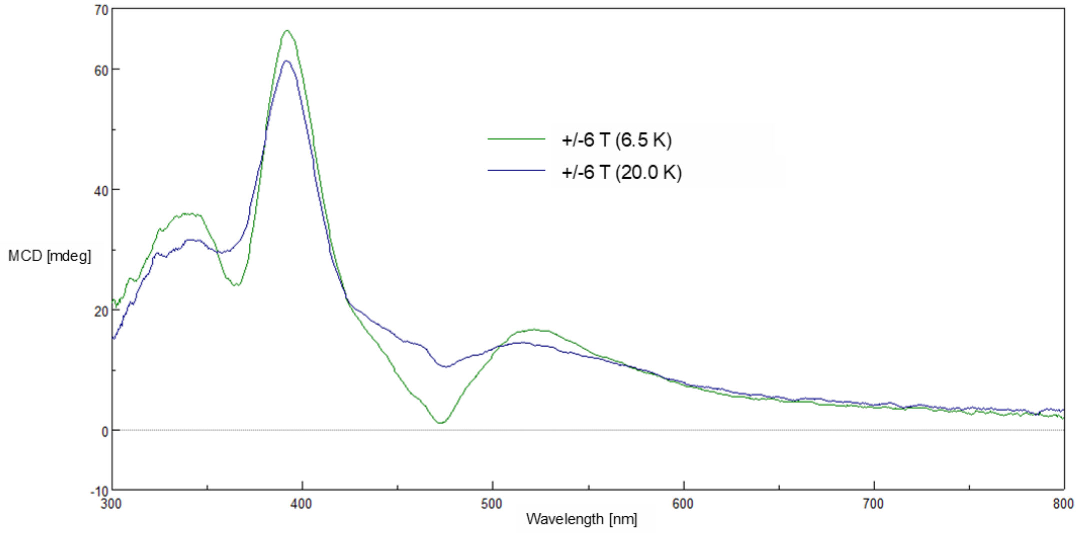

Figure S62: MCD spectra of $(PPh_4)_2[Cu(bdt)_2]$ in 1:1 butyronitrile:DCM glass at 6.5 K and 20.0 K.

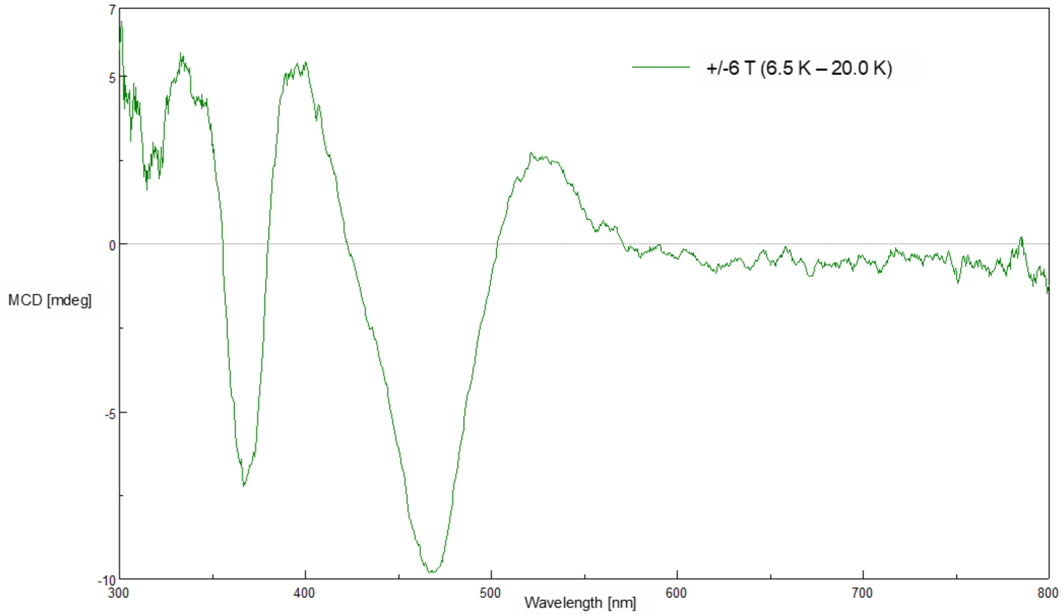

Figure S63: Pure C-term MCD spectra of $(PPh_4)_2[Cu(bdt)_2]$ in 1:1 butyronitrile:DCM glass obtained by subtracting 6.5 K spectra from 20.0 K spectra.

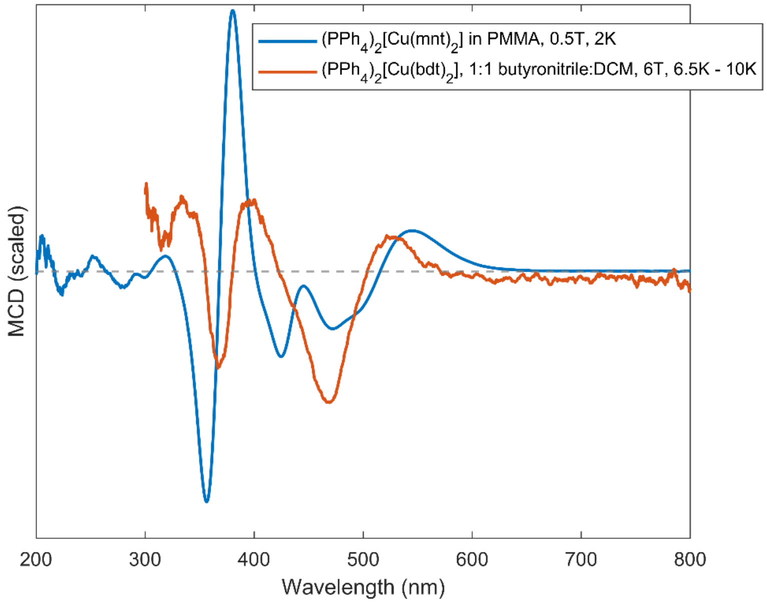

Figure S64: Comparison of scaled MCD spectra for $(PPh_4)_2[Cu(mnt)_2]$ in PMMA and $(PPh_4)_2[Cu(bdt)_2]$ in 1:1 butyronitrile:DCM glass.

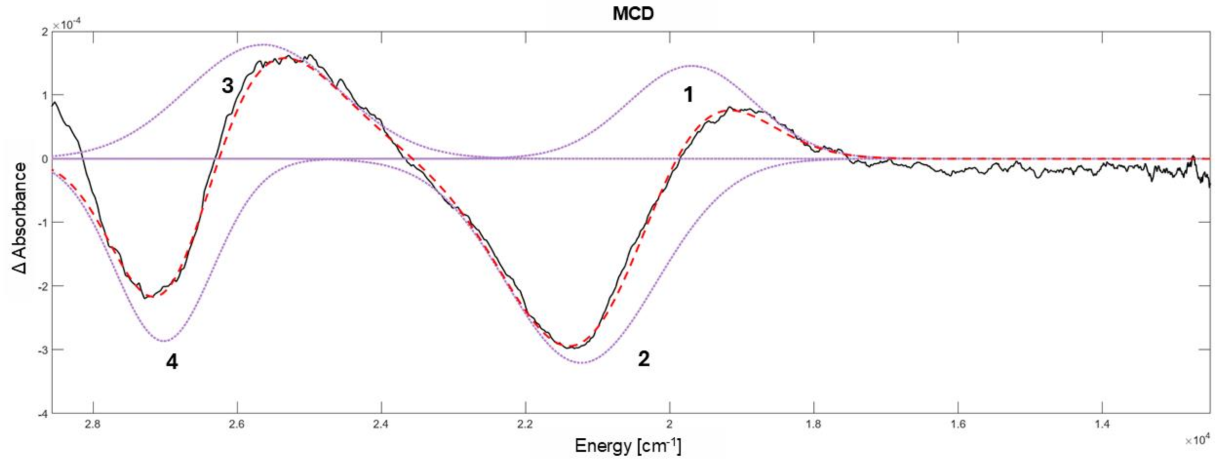

Figure S65: Gaussian band fits to $(PPh_4)_2[Cu(bdt)_2]$ in 1:1 butyronitrile:DCM frozen solution. MCD is the pure C-term spectrum obtained at +/-6 T by subtracting the 6.5 K spectrum from the 20.0 K spectrum.

Table S17: MCD fit parameters for $(PPh_4)_2[Cu(bdt)_2]$ in 1:1 butyronitrile:DCM frozen solution (see above). Assigned ligand field transitions given in bold.

Peak number	Center (cm^{-1})	FWHM (cm^{-1})	MCD height (ΔAbs)	Assignment
**1**	**19698**	**2099**	**1.46E-04**	**d-d**
**2**	**21228**	**2502**	**-3.20E-04**	**d-d (x^2–y^2 → xy)**
3	25643	2528	1.79E-04	
4	27017	1630	-2.86E-04	

5.4.2 $Cu(acac)_2$ in 1:1 DCM:toluene frozen solution

Only one temperature point was acquired for this sample. The baseline appears to have good fidelity to the 0 mdeg line, suggesting a minimum of temperature-independent effects.

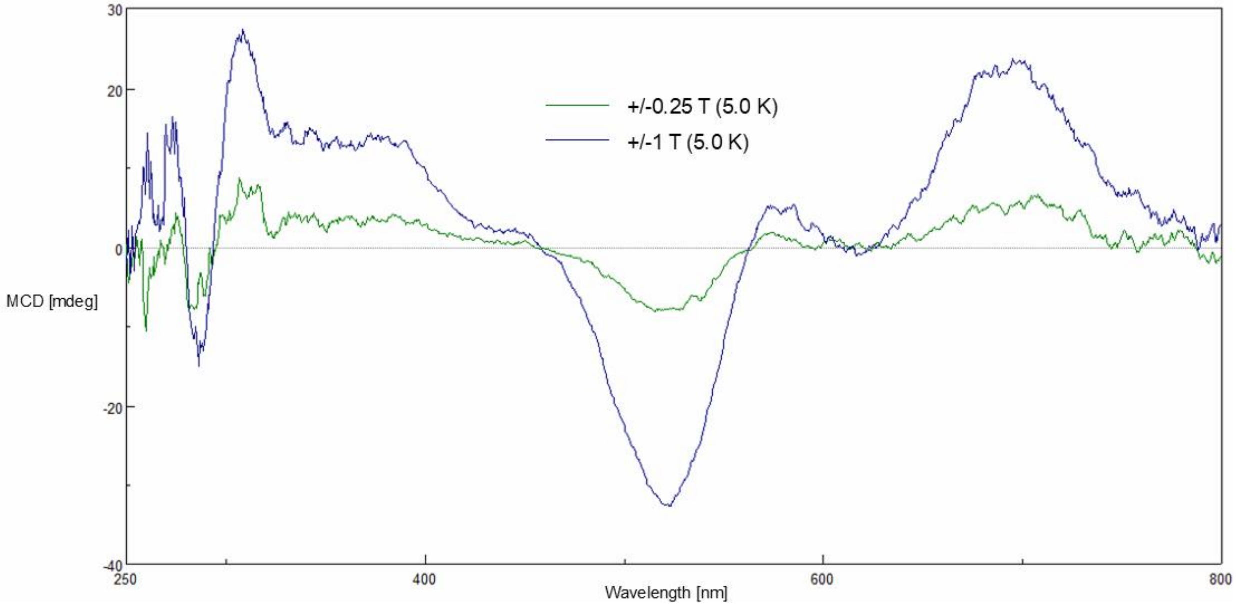

Figure S66: MCD spectra of $Cu(acac)_2$ in 1:1 DCM:toluene frozen solution at 5.0 K.

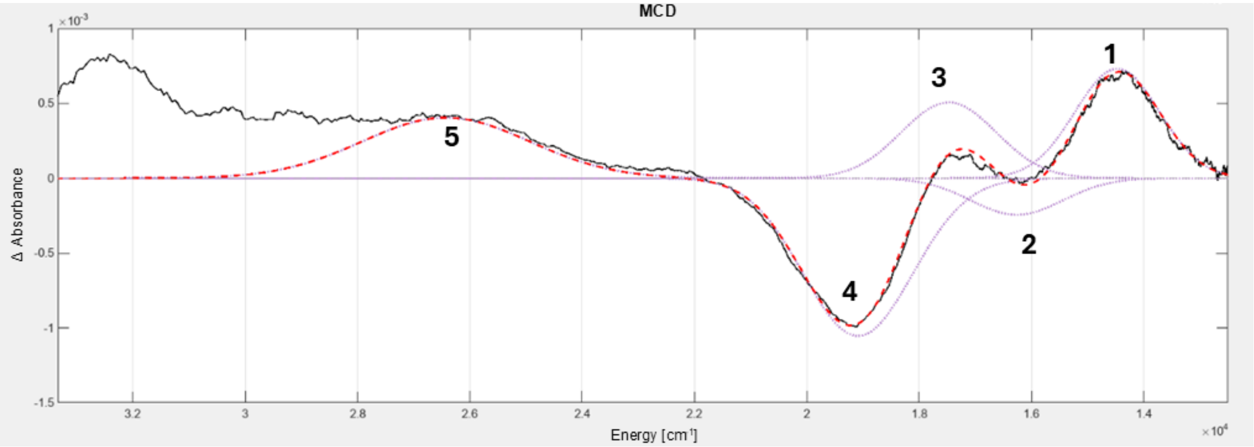

Figure S67: Gaussian band fits to $Cu(acac)_2$ in 1:1 DCM:toluene frozen solution. MCD is obtained at 5.0 K and +/-1 T.

Table S18: MCD fit parameters for $Cu(acac)_2$ in 1:1 DCM:toluene frozen solution (see above). Assigned ligand field transitions given in bold.

Peak number	Center (cm^{-1})	FWHM (cm^{-1})	MCD height (ΔAbs)	Assignment
**1**	**14472**	**1808**	**7.3E-04**	**d-d**
**2**	**16260**	**1970**	**-2.4E-04**	**d-d**
**3**	**17474**	**2027**	**5.1E-04**	**d-d**
**4**	**19088**	**2342**	**-1.0E-03**	**d-d (x^2–y^2 → xy)**
5	26424	3588	4.0E-04	

5.5 Fluorolube Mull MCD

5.5.1 $(PPh_4)_2[Cu(mnt)_2]$ Fluorolube mull

For this sample only, the impact of a sloping baseline due to scattering was removed by a regularized polynomial fit. The resulting spectrum is in agreement with the polymer film spectrum, displaying small shifts in peak position.

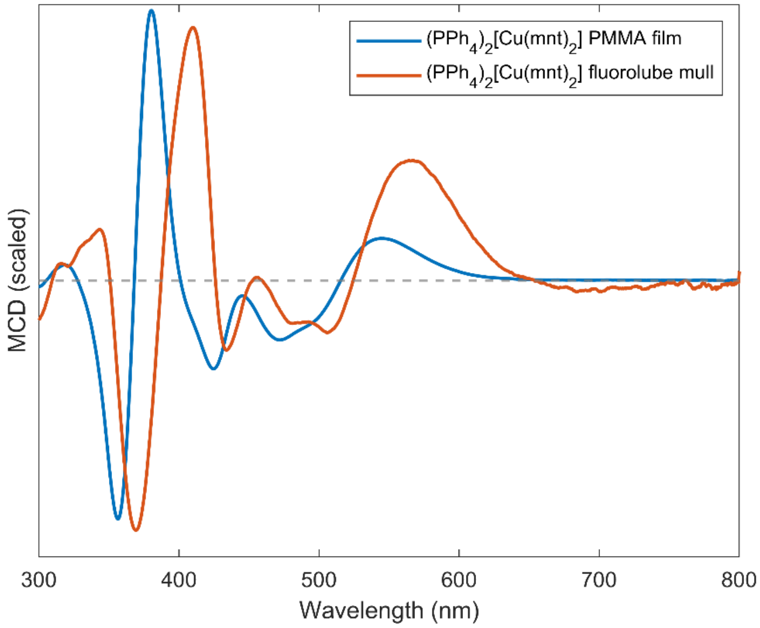

Figure S68: Comparison of scaled MCD spectra for $(PPh_4)_2[Cu(mnt)_2]$ in PMMA and $(PPh_4)_2[Cu(mnt)_2]$ in Fluorolube mull. Spectrum in PMMA acquired at 2.0 K and +/-0.5 T, while spectrum in Fluorolube mull acquired at 5.0 K and +/-7 T (polynomial baseline subtracted).

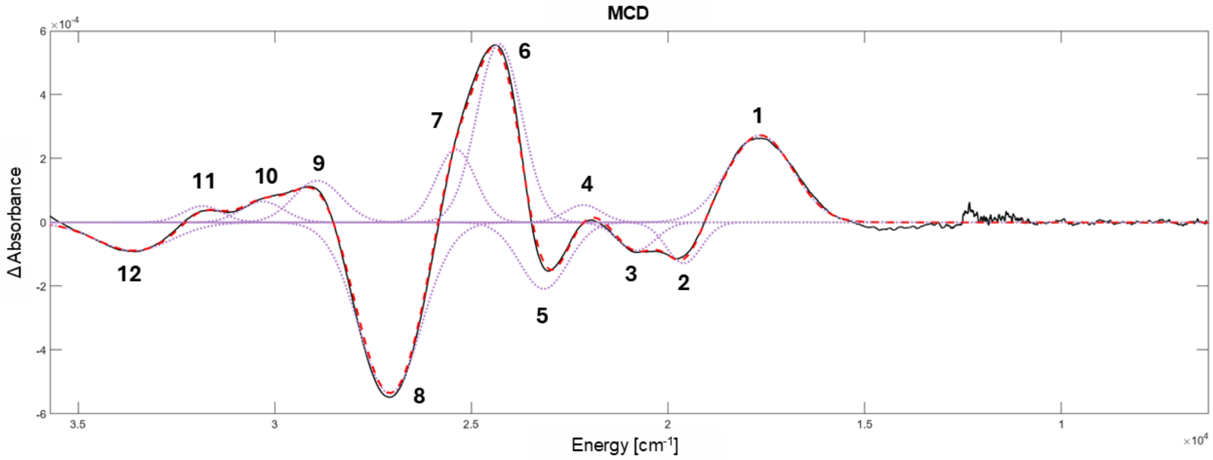

Figure S69: Gaussian band fits to $(PPh_4)_2[Cu(mnt)_2]$ in Fluorolube mull. MCD acquired at 5.0 K and +/-7 T (polynomial baseline subtracted).

Table S19: MCD fit parameters for $(PPh_4)_2[Cu(mnt)_2]$ in Fluorolube mull (see above). Assigned ligand field transitions given in bold.

Peak number	Center (cm^{-1})	FWHM (cm^{-1})	MCD height (ΔAbs)	Assignment
**1**	**17655**	**1994**	**2.73E-04**	**d-d**
**2**	**19599**	**1064**	**-1.28E-04**	**d-d**
**3**	**20796**	**1151**	**-8.80E-05**	**d-d**
4	22153	1111	5.42E-05	
5	23156	1477	-2.09E-04	
6	24291	1375	5.59E-04	
7	25396	1199	2.29E-04	
8	27070	1919	-5.37E-04	
9	28913	1468	1.31E-04	
10	30314	1310	6.55E-05	
11	31835	1190	5.12E-05	
12	33581	2319	-8.86E-05	

5.5.2 Cu(dtc)$_2$ in Fluorolube mull

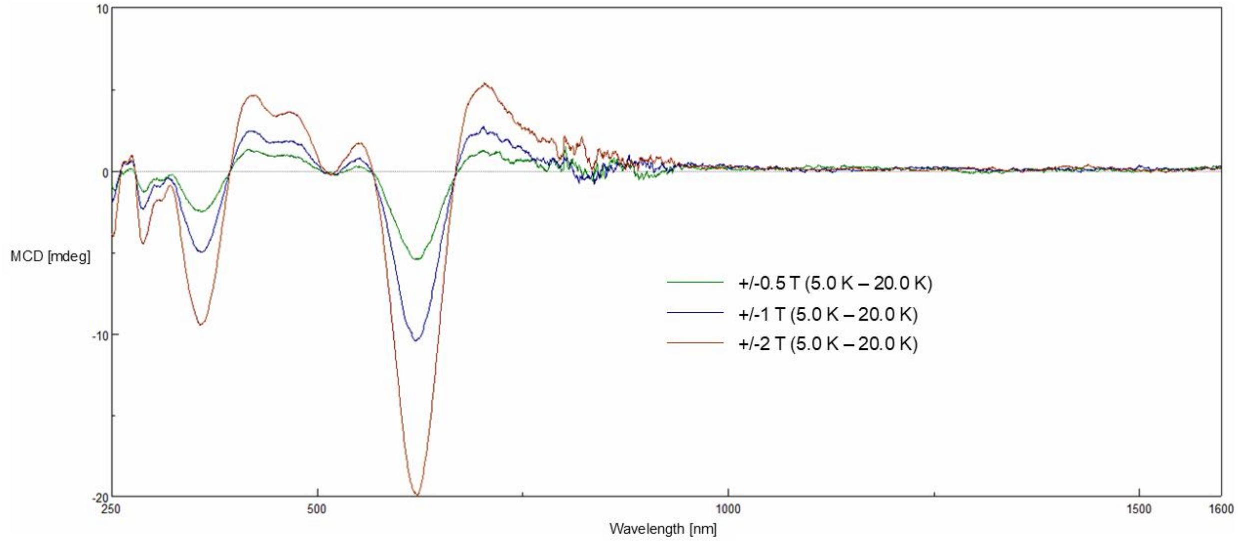

Figure S70: Pure C-term MCD spectra of Cu(dtc)$_2$ in Fluorolube mull obtained by subtracting 5.0 K spectra from 20.0 K spectra.

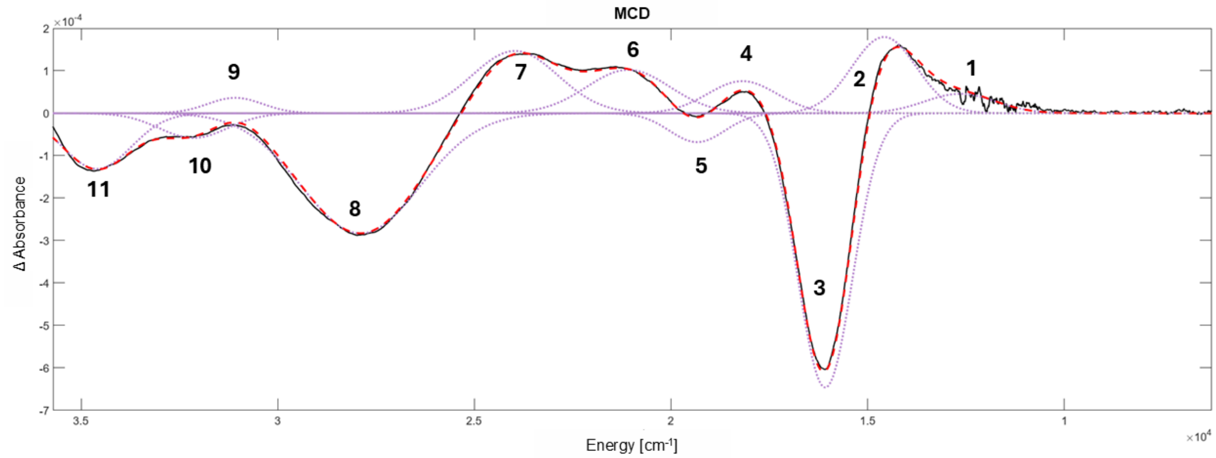

Figure S71: Gaussian band fits to Cu(dtc)$_2$ in Fluorolube mull. MCD is the pure C-term spectrum obtained at +/-2 T by subtracting the 5.0 K spectrum from the 20.0 K spectrum.

Table S20: MCD fit parameters for $Cu(dtc)_2$ in Fluorolube mull (see above). Assigned ligand field transitions given in bold.

Peak number	Center (cm^{-1})	FWHM (cm^{-1})	MCD height (ΔAbs)	Assignment
**1**	**12646**	**2166**	**4.58E-05**	**d-d / bandshape?**
**2**	**14581**	**1975**	**1.80E-04**	**d-d**
**3**	**16077**	**1657**	**-6.46E-04**	**d-d (x^2–y^2 → xy)**
4	18185	2116	7.56E-05	
5	19335	1687	-6.80E-05	
6	21084	2545	1.02E-04	
7	23966	2628	1.47E-04	
8	27892	3632	-2.83E-04	
9	31081	1667	3.63E-05	
10	32145	2084	-5.81E-05	
11	34597	2089	-1.31E-04	

5.5.3 Cu(tmhd)$_2$ in Fluorolube mull

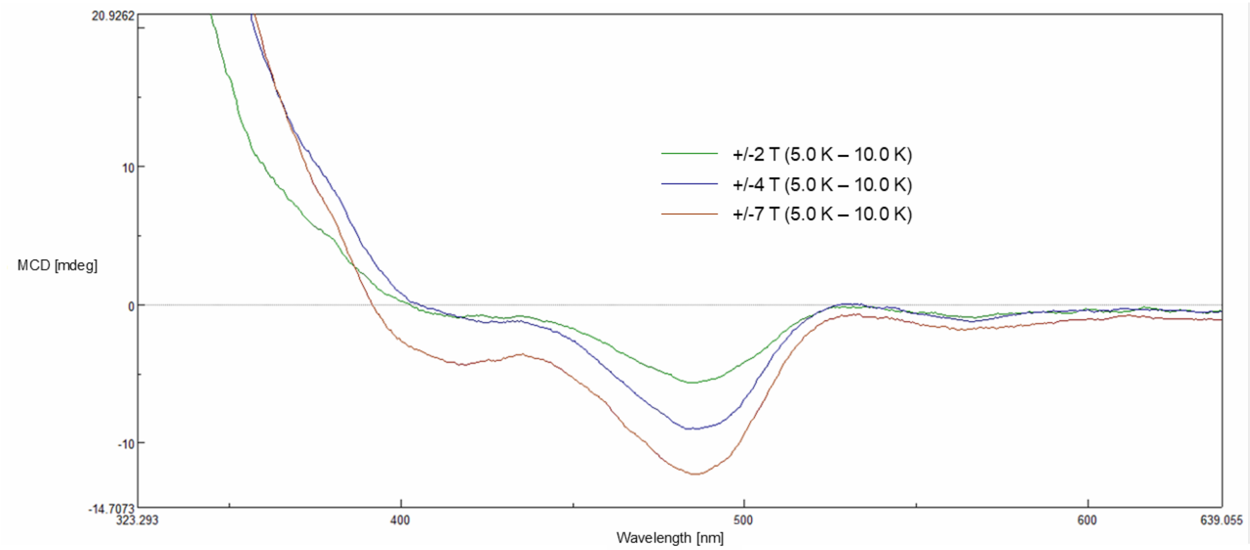

Figure S72: Pure C-term MCD spectra of Cu(tmhd)$_2$ in Fluorolube mull obtained by subtracting 5.0 K spectra from 10.0 K spectra.

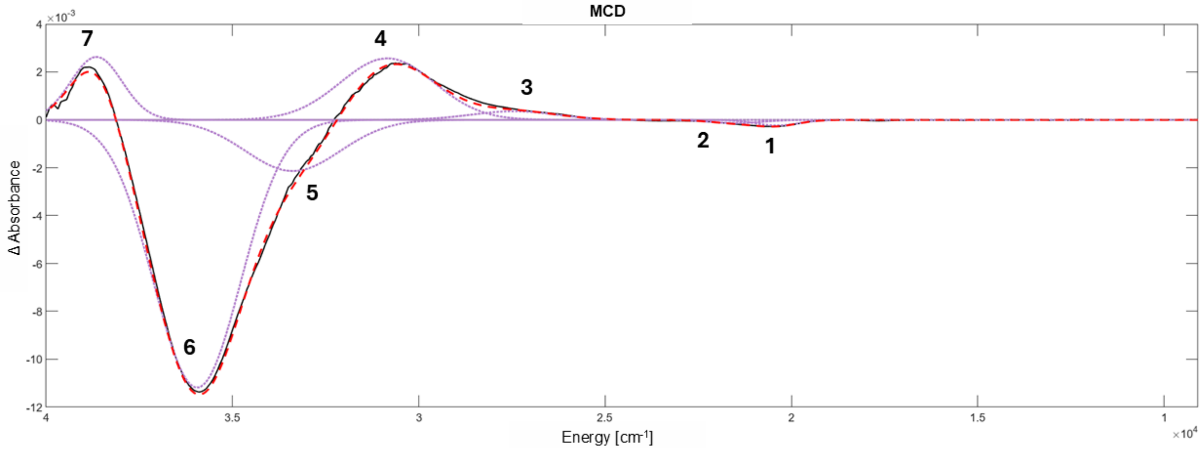

Figure S73: Gaussian band fits to Cu(tmhd)$_2$ in Fluorolube mull. MCD is the pure C-term spectrum obtained at +/-4 T by subtracting the 5.0 K spectrum from the 10.0 K spectrum.

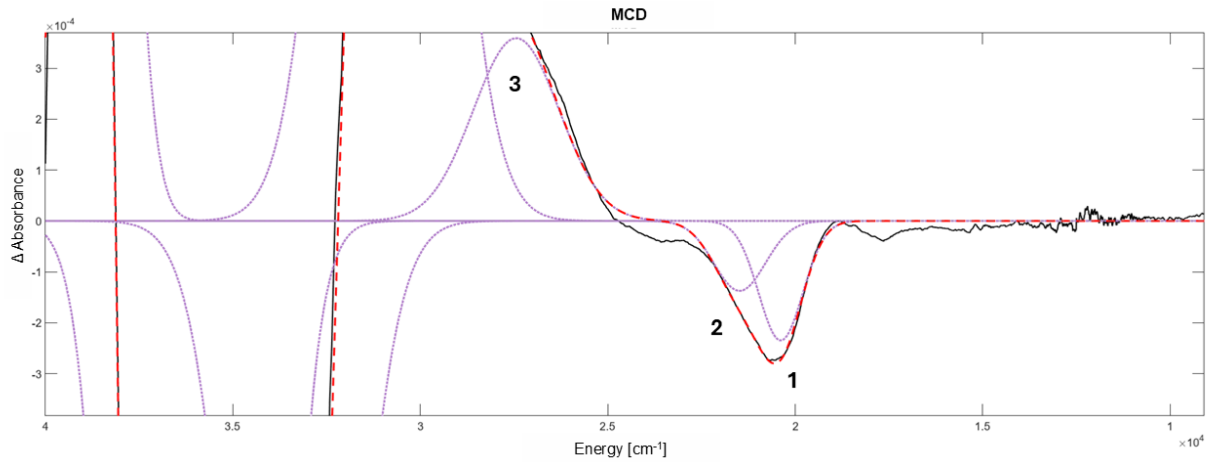

Figure S74: Gaussian band fits to Cu(tmhd)$_2$ in Fluorolube mull (zoomed in). MCD is the pure C-term spectrum obtained at +/-4 T by subtracting the 5.0 K spectrum from the 10.0 K spectrum.

Table S21: MCD fit parameters for Cu(tmhd)$_2$ in Fluorolube mull (see above). Assigned ligand field transitions given in bold.

Peak number	Center (cm^{-1})	FWHM (cm^{-1})	MCD height (ΔAbs)	Assignment
**1**	**20388**	**1409**	**-2.35E-04**	**d-d (x^2–y^2 → xy)**
**2**	**21484**	**1647**	**-1.37E-04**	**d-d**
3	27442	2752	3.59E-04	
4	30847	3027	2.57E-03	
5	33379	3066	-2.14E-03	
6	35950	2807	-1.12E-02	
7	38650	1657	2.63E-03	

5.5.4 Cu(acac)$_2$ in Fluorolube mull

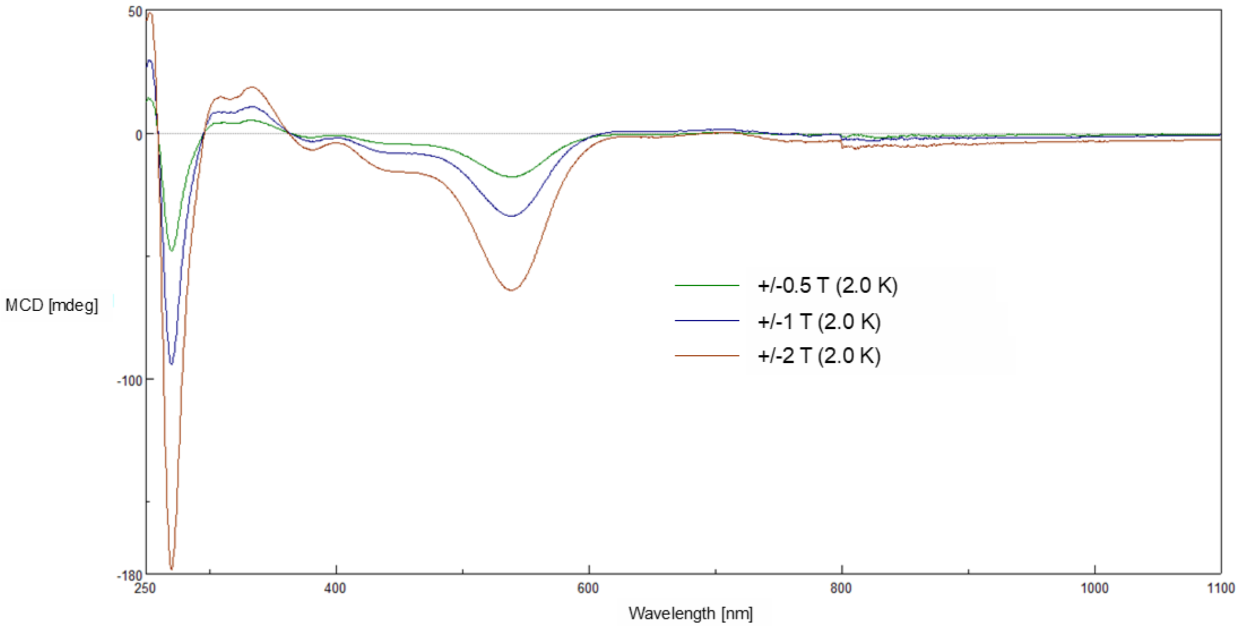

Figure S75: MCD spectra of Cu(acac)$_2$ in Fluorolube mull at 2.0 K.

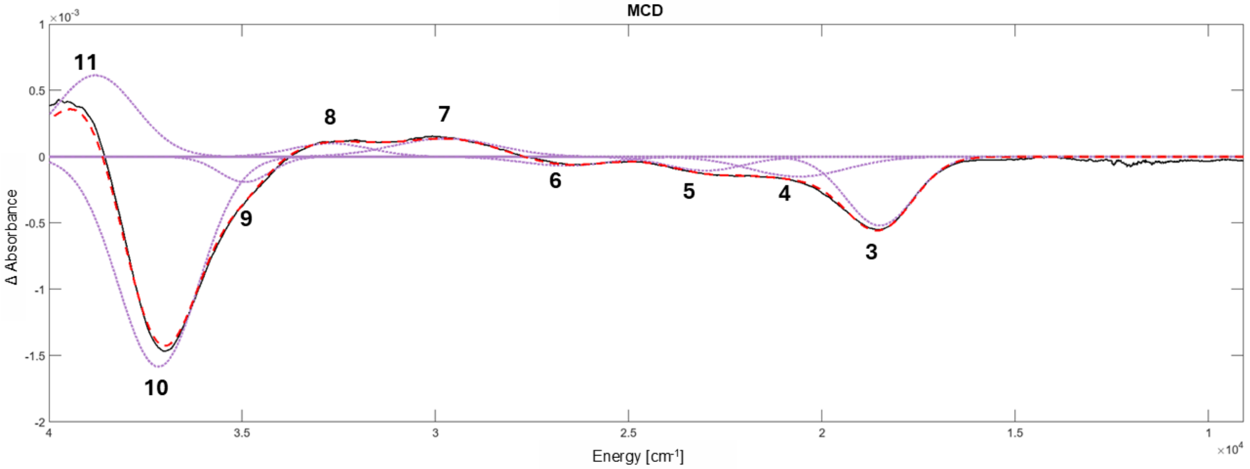

Figure S76: Gaussian band fits to Cu(acac)$_2$ in Fluorolube mull. MCD obtained at +/-0.5 T and 2.0 K.

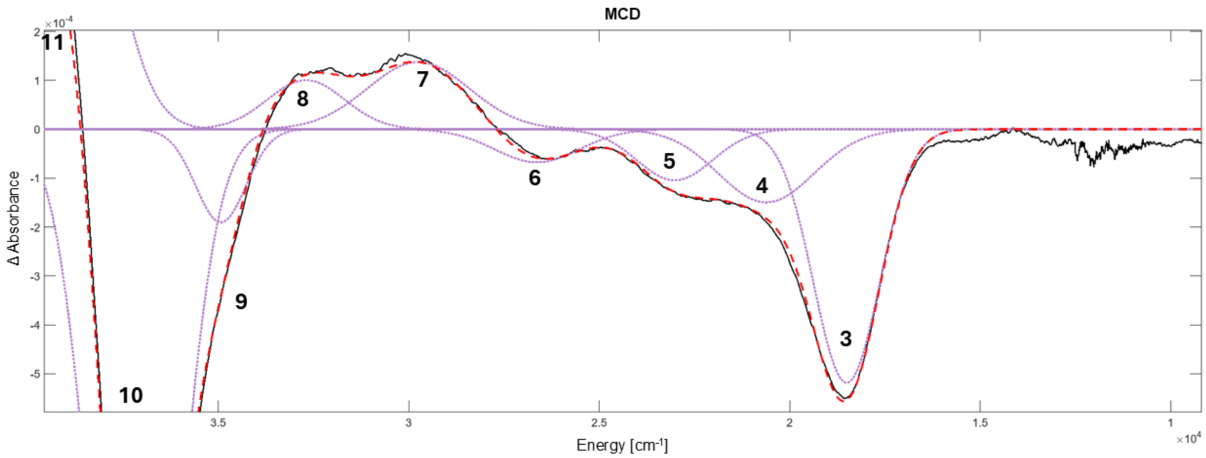

Figure S77: Gaussian band fits to $Cu(acac)_2$ in Fluorolube mull (zoomed in). MCD obtained at +/-0.5 T and 2.0 K.

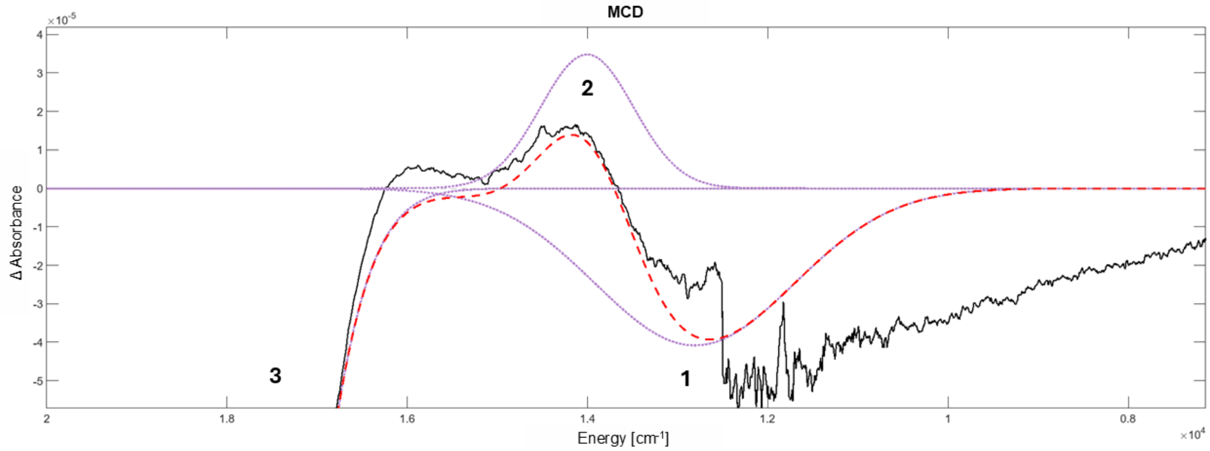

Figure S78: Gaussian band fits to $Cu(acac)_2$ in Fluorolube mull (zoomed in further). MCD obtained at +/-0.5 T and 1.8 K.

Table S22: MCD fit parameters for $Cu(acac)_2$ in Fluorolube mull (see above). Assigned ligand field transitions given in bold.

Peak number	Center (cm^{-1})	FWHM (cm^{-1})	MCD height (ΔAbs)	Assignment
1	12811	2653	-4.08E-05	baseline scatter
**2**	**14006**	**1239**	**3.48E-05**	**d-d**
**3**	**18589**	**2054**	**-5.58E-04**	**d-d (x^2–y^2 → xy)**
4	20617	3009	-1.50E-04	
5	23020	2382	-1.05E-04	
6	26635	2675	-6.76E-05	
7	29749	3218	1.37E-04	
8	32696	2424	1.00E-04	
9	34932	1588	-1.91E-04	
10	37168	2508	-1.58E-03	
11	38798	2498	6.14E-04	

5.5.5 $Cu(hfac)_2$ hydrate in Fluorolube mull

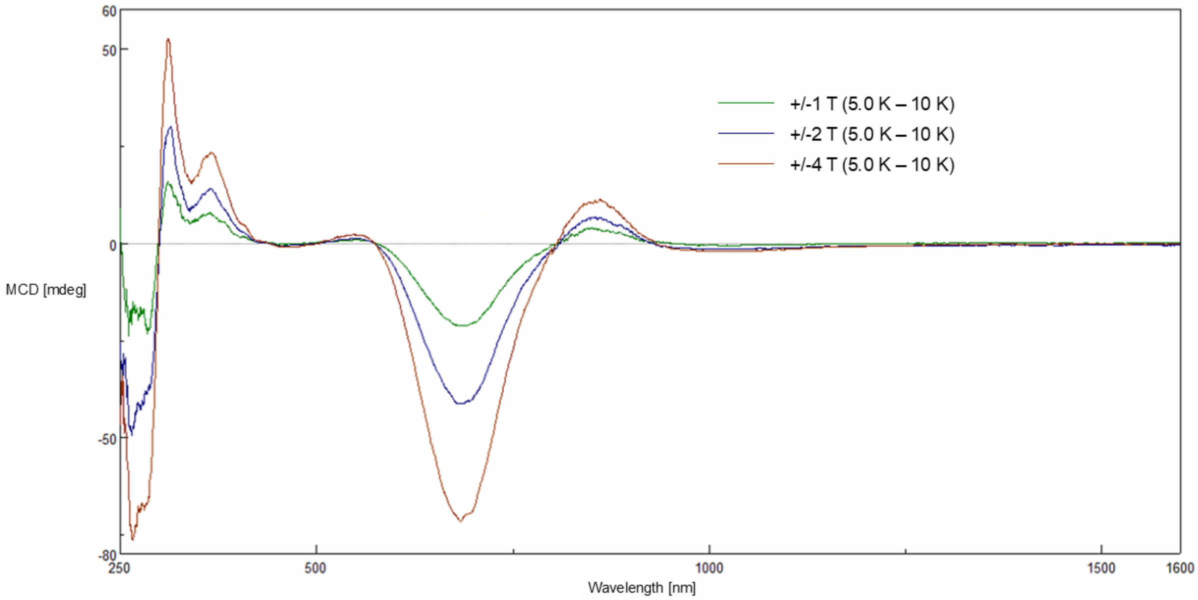

Figure S79: Pure C-term MCD spectra of $Cu(hfac)_2$ hydrate in Fluorolube mull obtained by subtracting 5.0 K spectra from 10.0 K spectra.

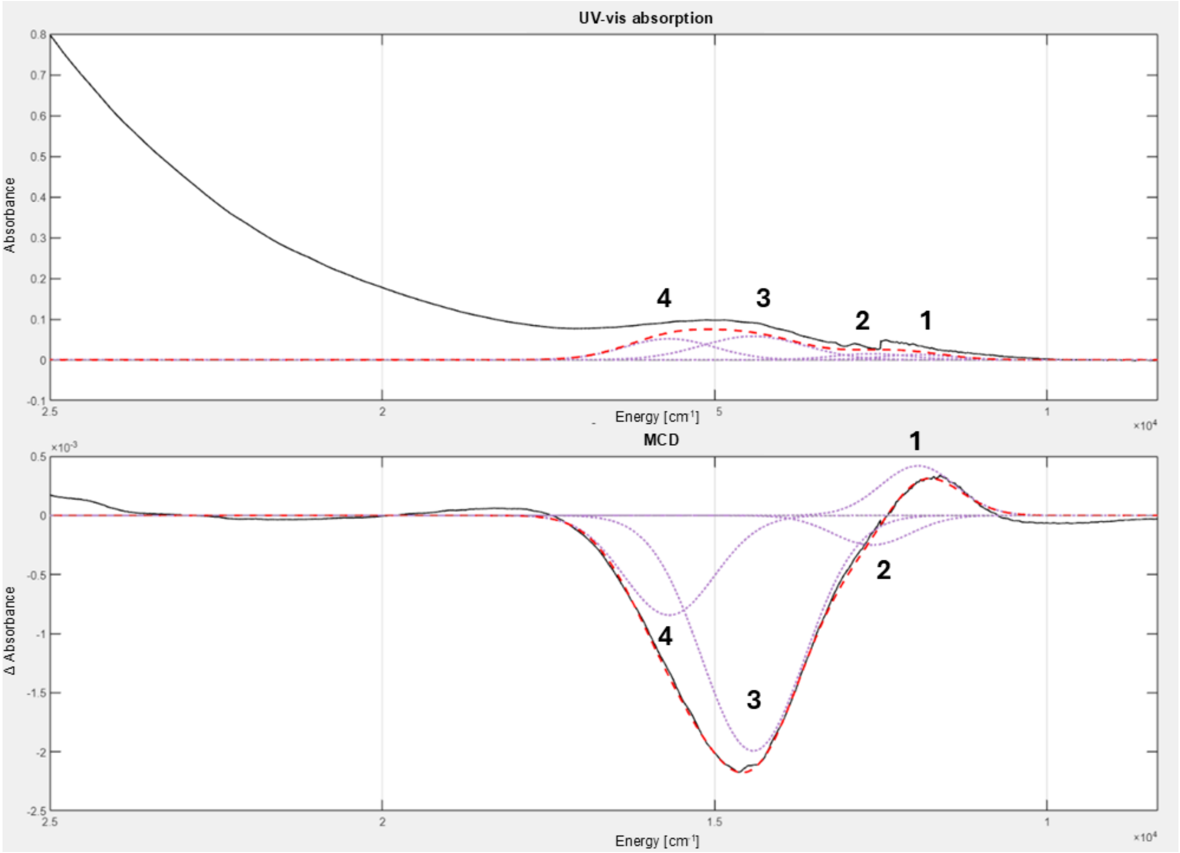

Figure S80: Gaussian band fits to $Cu(hfac)_2$ hydrate in Fluorolube mull. MCD is the pure C-term spectrum obtained at +/-4 T by subtracting the 5.0 K spectrum from the 10.0 K spectrum.

Table S23: MCD fit parameters for $Cu(hfac)_2$ hydrate in Fluorolube mull. Assigned ligand field transitions given in bold.

Peak number	Center (cm^{-1})	FWHM (cm^{-1})	Absorption height (Abs)	MCD height (ΔAbs)	C/D @ 5K – 10K, 4T	Assignment
**1**	**11941**	**1352**	**0.013**	**4.20E-04**	**0.144**	**d-d**
**2**	**12639**	**1399**	**0.015**	**-2.50E-04**	**-0.073**	**d-d**
**3**	**14419**	**1858**	**0.058**	**-1.99E-03**	**-0.150**	**d-d (x^2–y^2 → xy)**
**4**	**15683**	**1632**	**0.052**	**-8.44E-04**	**-0.071**	**d-d**

5.5.6 $K_2[Cu(ox)_2]$ hydrate in Fluorolube mull

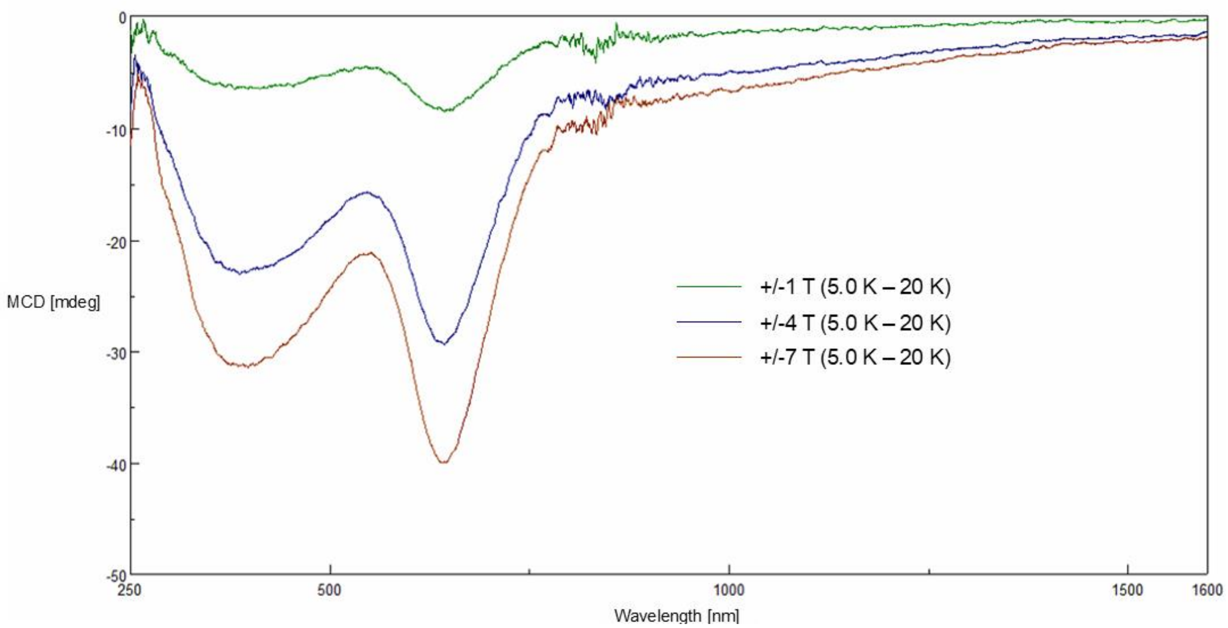

Figure S81: Pure C-term MCD spectra of $K_2[Cu(ox)_2]$ hydrate in Fluorolube mull obtained by subtracting 5.0 K spectra from 20.0 K spectra.

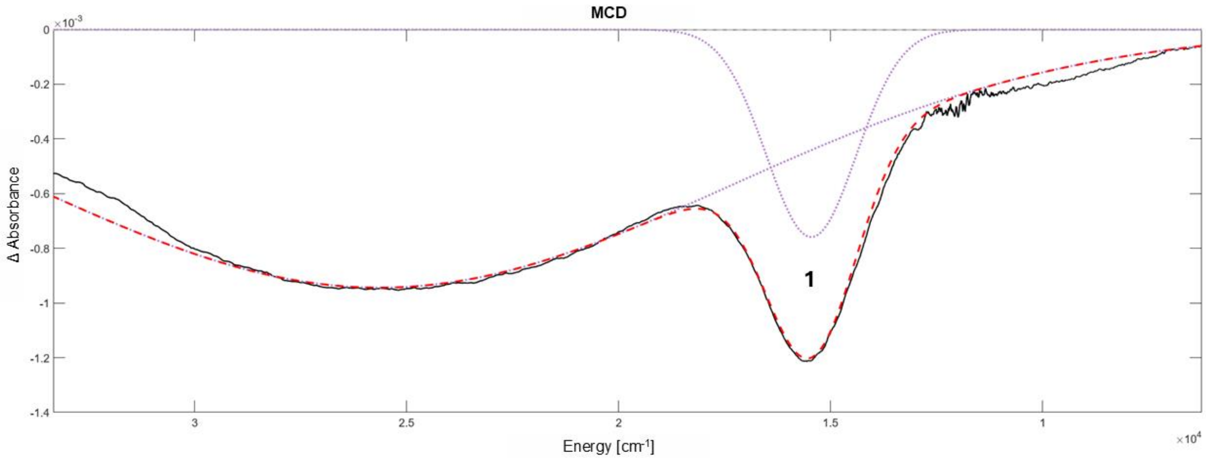

Figure S82: Gaussian band fits to $K_2[Cu(ox)_2]$ hydrate in Fluorolube mull. MCD is the pure C-term spectrum obtained at +/-7 T by subtracting the 5.0 K spectrum from the 20.0 K spectrum.

Table S24: MCD fit parameters for $K_2[Cu(ox)_2]$ hydrate in Fluorolube mull (see above). Assigned ligand field transitions given in bold.

Peak number	Center (cm^{-1})	FWHM (cm^{-1})	MCD height (ΔAbs)	Assignment
**1**	**15457**	**2545**	**-7.59E-04**	**d-d (x^2–y^2 → xy)**

6. EPR Methods

CW X-band spectra were obtained on a Bruker EMX spectrometer using a liquid nitrogen immersion dewar. X-band CW spectra were fit in EasySpin 5.2.35 to extract the spin-Hamiltonian parameters.[13]All spectra were fit with the pepper function. Where possible, spectra were fit to an axial spin Hamiltonian for simplicity of interpretation, though some fits could be improved by allowing minor rhombic deviations. While all other compounds could be fit well to a single spin system, $Cu(tmhd)_2$ and $Cu(hfac)_2$ were fit to two spin systems, likely due to a mixture of axial coordination states from water or the polymer matrix. Fit results are tabulated in **Table S25**, and spectra with associated fits are given **SI Section 6.1**.

Pulse X-band EPR experiments were conducted with a Bruker ELEXSYS E580 pulse EPR spectrometer, using a combination of Bruker MS-5 and MD-4 resonators. Temperature control was achieved using an Oxford Instruments CF935 cryogen flow cryostat using liquid helium (5−100 K) or liquid nitrogen (>100 K) and a Mercury iTC temperature controller. Echo-detected field sweep spectra employed a two-pulse Haho-echo sequence (π/2−τ−π−τ−echo). Inversion recovery experiments employed the pulse sequence π−t−π/2−τ−π−τ−echo, where t is the variable time delay and τ is a fixed constant. The fixed delay τ was optimized independently for each sample to maximize the echo intensity. The values of t (inversion recovery) and 2τ (Hahn-echo decay) include the spectrometer deadtime. The video gain was optimized at each field position and temperature. Four-step phase cycling was used on inversion recovery measurements to eliminate influence of secondary echoes and microwave ringdown, while two-step phase cycling was used on Hahn-echo decay measurements. The shot repetition time was chosen to be at least five times the value of T_1. The π/2 pulses had a duration of 8 ns at X-band, while the π pulses had a duration of 16 ns. Inversion recovery experiments were fit to stretched exponential functions in Matlab R2023b according to Equation S1. Hahn-echo decay experiments to quantify T_m were fit to Equation S2. Error bars were obtained from the 95% confidence intervals on the fitted T_1 or T_m parameters.

$$I = Ae^{[-\left(\frac{t}{T_1}\right)^{\beta}]} + I_0 \tag{S7}$$

$$I = Ae^{[-\left(\frac{2\tau}{T_m}\right)^{\beta}]} + I_0 \tag{S8}$$

EPR samples were prepared to mimic the MCD sample conditions as closely as possible. A complete list of sample preparation choices is given **SI Section 1.3**.

6.1 Spin Hamiltonian Fitting of EPR Spectra

Table S25: Simulated spin Hamiltonian parameters for CW and pulsed EDFS EPR spectra.

All CW spectra were collected at 77 K. All EDFS spectra were collected at 100 K. Rows shaded in grey correspond to literature reported values.

Compound (matrix, acquisition method)	g_x	g_y	g_z	$\|A_x\|$ (MHz)	$\|A_y\|$ (MHz)	$\|A_z\|$ (MHz)	^{14}N $A_\perp$, $A_\parallel$ (MHz)	System Weight
$Cu(dtc)_2$ (PS film, CW)	2.023	2.023	2.090	120	120	490	--	--
$Cu(dtc)_2$ (PS film, EDFS)	2.023	2.023	2.095	110	110	490	--	--
$Cu(dtc)_2$ (2:1 toluene: $CHCl_3$, CW)[14]	2.019	2.019	2.087	40	40	164	--	--
$Cu(pci)_2$ (PS film, CW)	2.048	2.048	2.190	100	100	580	48, 40	--
$Cu(pci)_2$ (PS film, EDFS)	2.037	2.037	2.195	65	65	580	48, 40	--
$Cu(pci)_2$ (2:1 toluene:$CHCl_3$, CW)[4]	2.049	2.049	2.190	10	10	208	--	--
Cu(acacen) (PMMA film, CW)	2.043	2.043	2.188	131	131	618	45, 40	--
Cu(acacen) (PMMA film, EDFS)	2.043	2.043	2.188	70	70	618	45, 40	--
Cu(acacen) (*ortho*-terphenyl, CW)[3]	2.045	2.045	2.18	100	100	650	40, 45	
$Cu(tmhd)_2$ (PMMA film, CW)	2.056	2.049	2.270	38	86	555	--	0.44
	2.049	2.047	2.250	58	85	565	--	0.56
$Cu(tmhd)_2$ (PMMA film, EDFS)	2.059	2.053	2.270	35	80	555	--	0.43
	2.052	2.048	2.255	58	85	570	--	0.57
$Cu(tbaa)_2$ (PS film, CW)	2.052	2.054	2.270	83	52	560	--	--
$Cu(tbaa)_2$ (PS film, EDFS)	2.055	2.058	2.270	83	52	560	--	--

(continued on the next page)

Table S25 continued:

Compound (matrix, acquisition method)	g_x	g_y	g_z	$\|A_x\|$ (MHz)	$\|A_y\|$ (MHz)	$\|A_z\|$ (MHz)	$^{14}N\ A_\perp$, $A_\parallel$ (MHz)	System Weight
$Cu(hfac)_2$ (PS film, CW)	2.068	2.059	2.320	10	10	470	--	0.73
	2.057	2.048	2.285	46	51	530	--	0.27
$Cu(hfac)_2$ (PS film, EDFS)	2.079	2.043	2.325	15	10	470	--	0.75
	2.061	2.047	2.294	48	63	530	--	0.25
$Cu(hfac)_2 \bullet H_2O$ (xylene, CW)[15]	2.069	2.070	2.360	9	9	480	--	--
$Cu(hfac)_2$ (xylene, CW)[15]	2.061	2.063	2.290	51	51	528	--	--
$(PPN)_2[Cu(ox)_2]$ (PS film, CW)	2.048	2.048	2.255	85	85	590	--	--
$(PPN)_2[Cu(ox)_2]$ (PS film, EDFS)	2.052	2.052	2.260	85	85	590	--	--
$K_2[Cu(ox)_2]$ (30%:70% glycerol:water, CW)	2.067	2.067	2.322	14	14	497	--	--
$K_2[Cu(ox)_2] \bullet 2(H_2O)$ (3% diluted crystal in $K_2[Pd(ox)_2] \bullet 2(H_2O)$, CW)[16]	2.060	2.056	2.282	15	20	181	--	--
$(PPh_4)_2[Cu(mnt)_2]$ (PMMA film, EDFS)	2.024	2.024	2.091	100	100	450	--	--
$(PPh_4)_2[Cu(mnt)_2]$ (0.1% diluted crystal in $(PPh_4)_2[Ni(mnt)_2]$, EDFS)[17]	2.023	2.023	2.093	116	116	499	--	--
$(PPh_4)_2[Cu(bdt)_2]$ (2:1 MeCN:toluene, CW)	2.021	2.021	2.089	100	100	470	--	--
$(PPh_4)_2[Cu(bdt)_2]$ (0.5% diluted crystal in $(PPh_4)_2[Ni(bdt)_2]$, CW)[8]	2.019	2.019	2.085	115	115	500	--	--
$Cu(acac)_2$ (3:1 DCM:benzene, EDFS)	2.052	2.054	2.257	84	71	560	--	--
$Cu(acac)_2$ (0.1% diluted crystal in $Pd(acac)_2$, EDFS)[17]	2.055	2.051	2.260	79	72	556	--	--

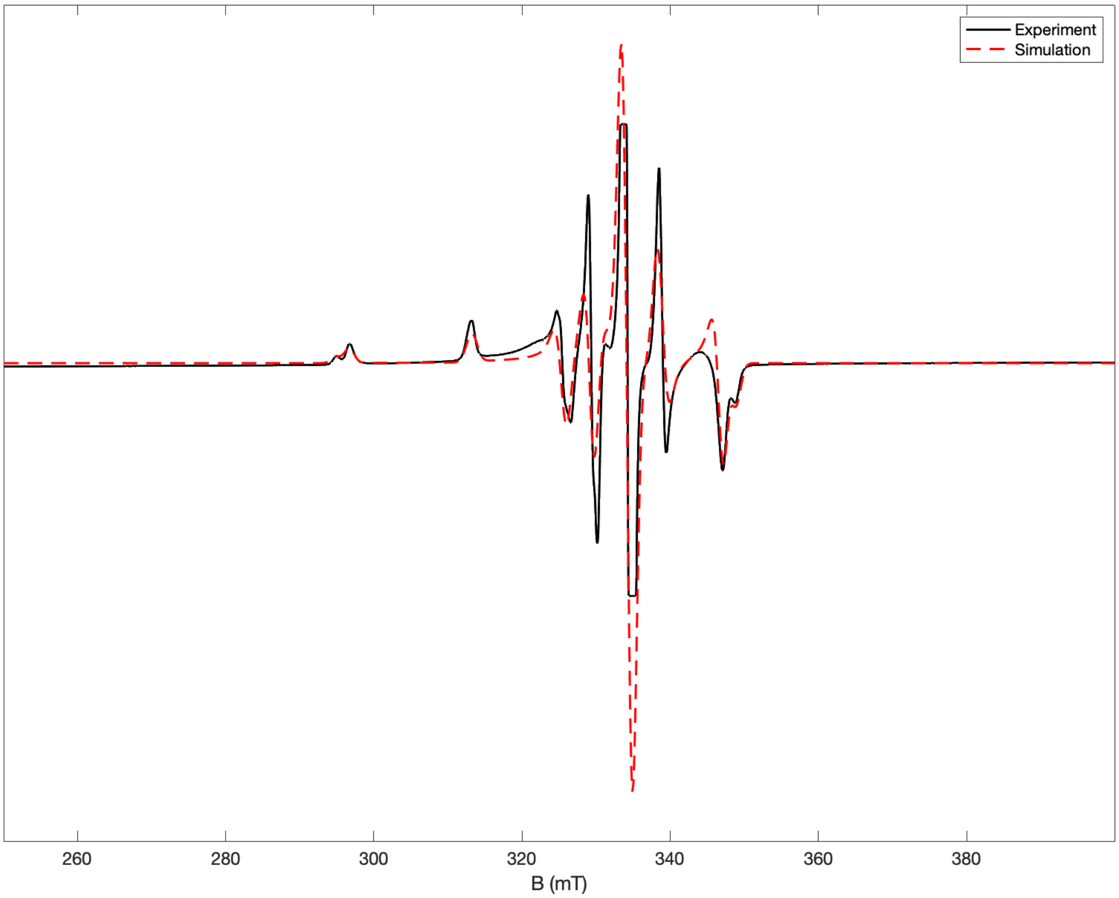

Figure S83: X-band CW EPR spectrum and fit for Cu(dtc)$_2$ in PS film. (ν = 9.4148 GHz, 4 G modulation amplitude, 77 K).

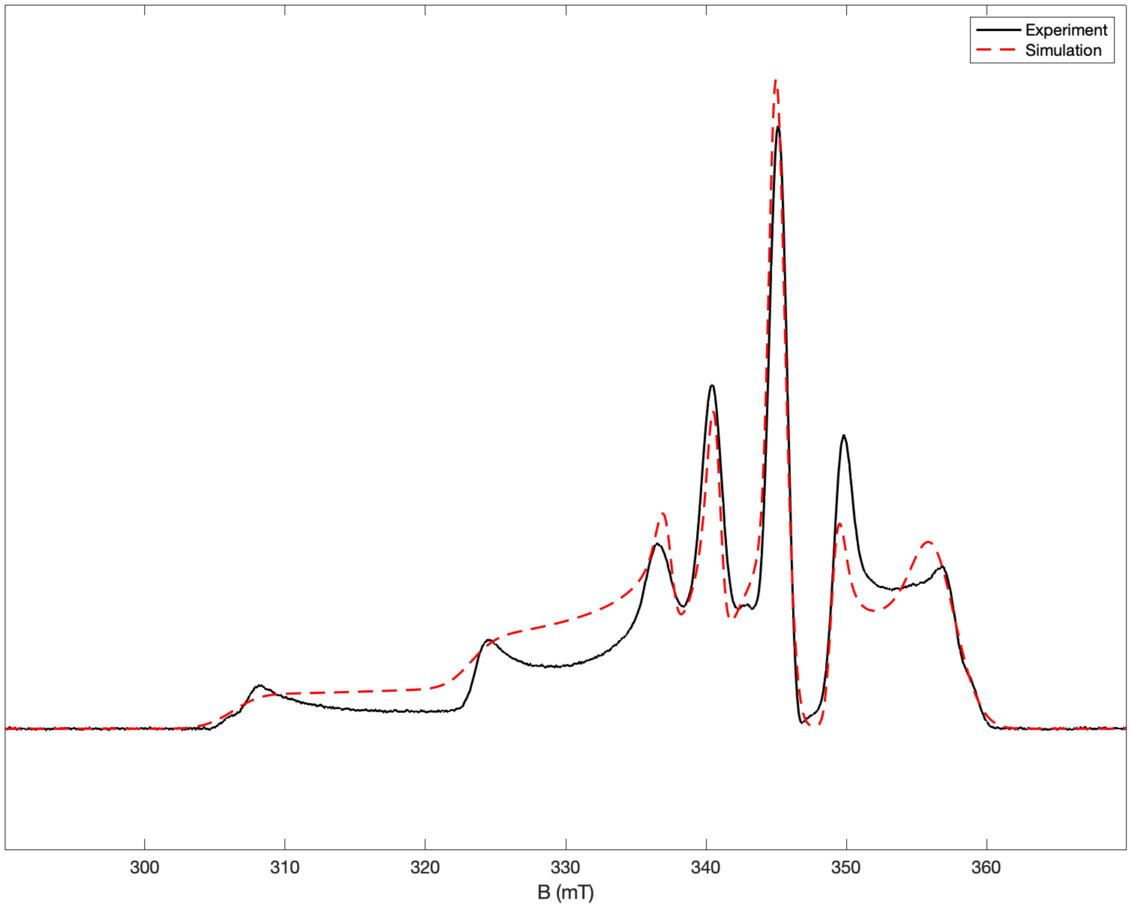

Figure S84: X-band pulsed EDFS spectrum and fit for Cu(dtc)$_2$ in PS film. (ν = 9.7280 GHz, 100 K).

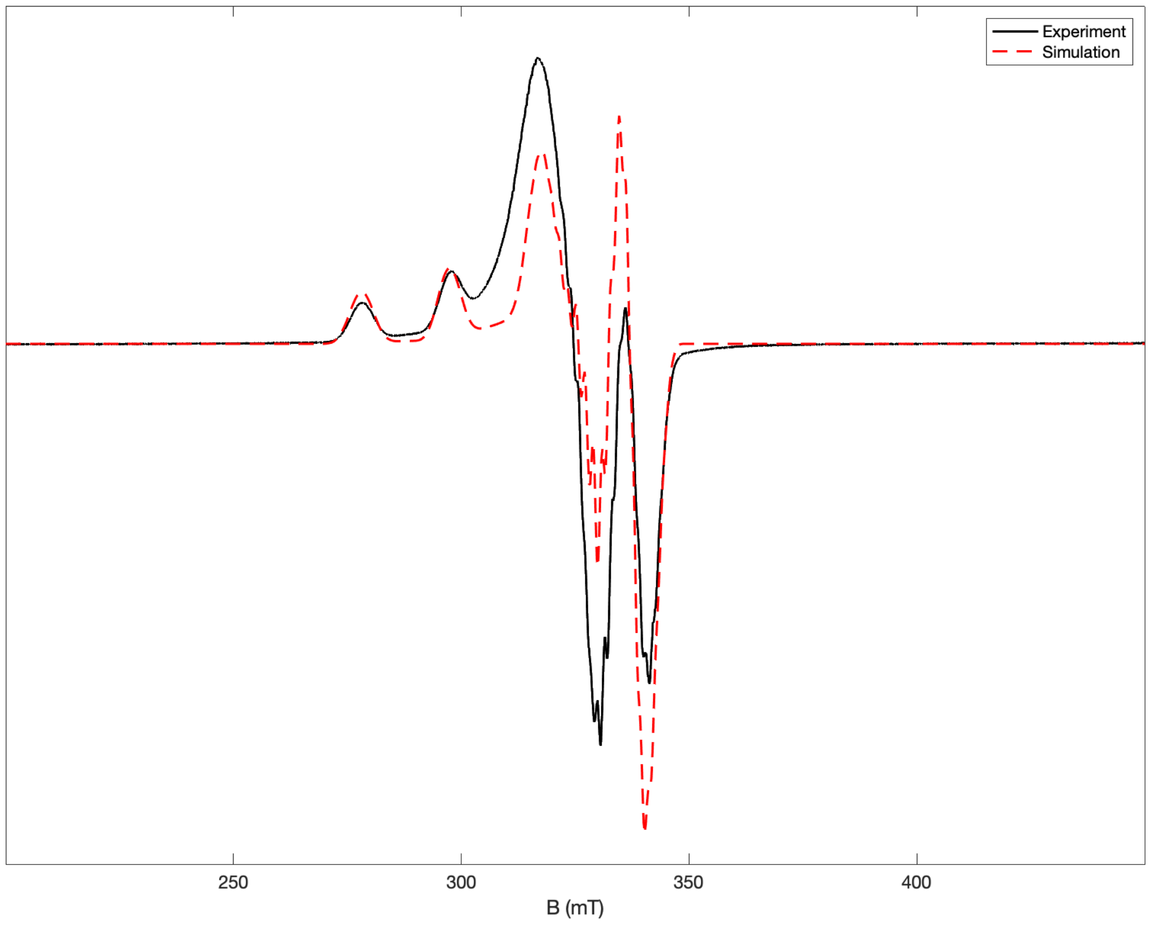

Figure S85: X-band CW EPR spectrum and fit for Cu(pci)$_2$ in PS film. (ν = 9.4148 GHz, 4 G modulation amplitude, 77 K).

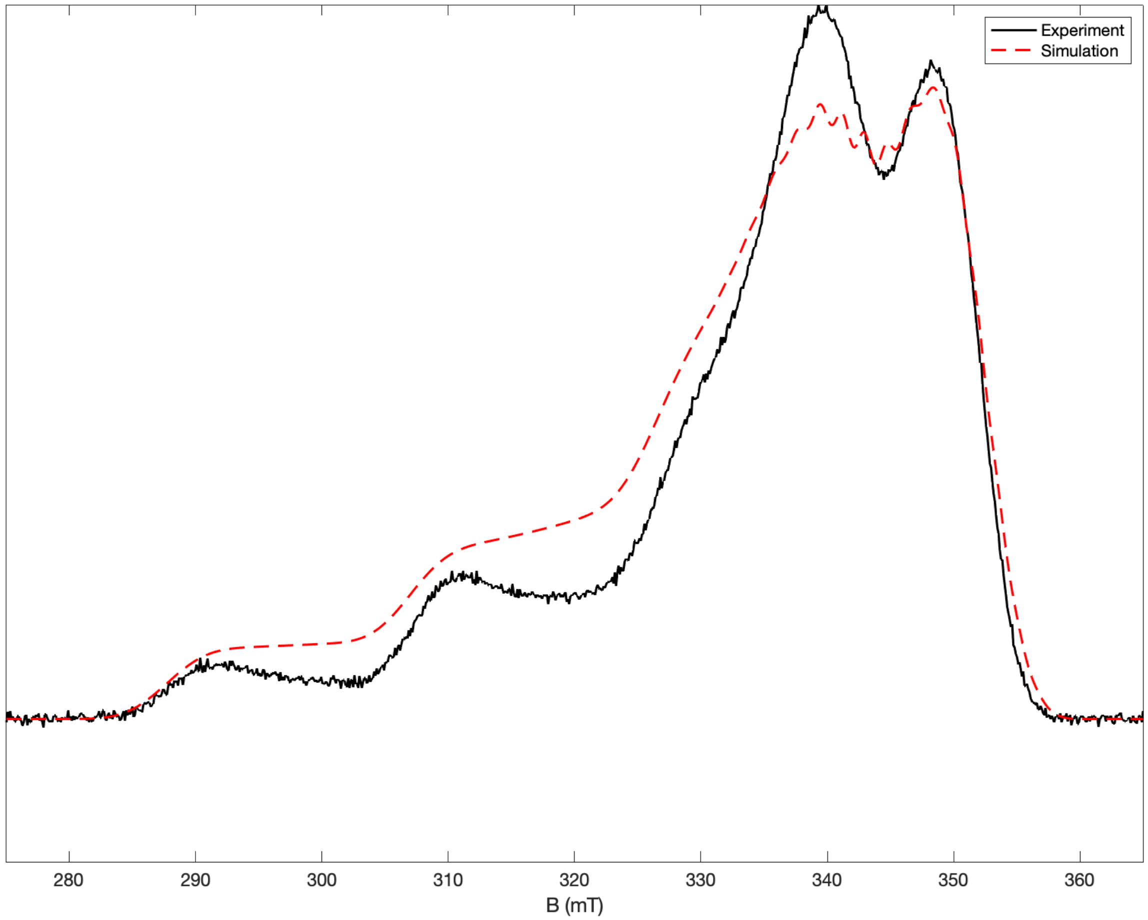

Figure S86: X-band pulsed EDFS spectrum and fit for Cu(pci)$_2$ in PS film. (ν = 9.7284 GHz, 100 K).

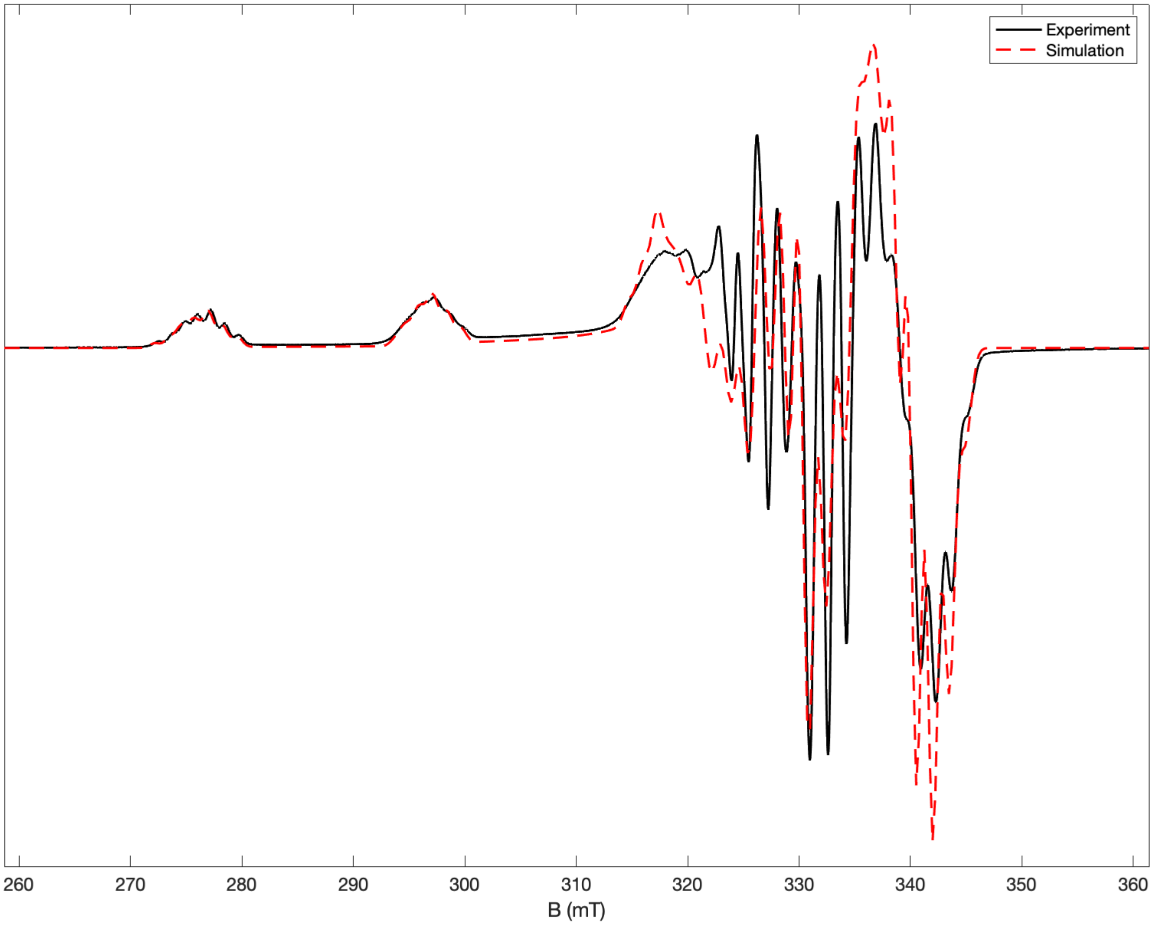

Figure S87: X-band CW EPR spectrum and fit for Cu(acacen) in PMMA film. (ν = 9.4101 GHz, 4 G modulation amplitude, 77 K).

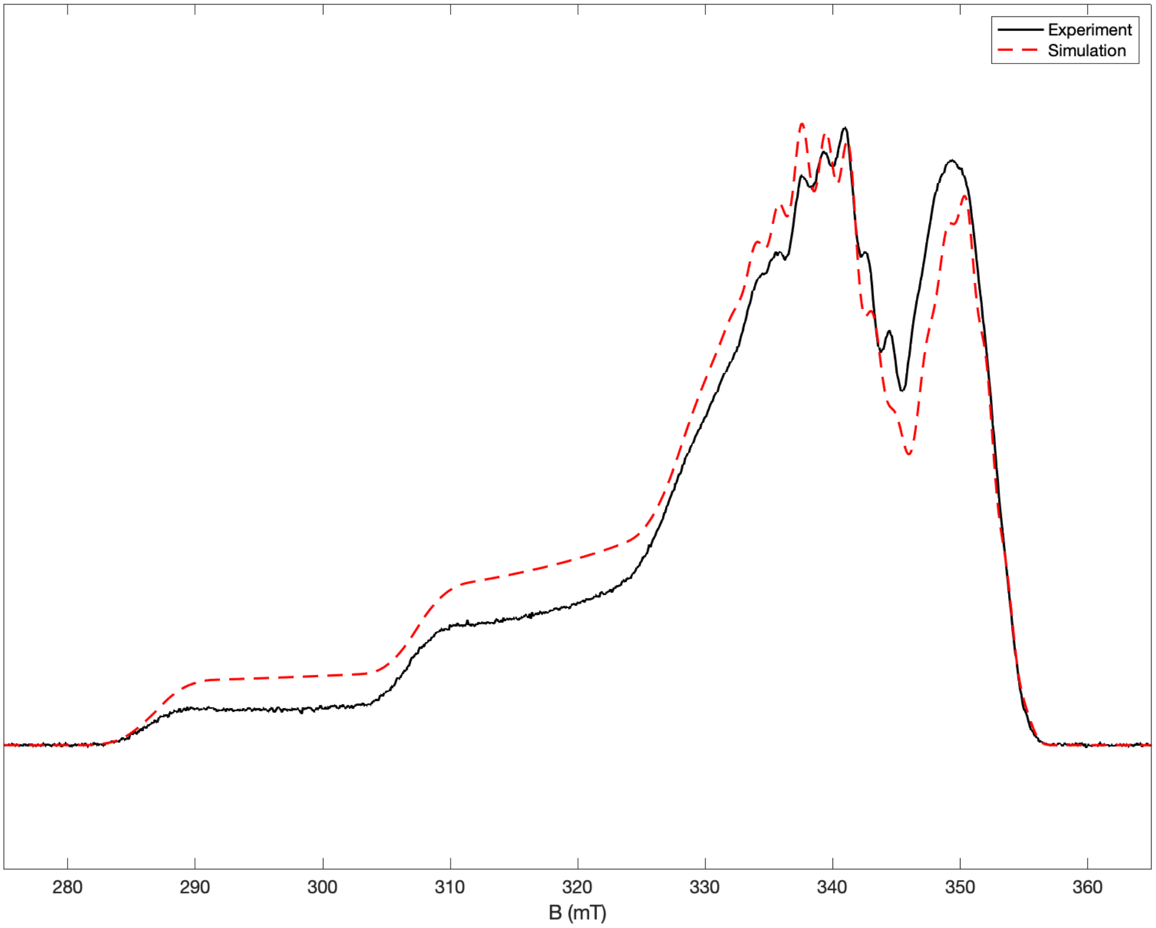

Figure S88: X-band pulsed EDFS spectrum and fit for Cu(acacen) in PMMA film. (ν = 9.7284 GHz, 100 K).

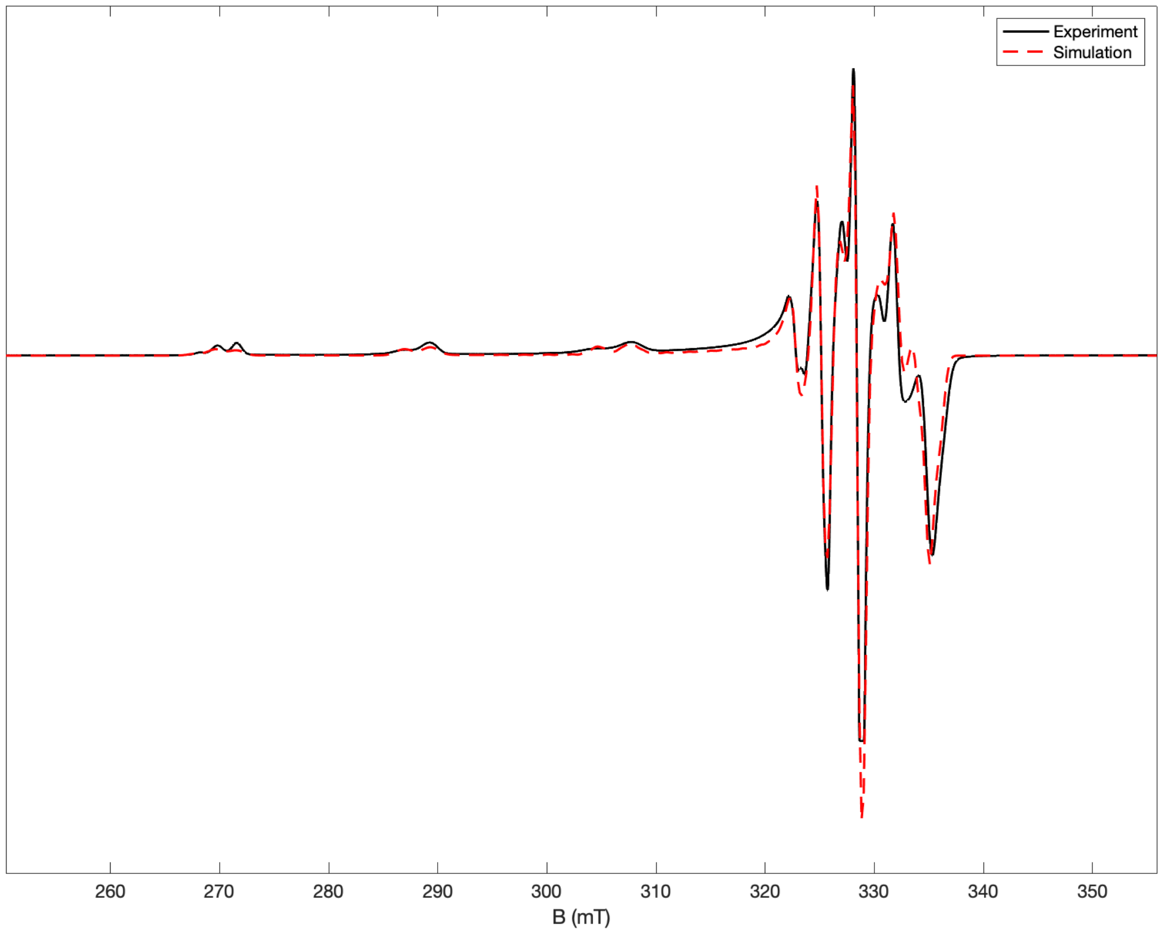

Figure S89: X-band CW EPR spectrum and fit for Cu(tmhd)$_2$ in PMMA film. (v = 9.3968 GHz, 4 G modulation amplitude, 77 K).

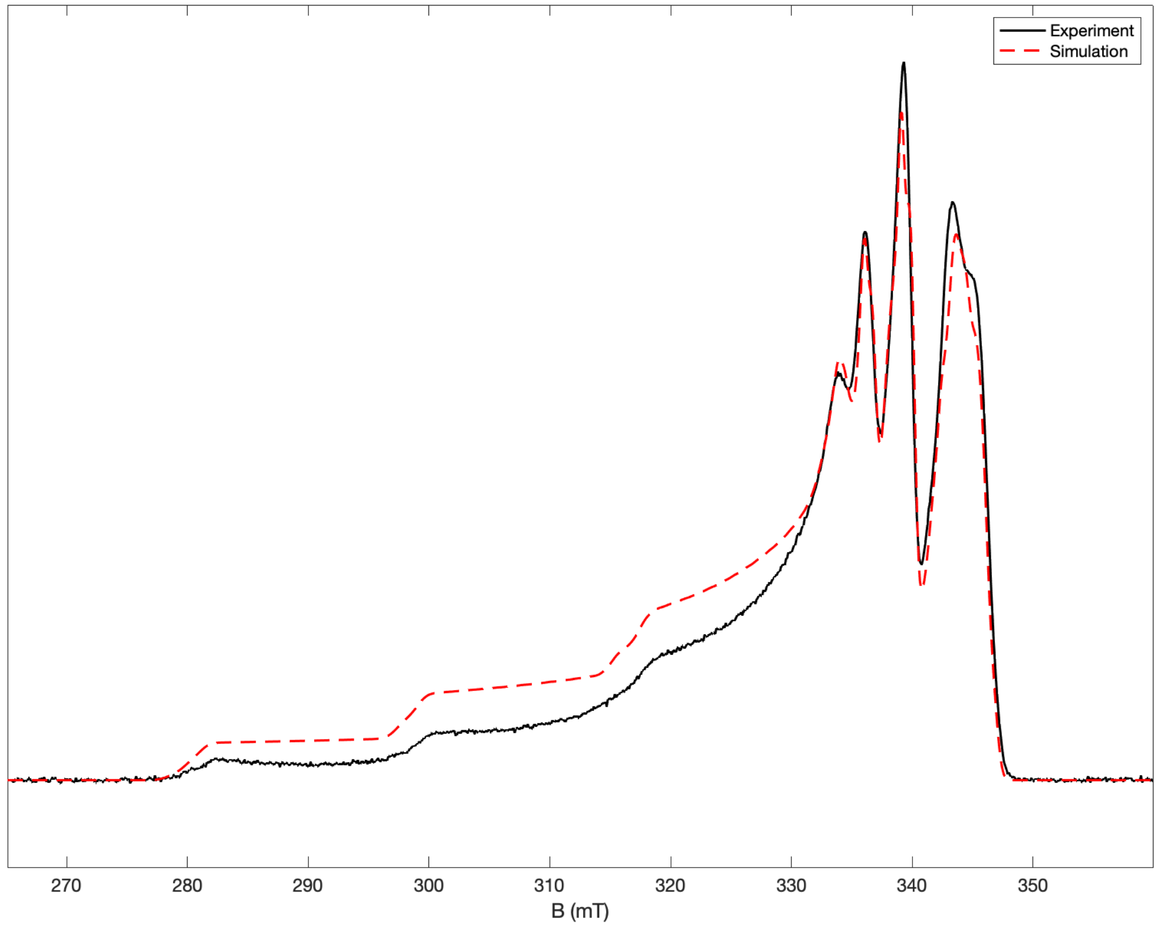

Figure S90: X-band pulsed EDFS spectrum and fit for $Cu(tmhd)_2$ in PMMA film. (ν = 9.7284 GHz, 100 K).

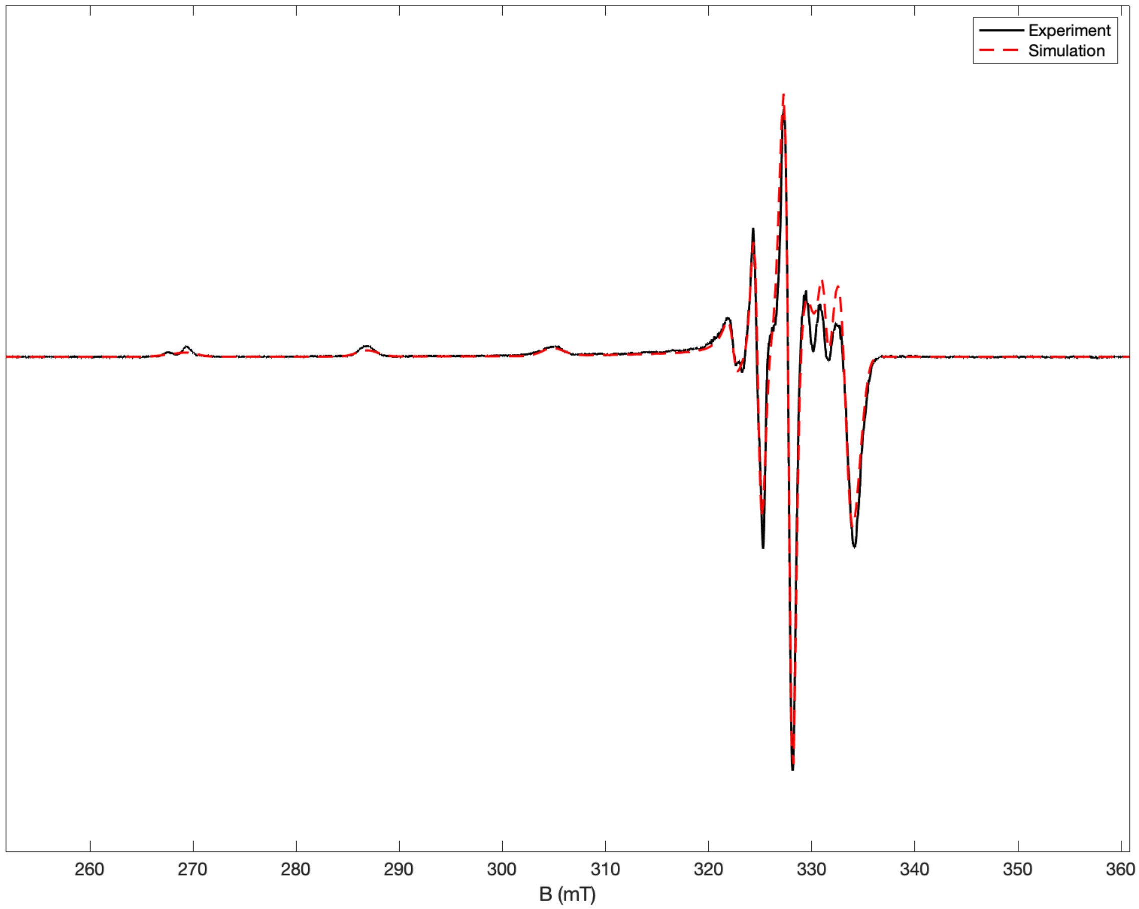

Figure S91: X-band CW EPR spectrum and fit for Cu(tbaa)$_2$ in PS film. (ν = 9.4007 GHz, 4 G modulation amplitude, 77 K).

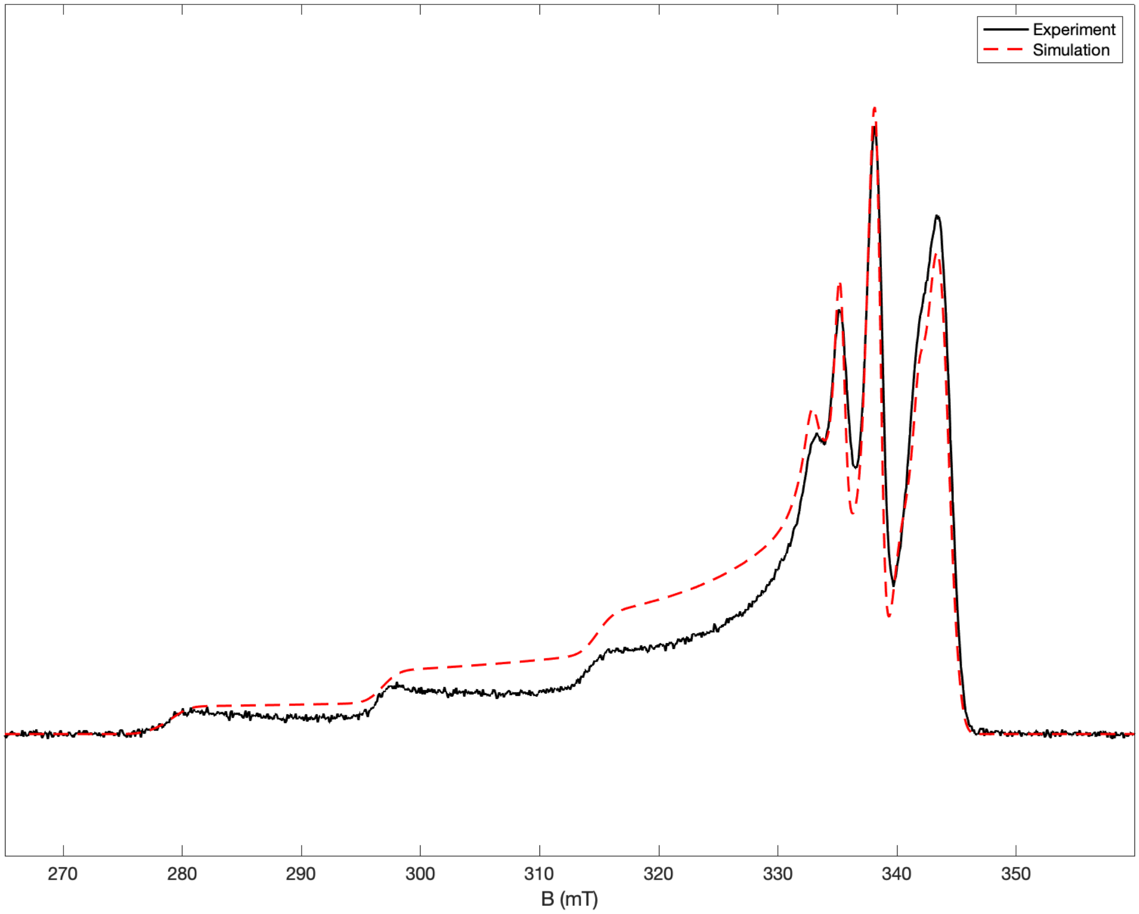

Figure S92: X-band pulsed EDFS spectrum and fit for Cu(tbaa)$_2$ in PS film. (ν = 9.7162 GHz, 100 K).

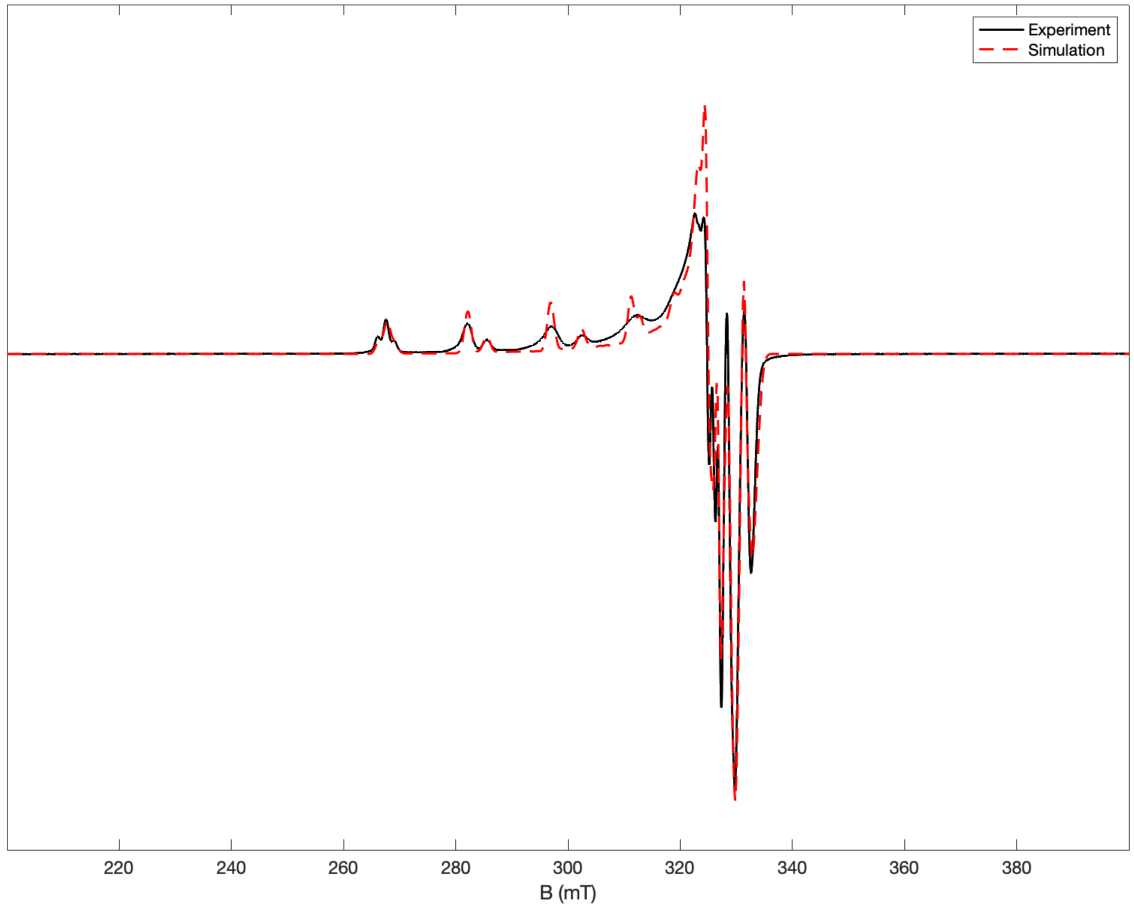

Figure S93: X-band CW EPR spectrum and fit for Cu(hfac)$_2$ in PS film. (ν = 9.4014 GHz, 4 G modulation amplitude, 77 K).

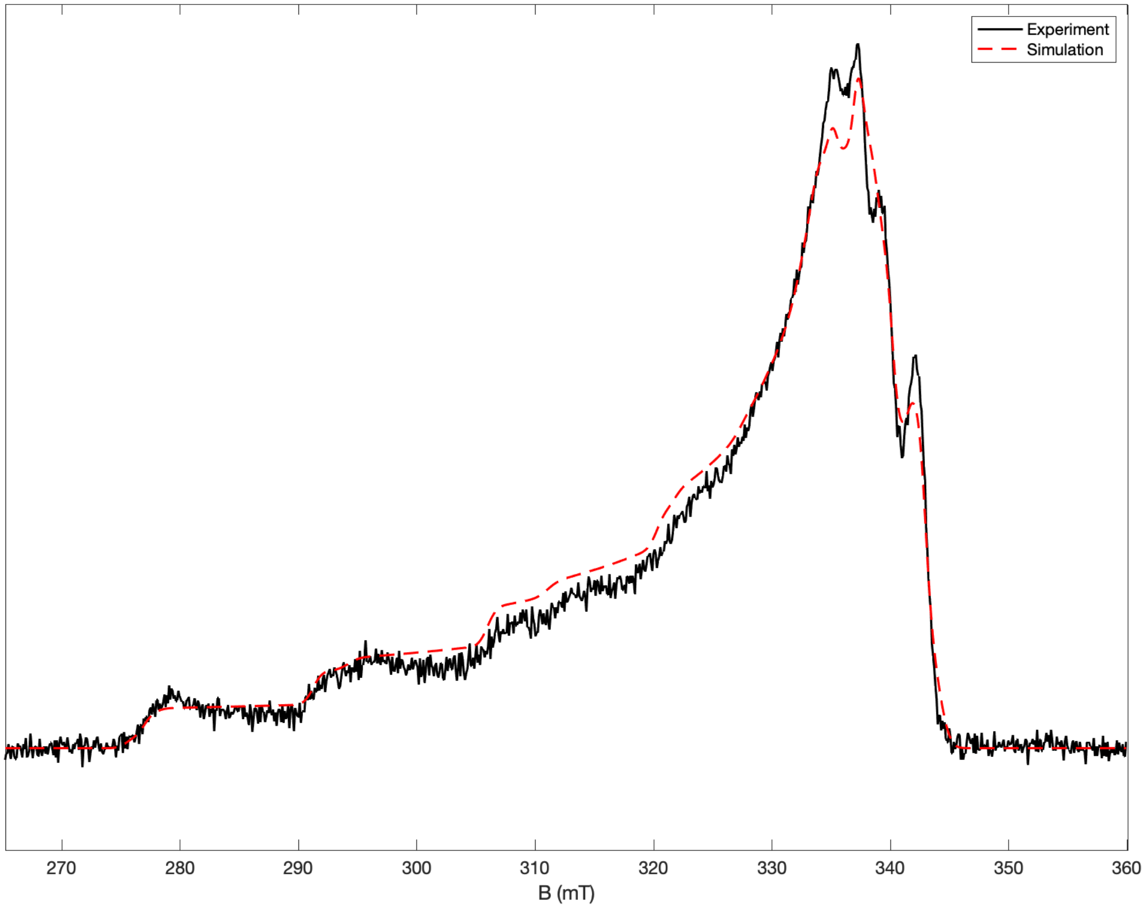

Figure S94: X-band pulsed EDFS spectrum and fit for Cu(hfac)$_2$ in PS film. (ν = 9.7165 GHz, 100 K).

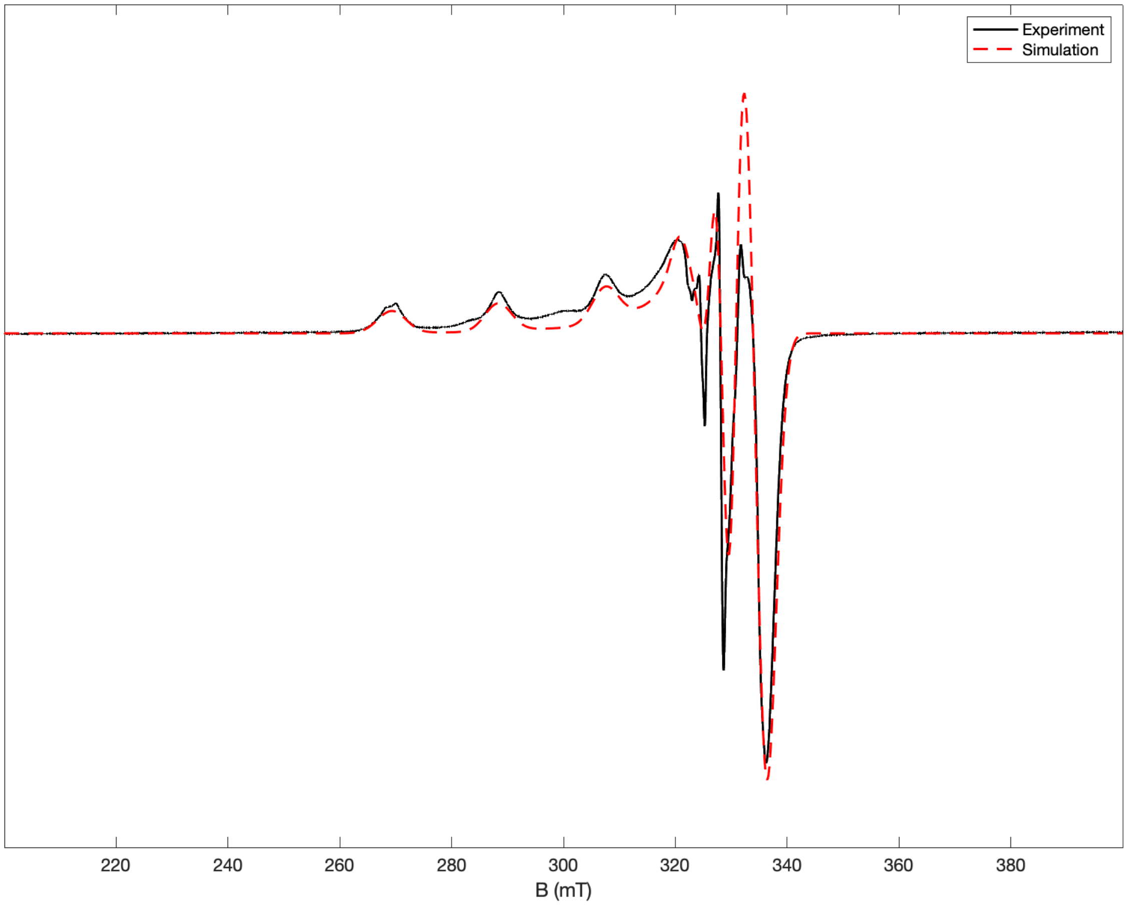

Figure S95: X-band CW EPR spectrum and fit for $(PPN)_2[Cu(ox)_2]$ in PS film. (ν = 9.4011 GHz, 4 G modulation amplitude, 77 K).

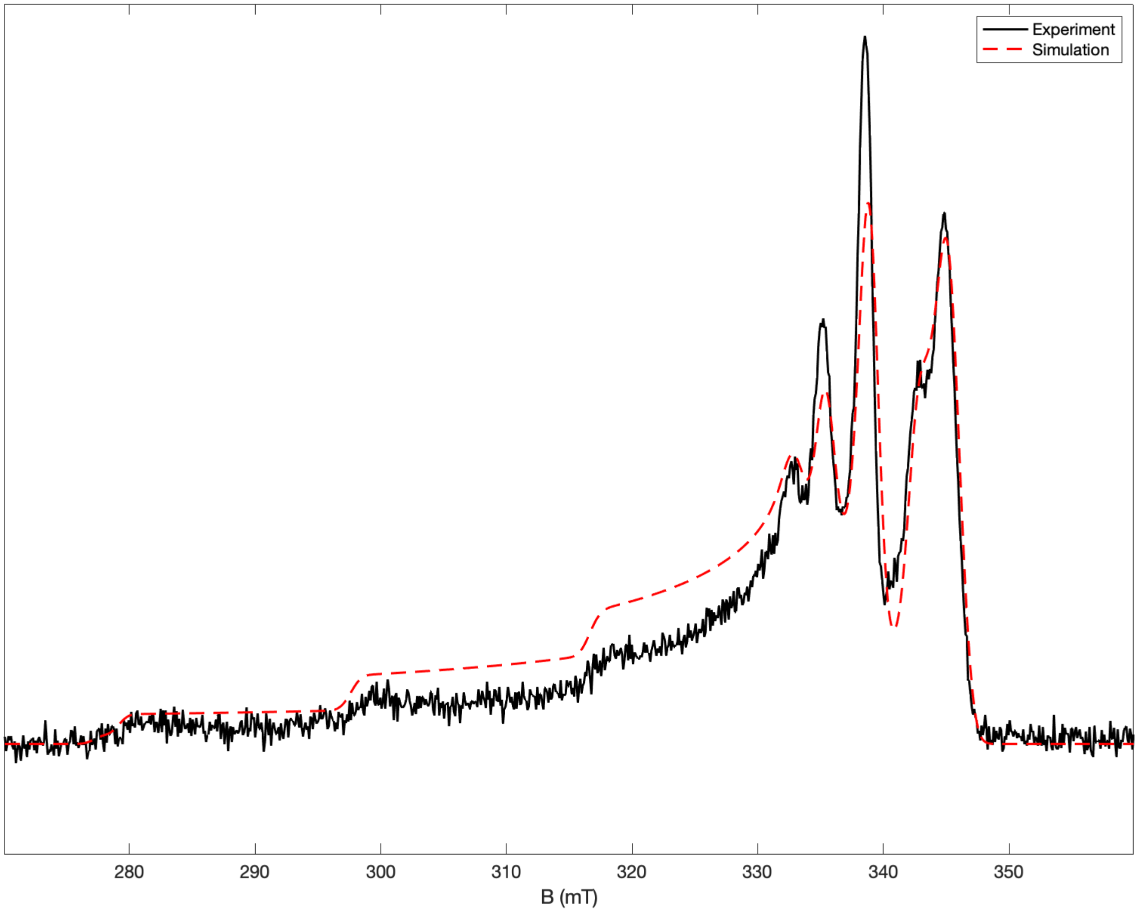

Figure S96: X-band pulsed EDFS spectrum and fit for $(PPN)_2[Cu(ox)_2]$ in PS film. (ν = 9.7161 GHz, 100 K).

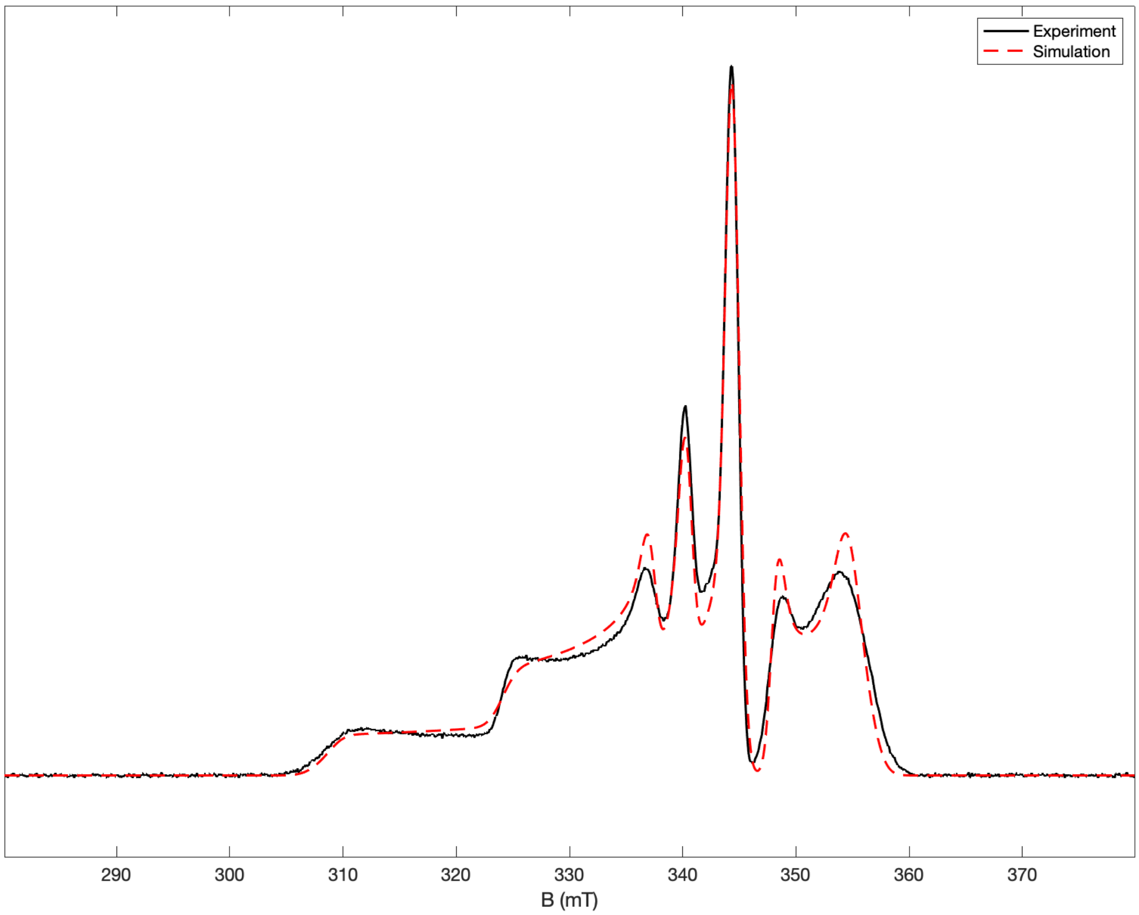

Figure S97: X-band pulsed EDFS spectrum and fit for $(PPh_4)_2[Cu(mnt)_2]$ in PMMA film. (ν = 9.7161 GHz, 100 K).

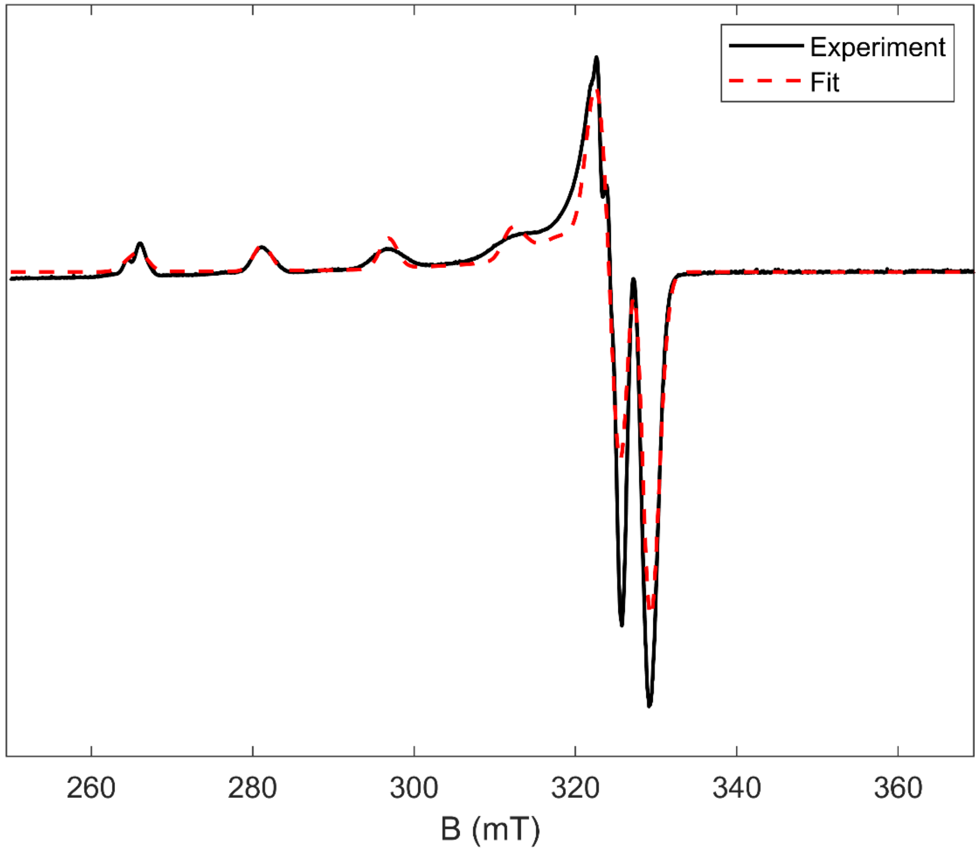

Figure S98: X-band CW EPR spectrum and fit for $K_2[Cu(ox)_2]$in 30%:70% glycerol:water. (ν = 9.3927 GHz, 4 G modulation amplitude, 77 K).

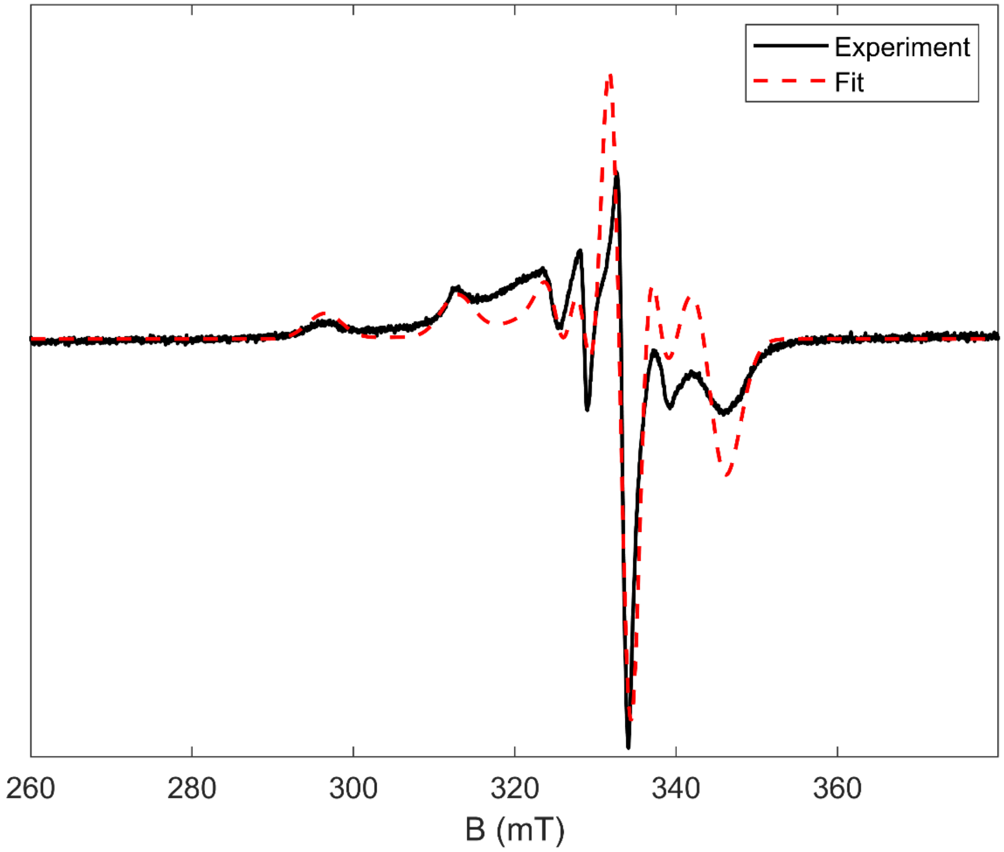

Figure S99: X-band CW EPR spectrum and fit for $(PPh_4)_2[Cu(bdt)_2]$ in 2:1 MeCN:toluene. (ν = 9.3927 GHz, 4 G modulation amplitude, 77 K).

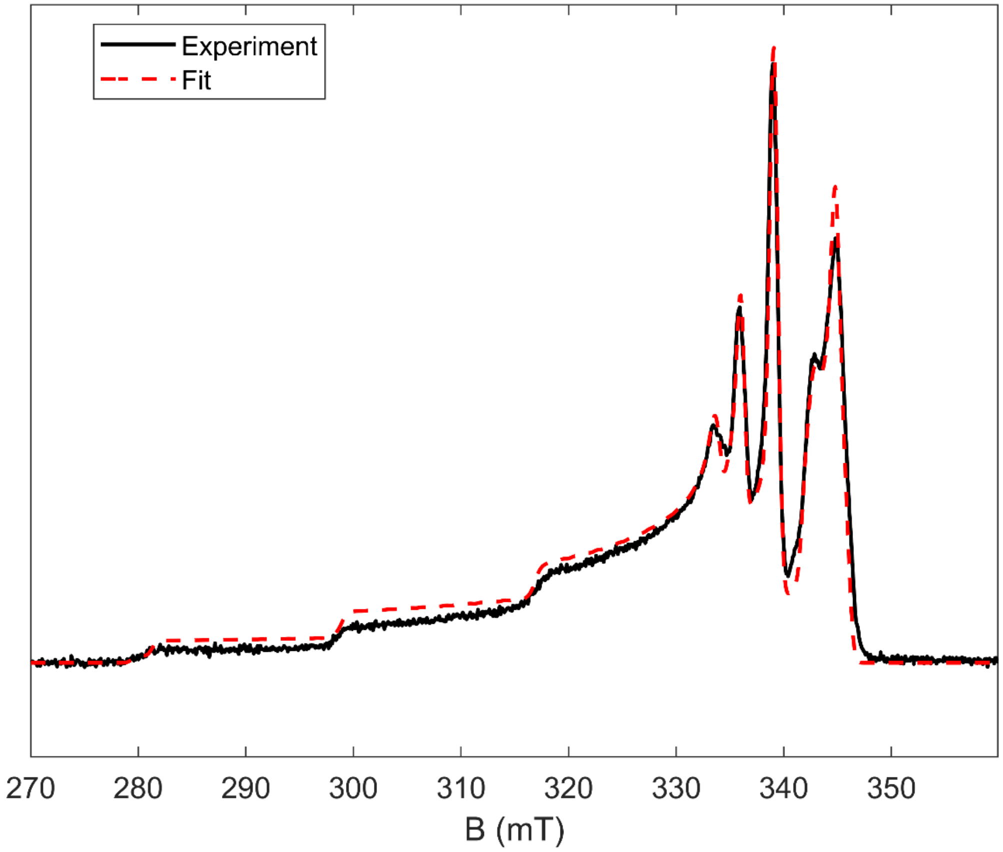

Figure S100: X-band pulsed EDFS spectrum and fit for Cu(acac)$_2$ in 3:1 DCM:benzene. (ν = 9.7257 GHz, 100 K).

6.2 Spin Relaxation EPR Data and Fitting

Table S26: Fitted time constants for pulse EPR samples. T_1 and T_m values reported for measurements at the powder line (peak of maximum intensity), which averages over all orientations of the molecule. T_1 recorded by inversion recovery, while T_m recorded by Hahn echo decay at X-band, 100 K.

Compound	Sample matrix	T_1 (μs)	T_1 % uncertainty	Inversion recovery β	T_m (μs)
$(PPh_4)_2[Cu(bdt)_2]$	2:1 MeCN:toluene	8.151	2.2	0.93	1.566
$(PPh_4)_2[Cu(mnt)_2]$	PMMA	5.086	0.4	0.91	0.682
$Cu(dtc)_2$	PS	3.277	0.3	0.89	0.248
Cu(acacen)	PMMA	1.380	0.7	0.91	0.225
$Cu(pci)_2$	PS	1.096	2.0	0.84	0.179
$Cu(tbaa)_2$	PS	0.636	0.5	0.94	0.203
$Cu(acac)_2$	3:1 DCM:benzene	0.573	1.5	0.92	
$Cu(tmhd)_2$	PMMA	0.565	0.7	0.92	0.152
$(PPN)_2[Cu(ox)_2]$	PS	0.480	4.6	0.89	0.417
$Cu(hfac)_2$	PS	0.309	5.0	0.73	0.117
$K_2[Cu(ox)_2]$	3:7 glycerol:water	0.254	3.4	0.86	0.247

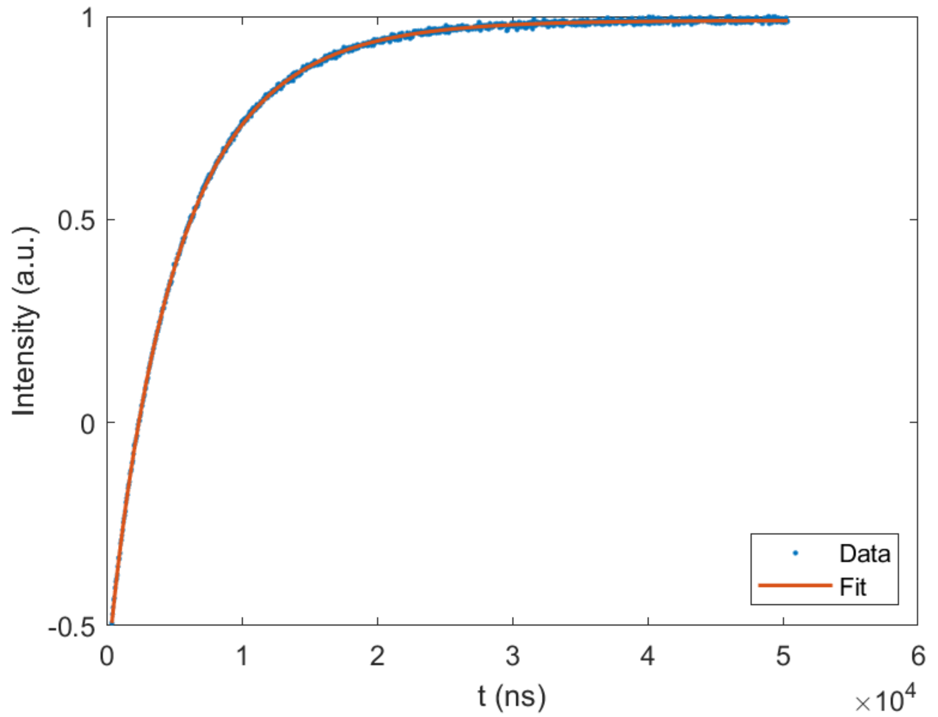

Figure S101: T_1 by inversion recovery for $(PPh_4)_2[Cu(mnt)_2]$ in PMMA film (9.7161 GHz, 3444 G, 100 K).

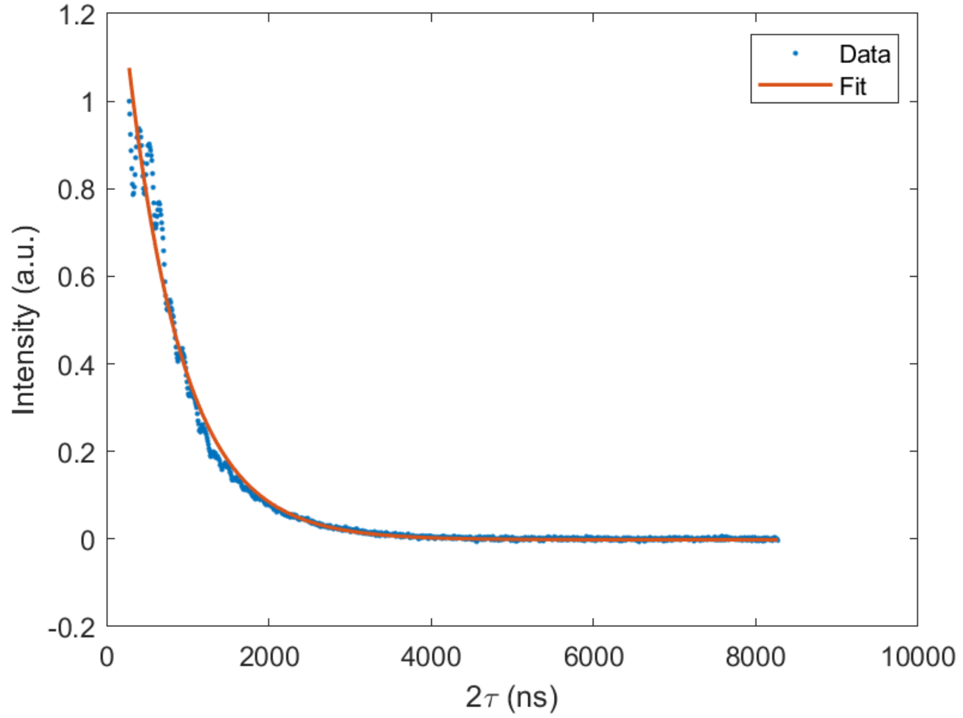

Figure S102: T_m by Hahn-echo decay for $(PPh_4)_2[Cu(mnt)_2]$ in PMMA film (9.7161 GHz, 3444 G, 100 K).

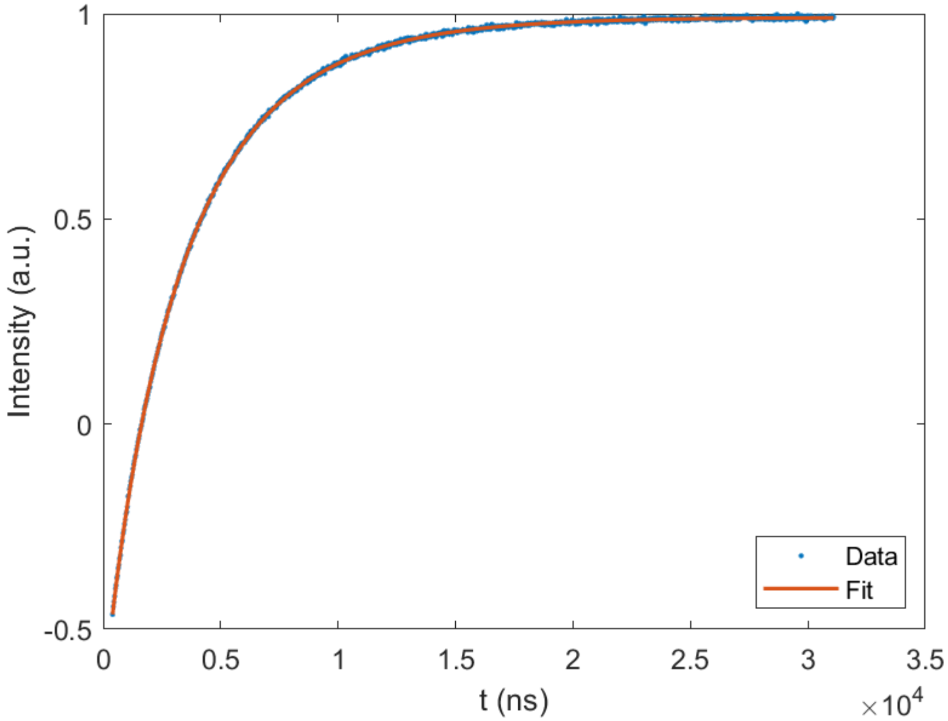

Figure S103: T_1 by inversion recovery for $Cu(dtc)_2$ in PS film (9.7280 GHz, 3451 G, 100 K).

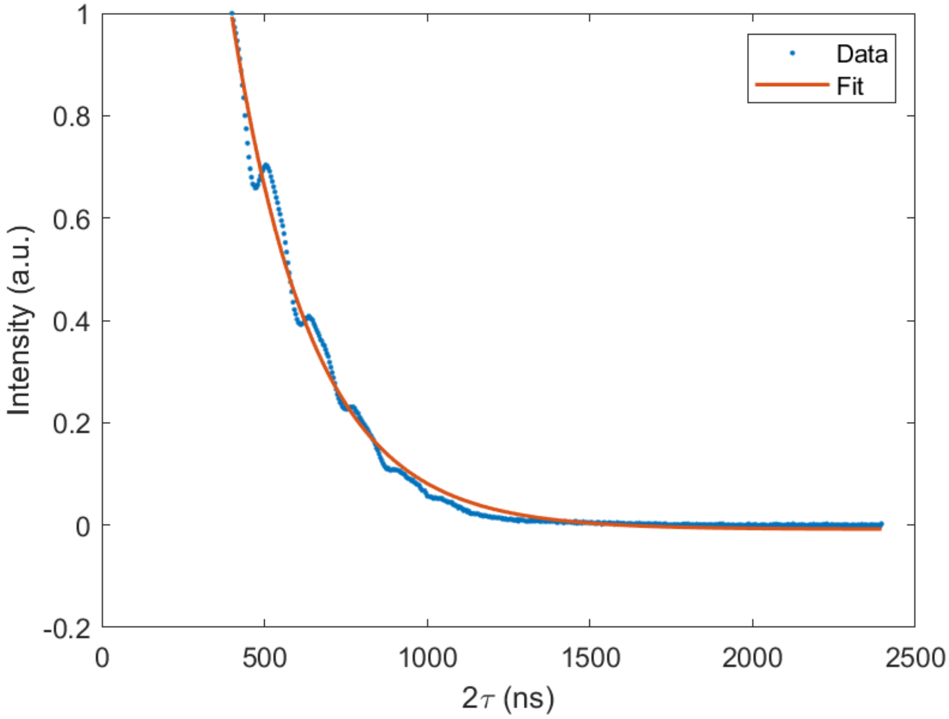

Figure S104: T_m by Hahn-echo decay for $Cu(dtc)_2$ in PS film (9.7280 GHz, 3451 G, 100 K).

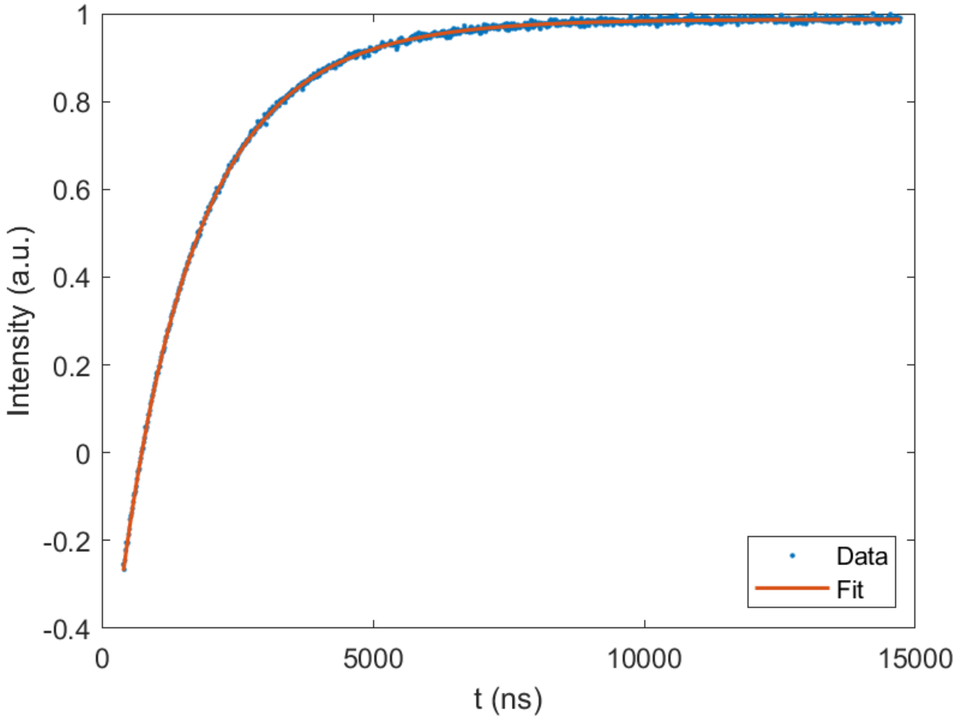

Figure S105: T_1 by inversion recovery for Cu(acacen) in PMMA film (9.7284 GHz, 3410 G, 100 K).

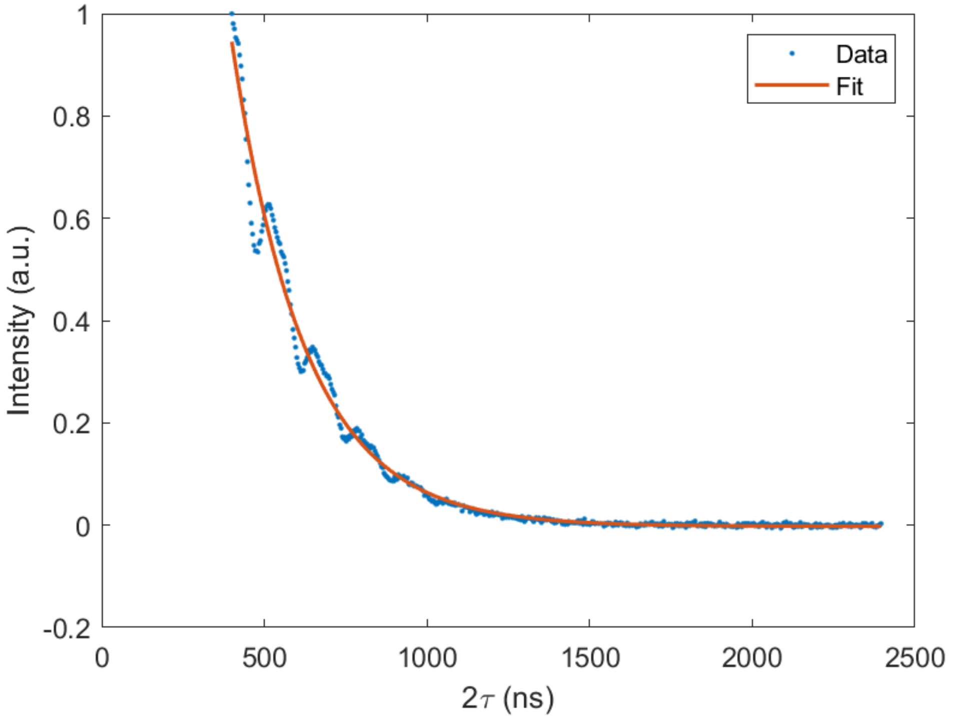

Figure S106: T_m by Hahn-echo decay for Cu(acacen) in PMMA film (9.7284 GHz, 3410 G, 100 K).

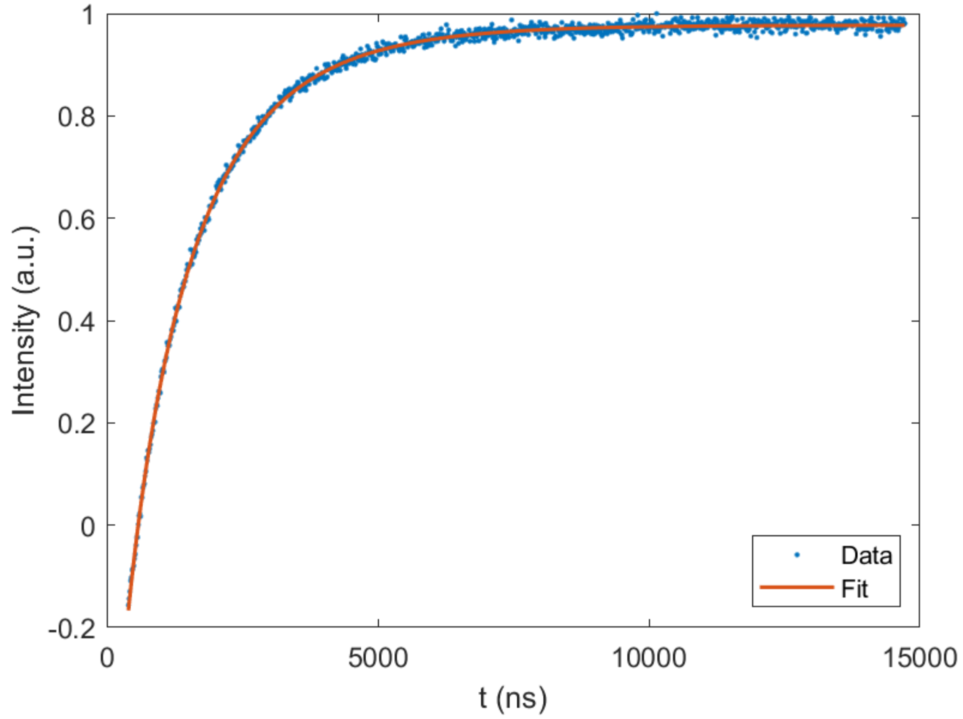

Figure S107: T_1 by inversion recovery for $Cu(pci)_2$ in PS film (9.7284 GHz, 3396 G, 100 K).

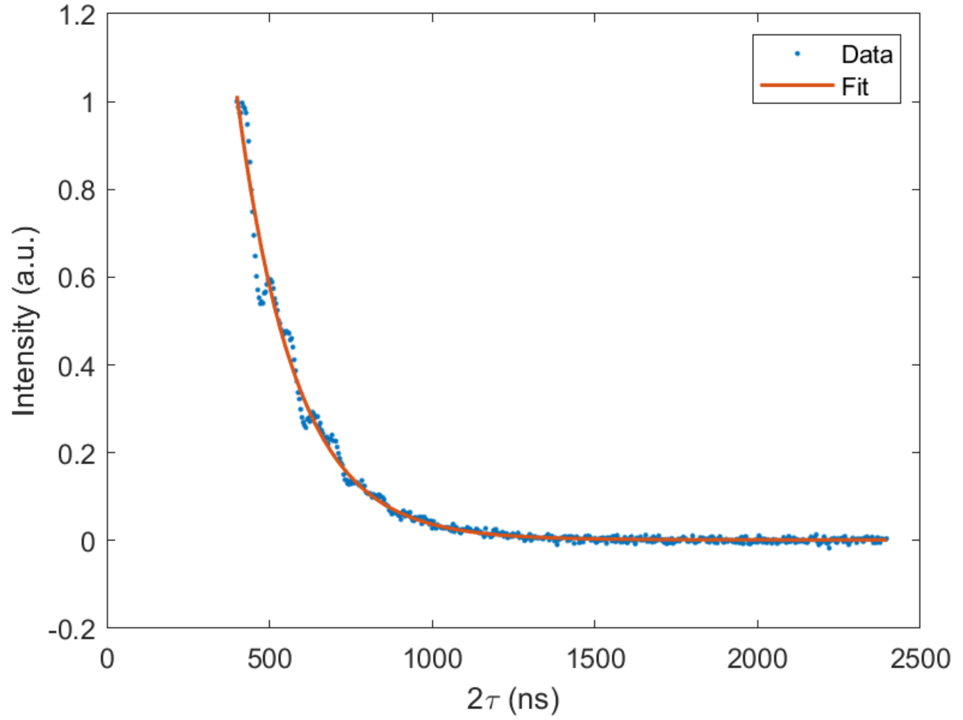

Figure S108: T_m by Hahn-echo decay for $Cu(pci)_2$ in PS film (9.7284 GHz, 3396 G, 100 K).

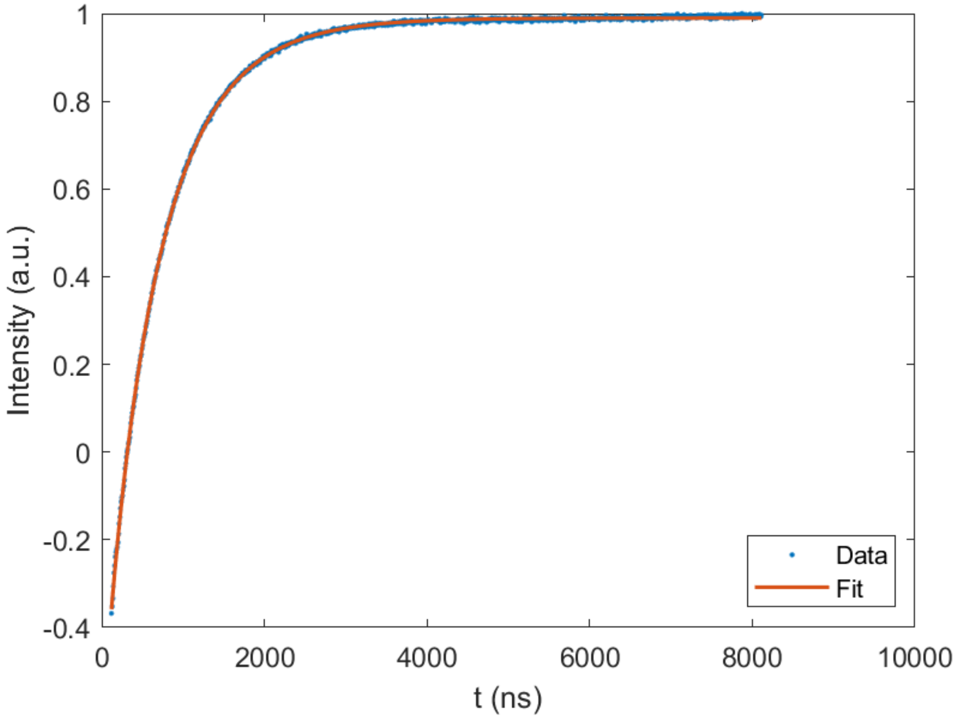

Figure S109: T_1 by inversion recovery for $Cu(tbaa)_2$ in PS film (9.7162 GHz, 3382 G, 100 K).

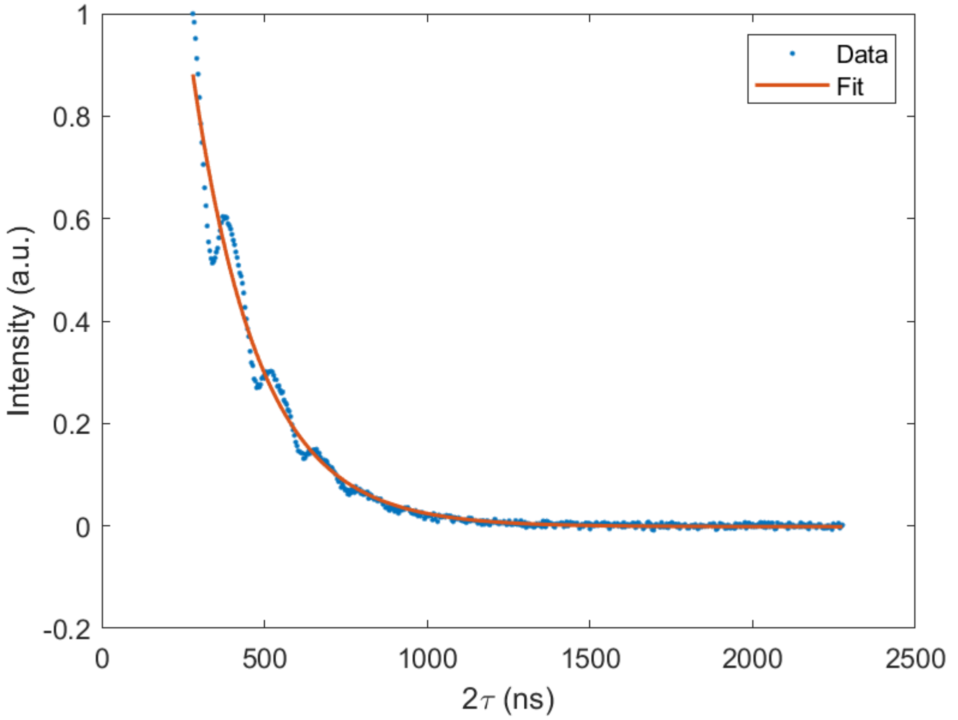

Figure S110: T_m by Hahn-echo decay for $Cu(tbaa)_2$ in PS film (9.7162 GHz, 3382 G, 100 K).

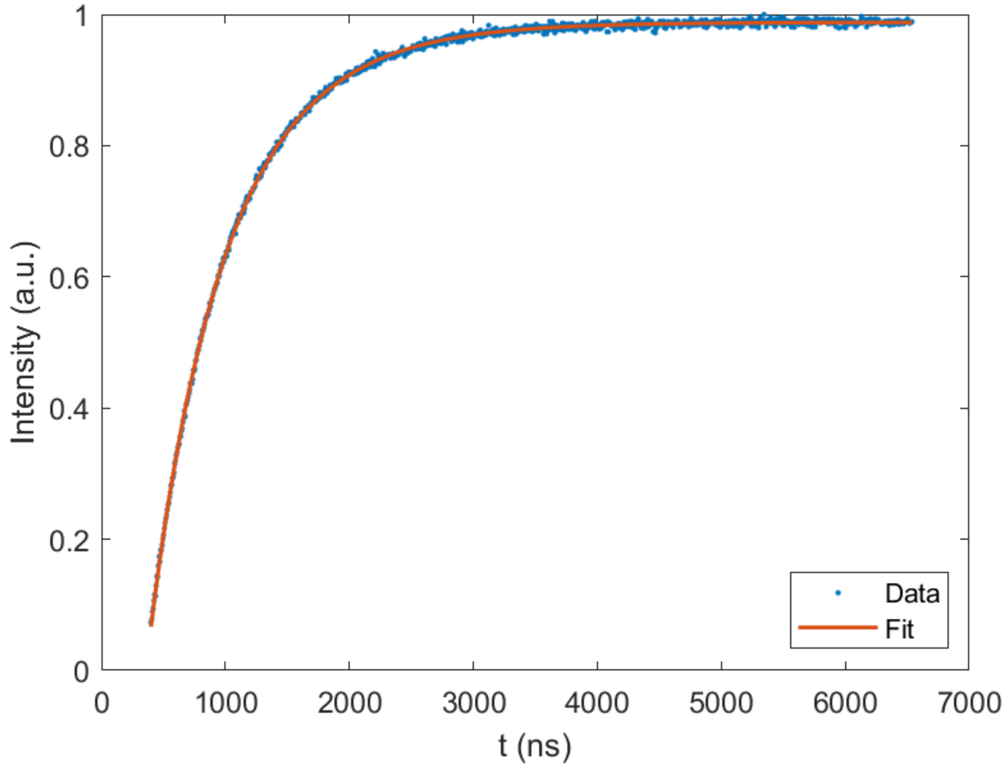

Figure S111: T_1 by Hahn-echo decay for $Cu(acac)_2$ in 3:1 DCM:benzene frozen glass (9.7257 GHz, 3390 G, 100 K).

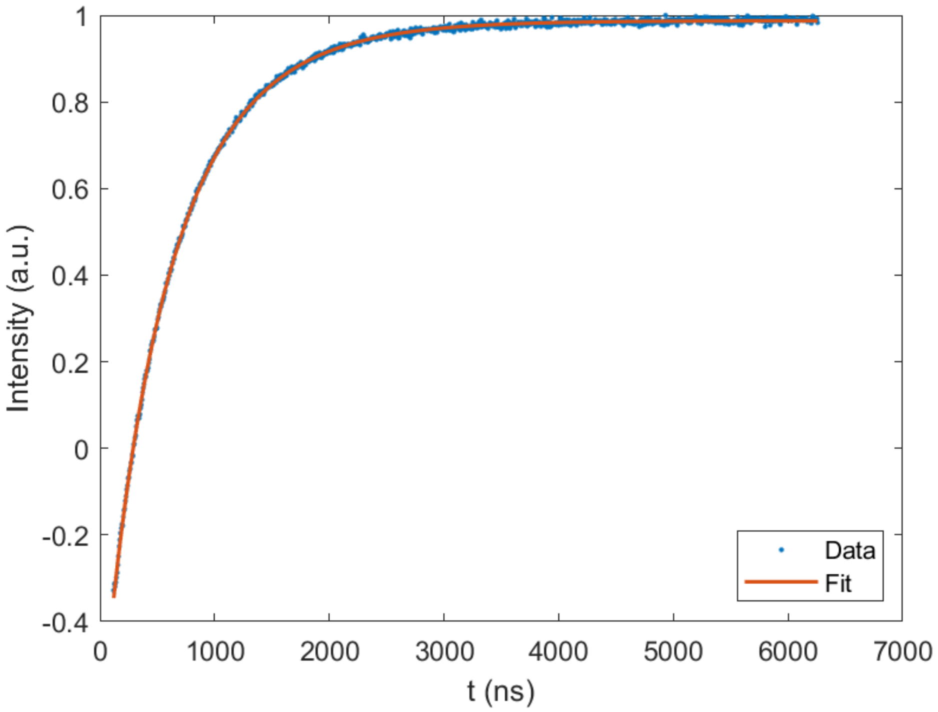

Figure S112: T_1 by inversion recovery for $Cu(tmhd)_2$ in PMMA film (9.7284 GHz, 3392 G, 100 K).

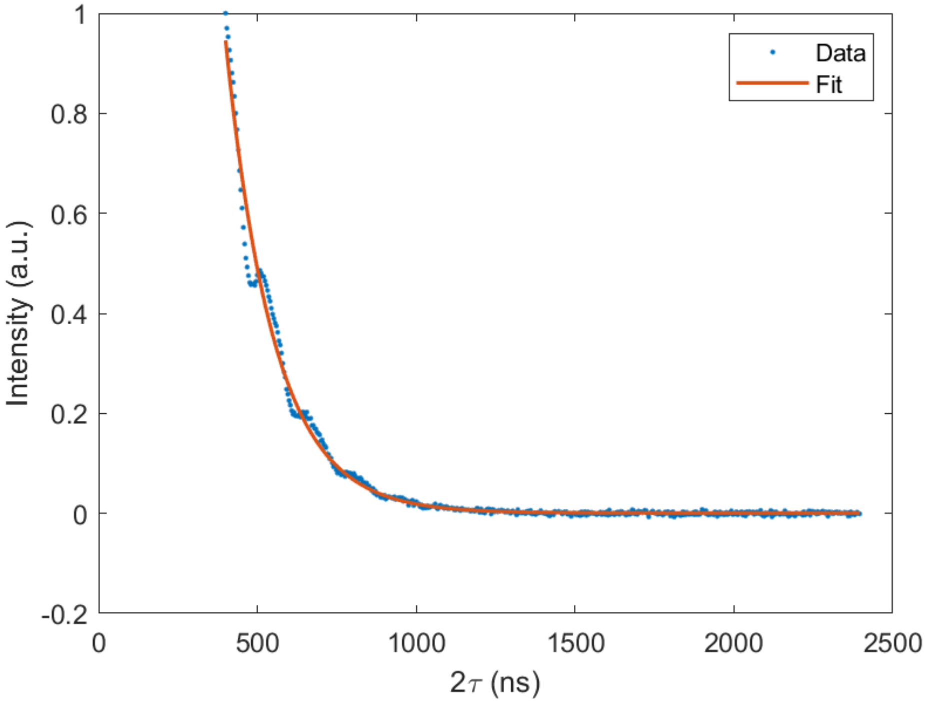

Figure S113: T_m by Hahn-echo decay for $Cu(tmhd)_2$ in PMMA film (9.7284 GHz, 3392 G, 100 K).

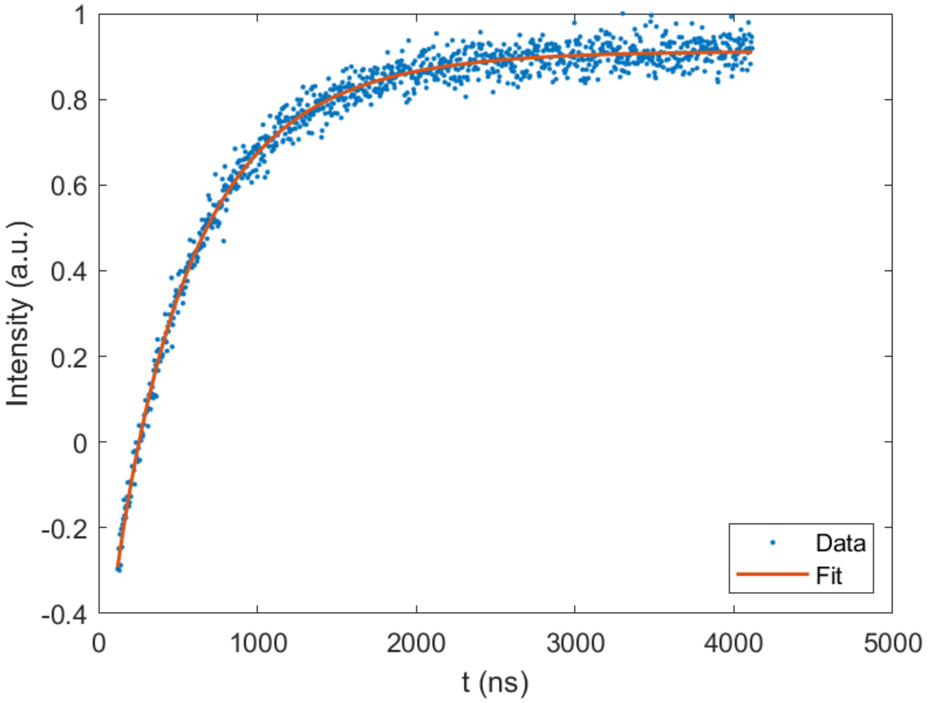

Figure S114: T_1 by inversion recovery for $(PPN)_2[Cu(ox)_2]$ in PS film (9.7161 GHz, 3386 G, 100 K).

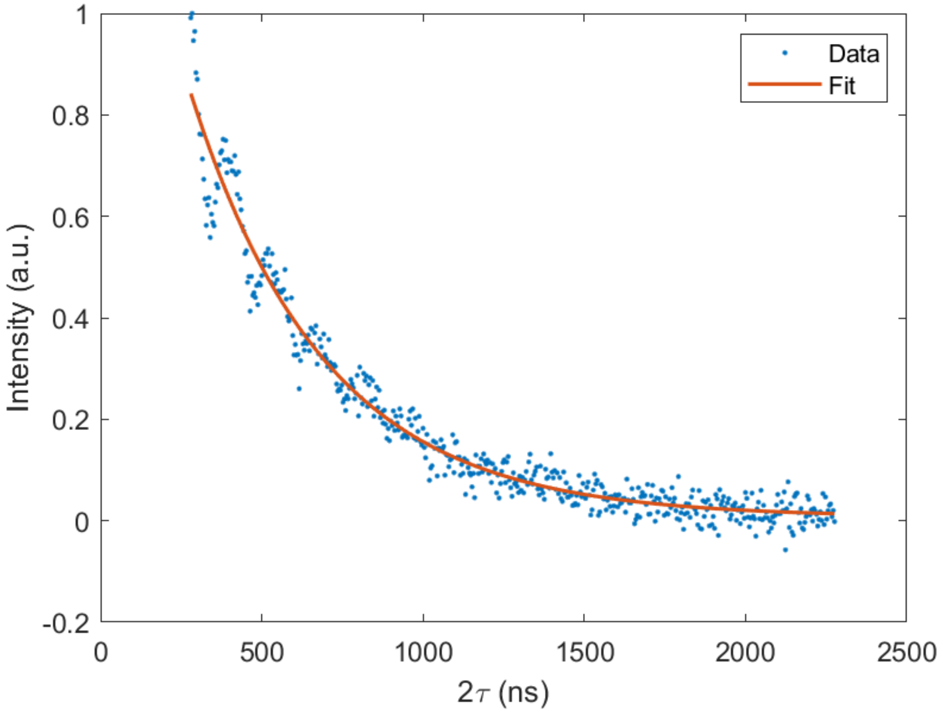

Figure S115: T_m by Hahn-echo decay for $(PPN)_2[Cu(ox)_2]$ in PS film (9.7161 GHz, 3386 G, 100 K).

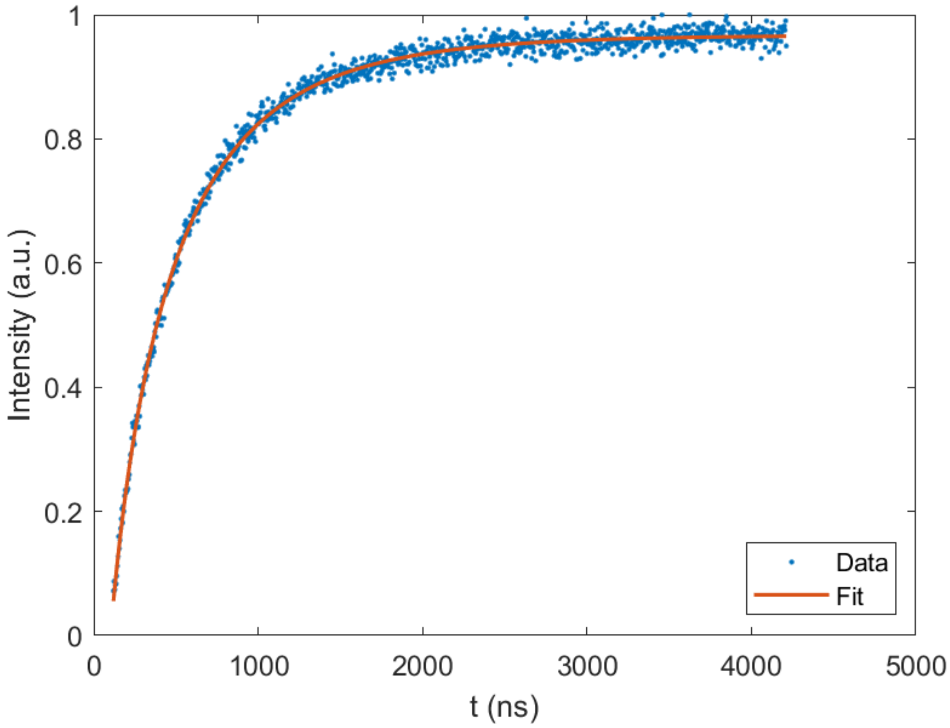

Figure S116: T_1 by inversion recovery for $Cu(hfac)_2$ in PS film (9.7165 GHz, 3372 G, 100 K).

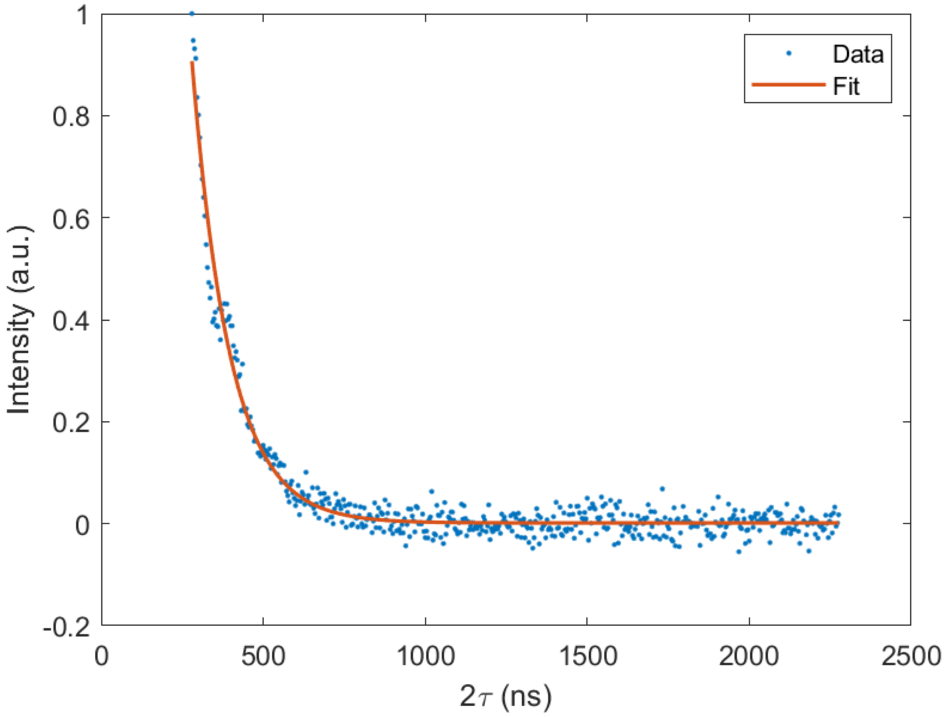

Figure S117: T_m by Hahn-echo decay for $Cu(hfac)_2$ in PS film (9.7165 GHz, 3372 G, 100 K).

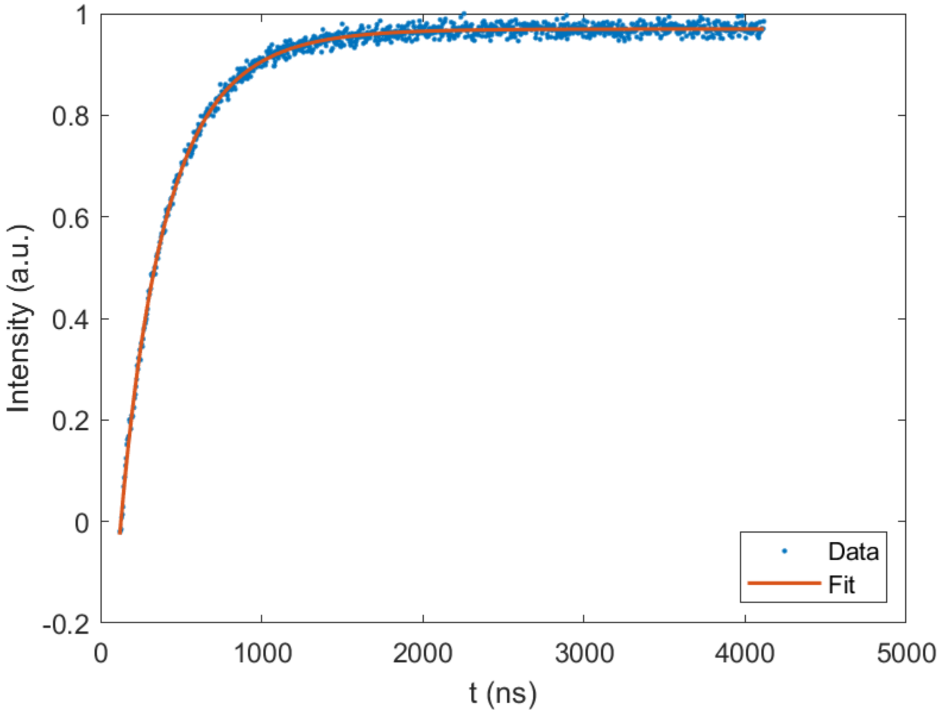

Figure S118: T_1 by inversion recovery for $K_2[Cu(ox)_2]$ in 3:7 glycerol:water (9.7174 GHz, 3352 G, 100 K).

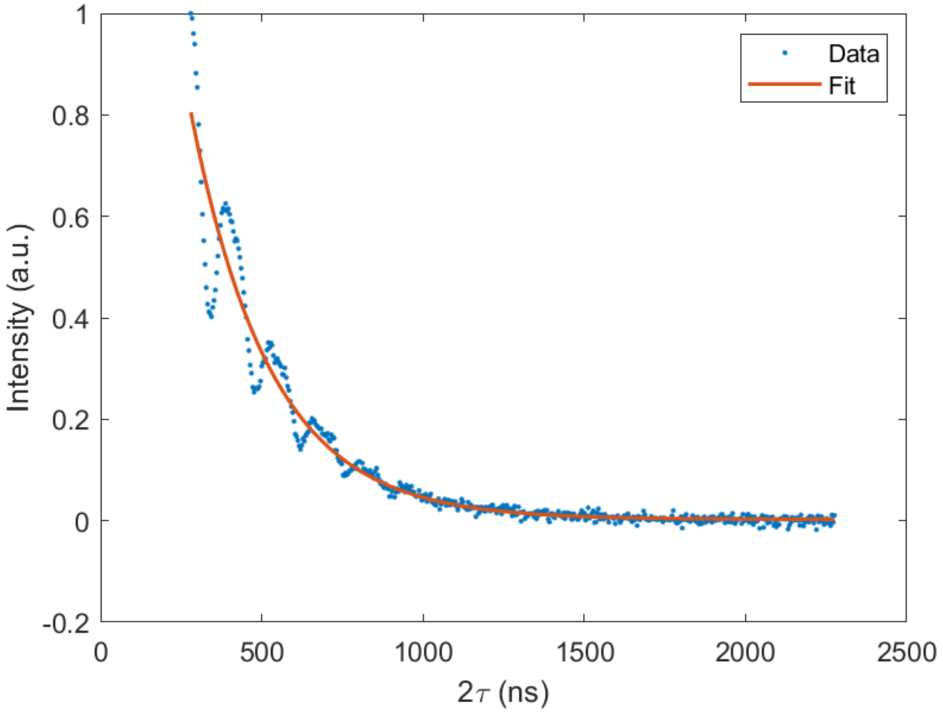

Figure S119: T_m by Hahn-echo decay for $K_2[Cu(ox)_2]$ in 3:7 glycerol:water (9.7174 GHz, 3352 G, 100 K).

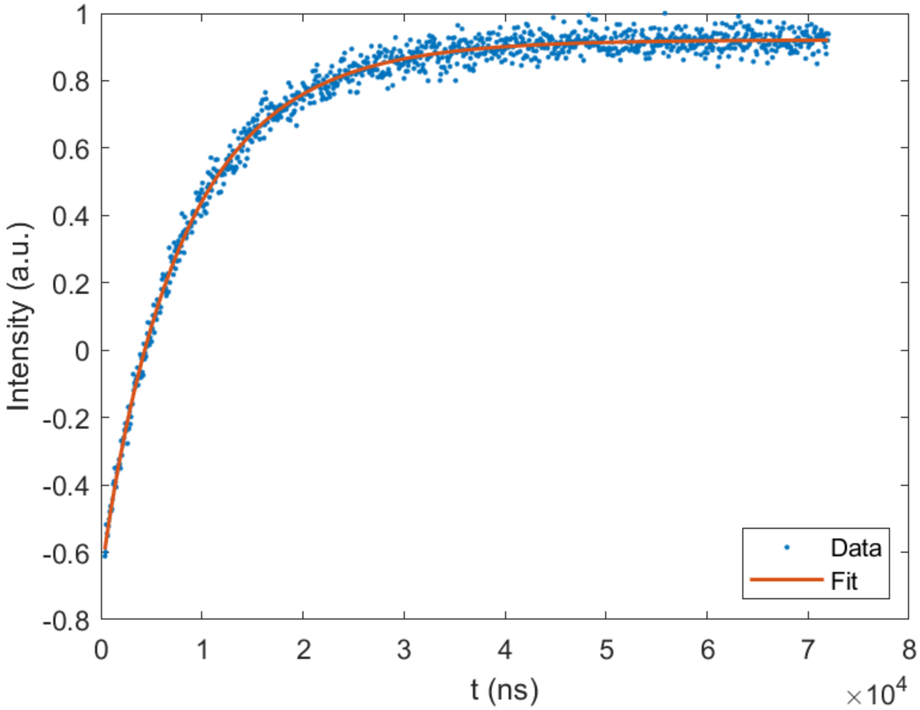

Figure S120: T_1 by inversion recovery for $(PPh_4)_2[Cu(mnt)_2]$ in 2:1 MeCN:toluene (9.7204 GHz, 3448 G, 100 K).

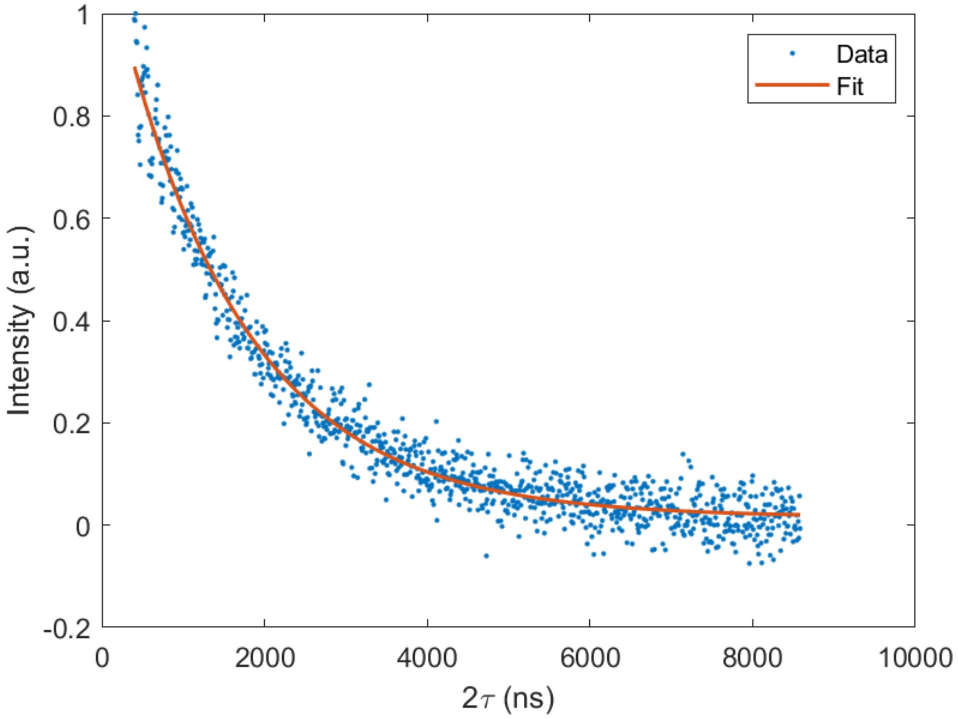

Figure S121: T_m by Hahn-echo decay for $(PPh_4)_2[Cu(mnt)_2]$ in 2:1 MeCN:toluene (9.7204 GHz, 3448 G, 100 K).

6.3 Orbital Reduction Factors for T_1 Scaling

While MCD directly measures the change in d-d state energies across this compound series, there may be changes in metal-ligand bond covalency that impact T_1 as well. To estimate these effects, we used the spin Hamiltonian fits to the EPR spectra to extract effective orbital reduction factors. These orbital reduction factors describe the reduction of spin-orbit coupling due to covalency and potentially other effects, as compared to free-ion d-orbitals. Orbital reduction factors are then used to refine the trend of $1/T_1$ vs. ΔE within the context of the dg/dQ model.

As given in the main text, the g values are given by the following expression:

$$g_i = g_e - 2\lambda \sum_{e \neq g} \frac{\left|\langle \Psi_e | \hat{L}_i | \Psi_g \rangle\right|^2}{E_e - E_g} \tag{S9}$$

The matrix element in the numerator is proportional to the orbital angular momentum matrix elements for the free-ion d-orbital, multiplied by coefficients which account for the fraction of the ground and excited states composed of this d-orbital character. The orbital coefficients $\alpha^2\beta^2$ can be lumped together into an "orbital reduction factor", η. The absolute value of the matrix element is denoted as k.

$$\left|\langle \Psi_e | \hat{L}_i | \Psi_g \rangle\right|^2 = \left|\langle d_e | \hat{L}_i | d_g \rangle\right|^2 \alpha^2\beta^2 = k_i^{\,2}\eta \tag{S10}$$

For a square planar Cu(II) complex, inspection of a table of angular momentum matrix elements shows that $k_i = 1$ for g_x and g_y, while $k_i = 2$ for g_z owing to the larger L_z matrix element between xy and x^2–y^2. We can solve for the average orbital reduction η as follows:

$$\eta = \frac{1}{3} \sum_{i=x,y,z} \frac{|g_i - g_e|(\Delta E_i)}{2k_i^{\,2}|\lambda|} \tag{S11}$$

Here ΔE_i is the excited state energy gap appropriate for the given spin-orbit coupling orientation, and λ = -830 cm^{-1} is the spin-orbit coupling constant for Cu(II). From this equation, we obtain the following effective orbital reduction factors (listed with the average d-d energy for comparison) in **Table S27**.

Table S27: Tabulation of orbital reduction factors extracted from EPR g values and MCD excited state energies, according to Equation S11.

Compound	η	Average d-d (cm^{-1})
$(PPh_4)_2[Cu(mnt)_2]$	0.264	20070
$Cu(dtc)_2$	0.239	18795
$Cu(pci)_2$	0.505	18166
Cu(acacen)	0.458	17849
$Cu(tbaa)_2$	0.547	16176
$Cu(tmhd)_2$	0.535	16607
$(PPN_2)[Cu(ox)_2]$	0.504	16235
$K_2[Cu(ox)_2]$	0.641	15252
$Cu(hfac)_2$	0.570	15234
$(PPh_4)_2[Cu(bdt)_2]$	0.243	20463
$Cu(acac)_2$	0.558	16823

Evidently, compounds with larger d-d excited state energies tend to have smaller orbital reduction factors. This indicates that bond covalency and excited state energy are trending in the same way for this series, and combined, they may account for a larger fraction of the $1/T_1 \propto \Delta E^{-11}$ than excited state energies alone. We thus sought to quantify the effect of these orbital reduction factors on the correlation in the context of the dg/dQ model. The leading order term for spin-phonon coupling is given as follows:[18]

$$\frac{\partial g_i}{\partial Q} = 2\lambda {k_i}^2 \left(\frac{\eta}{\Delta E^2}\right)\left(\frac{\partial E}{\partial Q}\right) + \cdots \quad \text{(S12)}$$

Without the impact of η, dg/dQ $\propto 1/T_1$ implies $1/T_1 \propto \Delta E^{-2}$. When the collinear effects of η are included (**Figure 6A**), we find that $1/T_1 \propto \Delta E^{-5}$ is approximately obtained. Thus, variation in bond covalency across this compound series may also account for some of the changes observed, but the entirety of the $1/T_1 \propto \Delta E^{-11}$ scaling still falls well outside of these predictions. Further theoretical modeling is needed to fully elucidate the origins of this effect.

7. Computational Methods

7.1 Analysis of Excited State Energies

All computations were performed using Orca 5.0.3.[19] Geometry optimizations were conducted using the CPCM implicit solvation model without a solvent specified to approximate a solid state polar crystal.[20] The starting geometries of all compounds were obtained from crystal structure geometries. Counterions and solvent molecules that are not directly bonded to the metal center were deleted. **Table S28** below summarizes the Cambridge Crystallographic Database Centre (CCDC) codes for the crystal structures used for each compound. $Cu(dtc)_2$ crystallizes as a staggered dimer with an axial coordination between the sulfur of one compound to the copper center of another. One of these compounds was deleted from the structure and $Cu(dtc)_2$ was modeled as a monomer, whose optimized geometry was close to square planar. In some cases, the geometry optimization resulted in a distortion of the metal center geometry away from square planar. It was found that the distorted structures have a worse match for the experimental spectra, suggesting the distortion arises from an incomplete modelling of packing forces. In these cases, a constrained optimization was performed, in which two angles were constrained to 180 degrees between the ligating atoms across from each other, through the Cu center (S–Cu–S, for example). Time-dependent density functional theory (TDDFT) calculations were used to predict the excited state energies and UV-vis-NIR absorption spectrum. The def2-TZVP and def2/J basis sets were assigned to all atoms. All calculations employed the RIJCOSX approximation, TIGHTSCF convergence criteria, SlowConv convergence strategy, and D3BJ dispersion correction. Detailed information on the calculated absorption spectrum for each compound, including natural transition donor and acceptor orbitals, is shown below.

Table S28: Crystal structures used as starting geometries.

Compound	CCDC Identifier	CCDC Deposition Number
$K_2Cu(ox)_2$	RIZQIA	155318
$K_2Cu(ox)_2\ (H_2O)_2$	KCUOXD02	754838
$(PPN)_2Cu(ox)_2$	*	2383406
$Cu(acac)_2$	ACACCU	1100332
$Cu(tmhd)_2$	DERNOD06	1010705
$Cu(hfac)_2$	BAMGAY	201577
$Cu(hfac)_2\ (H_2O)$	ZEJKII01	1310696
$Cu(hfac)_2\ (H_2O)_2$	BAMGIG	201579
$Cu(tbaa)_2$	ZUHDOV	126562
$Cu(tbaa)_2\ (H_2O)$	ZUHDOV	126562
Cu(acacen)	ACIMCU10	1100734
$Cu(pci)_2$	PYALCU	1240391
$Cu(mnt)_2$	DUHSOO	150703
$Cu(dtc)_2$	CETCAM01	111991
$Cu(bdt)_2$	KODYAD	1198543

*not yet assigned at time of publication

Table S29: TDDFT-calculated excited state d–d transition energies for all compounds.

**Compound**	***d–d* Transition Energies (cm^{-1})**			
$K_2[Cu(ox)_2]$	16849.2 ($z^2/x^2–y^2$)	17717.9 (xz)	17823.2 (yz)	19152.9 ($z^2/x^2–y^2$)
$K_2[Cu(ox)_2]$•$2H_2O$	14290.3 (z^2)	16692.3 (xz)	16868.3 (yz)	17567.7 ($x^2–y^2$)
$(PPN)_2[Cu(ox)_2]$	16875.9 ($z^2/x^2–y^2$)	17727.1 (xz)	17831.1 (yz)	19161.7 ($z^2/x^2–y^2$)
$Cu(acac)_2$	15717.5 (xz)	17406.7 ($z^2/x^2–y^2$)	19221.6 ($z^2/x^2–y^2$)	19451.6 (yz)
$Cu(tmhd)_2$	15803.4 (xz)	17571.6 ($z^2/x^2–y^2$)	19549.4 ($z^2/x^2–y^2$)	19808.1 (yz)
$Cu(hfac)_2$	14616.1 (xz)	15917.7 (z^2)	17959.9 ($x^2–y^2$)	18377 (yz)
$Cu(hfac)_2$•H_2O	11705.1 (z^2)	13809.4 (xz)	16716.5 ($x^2–y^2$)	16785 (yz)
$Cu(hfac)_2$•$2H_2O$	9043.4 (z^2)	13238.6 (xz)	15893.8 (yz)	16209.8 ($x^2–y^2$)
$Cu(tbaa)_2$	14405.7 (xz)	16456.0 (z^2/ $x^2–y^2$)	17471.4 (yz)	17542.5 (z^2/ $x^2–y^2$)
$Cu(tbaa)_2$•H_2O	14284.3 (z^2)	14537.1 (xz)	17051.8 (yz/ $x^2–y^2$)	17542.5 (yz/ $x^2–y^2$)
Cu(acacen)	15846.8 (xz)	20687.1 (z^2)	21573.9 (yz)	21923.7 ($x^2–y^2$)
Cu(acacen) (Const. Opt.)	16255.3 (xz)	21106.2 (z^2)	21991.2 (yz)	22409.4 ($x^2–y^2$)
$Cu(pci)_2$	17484.9 (yz)	18961.7 (xz)	20233 (z^2)	22457.6 ($x^2–y^2$)
$[Cu(mnt)_2]^{2–}$	7700.6 (xz)	17048.8 (yz)	18012.4 (z^2)	21118.1 ($x^2–y^2$)
$[Cu(mnt)_2]^{2–}$ (Const. Opt.)	8288.8 (xz)	18362.1 (yz)	19618.9 (z^2)	22960.7 ($x^2–y^2$)
$Cu(dtc)_2$	15643.3 (yz)	17622.5 (xz)	20320.3 (z^2)	22856.7 ($x^2–y^2$)
$Cu(dtc)_2$ (Const. Opt.)	15654.5 (yz)	17715.6 (xz)	20242.4 (z^2)	22852.6 ($x^2–y^2$)
$[Cu(bdt)_2]^{2–}$	10080.2 (xz)	16196.7 (yz)	21314.8 (z^2)	23813.2 ($x^2–y^2$)
$[Cu(bdt)_2]^{2–}$ (Const. Opt.)	10488.6 (xz)	16820.3 (yz)	21166.7 (z^2)	23528.1 ($x^2–y^2$)

Table S30: Comparison of the average d–d transition energies as calculated by TDDFT and as measured by MCD spectroscopy.

Compound	Average *d–d* Transition Energy (cm^{-1})	
	Measured	Calculated
$K_2[Cu(ox)_2]$	15253	16355
$PPN_2[Cu(ox)_2]$	16235	17899
$Cu(hfac)_2$	15234	14754
$Cu(tmhd)_2$	16608	18183
$Cu(tbaa)_2$	16176	15854
$Cu(acac)_2$	16824	17949
$Cu(pci)_2$	18166	19784
Cu(acacen)	17849	20441
$Cu(dtc)_2$	18795	19116
$[Cu(mnt)_2]^{2-}$	17115	15970
$[Cu(bdt)_2]^{2-}$	20415	18001

Table S31: Properties used to evaluate covalency. Tabulated average copper–ligand bond lengths in optimized geometries and Loewdin density of excited state acceptor d(xy) orbital.

Compound	Average Cu-L Bond Length (Å)	Loewdin Density of Acceptor *d*(xy) Orbital
$K_2[Cu(ox)_2]$	1.952	66.3%
$K_2[Cu(ox)_2]•2H_2O$	1.967	64.8%
$(PPN)_2[Cu(ox)_2]$	1.952	66.5%
$Cu(acac)_2$	1.945	65.8%
$Cu(tmhd)_2$	1.936	65.4%
$Cu(hfac)_2$	1.947	68.2%
$Cu(hfac)_2•H_2O$	1.965	68.6%
$Cu(hfac)_2•2H_2O$	1.980	69.8%
$Cu(tbaa)_2$	1.941	66.7%
$Cu(tbaa)_2•H_2O$	1.954	68.1%
Cu(acacen)	1.956	61.8%
Cu(acacen) (constrained)	1.953	61.7%
$Cu(pci)_2$	2.002	64.3%
$[Cu(mnt)_2]^{2-}$	2.309	38.9%
$[Cu(mnt)_2]^{2-}$ (constrained)	2.324	41.3%
$Cu(dtc)_2$	2.347	40.0%
$Cu(dtc)_2$ (constrained)	2.348	40.6%
$[Cu(bdt)_2]^{2-}$	2.316	40.8%
$[Cu(bdt)_2]^{2-}$ (constrained)	2.323	41.8%

7.1.1 $K_2[Cu(ox)_2]$

Table S32: TDDFT-calculated $K_2[Cu(ox)_2]$ excited states assigned to d-d transitions.

State	NTO Occupation Number	Energy (cm^{-1})	Assignment
1	0.99719415	16849.2	$z^2/x^2–y^2 \rightarrow xy$
2	0.99863175	17717.9	$xz \rightarrow xy$
3	0.99883231	17823.2	$yz \rightarrow xy$
4	0.99868532	19152.9	$z^2/x^2–y^2 \rightarrow xy$

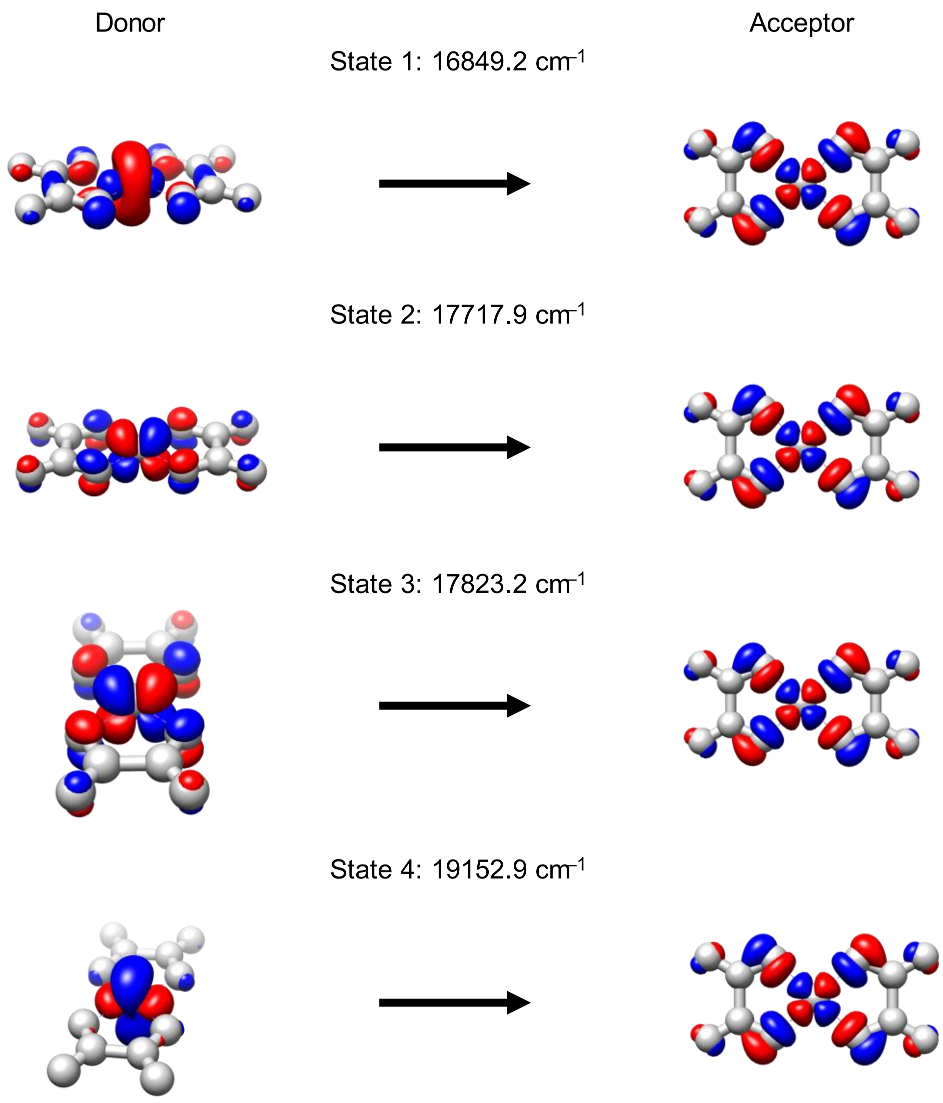

Figure S122: TDDFT natural transition orbitals for $K_2[Cu(ox)_2]$.

Table S33: TDDFT-calculated $K_2[Cu(ox)_2]$ UV-vis-NIR absorption spectrum.

Calculated via transition electric dipole moments, including oscillator strength (f_{osc}), net squared transition electric dipole moment (T^2), and transition electric dipole moment vector components.

State	Energy (cm^{-1})	Wavelength (nm)	f_{osc}	T^2 (a.u.^{2})	T_x (a.u.)	T_y (a.u.)	T_z (a.u.)
1	16849.2	593.5	0	0	0	0	0
2	17717.9	564.4	0	0	0	0	0
3	17823.2	561.1	0	0	0	0	0
4	19152.9	522.1	0	0	0	0	0
5	26652.4	375.2	0.000012446	0.00015	0.0006	-0.01231	0.00134
6	29508.1	338.9	0	0	0	0	0
7	30568.9	327.1	0.283624438	3.0545	-1.74533	0.00434	-0.09105
8	32097.6	311.5	0	0	0.00002	0	-0.00002
9	32100	311.5	0.000007846	0.00008	-0.00587	0.00068	0.00675
10	33046	302.6	0.000256526	0.00256	0.04935	0.00089	-0.01094
11	33089.6	302.2	0.000010018	0.0001	0.00995	-0.00057	0.00053
12	33911.6	294.9	0	0	-0.00001	0	0
13	36218.2	276.1	0	0	0	0	0
14	36449.3	274.4	0.000921965	0.00833	0.00562	-0.00257	-0.09104
15	36498	274	0	0	0	0.00001	0.00002
16	37038	270	0	0	0	0.00001	0
17	37606.7	265.9	0.000030731	0.00027	0.01526	-0.00593	0.00096
18	38230.2	261.6	0.003460617	0.0298	-0.00023	-0.17261	-0.00243
19	38338.5	260.8	0	0	0	-0.00002	0
20	38482.6	259.9	0.000000004	0	-0.00001	-0.00019	0
21	38551.7	259.4	0.066491517	0.5678	-0.0001	0.75345	0.01076
22	38798.7	257.7	0	0	0.00001	0	0
23	39305.9	254.4	0.000017261	0.00014	-0.012	-0.0004	-0.00063
24	40701.3	245.7	0.00046722	0.00378	-0.06141	0.00021	-0.00284
25	41098.7	243.3	0	0	0	0	0

7.1.2 $K_2[Cu(ox)_2]\bullet2H_2O$

Table S34: TDDFT-calculated $K_2[Cu(ox)_2]\bullet2H_2O$ excited states assigned to d-d transitions.

State	NTO Occupation Number	Energy (cm^{-1})	Assignment
1	0.99733159	14290.3	$z^2 \rightarrow xy$
2	0.99884998	16692.3	$xz \rightarrow xy$
3	0.99899547	16868.3	$yz \rightarrow xy$
4	0.99932226	17567.7	$x^2–y^2 \rightarrow xy$

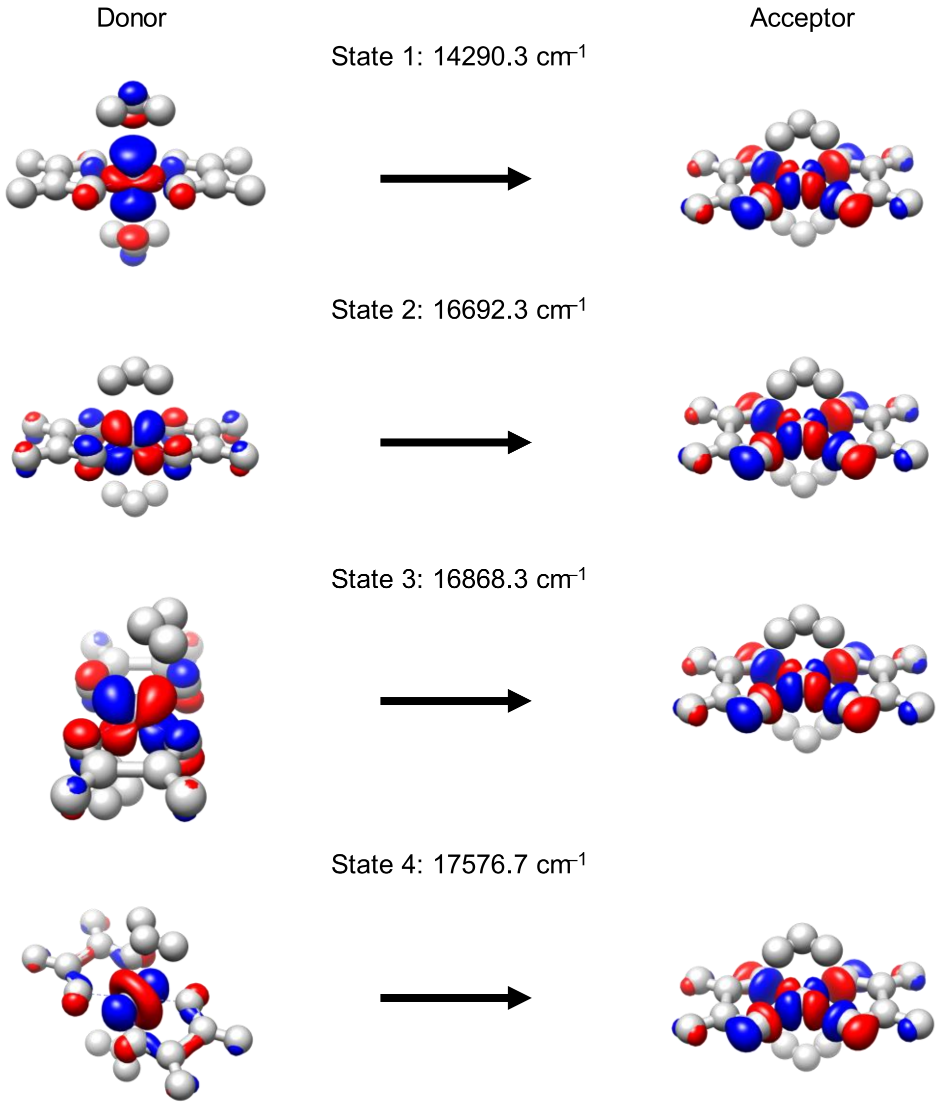

Figure S123: TDDFT natural transition orbitals for $K_2[Cu(ox)_2]\bullet2H_2O$.

Table S35: TDDFT-calculated $K_2[Cu(ox)_2]$•$2H_2O$ UV-vis-NIR absorption spectrum.

Calculated via transition electric dipole moments, including oscillator strength (f_{osc}), net squared transition electric dipole moment (T^2), and transition electric dipole moment vector components.

State	Energy (cm^{-1})	Wavelength (nm)	f_{osc}	T^2 ($a.u.^2$)	T_x (a.u.)	T_y (a.u.)	T_z (a.u.)
1	14290.3	699.8	0	0	0.00003	0	0
2	16692.3	599.1	0	0	0.00001	0.00006	-0.00001
3	16868.3	592.8	0.000000002	0	-0.00019	0	0
4	17567.7	569.2	0	0	0.00005	0	0
5	26875.7	372.1	0.000029178	0.00036	-0.00053	-0.01744	0.00727
6	29473.1	339.3	0.000000002	0	-0.00014	0	0
7	30551.6	327.3	0.269785396	2.9071	-1.70479	0.01505	0.02351
8	32170.7	310.8	0.000000002	0	-0.00012	0.00002	0.00003
9	32175.6	310.8	0.000005616	0.00006	0.00086	0.00367	0.00658
10	33268.8	300.6	0.000069561	0.00069	-0.02623	0.00009	0.00072
11	33346.2	299.9	0.000065037	0.00064	-0.02054	-0.00577	-0.01367
12	33997.5	294.1	0	0	-0.00001	-0.00001	0
13	35907	278.5	0.000000003	0	0.00015	0.00001	0
14	36442.9	274.4	0.000387577	0.0035	0.05916	0.00082	-0.00067
15	36508.5	273.9	0.000000033	0	-0.00013	-0.00018	-0.0005
16	36512.1	273.9	0.000842417	0.0076	0.00201	0.02772	0.0826
17	37149.3	269.2	0.000000002	0	0.00001	-0.00013	-0.00001
18	38242.1	261.5	0.00176257	0.01517	0.00038	0.12007	-0.0275
19	38347.4	260.8	0	0	0	0.00004	-0.00001
20	38389.2	260.5	0.000000184	0	-0.00004	-0.00122	0.00028
21	38669.5	258.6	0.063819106	0.54332	0.00306	0.71925	-0.16121
22	38734.5	258.2	0.000000157	0	0.00003	-0.00113	0.00025
23	39178.6	255.2	0.000075574	0.00064	0.0252	-0.00009	-0.00041
24	40496.9	246.9	0.000428324	0.00348	-0.059	0.00049	0.00063
25	40795.2	245.1	0	0	0	0	-0.00001

7.1.3 $(PPN)_2[Cu(ox)_2]$

Table S36: TDDFT-calculated $(PPN)_2[Cu(ox)_2]$ excited states assigned to d-d transitions.

State	NTO Occupation Number	Energy (cm^{-1})	Assignment
1	0.99719374	16875.9	$z^2/x^2–y^2 \rightarrow xy$
2	0.99865662	17727.1	$xz \rightarrow xy$
3	0.99884403	17831.1	$yz \rightarrow xy$
4	0.99868671	19161.7	$z^2/x^2–y^2 \rightarrow xy$

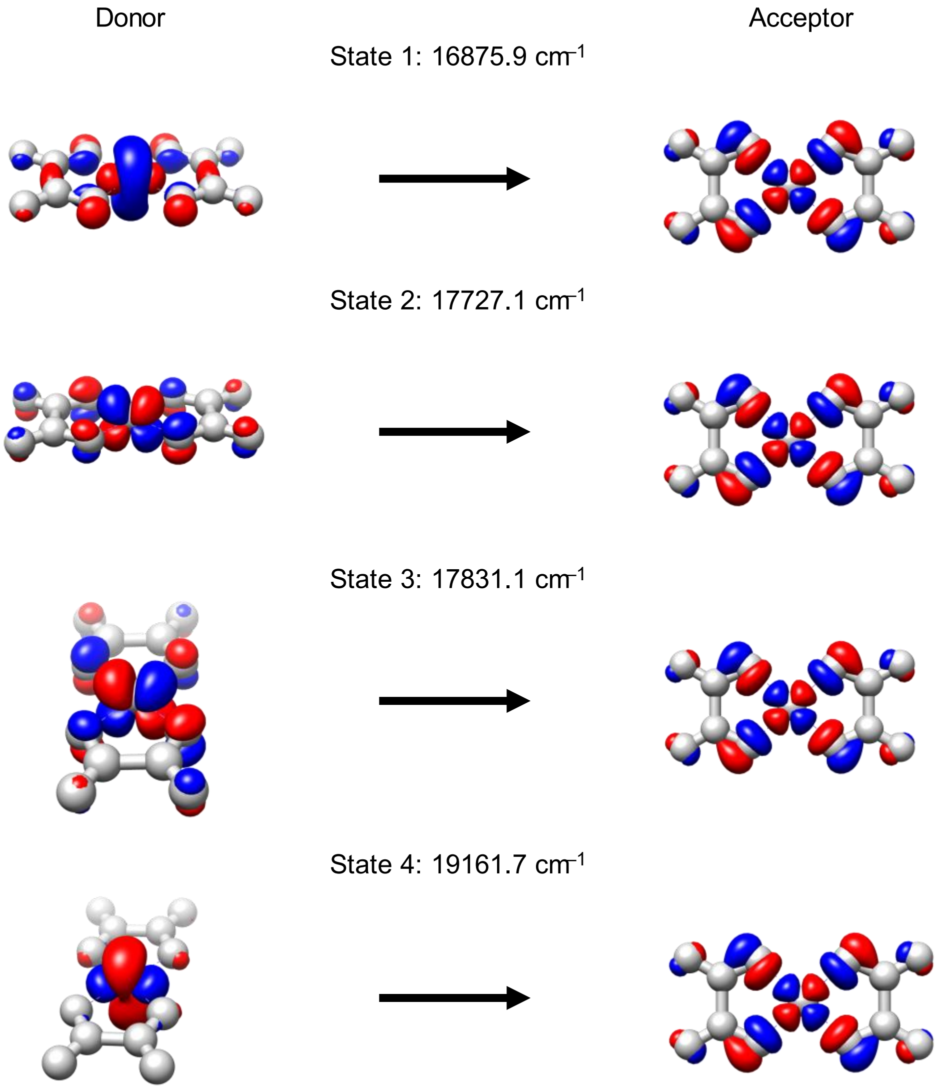

Figure S124: TDDFT natural transition orbitals for $(PPN)_2[Cu(ox)_2]$.

Table S37: TDDFT-calculated $(PPN)_2[Cu(ox)_2]$ UV-vis-NIR absorption spectrum.

Calculated via transition electric dipole moments, including oscillator strength (f_{osc}), net squared transition electric dipole moment (T^2), and transition electric dipole moment vector components.

State	Energy (cm^{-1})	Wavelength (nm)	f_{osc}	T^2 (a.u.^{2})	T_x (a.u.)	T_y (a.u.)	T_z (a.u.)
1	16875.9	592.6	0	0	0	0	0
2	17727.1	564.1	0	0	0	0	0
3	17831.1	560.8	0	0	0	0	0
4	19161.7	521.9	0	0	0	0	0
5	26677.8	374.8	0.000016007	0.0002	0.00022	-0.01405	0.00031
6	29534.4	338.6	0	0	-0.00001	0	0
7	30629	326.5	0.2832564	3.04455	1.74476	0.00687	0.0182
8	32096.3	311.6	0	0	-0.00001	0	0.00002
9	32100.7	311.5	0.000004685	0.00005	0.00075	-0.00014	-0.00689
10	33089.4	302.2	0.000019166	0.00019	-0.00486	-0.00024	0.01292
11	33094.3	302.2	0.000002321	0.00002	-0.00341	-0.00003	0.00339
12	33917.3	294.8	0	0	0	0	0
13	36254.7	275.8	0	0	0	0	0
14	36451.4	274.3	0.000926676	0.00837	-0.00102	0.00077	0.09147
15	36520.3	273.8	0	0	0	0	-0.00001
16	37067.3	269.8	0	0	0	-0.00002	0
17	37657.2	265.6	0.000001424	0.00001	-0.00311	0.00141	0.00088
18	38244.9	261.5	0.002949798	0.02539	-0.00005	0.15935	-0.0001
19	38353.4	260.7	0	0	0	0.00003	0
20	38522.3	259.6	0.000000001	0	0.00001	-0.0001	0
21	38592.3	259.1	0.067018412	0.5717	0.00062	-0.75611	0.00045
22	38814.1	257.6	0	0	0.00001	0	0
23	39319.9	254.3	0.000000697	0.00001	0.00241	0.00014	0.00009
24	40712.7	245.6	0.000476614	0.00385	0.06208	0.0002	0.0006
25	41111	243.2	0	0	0	0	0

7.1.4 Cu(acac)₂

Table S38: TDDFT-calculated $Cu(acac)_2$ excited states assigned to d-d transitions.

State	NTO Occupation Number	Energy (cm^{-1})	Assignment
1	0.99861423	15717.5	xz → xy
2	0.99376938	17406.7	$z^2/x^2–y^2$ → xy
3	0.99870922	19221.6	$z^2/x^2–y^2$ → xy
4	0.99698818	19451.6	yz → xy

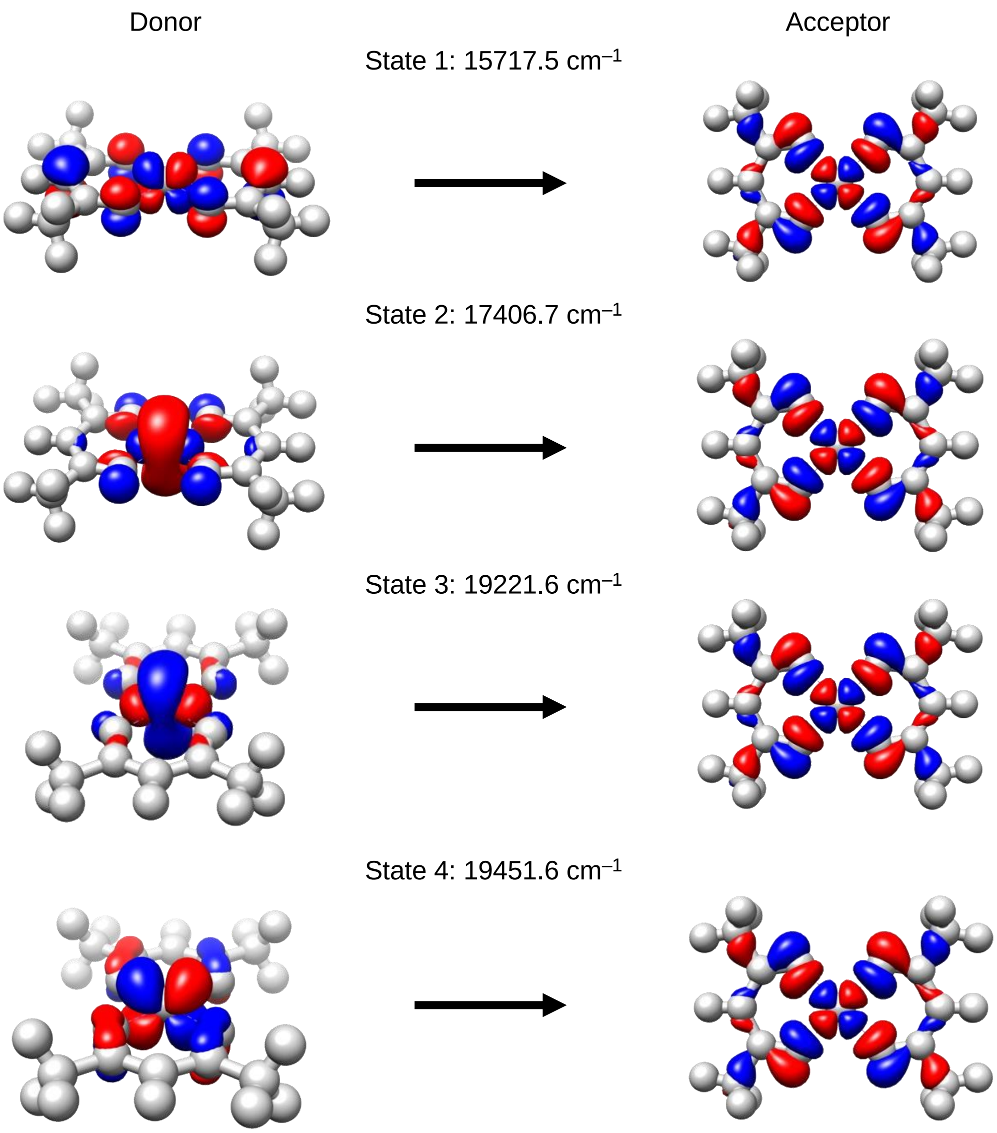

Figure S125: TDDFT natural transition orbitals for $Cu(acac)_2$.

Table S39: TDDFT-calculated $Cu(acac)_2$ UV-vis-NIR absorption spectrum.

Calculated via transition electric dipole moments, including oscillator strength (f_{osc}), net squared transition electric dipole moment (T^2), and transition electric dipole moment vector components.

State	Energy (cm^{-1})	Wavelength (nm)	f_{osc}	T^2 (a.u.^{2})	T_x (a.u.)	T_y (a.u.)	T_z (a.u.)
1	15717.5	636.2	0	0	0.00001	0	0
2	17406.7	574.5	0	0	0	0	0
3	19221.6	520.2	0	0	0	0	0
4	19451.6	514.1	0.000000005	0	-0.00028	0	0
5	23727.4	421.5	0.000001252	0.00002	-0.00077	-0.0041	0.00003
6	25888.8	386.3	0.000000002	0	-0.00001	-0.00014	0
7	26070.7	383.6	0	0	0	0.00002	0
8	26270.7	380.7	0.000016999	0.00021	0.00009	0.01459	0.00012
9	29418.6	339.9	0.310823458	3.4783	1.86487	-0.00256	0.02339
10	30092.6	332.3	0.000000064	0	-0.00084	0	-0.00001
11	30866.1	324	0.000244206	0.0026	0.02	0.00044	-0.04695
12	33188.8	301.3	0.011180977	0.11091	-0.00127	-0.33301	-0.00314
13	35362.4	282.8	0	0	0	-0.00001	-0.00001
14	36599	273.2	0.001543892	0.01389	0.00076	0.11784	0.00118
15	36865.3	271.3	0.001031584	0.00921	0.00067	0.09598	0.00052
16	36931.4	270.8	0	0	0	0.00002	-0.00004
17	36950.9	270.6	0.001449919	0.01292	-0.00971	0.00109	-0.11324
18	37506.2	266.6	0.000000007	0	-0.00025	-0.00001	-0.00001
19	37949.9	263.5	0.000000002	0	-0.00002	0	0.00014
20	38780	257.9	0	0	0	-0.00002	-0.00001
21	41536.6	240.8	0.000000052	0	0	0.00064	0
22	41603.2	240.4	0.001131449	0.00895	-0.00528	0.00074	-0.09447
23	41725.5	239.7	0.000039686	0.00031	0.00009	-0.01769	-0.00031
24	42336.5	236.2	0.000000002	0	-0.00011	-0.00005	0
25	43245.6	231.2	0	0	0	0.00005	-0.00002

7.1.5 Cu(tmhd)$_2$

Table S40: TDDFT-calculated Cu(tmhd)$_2$ excited states assigned to d-d transitions.

State	NTO Occupation Number	Energy (cm^{-1})	Assignment
1	0.99859187	15803.4	xz → xy
2	0.99367031	17571.6	$z^2/x^2–y^2$ → xy
3	0.99834554	19549.4	$z^2/x^2–y^2$ → xy
4	0.99634703	19808.1	yz → xy

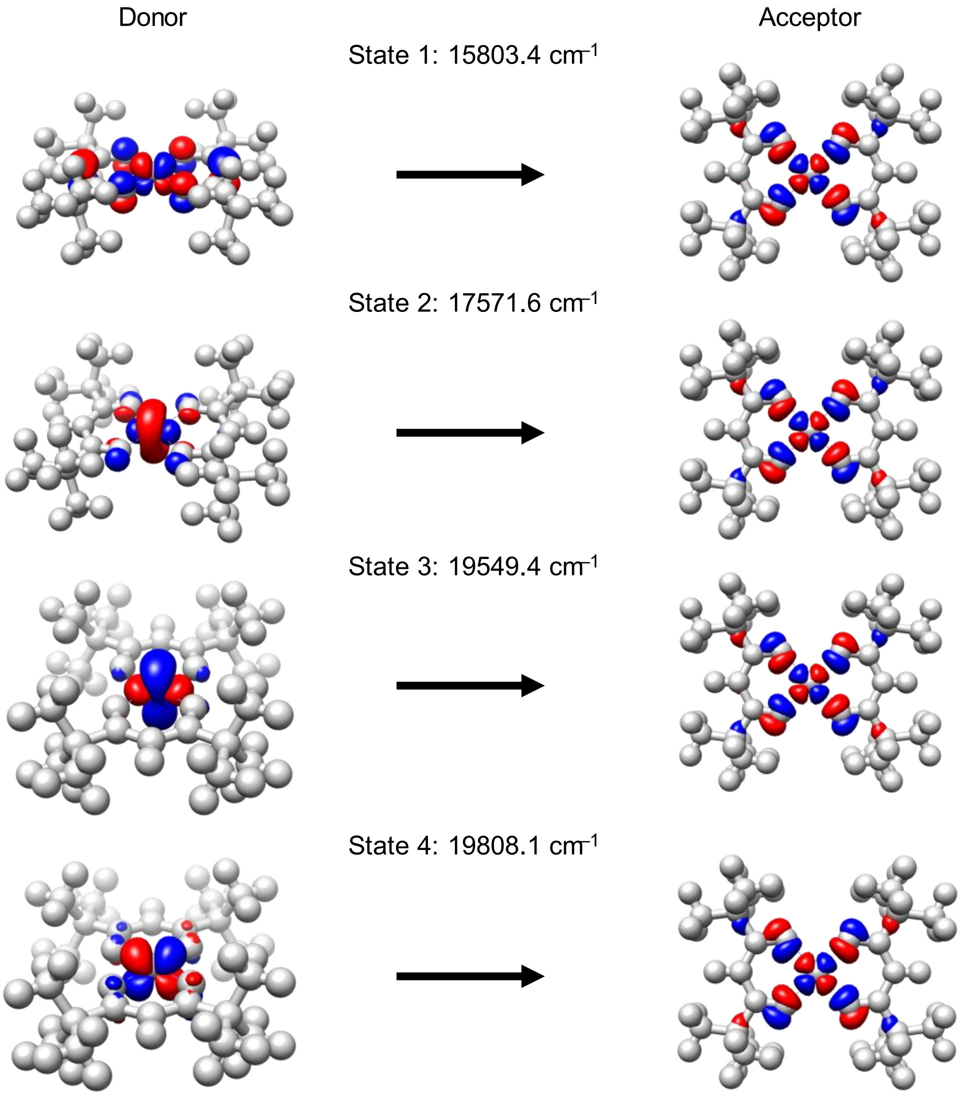

Figure S126: TDDFT natural transition orbitals for Cu(tmhd)$_2$.

Table S41: TDDFT-calculated $Cu(tmhd)_2$ UV-vis-NIR absorption spectrum.

Calculated via transition electric dipole moments, including oscillator strength (f_{osc}), net squared transition electric dipole moment (T^2), and transition electric dipole moment vector components.

State	Energy (cm^{-1})	Wavelength (nm)	f_{osc}	T^2 (a.u.^{2})	T_x (a.u.)	T_y (a.u.)	T_z (a.u.)
1	15803.4	632.8	0.00000384	0.00008	-0.00893	0.00025	0.00039
2	17571.6	569.1	0.000000936	0.00002	0.00072	-0.00005	0.00412
3	19549.4	511.5	0.00000012	0	-0.00069	0.00001	0.00125
4	19808.1	504.8	0.000000181	0	-0.00146	0.00094	0.00004
5	24069.2	415.5	0.000003137	0.00004	0.00226	-0.00615	0.00008
6	25767.9	388.1	0.000001107	0.00001	-0.00376	0.00008	0.00008
7	25975.6	385	0.000021897	0.00028	0.00025	-0.01666	-0.00024
8	26415.2	378.6	0.000791337	0.00986	0.09927	-0.00047	-0.0029
9	28426.8	351.8	0.325191682	3.76606	-1.93972	0.00602	0.05906
10	28997.6	344.9	0.000000093	0	-0.00075	0.00071	-0.00002
11	29717.5	336.5	0.000763931	0.00846	0.03717	-0.00139	0.08414
12	32707.7	305.7	0.006060676	0.061	0.00273	-0.24695	-0.003
13	35043.2	285.4	0.000008745	0.00008	0.0003	-0.00017	0.00906
14	35440	282.2	0.003586557	0.03332	0.01311	0.00165	-0.18205
15	35953.8	278.1	0.000004871	0.00004	-0.00012	-0.00668	0.00005
16	36334.7	275.2	0.003585408	0.03249	0.00071	-0.18023	-0.00136
17	36563.2	273.5	0.000000168	0	0.00003	0.00103	-0.00067
18	36616.9	273.1	0.002001955	0.018	0.00065	-0.13415	-0.00178
19	36695.9	272.5	0.00000042	0	-0.00003	0.00002	0.00194
20	38274.6	261.3	0.000008399	0.00007	-0.00096	0.00013	-0.00844
21	39814.9	251.2	0.002709838	0.02241	-0.00659	-0.00222	0.14953
22	40268.3	248.3	0.000001184	0.00001	-0.00298	-0.00064	0.00062
23	40400.9	247.5	0.00003255	0.00027	0.00004	0.01629	0.00017
24	40508.7	246.9	0.000145045	0.00118	-0.00024	-0.03433	-0.0003
25	42383.5	235.9	0.000124583	0.00097	-0.00104	0.00212	-0.03102

7.1.6 Cu(hfac)$_2$

Table S42: TDDFT-calculated Cu(hfac)$_2$ excited states assigned to d-d transitions.

State	NTO Occupation Number	Energy (cm^{-1})	Assignment
1	0.99859223	14616.1	xz → xy
2	0.99292093	15917.7	z^2 → xy
3	0.99931804	17959.9	x^2–y^2 → xy
4	0.99663381	18377	yz → xy

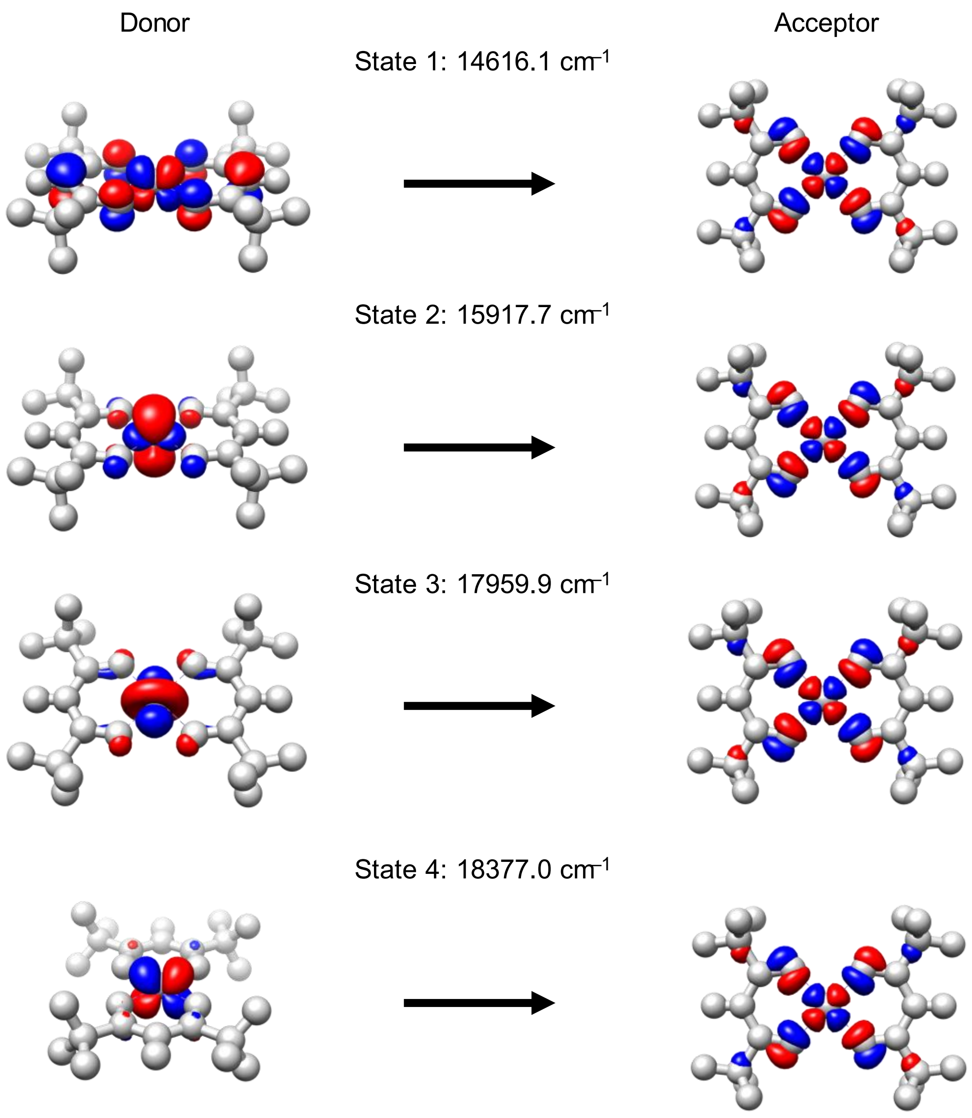

Figure S127: TDDFT natural transition orbitals for Cu(hfac)$_2$.

Table S43: TDDFT-calculated $Cu(hfac)_2$ UV-vis-NIR absorption spectrum.

Calculated via transition electric dipole moments, including oscillator strength (f_{osc}), net squared transition electric dipole moment (T^2), and transition electric dipole moment vector components.

State	Energy (cm^{-1})	Wavelength (nm)	f_{osc}	T^2 (a.u.^{2})	T_x (a.u.)	T_y (a.u.)	T_z (a.u.)
1	14616.1	684.2	0.000000004	0	-0.00031	0	-0.00001
2	15917.7	628.2	0.000000004	0	0	-0.00001	0.0003
3	17959.9	556.8	0	0	0.00007	-0.00001	0.00001
4	18377	544.2	0.000000003	0	0.00023	-0.00009	0.00001
5	22534	443.8	0	0	0.00004	0	0
6	22694.2	440.6	0.000010342	0.00015	0.00103	-0.0122	-0.00047
7	23426.5	426.9	0.000044753	0.00063	-0.02478	0.00375	-0.00102
8	25491.2	392.3	0.000001515	0.00002	-0.00442	0.00008	-0.00014
9	28106.9	355.8	0.000000008	0	0.0001	0.00029	0
10	28714	348.3	0.000231188	0.00265	0.00996	0.00702	-0.05002
11	30566.2	327.2	0.293904314	3.16548	1.7783	-0.00166	0.05582
12	32706.3	305.8	0.000000076	0	-0.00002	0.00035	0.0008
13	32899.6	304	0.026148257	0.26165	0.01329	-0.51119	-0.01287
14	33624.1	297.4	0.00129441	0.01267	0.00166	-0.11256	-0.00055
15	33766.7	296.1	0.000000007	0	0	-0.00024	0.00009
16	34212.1	292.3	0.012149904	0.11691	0.00288	-0.34187	-0.00577
17	35038.9	285.4	0.001513035	0.01422	0.02188	0.0025	-0.11718
18	35403.6	282.5	0.000000011	0	-0.00023	-0.00023	-0.00002
19	37597.1	266	0.00000023	0	0.00001	-0.00001	-0.00142
20	38416.8	260.3	0.000000005	0	-0.00007	-0.00005	0.00018
21	38734.9	258.2	0.000000033	0	-0.00048	-0.00022	0
22	39020	256.3	0.00001549	0.00013	0.00523	0.01013	0.00079
23	39584.6	252.6	0.00113588	0.00945	-0.01068	-0.00606	0.09642
24	40149.5	249.1	0.000000666	0.00001	0.00007	-0.00233	-0.0001
25	41622.9	240.3	0.000000065	0	-0.00045	-0.00055	-0.00003

7.1.7 Cu(hfac)$_2$•H$_2$O

Table S44: TDDFT-calculated Cu(hfac)$_2$•H$_2$O excited states assigned to d-d transitions.

State	NTO Occupation Number	Energy (cm^{-1})	Assignment
1	0.99448698	11705.1	$z^2 \rightarrow xy$
2	0.99803301	13809.4	$xz \rightarrow xy$
3	0.99849440	16716.5	$x^2–y^2 \rightarrow xy$
4	0.99761619	16785	$yz \rightarrow xy$

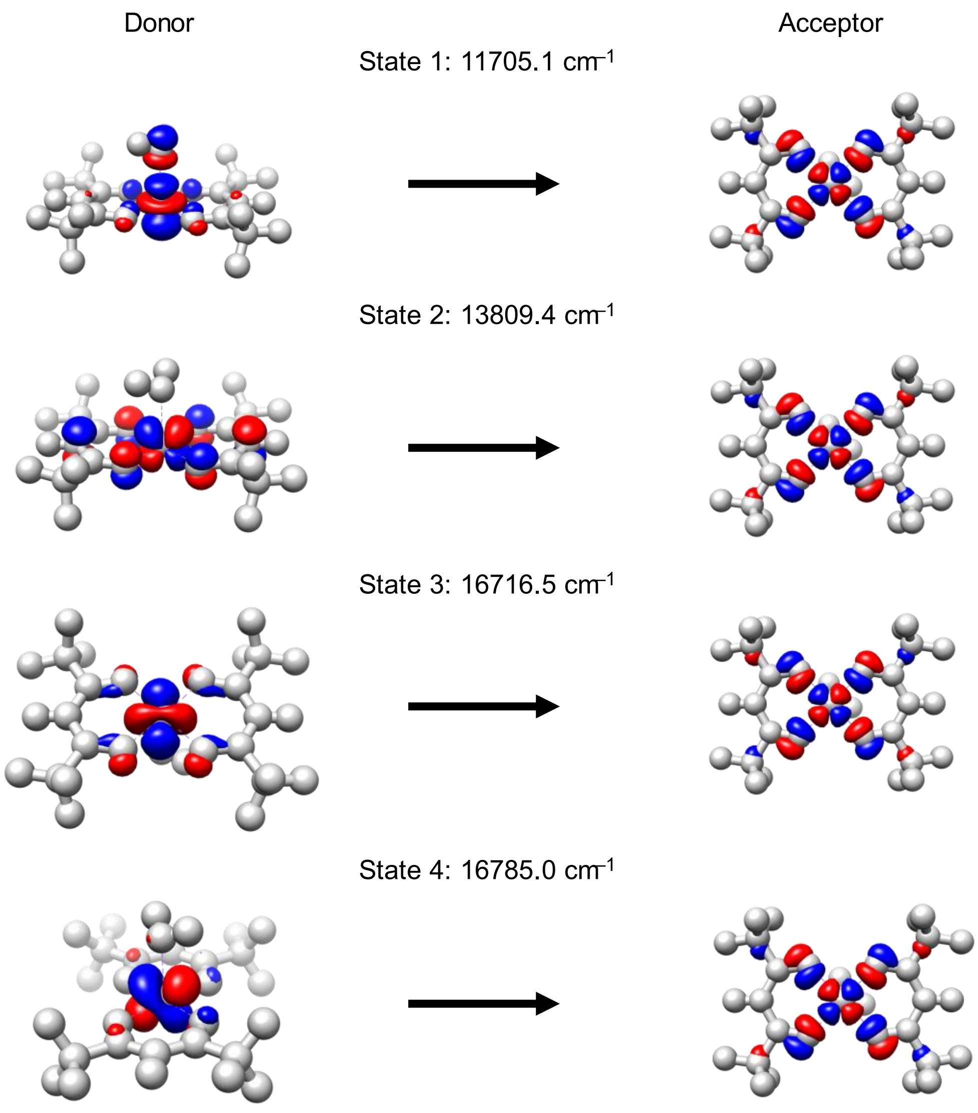

Figure S128: TDDFT natural transition orbitals for Cu(hfac)$_2$•H$_2$O.

Table S45: TDDFT-calculated Cu(hfac)$_2$•H_2O UV-vis-NIR absorption spectrum.

Calculated via transition electric dipole moments, including oscillator strength (f_{osc}), net squared transition electric dipole moment (T^2), and transition electric dipole moment vector components.

State	Energy (cm^{-1})	Wavelength (nm)	f_{osc}	T^2 (a.u.^{2})	T_x (a.u.)	T_y (a.u.)	T_z (a.u.)
1	11705.1	854.3	0.000004176	0.00012	-0.00598	-0.00214	-0.00878
2	13809.4	724.1	0.000106403	0.00254	0.01621	0.04766	0.00166
3	16716.5	598.2	0.000056722	0.00112	0.03341	0.00067	-0.00025
4	16785	595.8	0.000439323	0.00862	0.09276	0.00058	0.0035
5	22256	449.3	0.000000519	0.00001	-0.00154	0.00228	0.00033
6	22435	445.7	0.000024123	0.00035	0.00782	-0.01711	0.0001
7	23697.9	422	0.000001601	0.00002	-0.00343	0.00322	0.00029
8	24838.1	402.6	0.000529204	0.00701	0.06334	-0.05478	0.00135
9	28227.5	354.3	0.000159989	0.00187	0.04314	0.00208	0.00049
10	28823	346.9	0.000208271	0.00238	0.00519	-0.00022	0.0485
11	30572.2	327.1	0.271648622	2.9252	-1.70971	-0.00928	-0.04471
12	32551.3	307.2	0.000037831	0.00038	-0.0003	0.0138	-0.01386
13	32764.1	305.2	0.002656749	0.02669	0.00648	0.16308	0.00768
14	33103.9	302.1	0.013514753	0.1344	-0.00222	-0.36652	-0.00775
15	33205.6	301.2	0.000359131	0.00356	0.00634	0.05891	0.00708
16	33583.9	297.8	0.027472372	0.2693	-0.00149	-0.51893	-0.00289
17	34766.1	287.6	0.001208584	0.01144	-0.00732	0.00984	-0.10627
18	35268.3	283.5	0.009102183	0.08496	-0.29131	0.00577	-0.00844
19	35983.7	277.9	0.000245427	0.00225	0.02324	0.03319	-0.02457
20	36404.3	274.7	0.00014151	0.00128	-0.02579	0.01869	-0.01628
21	37669.8	265.5	0.000055294	0.00048	-0.001	-0.01151	-0.0187
22	38129.8	262.3	0.000465349	0.00402	0.00272	0.043	-0.04649
23	38708	258.3	0.020489049	0.17426	-0.00692	0.41738	0.00283
24	39188.7	255.2	0.000397354	0.00334	0.00337	-0.05283	-0.02314
25	39439.1	253.6	0.00149457	0.01248	0.00268	-0.05787	0.0955
26	39784	251.4	0.001626236	0.01346	-0.04765	0.10408	0.01878
27	39987.6	250.1	0.002482425	0.02044	0.14141	-0.01858	0.00981
28	41792.5	239.3	0.05669716	0.44662	-0.00996	0.66822	0.00256
29	42239.1	236.7	0.048811659	0.38044	0.00196	0.61623	0.02642
30	43067.9	232.2	0.295979543	2.26248	0.00074	-1.50415	0.00221

7.1.8 *Cu(hfac)$_2$•2H_2O*

Table S46: TDDFT-calculated Cu(hfac)$_2$•2H_2O excited states assigned to d-d transitions.

State	NTO Occupation Number	Energy (cm^{-1})	Assignment
1	0.99495715	9043.4	$z^2 \rightarrow xy$
2	0.99864681	13238.6	$xz \rightarrow xy$
3	0.99789505	15893.8	$yz \rightarrow xy$
4	0.99893877	16209.8	$x^2–y^2 \rightarrow xy$

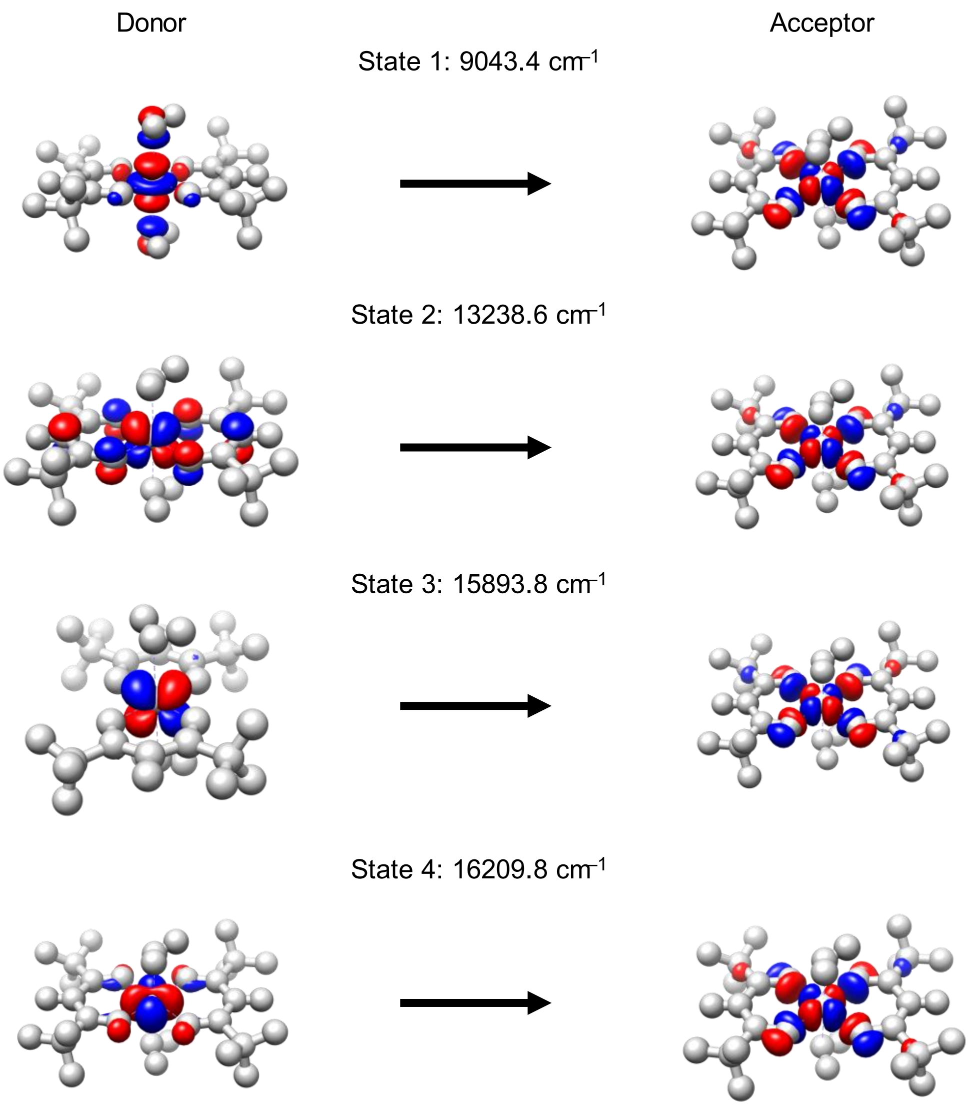

Figure S129: TDDFT natural transition orbitals for Cu(hfac)$_2$•2H_2O.

Table S47: TDDFT-calculated Cu(hfac)$_2$•2H_2O UV-vis-NIR absorption spectrum.

Calculated via transition electric dipole moments, including oscillator strength (f_{osc}), net squared transition electric dipole moment (T^2), and transition electric dipole moment vector components.

State	Energy (cm^{-1})	Wavelength (nm)	f_{osc}	T^2 (a.u.^{2})	T_x (a.u.)	T_y (a.u.)	T_z (a.u.)
1	9043.4	1105.8	0.000002845	0.0001	0.00366	-0.0007	-0.00947
2	13238.6	755.4	0.000020252	0.0005	0.0219	0.00361	-0.00333
3	15893.8	629.2	0.000001245	0.00003	0.00498	-0.00011	0.001
4	16209.8	616.9	0.000002587	0.00005	0.00695	0.00028	-0.00203
5	22364.9	447.1	0.000000906	0.00001	-0.00025	0.00364	0.00005
6	22465.1	445.1	0.000015983	0.00023	0.00232	-0.01499	0.00203
7	23102.8	432.8	0.000031538	0.00045	0.0069	-0.01899	0.00641
8	24623.2	406.1	0.000002557	0.00003	0.00204	-0.0054	-0.00094
9	28560.6	350.1	0.000012433	0.00014	-0.0011	-0.00984	0.00673
10	29108.9	343.5	0.00036299	0.00411	0.04188	-0.0008	-0.04849
11	30927.1	323.3	0.27360177	2.91243	-1.70136	-0.00988	0.13302
12	32609.6	306.7	0.001875794	0.01894	-0.01198	0.13706	0.00285
13	32952.9	303.5	0.002277424	0.02275	-0.00169	0.14988	-0.01691
14	33007.1	303	0.008284287	0.08263	-0.01532	-0.28676	0.0128
15	33161.7	301.6	0.002746026	0.02726	-0.00036	0.16495	-0.0073
16	33483.3	298.7	0.001055933	0.01038	-0.00866	-0.10123	0.00777
17	33850.8	295.4	0.021282903	0.20698	0.01505	-0.4545	0.01377
18	33972.8	294.4	0.00766832	0.07431	0.00276	0.27166	-0.02241
19	35079.9	285.1	0.002025169	0.01901	-0.08029	0.00444	0.11198
20	35391.9	282.6	0.000442552	0.00412	0.05306	-0.00373	-0.03588
21	36028.8	277.6	0.000050585	0.00046	0.00298	0.00581	0.02048
22	36823.2	271.6	0.000218001	0.00195	-0.00684	-0.02596	0.03505
23	36950.5	270.6	0.000031486	0.00028	0.00389	-0.00256	-0.01609
24	38300.4	261.1	0.000038921	0.00033	-0.012	0.00762	-0.01151
25	38456.2	260	0.000068763	0.00059	-0.01123	-0.01419	-0.01616
26	39658.2	252.2	0.001059355	0.00879	0.03428	-0.00737	-0.08697
27	39845.3	251	0.01275223	0.10536	-0.01427	-0.32403	-0.01277
28	40215.6	248.7	0.000216937	0.00178	-0.02053	0.0305	0.02059
29	41398.5	241.6	0.003676539	0.02924	0.00926	-0.17038	0.01103
30	41701.4	239.8	0.002577847	0.02035	-0.00023	-0.14259	0.00422

7.1.9 Cu(tbaa)$_2$

Table S48: TDDFT-calculated Cu(tbaa)$_2$ excited states assigned to d-d transitions.

State	NTO Occupation Number	Energy (cm^{-1})	Assignment
1	0.99865818	14879.7	xz → xy
2	0.99499804	16893.9	$z^2/x^2–y^2$ → xy
3	0.99817756	18137.4	yz → xy
4	0.99891906	18669.0	$z^2/x^2–y^2$ → xy

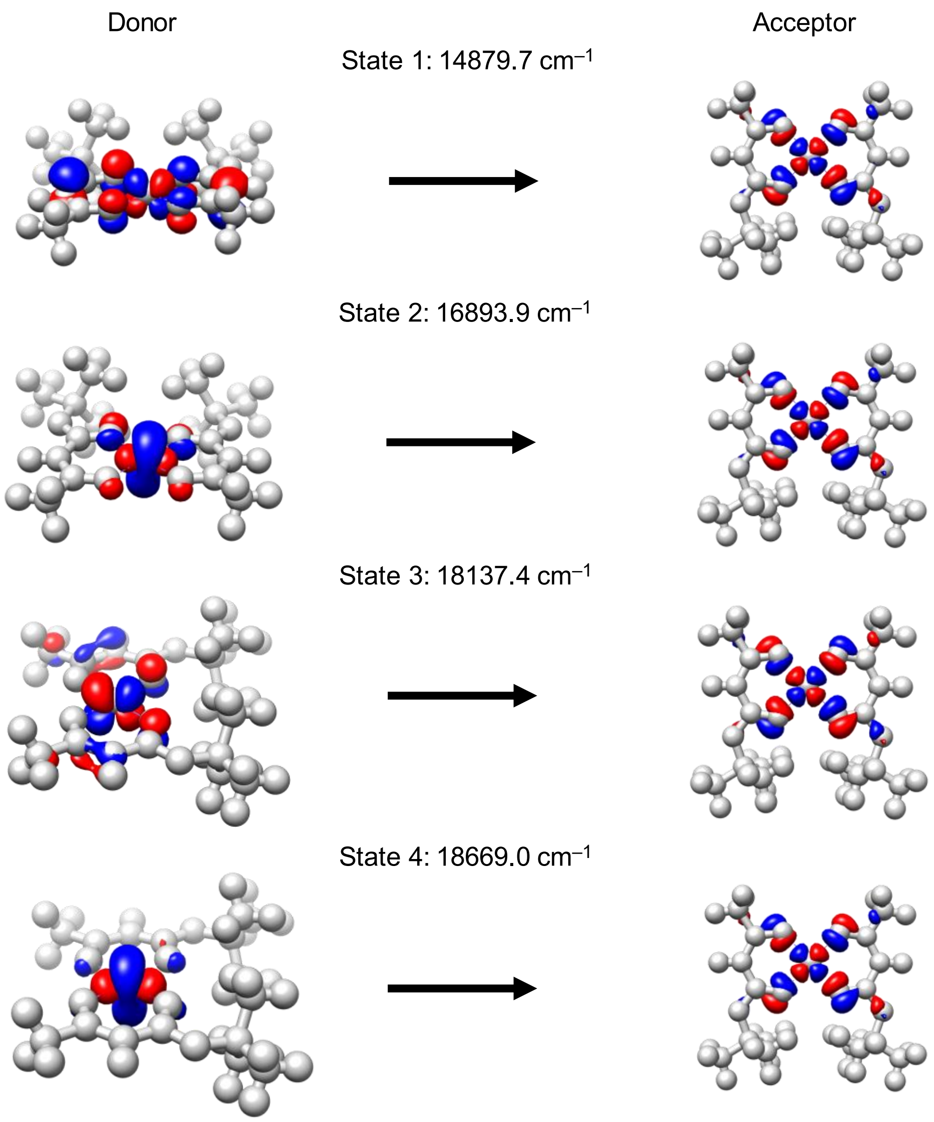

Figure S130: TDDFT natural transition orbitals for Cu(tbaa)$_2$.

Table S49: TDDFT-calculated Cu(tbaa)$_2$ UV-vis-NIR absorption spectrum.

Calculated via transition electric dipole moments, including oscillator strength (f_{osc}), net squared transition electric dipole moment (T^2), and transition electric dipole moment vector components.

State	Energy (cm^{-1})	Wavelength (nm)	f_{osc}	T^2 (a.u.^{2})	T_x (a.u.)	T_y (a.u.)	T_z (a.u.)
1	14879.7	672.1	0.000010112	0.00022	0.01485	-0.00002	-0.00176
2	16893.9	591.9	0.001655843	0.03227	-0.17626	-0.00306	-0.03452
3	18137.4	551.3	0.000000466	0.00001	-0.00006	0.00289	-0.00031
4	18669	535.6	0.000014452	0.00025	-0.01543	0.00012	0.00410
5	21914.2	456.3	0.000002532	0.00004	0.00174	0.00592	0.00003
6	23777.4	420.6	0.00219155	0.03034	-0.17407	0.00007	-0.00658
7	28759.4	347.7	0.0001547	0.00177	0.04193	0.0004	0.00350
8	28917.2	345.8	0.000011857	0.00013	-0.00044	-0.01159	0.00071
9	30444.9	328.5	0.26337075	2.84793	1.68403	0.01325	0.10865
10	33471.5	298.8	0.000104392	0.00103	-0.00037	-0.03202	0.00127
11	36307.3	275.4	0.000028676	0.00026	0.00057	-0.0161	0.00064
12	37086.8	269.6	0.000071476	0.00063	0.01958	0.00263	0.01563
13	37882.5	264	0.000977552	0.0085	0.03203	0.00423	0.08632
14	38919.6	256.9	0.000408324	0.00345	0.00906	0.05775	-0.00608
15	39001.5	256.4	0.010990738	0.09277	0.00225	-0.30393	0.01985
16	39227.3	254.9	0.002092304	0.01756	0.0004	0.1322	-0.00914
17	39440.8	253.5	0.001547	0.01291	0.00147	0.11345	-0.00628
18	39471.7	253.3	0.000082018	0.00068	-0.00584	0.02442	-0.00732
19	39988.2	250.1	0.001706679	0.01405	0.11814	0.00238	0.00932
20	40224.2	248.6	0.001413767	0.01157	0.00042	-0.1074	0.00606
21	42605	234.7	0.000422341	0.00326	0.00903	0.00471	-0.05621
22	43113.5	231.9	0.005778299	0.04412	0.00243	-0.20973	0.01134
23	43525.2	229.8	0.008613155	0.06515	-0.18548	-0.01361	0.17481
24	44228.8	226.1	1.134464208	8.44425	0.00494	-2.90032	0.17998
25	44560	224.4	0.000002222	0.00002	0.00008	0.00403	-0.00038

7.1.10 Cu(tbaa)$_2$•H_2O

Table S50: TDDFT-calculated Cu(tbaa)$_2$•H_2O excited states assigned to d-d transitions.

State	NTO Occupation Number	Energy (cm^{-1})	Assignment
1	0.99560726	14284.3	$z^2 \rightarrow xy$
2	0.99850760	14537.1	$xz \rightarrow xy$
3	0.99829861	17051.8	$yz/x^2{-}y^2 \rightarrow xy$
4	0.99902934	17542.5	$yz/x^2{-}y^2 \rightarrow xy$

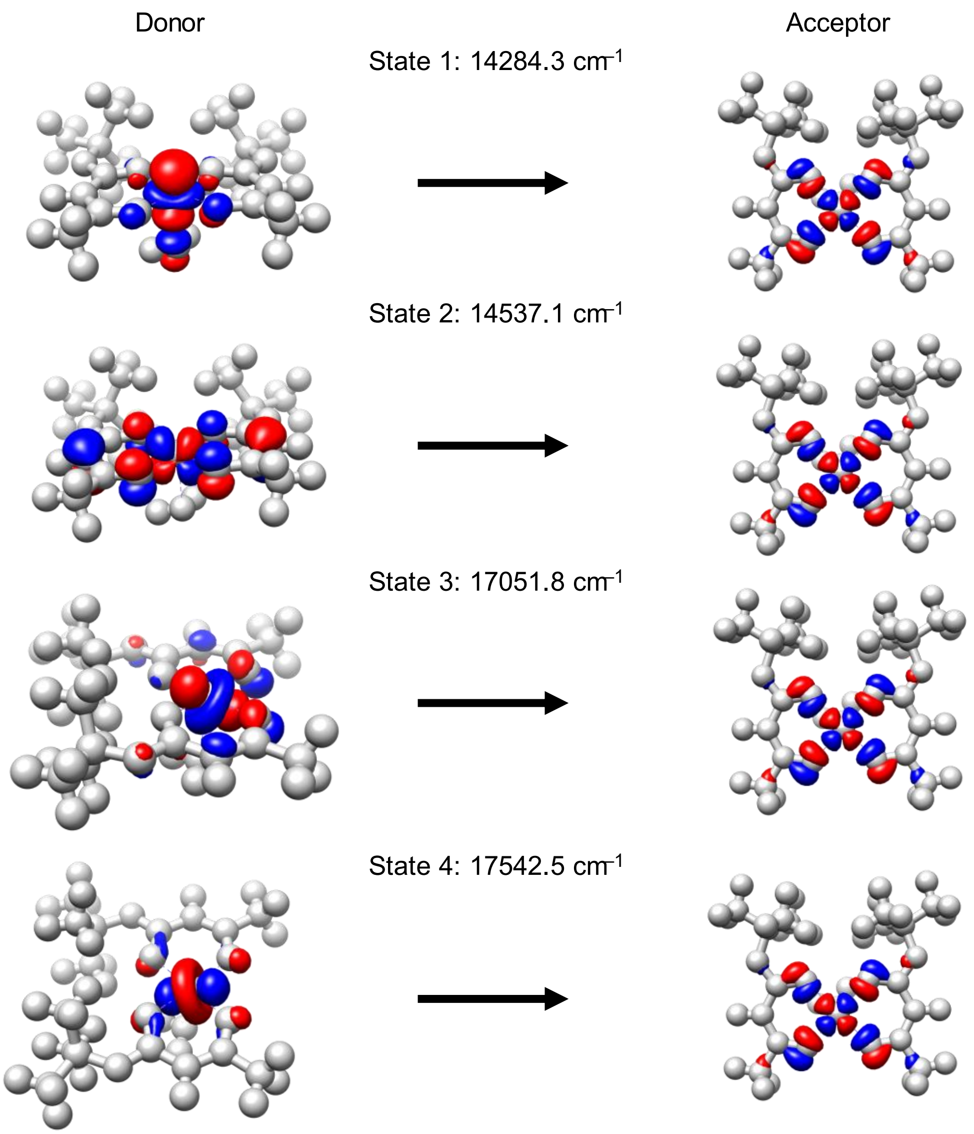

Figure S131: TDDFT natural transition orbitals for Cu(tbaa)$_2$•H_2O.

Table S51: TDDFT-calculated Cu(tbaa)$_2$•H_2O UV-vis-NIR absorption spectrum.

Calculated via transition electric dipole moments, including oscillator strength (f_{osc}), net squared transition electric dipole moment (T^2), and transition electric dipole moment vector components.

State	Energy (cm^{-1})	Wavelength (nm)	f_{osc}	T^2 (a.u.^{2})	T_x (a.u.)	T_y (a.u.)	T_z (a.u.)
1	14284.3	700.1	0.000539267	0.01243	-0.10975	-0.0086	-0.01759
2	14537.1	687.9	0.000125644	0.00285	-0.02618	0.04637	0.00314
3	17051.8	586.4	0.000905766	0.01749	-0.13215	-0.00192	-0.00439
4	17542.5	570	0.000002374	0.00004	0.00649	0.00122	0.00096
5	22243.9	449.6	0.000064635	0.00096	0.03056	-0.00438	-0.00184
6	23428.4	426.8	0.000473182	0.00665	0.07449	0.03277	-0.00517
7	28695.2	348.5	0.000139357	0.0016	0.03997	-0.00054	0.00078
8	28850.6	346.6	0.000006738	0.00008	0.00422	-0.00768	-0.00014
9	30579.2	327	0.255654555	2.75234	-1.65836	-0.04317	-0.01838
10	33612.6	297.5	0.0002608	0.00255	0.00484	-0.05006	-0.00499
11	36256.8	275.8	0.000870371	0.0079	-0.08852	-0.00811	-0.00071
12	37041.2	270	0.000288169	0.00256	0.01866	-0.04391	0.01689
13	37857.2	264.2	0.00035874	0.00312	-0.03643	0.00586	-0.04193
14	38613.8	259	0.008492203	0.0724	-0.01108	0.26811	-0.01989
15	39071.8	255.9	0.000524948	0.00442	-0.00966	0.06575	0.0025
16	39286.2	254.5	0.000018117	0.00015	-0.01193	-0.00242	0.00191
17	39306.6	254.4	0.000012206	0.0001	-0.0088	0.00212	-0.0045
18	39670.2	252.1	0.004471813	0.03711	-0.15415	-0.11522	0.00852
19	39788.8	251.3	0.007630118	0.06313	-0.0621	0.24335	-0.0076
20	39993.3	250	0.000576852	0.00475	-0.00851	-0.06667	0.0152
21	42175.8	237.1	0.000898387	0.00701	-0.00231	0.05891	-0.05947
22	42737	234	0.002904442	0.02237	0.11458	0.09574	-0.00892
23	43432	230.2	0.005247876	0.03978	-0.16868	0.03911	0.09898
24	43914.3	227.7	0.037380076	0.28023	-0.00457	-0.52919	0.01273
25	44042.2	227.1	1.026597174	7.67373	-0.05957	2.76951	0.00144

7.1.11 Cu(acacen)

Table S52: TDDFT-calculated Cu(acacen) excited states assigned to d-d transitions.

State	NTO Occupation Number	Energy (cm^{-1})	Assignment
1	0.99768409	15846.8	xz ⟶ xy
2	0.99142471	20687.1	z^2 ⟶ xy
3	0.99117948	21573.9	yz ⟶ xy
4	0.99766872	21923.7	$x^2–y^2$ ⟶ xy

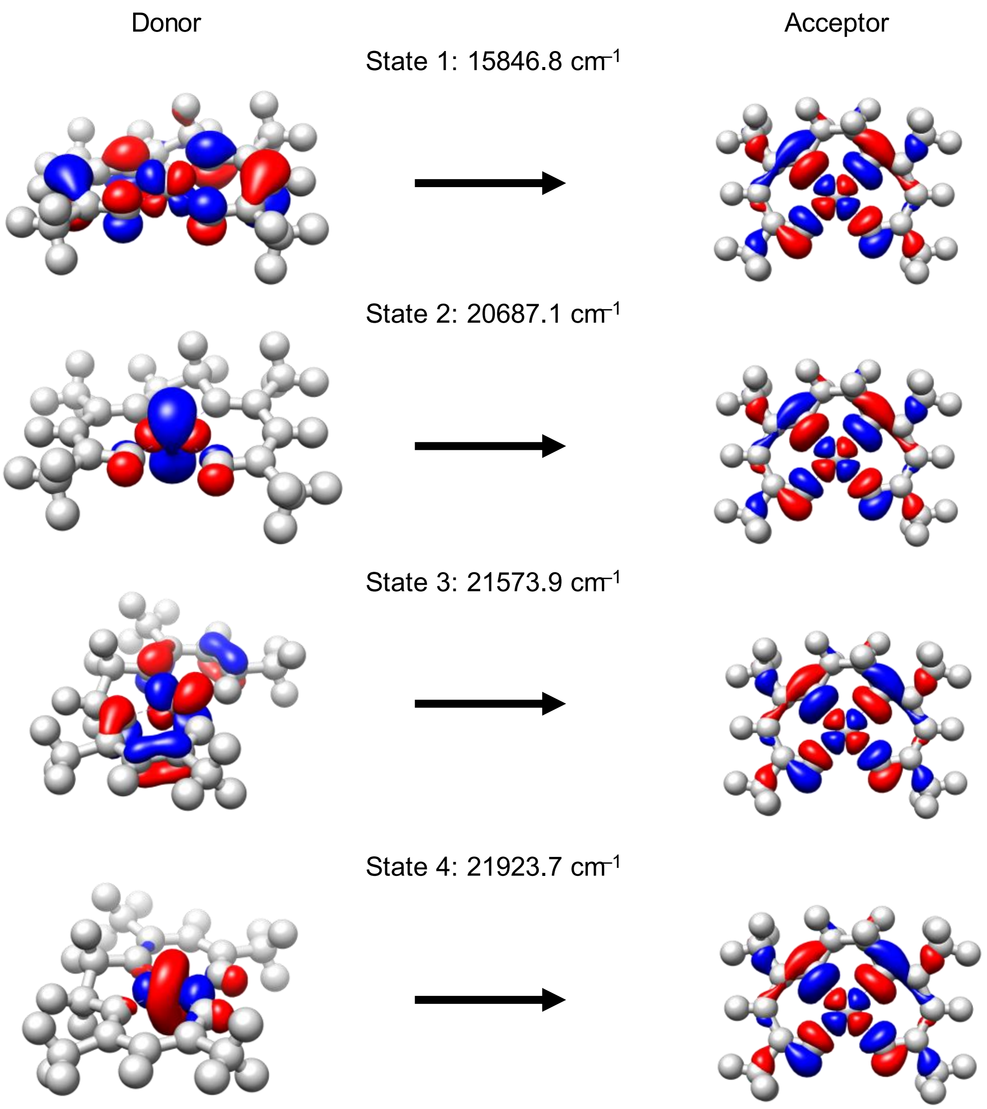

Figure S132: TDDFT natural transition orbitals for Cu(acacen).

Table S53: TDDFT-calculated Cu(acacen) UV-vis-NIR absorption spectrum.

Calculated via transition electric dipole moments, including oscillator strength (f_{osc}), net squared transition electric dipole moment (T^2), and transition electric dipole moment vector components.

State	Energy (cm^{-1})	Wavelength (nm)	f_{osc}	T^2 (a.u.^{2})	T_x (a.u.)	T_y (a.u.)	T_z (a.u.)
1	15846.8	631	0.000481907	0.01001	-0.09992	0.00125	-0.00512
2	20687.1	483.4	0.002583548	0.04111	0.2004	-0.00156	-0.03085
3	21573.9	463.5	0.000002892	0.00004	0.00281	-0.00602	0.00015
4	21923.7	456.1	0.007689171	0.11546	0.33979	-0.00196	-0.00007
5	23565.8	424.3	0.000067259	0.00094	-0.0051	-0.03022	0.00026
6	24162.5	413.9	0.000000062	0	0.00066	-0.00042	0.00048
7	24587.3	406.7	0.000012906	0.00017	-0.0005	0.01313	-0.00029
8	26980.1	370.6	0.000906055	0.01106	0.10379	0.0007	-0.01681
9	28927.1	345.7	0.000374727	0.00426	-0.00087	0.06529	-0.00117
10	30087.2	332.4	0.000661433	0.00724	-0.08418	-0.00038	0.01226
11	32358.9	309	0.005677981	0.05777	0.18099	-0.00096	-0.15814
12	33431.1	299.1	0.063116133	0.62153	-0.00179	-0.7883	0.01044
13	34322.8	291.4	0.00327948	0.03146	0.00542	-0.17726	0.0024
14	34491.4	289.9	0.195327803	1.86436	-1.36467	0.00831	0.04427
15	34677.1	288.4	0.048626378	0.46164	-0.67849	0.0055	0.03561
16	35247.2	283.7	0.047998149	0.44831	0.00883	0.66943	-0.00964
17	38119.5	262.3	0.098184357	0.84795	0.88298	-0.00916	-0.26119
18	39517.9	253	0.009279695	0.07731	-0.25921	-0.00091	0.10057
19	40185.4	248.8	0.016363769	0.13406	0.00829	0.366	-0.00591
20	40861.5	244.7	0.009741567	0.07849	-0.00088	-0.28012	0.00393
21	41292	242.2	0.082610137	0.65863	0.00731	0.81144	-0.01186
22	41482.8	241.1	0.003444848	0.02734	-0.16379	-0.01556	-0.01641
23	42010.4	238	0.287955169	2.25655	0.00765	1.50198	-0.02342
24	42169	237.1	0.010983804	0.08575	0.27566	-0.02643	-0.09521
25	42498.3	235.3	0.069938539	0.54178	-0.00756	-0.73592	0.01177

7.1.12 Cu(acacen) (Constrained Optimization)

Table S54: TDDFT-calculated Cu(acacen) (constrained optimization) excited states assigned to d-d transitions.

State	NTO Occupation Number	Energy (cm^{-1})	Assignment
1	0.99766124	16255.3	xz → xy
2	0.99074968	21106.2	z^2 → xy
3	0.99015404	21991.2	yz → xy
4	0.99823545	22409.4	x^2–y^2 → xy

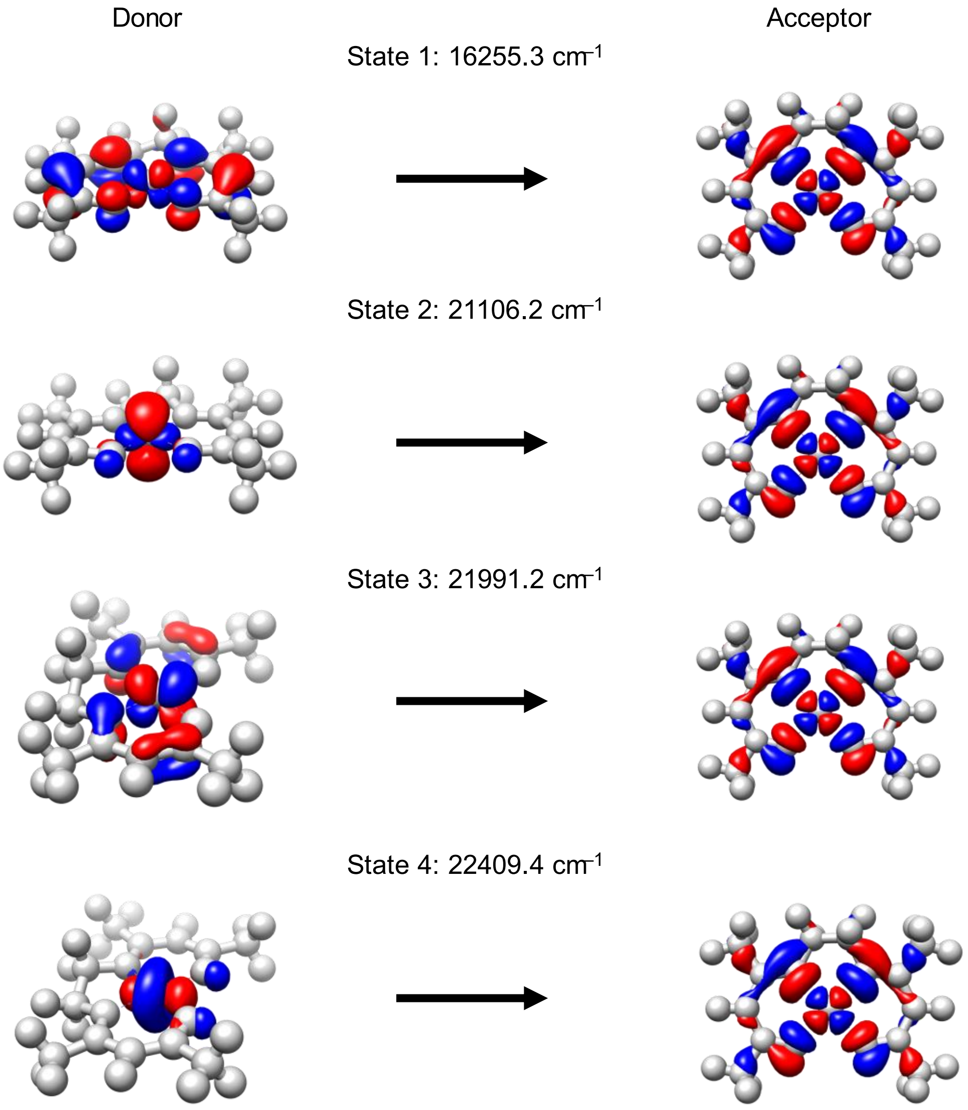

Figure S133: TDDFT natural transition orbitals for Cu(acacen) (constrained optimization).

Table S55: TDDFT-calculated Cu(acacen) (constrained optimization) UV-vis-NIR absorption spectrum.

Calculated via transition electric dipole moments, including oscillator strength (f_{osc}), net squared transition electric dipole moment (T^2), and transition electric dipole moment vector components.

State	Energy (cm^{-1})	Wavelength (nm)	f_{osc}	T^2 (a.u.^{2})	T_x (a.u.)	T_y (a.u.)	T_z (a.u.)
1	16255.3	615.2	0.000995274	0.02016	-0.14183	0.00063	-0.0064
2	21106.2	473.8	0.001220286	0.01903	0.13794	0.0001	-0.00241
3	21991.2	454.7	0.000014223	0.00021	0.00191	-0.01447	0.00008
4	22409.4	446.2	0.007034242	0.10334	0.32145	0.00019	-0.00262
5	24061.5	415.6	0.000021715	0.0003	0.0041	0.01674	-0.00016
6	24102.5	414.9	0.000003195	0.00004	0.00629	0.00087	0.00184
7	24564.2	407.1	0.000000775	0.00001	0.00233	-0.00223	-0.00001
8	27455.6	364.2	0.0045272	0.05428	0.23228	-0.00072	0.01822
9	28495.9	350.9	0.00149573	0.01728	0.00025	0.13145	-0.00031
10	29656.8	337.2	0.000525881	0.00584	-0.07567	-0.00143	0.01051
11	32247.4	310.1	0.002998875	0.03062	-0.16004	-0.00111	0.07072
12	33406.2	299.3	0.065223521	0.64277	0.00582	0.80171	-0.00049
13	34346.7	291.1	0.004619921	0.04428	0.00446	0.21039	-0.00029
14	34547.2	289.5	0.195867636	1.86649	-1.36614	0.00097	0.01172
15	34749.6	287.8	0.05293453	0.50149	0.70804	-0.00386	-0.01265
16	35399.8	282.5	0.054099006	0.50311	-0.0053	0.70928	-0.00072
17	38251	261.4	0.089771628	0.77263	-0.87211	0.00054	0.10979
18	39623.1	252.4	0.009145138	0.07598	-0.26446	0.00122	0.07774
19	40276.3	248.3	0.019276342	0.15756	-0.00186	-0.39694	0.00042
20	40833.9	244.9	0.011364842	0.09163	-0.00038	0.3027	-0.00016
21	41250.3	242.4	0.086916535	0.69367	0.00489	0.83285	-0.0022
22	41515.4	240.9	0.005278264	0.04186	0.18982	-0.02099	-0.07337
23	41822.6	239.1	0.003581616	0.02819	-0.16682	-0.01682	-0.009
24	42201.7	237	0.183480953	1.43132	-0.0061	1.19636	-0.00096
25	42456	235.5	0.161910389	1.25549	-0.00071	1.12048	-0.00127

7.1.13 Cu(pci)$_2$

Table S56: TDDFT-calculated Cu(pci)$_2$ excited states assigned to d-d transitions.

State	NTO Occupation Number	Energy (cm^{-1})	Assignment
1	0.99779971	17484.9	$yz \rightarrow xy$
2	0.99615993	18961.7	$xz \rightarrow xy$
3	0.99389232	20233	$z^2 \rightarrow xy$
7	0.99583767	22457.6	$x^2–y^2 \rightarrow xy$

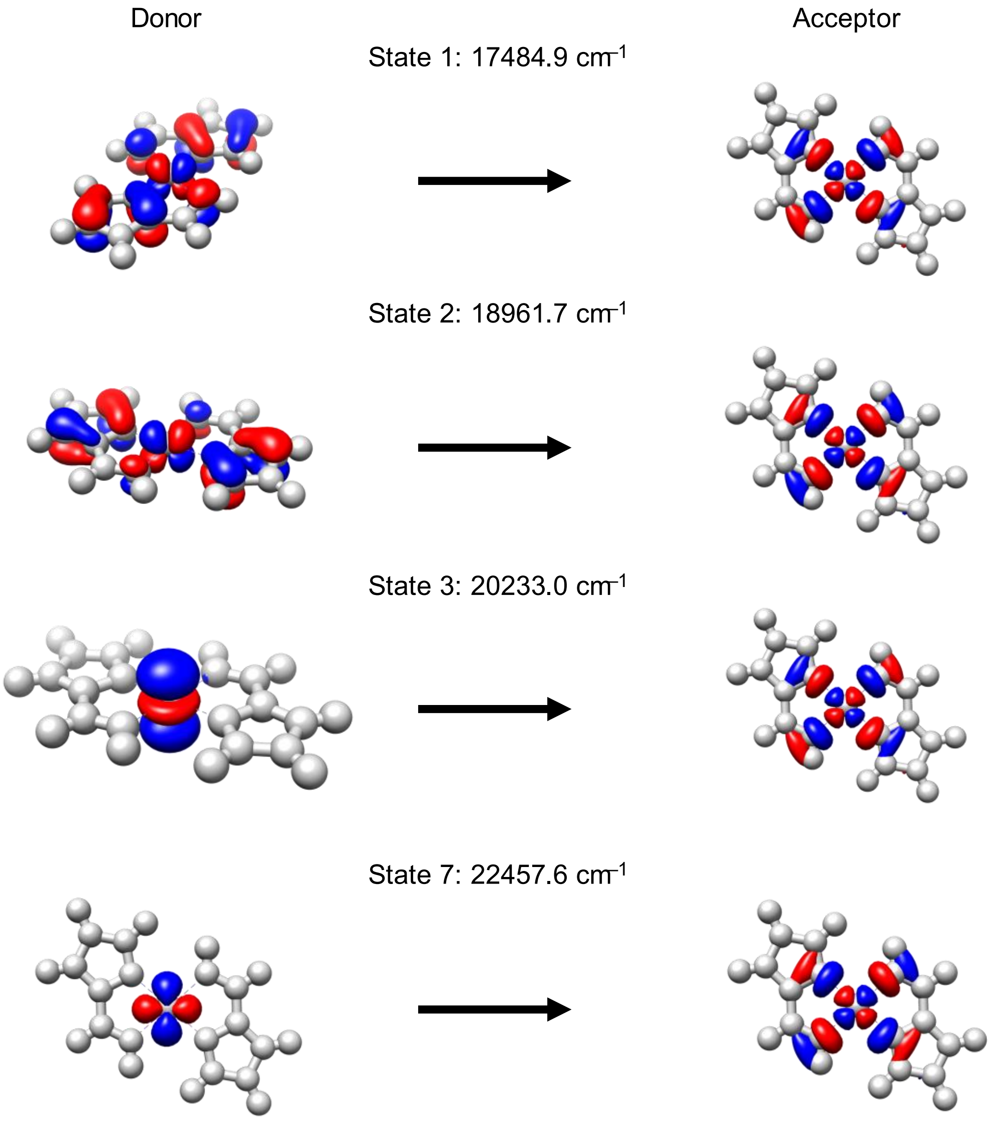

Figure S134: TDDFT natural transition orbitals for Cu(pci)$_2$.

Table S57: TDDFT-calculated $Cu(pci)_2$ UV-vis-NIR absorption spectrum.

Calculated via transition electric dipole moments, including oscillator strength (f_{osc}), net squared transition electric dipole moment (T^2), and transition electric dipole moment vector components.

State	Energy (cm^{-1})	Wavelength (nm)	f_{osc}	T^2 (a.u.^{2})	T_x (a.u.)	T_y (a.u.)	T_z (a.u.)
1	17484.9	571.9	0	0	0	0	0
2	18961.7	527.4	0	0	0.00001	-0.00001	0
3	20233	494.2	0	0	0	0	0
4	20865.4	479.3	0.000087245	0.00138	-0.00004	0.00085	-0.03709
5	22093.6	452.6	0.000007557	0.00011	-0.00034	0.01061	0.00004
6	22360.4	447.2	0	0	0	0	0
7	22457.6	445.3	0	0	0	0	0
8	24017	416.4	0	0	0.00001	-0.00001	0
9	27602.4	362.3	0.000119078	0.00142	0.00165	0.00031	-0.03765
10	28902	346	0.000137188	0.00156	0.00134	0.03951	0.00003
11	29098.5	343.7	0.000027579	0.00031	0.00149	0.00156	0.01753
12	29412.1	340	0	0	0	0	0
13	30273.7	330.3	0.119116179	1.29533	-0.38668	1.07023	0.02053
14	30734.7	325.4	0	0	0.00001	-0.00003	0
15	30753.5	325.2	0	0	-0.00004	0.00004	0
16	31167.7	320.8	0	0	0	0	0
17	31299.9	319.5	0	0	0	-0.00002	0
18	31379.3	318.7	0.000117746	0.00124	-0.01333	-0.03252	-0.00022
19	33661.6	297.1	0.144505985	1.41327	0.40338	1.11825	0.0084
20	33715	296.6	0.000000001	0	-0.00004	-0.00008	-0.00001
21	35855.3	278.9	0.445247184	4.08811	-1.2674	1.57482	0.04178
22	37129.2	269.3	0	0	0	0	0
23	37542.6	266.4	0	0	0	0	0
24	37715.3	265.1	0.000066446	0.00058	0.01622	0.0178	-0.00003
25	38446.8	260.1	0	0	0	0	-0.00001
26	39407.2	253.8	0.235208067	1.96495	-1.07105	0.90385	0.02944
27	41807.2	239.2	0.403608974	3.17823	-1.27118	1.24939	0.03683
28	42576.5	234.9	0.053548342	0.41405	0.13247	0.62965	0.00633
29	42923.5	233	0	0	-0.00001	-0.00003	0
30	43547.1	229.6	0.347919406	2.63024	0.47941	1.54927	0.01301

7.1.14 $(PPh_4)_2[Cu(mnt)_2]$

Table S58: TDDFT-calculated $(PPh_4)_2[Cu(mnt)_2]$ excited states assigned to d-d transitions.

State	NTO Occupation Number	Energy (cm^{-1})	Assignment
1	0.99715116	7700.6	xz → xy
5	0.96396799	17048.8	yz → xy
6	0.94669883	18012.4	z^2 → xy
10	0.97534984	21118.1	$x^2–y^2$ → xy

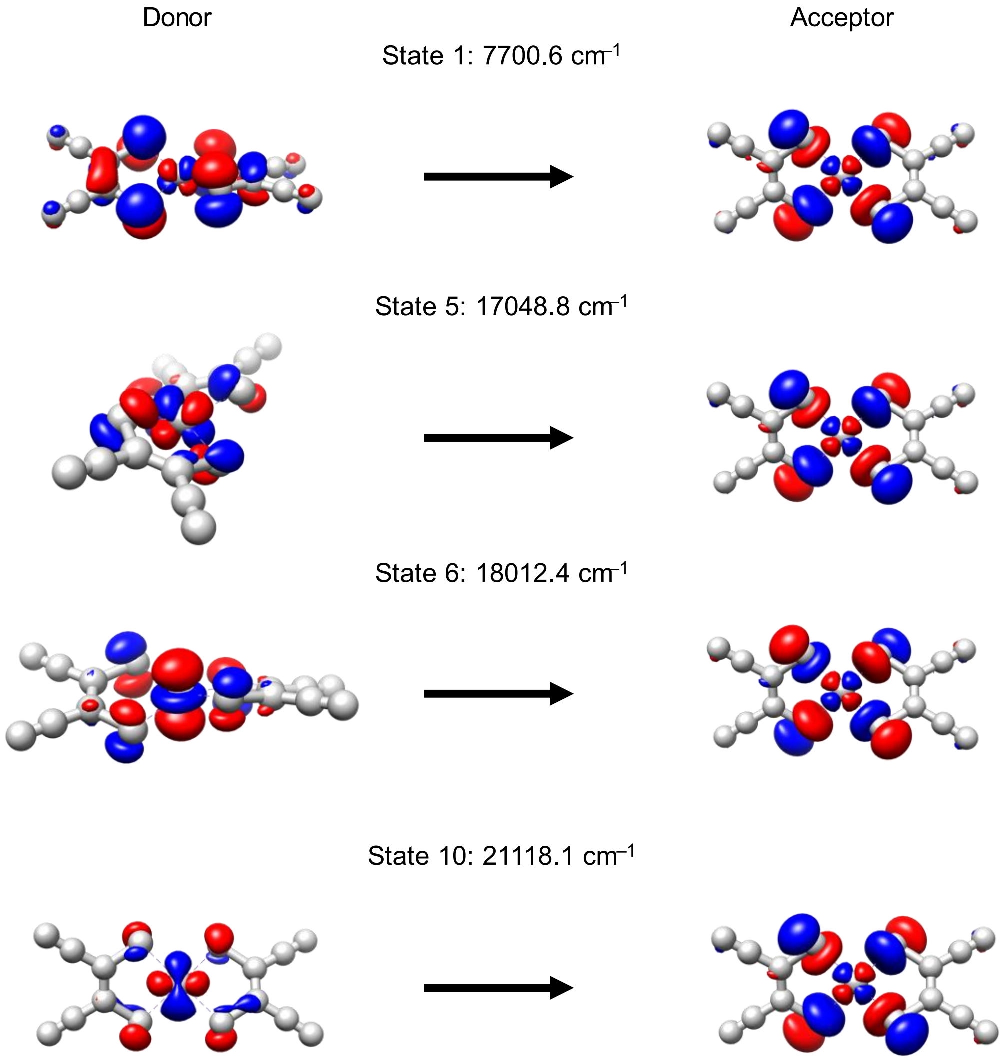

Figure S135: TDDFT natural transition orbitals for $(PPh_4)_2[Cu(mnt)_2]$.

Table S59: TDDFT-calculated $(PPh_4)_2[Cu(mnt)_2]$ UV-vis-NIR absorption spectrum.

Calculated via transition electric dipole moments, including oscillator strength (f_{osc}), net squared transition electric dipole moment (T^2), and transition electric dipole moment vector components.

State	Energy (cm^{-1})	Wavelength (nm)	f_{osc}	T^2 (a.u.^{2})	T_x (a.u.)	T_y (a.u.)	T_z (a.u.)
1	7700.6	1298.6	0.002110774	0.09024	0.30019	0.00087	0.01122
2	13891.4	719.9	0.000001274	0.00003	0.00048	0.00537	-0.00106
3	16209.7	616.9	0.000000781	0.00002	-0.00373	0.00041	0.00132
4	16241.6	615.7	0.000021626	0.00044	0.00007	-0.02093	0.00029
5	17048.8	586.6	0.000296022	0.00572	0	-0.0756	0.00034
6	18012.4	555.2	0.000571747	0.01045	0.06924	0.00005	-0.0752
7	20057.2	498.6	0.191856869	3.14907	1.77139	0.00555	0.10587
8	20270.1	493.3	0.001997943	0.03245	0.03514	0.17667	0.00111
9	20464.5	488.7	0.006292693	0.10123	0.31167	0.00113	-0.06397
10	21118.1	473.5	0.006898274	0.10754	0.32536	0.00195	0.04089
11	22474.1	445	0.015827053	0.23184	-0.00109	0.48149	-0.00215
12	23643.2	423	0.010867666	0.15132	0.06174	0.00198	0.38407
13	24905	401.5	0.014587422	0.19283	0.0027	-0.43911	0.00163
14	25563	391.2	0.001009569	0.013	0.04104	-0.00065	0.10638
15	25672	389.5	0.01164224	0.1493	0.00099	0.38639	-0.00087
16	26010.1	384.5	0.053025793	0.67115	0.81923	0.00253	-0.0037
17	26493.9	377.4	0.102254155	1.27061	-1.12114	-0.00509	-0.11672
18	28085	356.1	0.004594644	0.05386	-0.01073	0.23181	-0.00233
19	28628	349.3	0.026715056	0.30721	0.17995	-0.00058	-0.52424
20	29548.4	338.4	0.000363425	0.00405	-0.04412	-0.04477	-0.00992
21	29565	338.2	0.003024645	0.03368	0.17954	-0.00939	0.03683
22	30574	327.1	0.142022461	1.52926	-0.00116	1.23662	-0.00464
23	30759.5	325.1	0.000001736	0.00002	-0.00008	-0.00431	0.00004
24	30870.8	323.9	0.001456408	0.01553	-0.08131	-0.00776	-0.09412
25	31483.1	317.6	0.010120924	0.10583	0.00992	-0.00584	-0.32512
26	31775.5	314.7	0.256107426	2.65341	0.00788	-1.6289	0.00682
27	32166.4	310.9	0.04512438	0.46183	-0.00251	0.67957	-0.00278
28	33488.2	298.6	0.001724306	0.01695	-0.00848	-0.00003	-0.12992
29	33663.4	297.1	0.000002003	0.00002	-0.00011	0.00416	0.00152
30	33702.1	296.7	0.00036829	0.0036	-0.0011	0.05997	0.00049
31	33807.3	295.8	0.00047978	0.00467	0.06677	-0.00049	-0.01462
32	34077.3	293.5	0.004766513	0.04605	-0.18426	-0.00057	-0.10999
33	34869.1	286.8	0.190402941	1.79767	-0.02122	1.34059	-0.00529
34	35180.8	284.2	0.000351264	0.00329	0.02397	0.00466	-0.05187
35	35395.2	282.5	0.067184358	0.62488	0.79028	0.01232	0.01366
36	35605.4	280.9	0.002791614	0.02581	-0.00673	-0.16052	-0.00071
37	35806.5	279.3	0.022267273	0.20473	-0.05765	-0.00596	-0.44874
38	35857.4	278.9	0.034938726	0.32078	0.00256	-0.56634	0.00492

39	36188.5	276.3	0.00014263	0.0013	0.00949	0.03425	-0.00584
40	36195.7	276.3	0.005080099	0.04621	0.19592	-0.00653	-0.08819
41	36951.5	270.6	0.273882304	2.4401	-1.56207	-0.00715	0.00061
42	37527.9	266.5	0.000149537	0.00131	0.00783	-0.03513	0.00402
43	37847.6	264.2	0.042231363	0.36734	0.6049	0.00629	0.03737
44	37877.5	264	0.000022296	0.00019	0.00275	0.01296	0.00426
45	38226.7	261.6	0.002965418	0.02554	-0.08332	-0.00108	-0.13636
46	38771.6	257.9	0.015081015	0.12805	-0.2271	-0.00366	-0.27653
47	38989.5	256.5	0.000937143	0.00791	-0.00469	-0.08873	0.00418
48	39069.9	256	0.022852784	0.19256	0.43831	0.00459	-0.0207
49	39294.4	254.5	0.001890224	0.01584	0.00071	-0.12584	0.00037
50	39802.5	251.2	0.04150821	0.34332	0.0035	0.00364	0.58591

7.1.15 $(PPh_4)_2[Cu(mnt)_2]$ (Constrained Optimization)

Table S60: TDDFT-calculated $(PPh_4)_2[Cu(mnt)_2]$ (Constrained Optimization) excited states assigned to d-d transitions.

State	NTO Occupation Number	Energy (cm^{-1})	Assignment
1	0.99737459	8288.8	xz → xy
5	0.84136218	18362.1	yz → xy
7	0.94999679	19618.9	z^2 → xy
11	0.99229988	22960.7	$x^2–y^2$ → xy

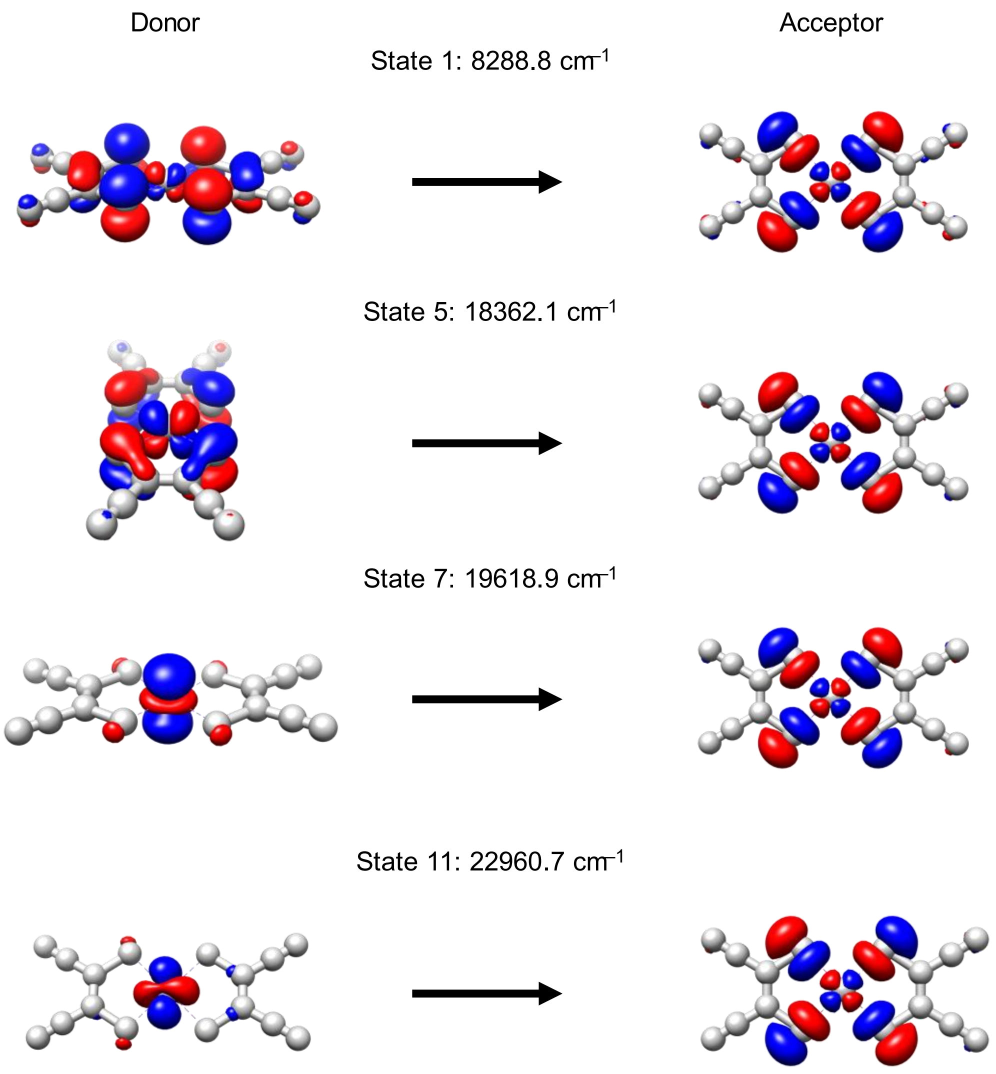

Figure S136: TDDFT natural transition orbitals for $(PPh_4)_2[Cu(mnt)_2]$ (Constrained Optimization).

Table S61: TDDFT-calculated $(PPh_4)_2[Cu(mnt)_2]$ (Constrained Optimization) UV-vis-NIR absorption spectrum.

Calculated via transition electric dipole moments, including oscillator strength (f_{osc}), net squared transition electric dipole moment (T^2), and transition electric dipole moment vector components.

State	Energy (cm^{-1})	Wavelength (nm)	f_{osc}	T^2 (a.u.^{2})	T_x (a.u.)	T_y (a.u.)	T_z (a.u.)
1	8288.8	1206.4	0.000000001	0	-0.00004	0.00014	-0.00002
2	15203.2	657.8	0.000028677	0.00062	-0.0027	-0.02475	-0.00106
3	15590.4	641.4	0	0	-0.00005	-0.00009	-0.00001
4	16393.2	610	0.000083445	0.00168	0.00024	-0.04093	0.00018
5	18362.1	544.6	0.000000005	0	-0.00031	0.00001	0.00003
6	19405.4	515.3	0.00000004	0	-0.00083	0.00004	-0.00004
7	19618.9	509.7	0.000000011	0	-0.00044	0.00001	-0.00003
8	20735	482.3	0.000029578	0.00047	0.02158	0.00186	0.00078
9	22433.1	445.8	0.000000032	0	0.00064	0.00011	-0.00021
10	22942.1	435.9	0.022667088	0.32527	0.04726	-0.56836	0.00214
11	22960.7	435.5	0.000085886	0.00123	-0.00349	0.03492	-0.00017
12	23658	422.7	0.198432136	2.76128	1.66087	0.01151	0.05147
13	23928.9	417.9	0.000005839	0.00008	0.00891	-0.00098	0.00023
14	24635.5	405.9	0.198560454	2.65343	1.62866	0.01045	0.0281
15	25224.8	396.4	0.001482372	0.01935	0.00878	-0.13881	-0.00005
16	25536.4	391.6	0.007337036	0.09459	-0.00556	0.3075	0.00011
17	26070	383.6	0	0	-0.00006	-0.00004	-0.00002
18	29176.2	342.7	0.000000003	0	0.00018	0.00004	0.00001
19	29565.4	338.2	0.000001736	0.00002	-0.0044	-0.00005	-0.00008
20	29623	337.6	0.002114583	0.0235	-0.15329	0.00139	-0.00134
21	30029.7	333	0.000000018	0	0.00036	-0.00006	0.00024
22	30071.6	332.5	0.000000081	0	-0.00088	-0.00033	-0.00008
23	30661.7	326.1	0.000000114	0	-0.0002	0.00109	-0.00001
24	30803.2	324.6	0.000000009	0	0.00002	0.00017	0.00025
25	31323.8	319.2	0.005309066	0.0558	0.23232	-0.00361	-0.04257
26	32713.2	305.7	0.726620762	7.3124	0.00906	2.70413	-0.00196
27	32713.8	305.7	0.092200036	0.92785	0.00489	0.96323	-0.0006
28	32895.8	304	0.000022048	0.00022	0.00986	-0.01073	-0.00289
29	34133.9	293	0.005166893	0.04983	-0.00413	0.2232	0.00037
30	34509.8	289.8	0.01702372	0.1624	0.39811	0.00993	-0.06172
31	34527.8	289.6	0.000055309	0.00053	-0.02259	-0.00054	0.00409
32	34701.5	288.2	0.006352504	0.06027	0.23701	-0.01457	0.06229
33	35358	282.8	0.000000263	0	0.00027	-0.00154	-0.00001
34	35978.2	277.9	0.0000009	0.00001	0.0028	0.00052	0.00035
35	36177.7	276.4	0.000000017	0	0.00037	-0.00008	0.00012
36	36342.8	275.2	0.098069885	0.88837	0.93895	0.00869	0.08164
37	36584	273.3	0.006954513	0.06258	-0.01388	-0.24978	-0.00119

38	36602.1	273.2	0.000000422	0	-0.00079	-0.00177	0.0002
39	36677.2	272.6	0.001460739	0.01311	-0.02849	0.11089	-0.00201
40	36821.4	271.6	0.100072052	0.89472	-0.94249	-0.00231	-0.08024
41	37070.6	269.8	0.000000506	0	0.00198	-0.00006	0.00076
42	37155.9	269.1	0.000000039	0	-0.00058	0.00011	-0.00002
43	37690.1	265.3	0.000040743	0.00036	0.01878	-0.00088	0.00161
44	37709.8	265.2	0.16695136	1.45751	1.203	-0.01071	0.10089
45	37913.8	263.8	0.000000066	0	0.00003	-0.00075	0.00004
46	38222.4	261.6	0.001171653	0.01009	-0.09648	-0.00006	0.02797
47	38355.8	260.7	0.000000281	0	-0.00121	-0.0004	-0.00089
48	38416.3	260.3	0.014841161	0.12718	0.35088	0.00067	-0.06376
49	38691.5	258.5	0.000000065	0	-0.00043	-0.00046	-0.00039
50	39673.6	252.1	0.001536066	0.01275	-0.0001	-0.1129	0.00095

7.1.16 Cu(dtc)$_2$

Table S62: TDDFT-calculated Cu(dtc)$_2$ excited states assigned to d-d transitions.

State	NTO Occupation Number	Energy (cm^{-1})	Assignment
1	0.99740486	15643.3	yz → xy
2	0.99584757	17622.5	xz → xy
4	0.99479663	20320.3	z^2 → xy
6	0.99866342	22856.7	x^2–y^2 → xy

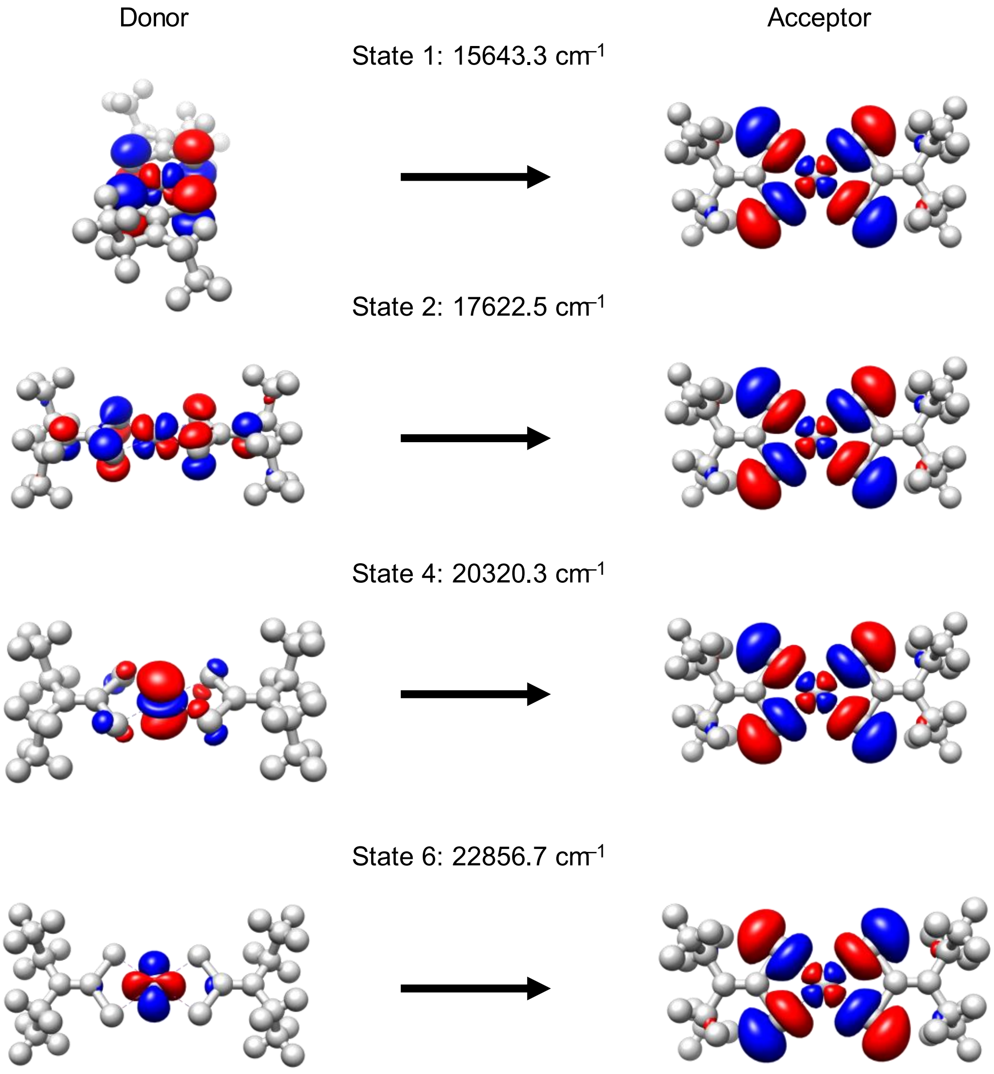

Figure S137: TDDFT natural transition orbitals for Cu(dtc)$_2$.

Table S63: TDDFT-calculated $Cu(dtc)_2$ UV-vis-NIR absorption spectrum.

Calculated via transition electric dipole moments, including oscillator strength (f_{osc}), net squared transition electric dipole moment (T^2), and transition electric dipole moment vector components.

State	Energy (cm^{-1})	Wavelength (nm)	f_{osc}	T^2 (a.u.^{2})	T_x (a.u.)	T_y (a.u.)	T_z (a.u.)
1	15643.3	639.3	0.000006552	0.00014	-0.00643	-0.00976	0.0011
2	17622.5	567.5	0.00321956	0.06015	-0.24474	-0.00919	-0.01277
3	18725.6	534	0.000227625	0.004	0.0367	-0.02748	-0.04359
4	20320.3	492.1	0.000024234	0.00039	-0.01411	0.00562	-0.01272
5	22176.3	450.9	0.434765986	6.4542	-2.53665	-0.04021	-0.13418
6	22856.7	437.5	0.000011717	0.00017	-0.00735	-0.00232	-0.01046
7	24088.8	415.1	0.001351832	0.01847	0.1357	0.00213	0.00752
8	25716.4	388.9	0.003232408	0.04138	0.2032	0.0007	0.00943
9	26498.9	377.4	0.004005958	0.04977	-0.03406	0.21976	-0.01775
10	26634.7	375.5	0.000160296	0.00198	0.0422	-0.01378	0.00328
11	27704.7	360.9	0.021128446	0.25107	0.0023	0.4996	-0.0382
12	28491.2	351	0.000156597	0.00181	-0.04199	-0.00658	-0.00178
13	30546.2	327.4	0.000339261	0.00366	-0.00005	0.06043	-0.00216
14	30549.7	327.3	0.000127884	0.00138	-0.00104	-0.03666	0.00574
15	31439.5	318.1	0.001125273	0.01178	-0.10841	-0.00126	-0.00546
16	32197.8	310.6	0.000006692	0.00007	0.00827	0.00004	0.00018
17	32425.7	308.4	0.000001046	0.00001	-0.00109	0.00136	0.00275
18	33550.3	298.1	0.001458394	0.01431	-0.11949	-0.00273	-0.00496
19	34917.8	286.4	0.000000198	0	-0.00028	0.00011	-0.00133
20	35662.2	280.4	0.000147877	0.00137	-0.00138	-0.00207	0.03686
21	36030.2	277.5	0.000596032	0.00545	-0.0727	-0.01225	-0.00325
22	36587.2	273.3	0.107978566	0.97159	-0.01754	-0.98352	0.06309
23	37750.2	264.9	0.008245263	0.07191	0.26784	-0.00016	0.01302
24	38324	260.9	0.000007091	0.00006	0.00553	0.00304	0.0046
25	38433.5	260.2	0.008991352	0.07702	-0.00205	-0.27696	0.01756
26	38585.6	259.2	0.00004854	0.00041	0.00239	-0.00294	0.02
27	39144.2	255.5	0.000016252	0.00014	-0.00948	0.00328	0.00601
28	40117.2	249.3	0.476430189	3.90971	-1.97496	-0.00652	-0.096
29	40148.6	249.1	0.044596879	0.36569	0.59938	0.07646	0.02405
30	40820.7	245	0.030694976	0.24755	0.03053	-0.4952	0.03732
31	41033.6	243.7	0.181883104	1.45925	-0.01343	1.20553	-0.07584
32	41054	243.6	0.003768502	0.03022	-0.01245	0.17302	-0.01128
33	41237.8	242.5	0.012858374	0.10265	-0.04731	0.31646	-0.01624
34	41252.9	242.4	0.00072861	0.00581	-0.00248	-0.07613	0.00359
35	42060.7	237.8	0.000016833	0.00013	-0.00522	0.01008	-0.00172
36	42408.2	235.8	0.000778212	0.00604	-0.00228	-0.00025	0.07769
37	42613	234.7	0.000003815	0.00003	0.00026	-0.00483	-0.00247
38	42922.4	233	0.006352986	0.04873	0.22041	0.00585	0.01056

39	43065.3	232.2	0.00088513	0.00677	0.08158	-0.01015	0.00282
40	43337.9	230.7	0.000789053	0.00599	0.00516	0.07472	0.01961
41	43402.4	230.4	0.000043946	0.00033	0.0089	0.01531	0.00445
42	43688.4	228.9	0.024396914	0.18384	0.42375	0.05823	0.02983
43	43824.8	228.2	0.009446276	0.07096	0.09385	-0.24707	-0.03326
44	44218.8	226.1	0.000111014	0.00083	0.01628	-0.02015	0.01246
45	44508	224.7	0.000866226	0.00641	0.00628	-0.07943	-0.00765
46	44737.1	223.5	0.000082172	0.0006	0.01107	-0.002	0.02187
47	45787.1	218.4	0.000269484	0.00194	0.00558	0.04356	0.00307
48	46247.6	216.2	0.001348075	0.0096	0.09795	0.0006	0.0009
49	46329.4	215.8	0.004170757	0.02964	0.16964	-0.02717	-0.01105
50	46364.8	215.7	0.390635033	2.77369	-1.66375	-0.01951	-0.07233

7.1.17 $Cu(dtc)_2$ (Constrained Optimization)

Table S64: TDDFT-calculated $Cu(dtc)_2$ (Constrained Optimization) excited states assigned to d-d transitions.

State	NTO Occupation Number	Energy (cm^{-1})	Assignment
1	0.99741057	15654.5	yz → xy
2	0.99609641	17715.6	xz → xy
4	0.99475322	20242.4	z^2 → xy
6	0.99871534	22852.6	$x^2–y^2$ → xy

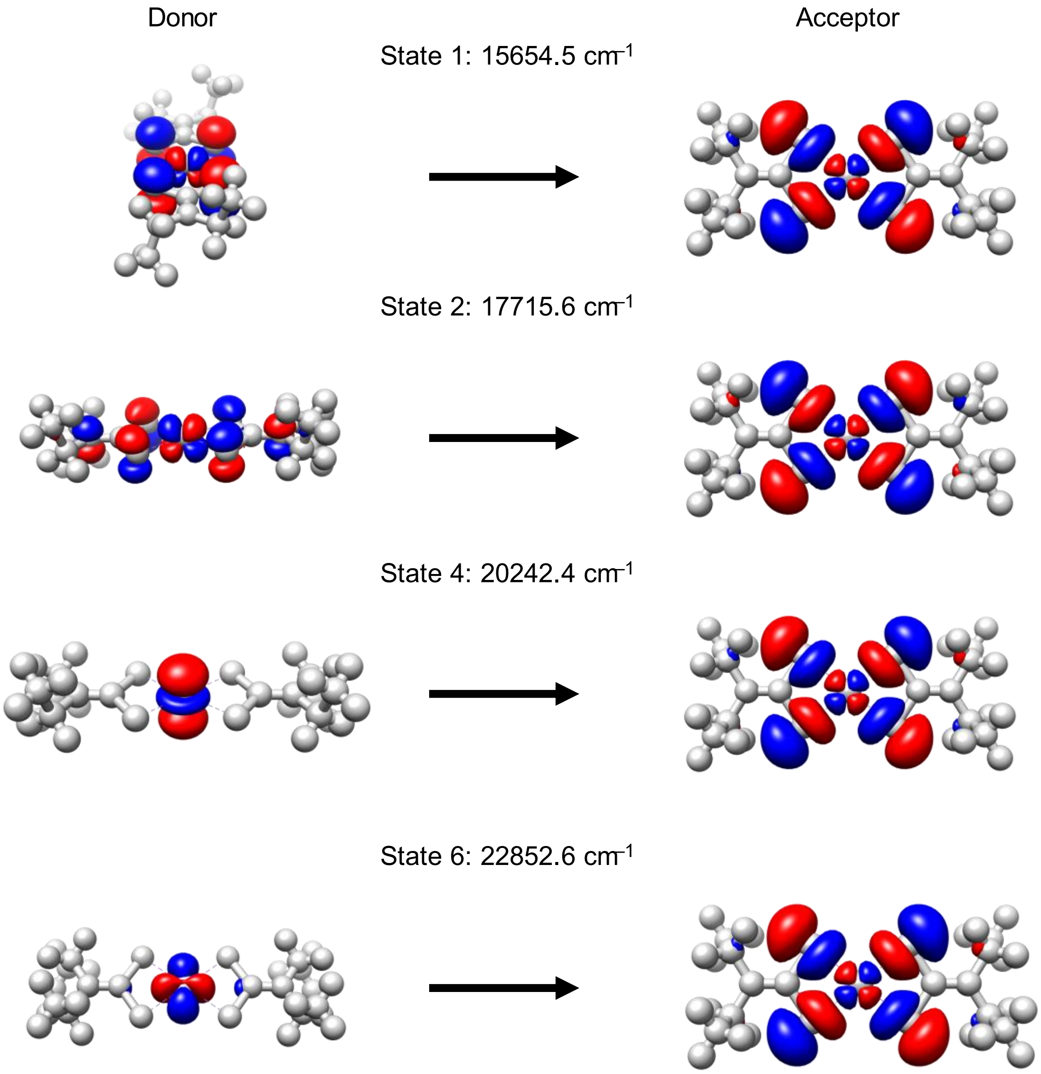

Figure S138: TDDFT natural transition orbitals for $Cu(dtc)_2$ (Constrained Optimization).

Table S65: TDDFT-calculated Cu(dtc)$_2$ (Constrained Optimization) UV-vis-NIR absorption spectrum.

Calculated via transition electric dipole moments, including oscillator strength (f_{osc}), net squared transition electric dipole moment (T^2), and transition electric dipole moment vector components.

State	Energy (cm^{-1})	Wavelength (nm)	f_{osc}	T^2 (a.u.^{2})	T_x (a.u.)	T_y (a.u.)	T_z (a.u.)
1	15654.5	638.8	0.000000003	0	0.00025	-0.00003	0.00005
2	17715.6	564.5	0.000000014	0	-0.00047	0.00018	-0.0001
3	18844.6	530.7	0.000571146	0.00998	-0.08578	-0.02556	0.04434
4	20242.4	494	0.000000014	0	0.00023	-0.00043	-0.00001
5	22152.5	451.4	0.439278938	6.52818	2.55501	-0.00543	-0.00884
6	22852.6	437.6	0.00002154	0.00031	-0.01758	-0.00113	0.0001
7	24072.1	415.4	0.001443962	0.01975	-0.14053	-0.0006	0.00011
8	25744.5	388.4	0.002960759	0.03786	-0.1943	-0.01031	0.00177
9	26639.8	375.4	0.000000004	0	0.00011	-0.0002	0.00002
10	26720.5	374.2	0.000000022	0	0.00007	0.00051	-0.00007
11	27544.9	363	0.025102261	0.30002	-0.00541	-0.54765	0.00809
12	28509.1	350.8	0.000000006	0	-0.00021	-0.00014	-0.00002
13	30539.4	327.4	0.000227945	0.00246	0.00116	-0.04955	0.00103
14	30539.9	327.4	0.000258158	0.00278	0.00135	-0.05272	0.00111
15	31457.9	317.9	0.001010808	0.01058	0.10285	0.0002	-0.00106
16	32211.6	310.4	0.000000004	0	-0.00018	0.00008	-0.00001
17	32365.1	309	0.000000007	0	0.00026	-0.00005	-0.00002
18	33544.1	298.1	0.000000002	0	-0.00009	-0.00007	0.00006
19	34940.1	286.2	0.000000503	0	-0.00218	0.00001	0.00006
20	35649.7	280.5	0.000000293	0	0.00156	0.00051	0.00004
21	36055.7	277.3	0.000610771	0.00558	0.06907	-0.0284	0.00024
22	36579.6	273.4	0.108587605	0.97728	0.02437	-0.98791	0.02667
23	37781.3	264.7	0.000000091	0	0.00088	0.00014	-0.00008
24	38332.2	260.9	0.000019962	0.00017	-0.0003	-0.01309	0.00037
25	38441.8	260.1	0.008779722	0.07519	-0.002	0.27409	-0.00773
26	38572.5	259.3	0.00000108	0.00001	-0.00087	-0.00291	0.00001
27	39159	255.4	0.000002305	0.00002	-0.00079	-0.00433	0.00016
28	40095.1	249.4	0.519602207	4.26634	2.0652	0.01489	-0.03296
29	40195.7	248.8	0.015431142	0.12638	-0.30834	0.17694	-0.00058
30	40806.5	245.1	0.017536532	0.14148	-0.02956	-0.37491	0.00701

7.1.18 $(PPh_4)_2[Cu(bdt)_2]$

Table S66: TDDFT-calculated $(PPh_4)_2[Cu(bdt)_2]$ excited states assigned to d-d transitions.

State	NTO Occupation Number	Energy (cm^{-1})	Assignment
1	0.99775490	10080.2	xz → xy
2	0.99643842	16196.7	yz → xy
5	0.99532803	21314.8	z^2 → xy
7	0.99706855	23813.2	$x^2–y^2$ → xy

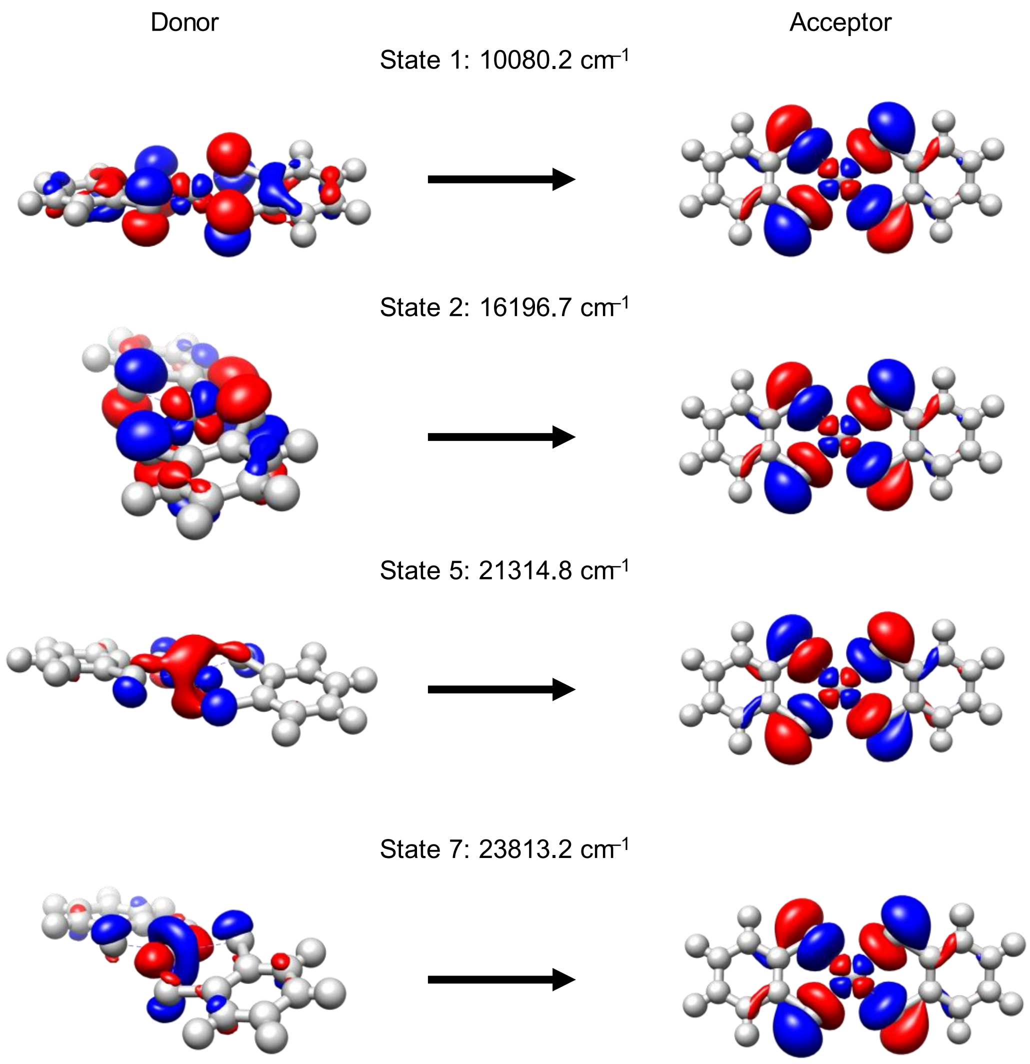

Figure S139: TDDFT natural transition orbitals for $(PPh_4)_2[Cu(bdt)_2]$.

Table S67: TDDFT-calculated $(PPh_4)_2[Cu(bdt)_2]$ UV-vis-NIR absorption spectrum.

Calculated via transition electric dipole moments, including oscillator strength (f_{osc}), net squared transition electric dipole moment (T^2), and transition electric dipole moment vector components.

State	Energy (cm^{-1})	Wavelength (nm)	f_{osc}	T^2 (a.u.^{2})	T_x (a.u.)	T_y (a.u.)	T_z (a.u.)
1	10080.2	992	0.001286927	0.04203	0.20501	-0.00009	-0.00078
2	16196.7	617.4	0.000026886	0.00055	-0.0004	0.02337	0.00004
3	16841.8	593.8	0.000000029	0	0.00042	0.00013	-0.0006
4	17885.2	559.1	0.000540419	0.00995	0.00028	-0.00023	0.09974
5	21314.8	469.2	0.000538363	0.00832	0.002	0.00016	-0.09117
6	22024.8	454	0.257620701	3.85075	-1.9623	-0.00732	0.00739
7	23813.2	419.9	0.000093316	0.00129	0.00018	0.00013	-0.03592
8	24763.2	403.8	0.035530106	0.47235	-0.00522	0.68726	0.00147
9	26317	380	0.127129769	1.59033	1.26107	0.00241	-0.00481
10	27511.5	363.5	0.000000301	0	-0.00005	0.00016	0.00189
11	27557.5	362.9	0.016045428	0.19168	-0.00261	-0.43781	-0.00091
12	27748.4	360.4	0.000302021	0.00358	0.00028	0.05986	0.00011
13	28027	356.8	0.000613401	0.00721	0.08488	0.00023	-0.00032
14	28073.5	356.2	0.000000026	0	0.00055	-0.00003	0
15	29726.9	336.4	0.000000046	0	-0.00061	0.00017	-0.00032
16	29855.6	334.9	0.00431786	0.04761	0.2182	0.00074	-0.00085
17	30327.6	329.7	0.002116581	0.02298	-0.00106	-0.15157	-0.00028
18	30960.4	323	0.00045628	0.00485	0.00059	-0.00033	0.06965
19	31990.1	312.6	0.000005621	0.00006	-0.00001	0.00005	0.00761
20	32080.9	311.7	0.000133126	0.00137	0.00397	-0.03675	-0.00006
21	32122.2	311.3	0.165870816	1.69997	1.30381	0.00413	-0.00496
22	32404	308.6	0.000000366	0	0.0009	0.00038	-0.00167
23	33436.6	299.1	0.001452598	0.0143	-0.00137	0.00048	-0.11958
24	34661.8	288.5	0.002857838	0.02714	0.00055	-0.00086	0.16475
25	34861.8	286.8	0.074658374	0.70502	-0.00344	0.83965	0.00192
26	34984.2	285.8	0.000000629	0.00001	0.00026	0.00199	0.00138
27	35007.4	285.7	0.002684635	0.02525	-0.15889	-0.00024	0.00061
28	35058.6	285.2	0.000000161	0	0.00098	0.00071	0.0002
29	35345.5	282.9	0.000000124	0	-0.00009	-0.00036	0.00101
30	35823.5	279.1	0.000010383	0.0001	0.00059	-0.00967	-0.00126
31	35973.1	278	0.006038924	0.05527	0.00103	-0.00016	0.23508
32	36256.3	275.8	0.002292416	0.02082	-0.00084	0.14427	0.0002
33	36319.1	275.3	0.000000077	0	-0.00003	-0.00081	-0.0002
34	36451.8	274.3	0.000039795	0.00036	-0.01896	-0.00034	0.00001
35	36771.1	272	0.000869878	0.00779	0.00062	-0.08825	-0.0003
36	37021.5	270.1	0.00233093	0.02073	-0.00036	0.0003	-0.14397
37	37150.4	269.2	0.000000006	0	-0.00003	0.00007	-0.00022
38	37232.6	268.6	0.020248681	0.17904	0.00064	-0.42313	-0.00086

39	37305	268.1	0.004273901	0.03772	0.19419	-0.00257	-0.00078
40	37367	267.6	0.0007283	0.00642	-0.0006	0.00016	-0.0801
41	38608.7	259	0.001981921	0.0169	-0.00053	0.13	0.0003
42	38961.2	256.7	0.006063572	0.05124	0.02046	0.00199	0.22542
43	38963.4	256.7	0.003720693	0.03144	-0.175	-0.01271	0.02552
44	38986.9	256.5	0.025991269	0.21947	-0.00552	0.46845	0.00023
45	39118.3	255.6	0.000000066	0	0.00012	0.00027	0.00068
46	39833.2	251	0.000484671	0.00401	-0.0021	-0.06326	0.00021
47	40065.1	249.6	0.129892096	1.06731	-0.00351	1.0331	0.00224
48	40202.1	248.7	0.007278938	0.05961	-0.00225	0.00335	-0.24411
49	40237.4	248.5	0.041232675	0.33736	-0.00381	0.58081	0.00216
50	40250.8	248.4	0.025949653	0.21224	-0.00181	0.0007	-0.46069

7.1.19 $(PPh_4)_2[Cu(bdt)_2]$ (Constrained Optimization)

Table S68: TDDFT-calculated $(PPh_4)_2[Cu(bdt)_2]$ (Constrained Optimization) excited states assigned to d-d transitions.

State	NTO Occupation Number	Energy (cm^{-1})	Assignment
1	0.99786832	10488.6	xz → xy
2	0.99636127	16820.3	yz → xy
5	0.99319356	21166.7	z^2 → xy
6	0.99886047	23528.1	x^2–y^2 → xy

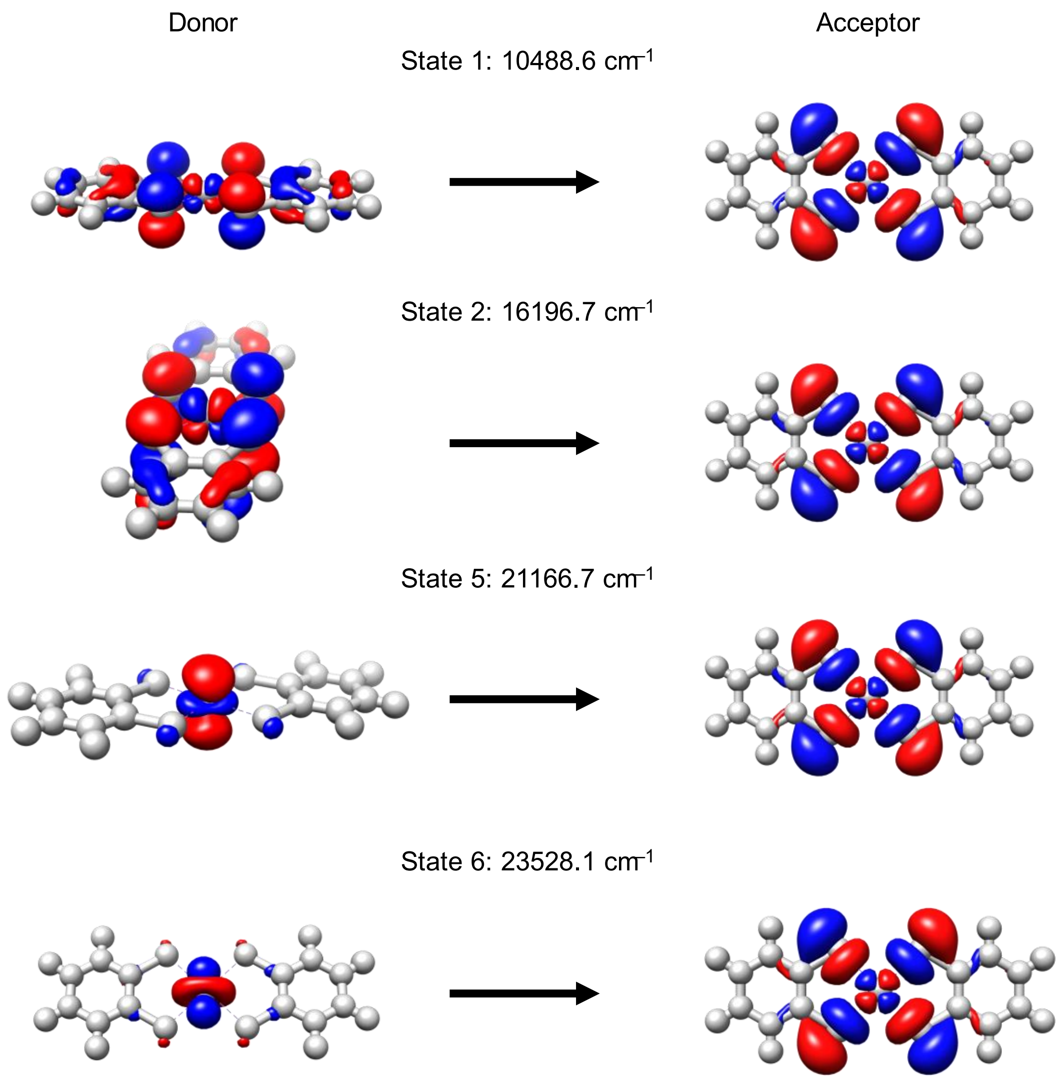

Figure S140: TDDFT natural transition orbitals for $(PPh_4)_2[Cu(bdt)_2]$ (Constrained Optimization).

Table S69: TDDFT-calculated $(PPh_4)_2[Cu(bdt)_2]$ (Constrained Optimization) UV-vis-NIR absorption spectrum.

Calculated via transition electric dipole moments, including oscillator strength (f_{osc}), net squared transition electric dipole moment (T^2), and transition electric dipole moment vector components.

State	Energy (cm^{-1})	Wavelength (nm)	f_{osc}	T^2 (a.u.^{2})	T_x (a.u.)	T_y (a.u.)	T_z (a.u.)
1	10488.6	953.4	0.000000049	0	-0.00124	0.00005	0
2	16820.3	594.5	0.000000001	0	-0.00012	-0.00004	0
3	17664.9	566.1	0.000013792	0.00026	-0.01603	-0.00022	0.00009
4	19434.2	514.6	0.000009275	0.00016	-0.00052	0.00187	-0.01238
5	21166.7	472.4	0	0	-0.00004	0	-0.00003
6	23528.1	425	0.000000011	0	-0.00039	-0.00004	0.00005
7	24183.2	413.5	0.408757119	5.56452	-2.35891	-0.00028	0.00879
8	25178.6	397.2	0.000000593	0.00001	0.00276	0.00035	-0.00001
9	26422.2	378.5	0.053815007	0.67052	0.0001	-0.81885	0.00064
10	27284	366.5	0	0	0	0.00002	0
11	27687.5	361.2	0.000000077	0	-0.00035	-0.00089	0
12	27852.6	359	0.001373911	0.01624	-0.12743	0.00004	0.00045
13	27944.7	357.8	0.000025835	0.0003	0.00033	0.01744	-0.00003
14	28071.2	356.2	0.000000001	0	0.00008	0	-0.00002
15	29194.1	342.5	0.000012704	0.00014	-0.01197	0.00005	0.00003
16	29578	338.1	0.000000016	0	0.00042	0.00002	0
17	29710.4	336.6	0.000000074	0	-0.00031	-0.00085	0
18	31377.8	318.7	0.000006972	0.00007	0.00003	0.00646	0.0056
19	31736.7	315.1	0.194009447	2.0125	1.41862	-0.00006	-0.00496
20	31938.9	313.1	0	0	0	-0.00003	0.00002
21	32113.9	311.4	0.000066265	0.00068	-0.00004	-0.02606	-0.00016
22	32254.1	310	0.000000057	0	-0.00073	-0.00001	0.00022
23	32774.8	305.1	0.000000011	0	-0.00004	0.0001	0.00032
24	33647	297.2	0.000000004	0	-0.00002	0.00008	-0.00019
25	34986.5	285.8	0.001772344	0.01668	0.12914	0.00001	-0.00043
26	35166.4	284.4	0.000000001	0	-0.00011	0.00001	-0.00003
27	35218.1	283.9	0.000000001	0	0.00007	-0.00009	0
28	35494.5	281.7	0	0	-0.00002	0.00001	-0.00004
29	35513.8	281.6	0.000000008	0	0	0.00025	-0.00012
30	35755.6	279.7	0.000000274	0	-0.00029	0.00156	0.00004
31	35778.7	279.5	0.001456843	0.0134	0.00012	0.1099	0.03642
32	35837.1	279	0.116743544	1.07245	0.00034	1.03559	-0.00259
33	36417	274.6	0	0	-0.00003	-0.00003	0.00001
34	36667.5	272.7	0.000213685	0.00192	0.0438	0.00006	-0.00015
35	36709.7	272.4	0.000000385	0	0.0001	-0.00185	-0.00005
36	36788.8	271.8	0.000000018	0	0.00003	-0.00039	-0.00012
37	36826	271.5	0.000000042	0	0.00004	-0.00058	0.00018

38	37230	268.6	0.000000004	0	-0.00017	-0.00003	0.00002
39	37373.7	267.6	0.002821112	0.02485	0.00003	-0.15764	-0.00016
40	37566.7	266.2	0.021601943	0.18931	0.43509	0.00013	-0.00148
41	38037.4	262.9	0	0	0	0.00006	0.00002
42	38250.8	261.4	0.00000011	0	-0.0002	0.00095	0
43	38987.6	256.5	0.000000271	0	-0.00002	0.00142	0.00053
44	39048.4	256.1	0.024162726	0.20371	0.00045	-0.45134	0.00165
45	39506	253.1	0.000000009	0	-0.00016	-0.00022	0
46	39644	252.2	0.000008516	0.00007	-0.00838	0.00074	0.00004
47	40165.2	249	0.118964147	0.97508	0.00019	0.98745	0.00427
48	40413.6	247.4	0.07149034	0.58237	0.00052	-0.76312	-0.00354
49	40436.4	247.3	0.000006042	0.00005	0.00009	0.00701	0.00017
50	40593.3	246.3	0.000051499	0.00042	0.00015	-0.01031	0.01765

7.2 Optimized Coordinates Used for TDDFT Calculations

$K_2[Cu(ox)_2]$

Cu	0.000000000	0.000000000	0.000000000
O	1.437952800	-1.319429733	0.067488289
O	3.667228568	-1.410350512	0.153125875
O	1.441704307	1.312158268	0.096048286
O	3.669887585	1.394905453	0.206900578
C	2.609964394	-0.789744720	0.120881580
C	2.611765519	0.778039742	0.144733035
O	-1.437952902	1.319429786	-0.067482746
O	-3.667228206	1.410350807	-0.153138652
O	-1.441704619	-1.312157925	-0.096052368
O	-3.669888476	-1.394905799	-0.206885250
C	-2.609964410	0.789744995	-0.120882040
C	-2.611766177	-0.778039408	-0.144727583

$K_2[Cu(ox)_2]\bullet 2H_2O$

Cu	0.000000000	0.000000000	0.000000000
O	-1.454407555	1.300287954	-0.256506830
O	-3.696753234	-1.345308025	0.324379999
O	-1.466041550	-1.274013775	0.303088108
O	-3.684406756	1.400878990	-0.232995551
C	-2.625879673	0.788662296	-0.124938114
C	-2.632986996	-0.746789466	0.194901174
O	-0.042687802	-0.209737407	-2.695149923
H	-0.807520290	0.381167915	-2.692567301
H	0.719072471	0.385112229	-2.706042406
O	1.454405564	-1.300235730	0.256796569
O	3.696759563	1.345315863	-0.324278227
O	1.466049465	1.274071769	-0.302764023
O	3.684402794	-1.400868596	0.233109825
C	2.625882019	-0.788631924	0.125125572
C	2.632988631	0.746820699	-0.194707051
O	0.042648254	0.208573633	2.696056109
H	0.807350894	-0.382489950	2.692130732
H	-0.718918644	-0.386548439	2.705486693

$(PPN)_2[Cu(ox)_2]$

Cu	0.000000000	0.000000000	0.000000000
O	1.438880089	1.321312892	0.014082155
C	2.611477939	0.790343513	0.026407713
O	3.670252094	1.409185092	0.037618771
O	1.442415399	-1.312353977	0.017459682
C	2.613666735	-0.778056869	0.027103562
O	3.673958845	-1.394066290	0.036544050
O	-1.438883007	-1.321311035	-0.014070868
C	-2.611479719	-0.790338726	-0.026380398

O	-3.670255298	-1.409177888	-0.037577235
O	-1.442413002	1.312356104	-0.017459893
C	-2.613665427	0.778061426	-0.027077403
O	-3.673956788	1.394072636	-0.036496128

Cu(acac)$_2$

Cu	0.000000000	0.000000000	0.000000000
O	-1.345761660	-1.403822316	-0.033930694
O	1.345767526	1.403836812	0.033517760
O	-1.341760961	1.407128016	-0.010799546
O	1.341769052	-1.407136854	0.010424731
C	-2.608649533	-1.234533453	-0.042990167
C	2.608661601	1.234544416	0.041697033
C	-2.605139078	1.241997651	-0.022435612
C	2.605152609	-1.241989819	0.021184117
H	-3.163626807	-3.084197710	-0.943279618
H	3.164177560	3.084360769	0.941336834
H	-3.139219669	3.120418380	-0.875495697
H	3.139781729	-3.120499005	0.873716045
C	-3.427566347	-2.496658480	-0.060984159
C	-3.257149594	0.004719332	-0.037459419
C	3.257160998	-0.004707092	0.035866914
C	3.427591641	2.496671656	0.058983920
C	-3.419990457	2.506739625	-0.016703429
C	3.420013143	-2.506725264	0.014814948
H	-3.179967315	-3.097878739	0.816823541
H	-4.496175828	-2.293013944	-0.069319525
H	-4.336507674	0.006564474	-0.046812125
H	4.336525569	-0.006545122	0.044460008
H	3.179470034	3.097743898	-0.818777338
H	4.496205996	2.293028300	0.066719387
H	-4.488913529	2.307001693	-0.048246310
H	-3.185926364	3.079796722	0.883582851
H	3.185393993	-3.079687104	-0.885386579
H	4.488954826	-2.306984574	0.045720570

Cu(tmhd)$_2$

C	3.006416114	-3.403082527	1.064235918
H	3.286928129	-2.893208610	1.988727229
H	3.529268132	-4.360840551	1.031340913
H	1.934152928	-3.593492528	1.085502740
C	2.978691869	-3.301262931	-1.451085278
H	1.905699828	-3.489062154	-1.464394905
H	3.500516337	-4.258562130	-1.505819822
H	3.240582436	-2.718611566	-2.337162044
C	4.913494522	-2.362409164	-0.173250014
H	5.243448925	-1.793031553	-1.043641518
H	5.398727278	-3.339412073	-0.209372736
H	5.258358812	-1.850984521	0.726881150

C	3.394900265	-2.561511366	-0.165638419
C	2.602292604	-1.246797684	-0.106335981
C	3.255410303	-0.009611480	-0.086265751
H	4.327532126	-0.012601139	-0.107757498
C	2.610402837	1.231196947	-0.045383187
C	3.412015730	2.541850851	-0.035422783
C	4.929283867	2.333035655	-0.052853716
H	5.271070301	1.775568049	0.820737855
H	5.420826932	3.307462647	-0.041181576
H	5.255266035	1.805212411	-0.950478470
C	3.003082542	3.351343168	-1.280524307
H	3.262299269	2.814406171	-2.195804217
H	3.531625975	4.306539199	-1.284358847
H	1.931527068	3.547455844	-1.285532828
C	3.028332003	3.320750684	1.236718610
H	1.957537557	3.517988388	1.267566685
H	3.558524773	4.274843287	1.253729450
H	3.304259195	2.761261818	2.133443779
O	1.338336958	-1.397552224	-0.081990239
O	1.347224250	1.388850242	-0.017281898
Cu	0.000000000	0.000000000	0.000000000
C	-3.006171107	3.368987572	-1.162802741
H	-3.283391890	2.832732234	-2.073243839
H	-3.530310162	4.326576865	-1.158517879
H	-1.934085116	3.560116552	-1.186461254
C	-2.986475131	3.339277923	1.354495123
H	-1.913783332	3.528795953	1.366200958
H	-3.509521764	4.297147509	1.379792753
H	-3.250844437	2.782176001	2.256127425
C	-4.915871452	2.361795373	0.098155319
H	-5.247664758	1.816514004	0.983132841
H	-5.402135809	3.338922074	0.105810228
H	-5.257750787	1.825141432	-0.788328653
C	-3.397445307	2.562263772	0.089489242
C	-2.603431836	1.247088200	0.069943047
C	-3.255502523	0.009243934	0.082513463
H	-4.327657713	0.011902947	0.102552432
C	-2.609449473	-1.231679852	0.075706221
C	-3.410021329	-2.542663587	0.102294228
C	-4.927422959	-2.334504848	0.113656982
H	-5.269082089	-1.801181032	-0.774912256
H	-5.418345987	-3.309206069	0.128135123
H	-5.254192358	-1.782683462	0.996450219
C	-3.000340235	-3.316630516	1.369585716
H	-3.259823671	-2.754410020	2.269474960
H	-3.528303213	-4.271643597	1.400114256
H	-1.928643131	-3.511781182	1.379928133
C	-3.025698556	-3.356398659	-1.147557216
H	-1.954720446	-3.553416628	-1.172793390
H	-3.554955631	-4.311138548	-1.138178007
H	-3.302049233	-2.822337266	-2.059537748
O	-1.339523788	1.398044118	0.045654336
O	-1.346149139	-1.389102278	0.050526500

$Cu(hfac)_2$

Cu	0.000000000	0.000000000	0.000000000
F	-4.708213751	-2.340885926	0.019569072
F	-3.253584041	-3.055358482	-1.423851393
F	-2.947506994	-3.409867342	0.695055306
F	-4.700052146	2.353669621	-0.266947188
F	-3.283258162	3.102897469	1.196159444
F	-2.916726662	3.397622398	-0.922403912
O	-1.350132353	-1.399001245	-0.073693243
O	-1.349182277	1.405807763	0.002183290
C	-3.398423330	-2.516637478	-0.198381590
C	-2.591223350	-1.205249098	-0.108579041
C	-3.276206121	0.006244705	-0.102403488
H	-4.351780276	0.007528799	-0.139800588
C	-2.590388862	1.215814485	-0.048341701
C	-3.396194212	2.530737119	-0.016864728
O	1.350174003	1.399088075	0.071042746
C	2.591238419	1.205365010	0.107118116
C	3.398395199	2.516903080	0.195243527
F	4.708289580	2.340799782	-0.021801675
F	3.252970872	3.057650992	1.419773888
F	2.947909192	3.408613592	-0.699917626
C	3.276197162	-0.006157607	0.103632748
H	4.351750915	-0.007398165	0.141629215
C	2.590341735	-1.215802348	0.051797681
O	1.349145980	-1.405846063	0.001304738
C	3.396004032	-2.530884667	0.023357856
F	4.700055224	-2.353287813	0.272047386
F	3.282139750	-3.106406806	-1.187994296
F	2.917055429	-3.395187685	0.931649320

$Cu(hfac)_2 \bullet H_2O$

Cu	0.000000000	0.000000000	0.000000000
O	1.364290577	-1.411909605	0.073611591
O	1.356878225	1.412364633	0.190507746
C	2.602863816	-1.208018234	0.051879269
C	3.281222930	0.007336829	0.078829653
C	2.596347283	1.216638503	0.165020766
C	3.426321019	-2.512280911	0.021842132
F	4.726164770	-2.308007131	-0.231746106
F	2.951447663	-3.347489100	-0.915414068
F	3.333564981	-3.138238520	1.210651591
C	3.409378711	2.526217153	0.230767115
F	4.734374310	2.326641779	0.254728080
F	3.128855846	3.291265747	-0.840157961
F	3.080306434	3.226968251	1.329310756
O	-1.369284521	1.406417793	0.050822353
O	-1.360958987	-1.415860466	0.059341805
C	-2.605079227	1.203952545	-0.039446026
C	-3.282027080	-0.011256314	-0.093372234

C	-2.598169746	-1.222206468	-0.020875601
C	-3.425244295	2.510299588	-0.057961175
F	-2.934981339	3.359695723	-0.975155243
F	-4.722382795	2.315573346	-0.332194642
F	-3.345592509	3.115067344	1.142377914
C	-3.407539987	-2.535898190	-0.039164976
F	-4.733454333	-2.340796131	-0.055054014
F	-3.114257445	-3.276859990	1.042835052
F	-3.086369892	-3.259119836	-1.127488139
O	0.052130942	0.054104269	-2.337619965
H	4.357257783	0.010621244	0.056863821
H	-4.355586890	-0.014178225	-0.168812873
H	-0.117509880	0.951579949	-2.655474153
H	0.929835807	-0.170256772	-2.676233617

$Cu(hfac)_2 \cdot 2H_2O$

Cu	0.000000000	0.000000000	0.000000000
F	-4.725337332	-2.318454341	0.510248945
F	-3.024003957	-3.214381440	1.510357054
F	-3.179201067	-3.297118848	-0.654000287
F	-4.592119310	2.417589473	0.777742435
F	-3.867257233	2.649680579	-1.257135938
F	-2.805640872	3.580161335	0.389667135
F	4.718030321	2.369402623	-0.329266214
F	3.111555944	3.318953053	0.775759595
F	3.047729831	3.252877964	-1.393133687
F	4.728012541	-2.298521720	0.195176269
F	3.587879738	-2.844221425	-1.570098253
F	2.928994190	-3.490309684	0.393381070
O	-1.353849442	-1.419741171	0.222373616
O	-1.380443292	1.422481829	0.044166284
O	1.349274664	1.429326944	-0.179834677
O	1.391492841	-1.416154427	-0.104316783
O	-0.286626635	-0.131905748	-2.384085233
H	0.392686479	0.409450489	-2.808400597
H	-0.093688263	-1.037566020	-2.662354663
O	0.223122562	0.083460375	2.391290365
H	0.245040370	-0.822793391	2.727513919
H	1.077914577	0.461951184	2.637321941
C	-3.403084045	-2.520439473	0.422295812
C	-2.590272028	-1.215185005	0.303323226
C	-3.275340868	-0.005282892	0.283150220
H	-4.349327961	-0.007823599	0.364156757
C	-2.608972663	1.213453578	0.142340905
C	-3.481431030	2.485008764	0.026059635
C	3.390617402	2.554487692	-0.295760583
C	2.589329865	1.238274443	-0.226933362
C	3.283348728	0.032862348	-0.221422296
H	4.358888890	0.045511245	-0.269121388
C	2.621114782	-1.194845649	-0.174842379
C	3.483934766	-2.473119330	-0.277183263

$Cu(tbaa)_2 \cdot H_2O$

Cu	0.000000000	0.000000000	0.000000000
O	1.306539282	1.463793992	0.071069377
O	1.393870463	-1.353761491	0.106571746
O	3.337594526	2.418543849	-0.000888519
O	-0.003089606	0.287930690	-2.474249880
C	1.973746953	4.171028169	1.081712256
C	4.085515517	4.630537858	-0.180442425
C	2.040018986	3.929785172	-1.444533702
C	2.811098377	3.796276660	-0.136058325
C	2.554608324	1.336769402	0.040730496
C	3.267479631	0.114178395	0.051374524
C	2.657981028	-1.129210364	0.089965085
C	3.518943284	-2.365215435	0.110897655
H	1.041786288	3.614564821	1.116050626
H	1.743170637	5.236668070	1.034908132
H	2.536021589	3.984315638	1.998283706
H	3.826914006	5.684612970	-0.285571806
H	4.707258389	4.338532987	-1.027868623
H	4.658761464	4.502322437	0.738729205
H	1.790504563	4.980309262	-1.602360440
H	1.118077870	3.354736624	-1.428932184
H	2.655265225	3.595100118	-2.281378035
H	4.344634342	0.168532920	0.028419106
H	4.580562357	-2.126216771	0.099822588
H	3.279862077	-2.985990837	-0.755725475
H	3.289938897	-2.950678775	1.004258943
H	0.402329867	1.162020425	-2.551356927
O	-1.383917156	1.386115030	0.146744496
C	-2.619085974	1.195363040	0.035419294
O	-3.461323913	2.232156477	0.044397294
C	-3.011675997	3.641057173	0.124149073
C	-2.303896962	3.894469089	1.450586891
H	-1.352937636	3.372090309	1.502346577
H	-2.120190027	4.965181766	1.553710441
H	-2.934440896	3.574170174	2.281748783
C	-4.327224509	4.407358119	0.067555041
H	-4.128580452	5.478105683	0.122929819
H	-4.853620544	4.199042230	-0.865063083
H	-4.969235115	4.128982178	0.904443172
C	-2.141988959	3.982109854	-1.080990204
H	-1.960575199	5.058096488	-1.091244753
H	-1.185042115	3.469940853	-1.043676149
H	-2.654967686	3.712933216	-2.005890625
C	-3.260755607	-0.059772294	-0.097858797
C	-2.587132749	-1.269817329	-0.093542317
O	-1.315835737	-1.427802070	-0.006991573
H	-0.932354682	0.427861625	-2.700312109
C	-3.374421095	-2.550001205	-0.194326758
H	-4.445676249	-2.370322981	-0.261070166
H	-3.046239702	-3.107562115	-1.074725015
H	-3.166242043	-3.170800818	0.680083611
H	-4.336268711	-0.060088699	-0.181063746

Cu(acacen)

Cu	0.000000000	0.000000000	0.000000000
O	-1.361855167	-1.385714295	0.192719834
O	1.346827965	-1.404361903	-0.154328350
N	-1.321261674	1.443171238	-0.099888021
N	1.337060953	1.431039803	0.064851853
C	-2.629401034	-1.175924138	0.237192012
C	-2.620759269	1.307944084	-0.090072200
C	-0.673910975	2.724328761	-0.359872479
H	-1.251249648	3.568067401	0.020036531
H	-0.563407980	2.857321179	-1.442516418
C	-3.257210288	0.052869751	0.106750485
H	-4.336461933	0.058562892	0.142091710
C	2.617019345	-1.210594958	-0.194348275
C	0.703259664	2.725233499	0.291471496
H	1.289491859	3.553029732	-0.109396751
H	0.594282296	2.886735618	1.370402448
C	2.634956803	1.281120414	0.065110074
C	-3.466572713	-2.413879436	0.440935879
H	-3.263226742	-3.127704465	-0.361137180
H	-4.532255057	-2.192357451	0.461003431
H	-3.182747158	-2.892724949	1.381470709
C	-3.516285198	2.505030943	-0.298441929
H	-3.359840533	3.247850458	0.487097593
H	-4.562513400	2.209275541	-0.292359967
H	-3.294070658	2.989964818	-1.251517782
C	3.258106871	0.014179997	-0.094401099
H	4.337586018	0.006369288	-0.121288302
C	3.542844662	2.473103481	0.246834504
H	3.394866207	3.199775483	-0.555183091
H	4.585880987	2.166275601	0.248888551
H	3.324940278	2.981739900	1.188577595
C	3.441268354	-2.463591705	-0.353831706
H	3.223761663	-3.148955376	0.469108323
H	4.509566627	-2.254961778	-0.372784377
H	3.158818857	-2.969227109	-1.280656391

Cu(acacen) (Constrained Optimization)

Cu	0.000000000	0.000000000	0.000000000
O	-1.341095923	-1.423336687	0.000050241
O	1.342660344	-1.423336687	0.000050241
N	-1.338467772	1.418892356	-0.000050072
N	1.337632287	1.419661163	-0.000050179
C	-2.608147774	-1.238512818	0.106634625
C	-2.636221831	1.264227910	0.025863843
C	-0.715333241	2.711494080	-0.270205795
H	-1.263416430	3.540346826	0.180318202
H	-0.698921368	2.876598074	-1.353913824

C	-3.252052302	-0.010525268	0.138854496
H	-4.329459871	-0.023105314	0.209394090
C	2.609941900	-1.236460388	-0.102514528
C	0.713094849	2.713164475	0.263812818
H	1.261508213	3.540513094	-0.188977599
H	0.695061066	2.883380839	1.346759577
C	2.635488428	1.266437270	-0.022661466
C	-3.426842319	-2.502972504	0.183032382
H	-3.223973183	-3.120055485	-0.695572386
H	-4.494964576	-2.300693122	0.240076217
H	-3.125271540	-3.079846679	1.061027587
C	-3.549105549	2.460248505	-0.088382024
H	-3.345571485	3.181121344	0.706133855
H	-4.591494990	2.157591196	-0.028350596
H	-3.387327824	2.973157146	-1.039287874
C	3.252758817	-0.007885439	-0.133485017
H	4.330389562	-0.019243031	-0.200620926
C	3.546771832	2.463569177	0.093272954
H	3.347182294	3.182629153	-0.703903837
H	4.589800560	2.161878365	0.039741682
H	3.379233593	2.978708698	1.042026546
C	3.431247713	-2.499667214	-0.173126034
H	3.242498212	-3.105505520	0.716513100
H	4.497895618	-2.294902310	-0.246214963
H	3.119352037	-3.089079315	-1.038945536

$Cu(pci)_2$

Cu	0.000000000	0.000000000	0.000000000
C	2.687059648	0.763744061	-0.032662539
C	2.727761555	-0.653488822	-0.051618872
C	3.755273837	-1.606709112	-0.082552215
C	3.124489375	-2.859112093	-0.089448089
C	1.740615105	-2.612935550	-0.062673828
N	1.521699339	1.333231940	-0.006936255
N	1.503354671	-1.290165133	-0.039888932
N	-1.521698234	-1.333233625	0.006917412
N	-1.503355507	1.290163056	0.039899357
C	-2.687058890	-0.763747030	0.032653808
C	-2.727761773	0.653485695	0.051626007
C	-1.740616649	2.612933147	0.062692969
C	-3.755274515	1.606704999	0.082573231
C	-3.124490521	2.859108108	0.089500089
H	3.607505317	1.343561299	-0.039948483
H	4.815438133	-1.404245425	-0.097547951
H	3.598461372	-3.828245179	-0.110982250
H	0.932276803	-3.329417844	-0.059103689
H	-3.607504202	-1.343564844	0.039934754
H	-0.932278728	3.329415888	0.059123393
H	-4.815438570	1.404240371	0.097570208
H	-3.598462903	3.828240354	0.111061906
H	1.518136447	2.345050935	0.007023376
H	-1.518134454	-2.345052471	-0.007053998

$(PPh_4)_2[Cu(mnt)_2]$

Cu	0.000000000	0.000000000	0.000000000
N	5.205812486	-2.020822981	0.790584153
N	-5.345714241	1.766682034	0.371718848
N	5.330034952	1.803987333	-0.416358377
N	-5.187257802	-2.065725686	-0.800284682
S	1.559564178	-1.567796735	0.656704917
S	-1.676139691	1.542964842	0.387450133
S	1.662721356	1.555286085	-0.401403607
S	-1.544767829	-1.586132247	-0.649737962
C	3.036923842	-0.698215694	0.370166804
C	-3.083480200	0.583365008	0.038747857
C	4.240847747	-1.412654486	0.598308639
C	-4.337299260	1.222203280	0.215537623
C	3.078877619	0.607716488	-0.055754949
C	-3.029770351	-0.725068716	-0.378166849
C	4.326859112	1.254340931	-0.245215388
C	-4.227396701	-1.449019994	-0.609660118

$(PPh_4)_2[Cu(mnt)_2]$ (Constrained Optimization)

Cu	0.000000000	0.000000000	0.000000000
N	5.295872236	2.005155794	0.288117360
N	-5.296378824	-2.004238838	-0.284333515
N	5.289933826	-2.015246135	0.298219209
N	-5.289838319	2.015998216	-0.291175262
S	1.648810763	1.641985409	-0.000600982
S	-1.649028054	-1.642202264	0.000601076
S	1.640829699	-1.642202264	0.000601076
S	-1.640206198	1.642195566	-0.000139126
C	3.094170094	0.682910084	0.113219019
C	-3.094177288	-0.682549815	-0.111273763
C	4.315648677	1.396114641	0.209249728
C	-4.315980027	-1.395419235	-0.205894207
C	3.090620263	-0.689627327	0.115312038
C	-3.090385591	0.689990031	-0.113037713
C	4.309759712	-1.406678518	0.214428371
C	-4.309554863	1.407252242	-0.210038985

$Cu(dtc)_2$

Cu	0.000000000	0.000000000	0.000000000
C	-2.788851522	-0.036194771	-0.161102375
C	-4.892560614	1.191621654	-0.323801445
C	-4.876874489	-1.301409405	-0.224327734
C	-5.298275112	1.711510460	1.048942066
C	-5.123084789	-1.830293886	-1.631348867
H	-4.302094207	1.931868297	-0.859522763

H	-4.179413058	-2.056243821	-2.128727817
H	-5.770932679	0.966288443	-0.927866450
H	-4.336043939	-2.030385802	0.375576569
H	-5.819446098	-1.089498779	0.279598722
H	-5.897403209	2.616273513	0.934191782
H	-5.893998777	0.973566524	1.588529495
H	-4.417339211	1.953042181	1.644380089
H	-5.714808460	-2.745793841	-1.578447471
H	-5.671030723	-1.104099636	-2.233871304
N	-4.106203182	-0.048051156	-0.232894497
S	-1.854884361	1.421258678	-0.175918425
S	-1.833416513	-1.474780507	-0.044230281
S	1.847226133	-1.410128444	0.291713871
S	1.838462124	1.460900448	-0.108275295
C	2.788526158	0.033583595	0.129640160
N	4.106949889	0.043449682	0.180704653
C	4.888531312	-1.187153409	0.377092431
C	4.883827577	1.284476889	0.037869393
H	5.777900564	-0.911298780	0.943053628
H	4.303323916	-1.869066040	0.990247058
C	5.270831296	-1.837118993	-0.946347892
C	5.162245123	1.942827752	1.383301688
H	5.815096780	1.019933606	-0.462596765
H	4.334497935	1.956908957	-0.617394529
H	5.867757549	-2.730351830	-0.755581431
H	4.380067133	-2.128587233	-1.503752533
H	5.861500691	-1.156502523	-1.561574381
H	5.759020859	2.844060946	1.232330789
H	5.717047491	1.272513571	2.041405828
H	4.230241454	2.222719557	1.875462693

$Cu(dtc)_2$ (Constrained Optimization)

Cu	0.000000000	0.000000000	0.000000000
C	2.794855132	0.003280844	-0.017960372
C	4.889471903	1.258334365	-0.057787857
C	4.898864015	-1.235830094	-0.066598312
C	5.187005909	1.732430217	-1.474249187
C	5.261074139	-1.708844447	1.334941124
H	4.331517820	2.011725969	0.494223795
H	4.362138129	-1.928810792	1.911626524
H	5.812488716	1.062451964	0.487301625
H	4.323531913	-1.993024097	-0.595038573
H	5.796182240	-1.032295914	-0.650464230
H	5.782526646	2.645935980	-1.435481961
H	5.750100013	0.981121596	-2.030139303
H	4.261989013	1.944377148	-2.011299708
H	5.861494777	-2.617561998	1.269157632
H	5.841929826	-0.953539319	1.866616247
N	4.113825456	0.008171762	-0.044864376
S	1.843273557	1.449583874	-0.000267888
S	1.853100465	-1.449027729	0.000267716
S	-1.842566817	-1.449027729	0.000267716

S	-1.850145305	1.450034512	0.000295190
C	-2.793104856	-0.001773804	0.018441761
N	-4.112027869	-0.005646819	0.044949604
C	-4.888491206	-1.255291030	0.057586070
C	-4.896193773	1.238978750	0.066198620
H	-5.811389447	-1.058517909	-0.487346063
H	-4.331031682	-2.008833639	-0.494764188
C	-5.186029475	-1.729976095	1.473775224
C	-5.257280832	1.712118905	-1.335485181
H	-5.793732221	1.036022157	0.649862224
H	-4.320403612	1.995887590	0.594641946
H	-5.782150546	-2.643085397	1.434455754
H	-4.261076879	-1.942926228	2.010485476
H	-5.748594004	-0.978750779	2.030318899
H	-5.857311059	2.621100692	-1.269927594
H	-5.838213462	0.957158486	-1.867647330
H	-4.357846693	1.931758261	-1.911477589

$(PPh_4)_2[Cu(bdt)_2]$

Cu	0.000000000	0.000000000	0.000000000
S	-1.637570963	-1.596537496	0.363865392
S	-1.649976689	1.586487183	-0.349259707
C	-3.149671666	0.682426436	-0.131056778
C	-4.381349449	1.341407250	-0.255480285
H	-4.383456201	2.402740120	-0.474876766
C	-5.586508659	0.666702448	-0.111373213
H	-6.521531873	1.203404682	-0.216597747
C	-5.581214606	-0.699068374	0.167683223
H	-6.512044726	-1.240969006	0.282941881
C	-4.370740295	-1.366848324	0.298717152
H	-4.364241334	-2.428164895	0.518117606
C	-3.144297644	-0.700812658	0.161227514
S	1.640250818	1.595140366	0.357916757
C	3.145500618	0.700183472	0.140428003
C	3.148550894	-0.681728756	-0.158034975
S	1.647289326	-1.584853753	-0.368807698
C	4.379107520	-1.340375552	-0.294664252
H	4.379332867	-2.400697729	-0.518908797
C	5.585448310	-0.666581873	-0.156408591
H	6.519573119	-1.202928822	-0.271067068
C	5.582446391	0.697855178	0.129094517
H	6.514214312	1.239036209	0.240104648
C	4.373124797	1.365284576	0.272112788
H	4.368527098	2.425562896	0.496524317

$(PPh_4)_2[Cu(bdt)_2]$ (Constrained Optimization)

Cu	0.000000000	0.000000000	0.000000000
S	-1.658856767	-1.626332897	0.006417840
S	-1.658856767	1.626120869	0.006417840

C	-3.164155850	0.706560418	0.014840998
C	-4.392575617	1.382368589	0.025788653
H	-4.390394959	2.466127721	0.034529051
C	-5.600555262	0.696768702	0.026797320
H	-6.533512010	1.247162238	0.036397244
C	-5.600592407	-0.696931313	0.014278861
H	-6.533587631	-1.247333355	0.011167071
C	-4.392650673	-1.382549959	0.006765372
H	-4.390563562	-2.466308961	-0.002148586
C	-3.164161866	-0.706764121	0.009081745
S	1.658830788	1.626357753	-0.006371775
C	3.164148403	0.706784527	-0.009113960
C	3.164155491	-0.706546081	-0.013408996
S	1.658852177	-1.626116377	-0.006417563
C	4.392583050	-1.382379550	-0.022065567
H	4.390406814	-2.466150435	-0.028717591
C	5.600559297	-0.696761884	-0.023646765
H	6.533526859	-1.247164305	-0.031695784
C	5.600581181	0.696954708	-0.013968719
H	6.533576415	1.247362194	-0.011633190
C	4.392634524	1.382593201	-0.008065199
H	4.390528598	2.466364985	-0.001272633

7.3 Example TDDFT Input File

For calculating Cu(acac)$_2$ excited state energies in a dielectric model of a polar crystal:

```
! UKS B3LYP RIJCOSX def2-TZVP def2/J TIGHTSCF SlowConv CPCM D3BJ LARGEPRINT

%pal nprocs 8
end
%maxcore 10000

%tddft
nroots 50
DoNTO true
end

%coords
   CTyp xyz
   Charge 0
   Mult 2
   Units Angs
   Coords
Cu       0.000000000      0.000000000      0.000000000
O       -1.345761660     -1.403822316     -0.033930694
O        1.345767526      1.403836812      0.033517760
O       -1.341760961      1.407128016     -0.010799546
O        1.341769052     -1.407136854      0.010424731
C       -2.608649533     -1.234533453     -0.042990167
C        2.608661601      1.234544416      0.041697033
C       -2.605139078      1.241997651     -0.022435612
C        2.605152609     -1.241989819      0.021184117
H       -3.163626807     -3.084197710     -0.943279618
H        3.164177560      3.084360769      0.941336834
H       -3.139219669      3.120418380     -0.875495697
H        3.139781729     -3.120499005      0.873716045
C       -3.427566347     -2.496658480     -0.060984159
C       -3.257149594      0.004719332     -0.037459419
C        3.257160998     -0.004707092      0.035866914
C        3.427591641      2.496671656      0.058983920
C       -3.419990457      2.506739625     -0.016703429
C        3.420013143     -2.506725264      0.014814948
H       -3.179967315     -3.097878739      0.816823541
H       -4.496175828     -2.293013944     -0.069319525
H       -4.336507674      0.006564474     -0.046812125
H        4.336525569     -0.006545122      0.044460008
H        3.179470034      3.097743898     -0.818777338
H        4.496205996      2.293028300      0.066719387
H       -4.488913529      2.307001693     -0.048246310
H       -3.185926364      3.079796722      0.883582851
H        3.185393993     -3.079687104     -0.885386579
H        4.488954826     -2.306984574      0.045720570

end
end
```

7.4 Explicit Solvation Computations

All solvation computations were performed using Orca 5.0.3 or Orca 6.0.0.[19] DFT calculations were conducted with the def2-TZVP basis set, and the B3LYP functional was adjusted to include 50% Hartree-Fock exchange (HFX), which has the effect of bringing the calculated g values closer to those observed experimentally via CW EPR. DFT calculations also incorporated the RIJCOSX approximation, the def2/J general auxiliary basis set, the D3BJ dispersion correction, and the %eprnmr block. Within the %eprnmr block, the isotropic (aiso), dipolar (adip), and spin-orbit coupling (aorb) contributions to the hyperfine tensor were calculated for copper, nitrogen (when applicable), and sulfur (when applicable). With the exceptions of $[Cu(mnt)_2]^{2-}$ and $Cu(dtc)_2$, geometry optimizations were performed with all atoms free to move. For $[Cu(mnt)_2]^{2-}$ and $Cu(dtc)_2$, the transverse S—Cu—S bond angles were restricted to 180° to prevent distortions from square planar geometry.

For each compound, an initial structure was manually drawn in Avogadro and optimized with the UFF force field. The structure was then optimized in the gas phase (no solvent, designated calculation **A**). A frequency calculation was performed after the optimization to obtain the Gibbs free energy and to verify that the optimized structure represented a minimum on the potential energy surface. The output of **A** was then used as the initial guess for an implicit-solvation optimization (CPCM, solvent = Water, Methanol, or Toluene, calculation **B**). Methanol was chosen to model alcohol groups in the polar PVA polymer, while toluene was chosen to model the nonpolar PS polymer. The output of **B** became the starting guess for two strategies of explicit solvation. The first strategy (**Method 1,** calculation **C**) utilized the recently released ORCA SOLVATOR for placement of 7 (for water and methanol) or 4 (toluene) solvent molecules via the XTB semi-empirical model.[21] The output of **C** became the input geometry for an additional B3LYP/def2-TZVP + CPCM optimization (calculation **D**). By including both explicit solvent molecules and the CPCM model, **D** accounts for both short-range coordination and hydrogen-bonding interactions, as well as long-range polarization contributions. The output of **D** became the input geometry for TDDFT calculations of d-d transition energies and absorption spectra.

The second method of explicit solvation (**Method 2**) began with manual addition of two solvent molecules in the axial coordination positions. The remaining solvent molecules (5 for water/methanol, 2 for toluene) were then added using the ORCA SOLVATOR (calculation **E**) and re-optimized at B3LYP/def2-TZVP with CPCM (calculation **F**). The purpose of **Method 2** was to test the reliability of the SOLVATOR as a tool for probing solvent coordination. Greater confidence was given to the results when both methods yielded the same number of coordinated solvent molecules after calculations **D** and **F** – as they did for 9 of the 11 qubit/solvent combinations (see **Table S77**). For the two outliers ($Cu(acac)_2$ and $Cu(tmhd)_2$), the Gibbs free energies of the **D** and **F** structures provided insight into their relative stability. In the case of $Cu(acac)_2$ with explicit water solvation, the singly-coordinated structure (from **D**) was nearly isoenergetic to the doubly-coordinated structure (from **F**), with $\Delta G = 0.7$ kJ/mol. This result indicated that at least one axially coordinated H_2O was favorable for $Cu(acac)_2$, with the second being less certain. The other outlier, $Cu(tmhd)_2$, displayed a minimal difference ($\Delta G = 1.3$ kJ/mol) between non-coordinated and doubly-coordinated structures, suggesting that water-coordination, while energetically accessible, was not particularly favorable.

TDDFT calculations to obtain natural transition orbitals and d-d transition energies were performed as described earlier in **SI Section 7.1**. Graphs of Δg_z vs. solvent coordination were prepared using Veusz. Visualizations of optimized geometries from **D** and **F** were prepared in ChemCraft.

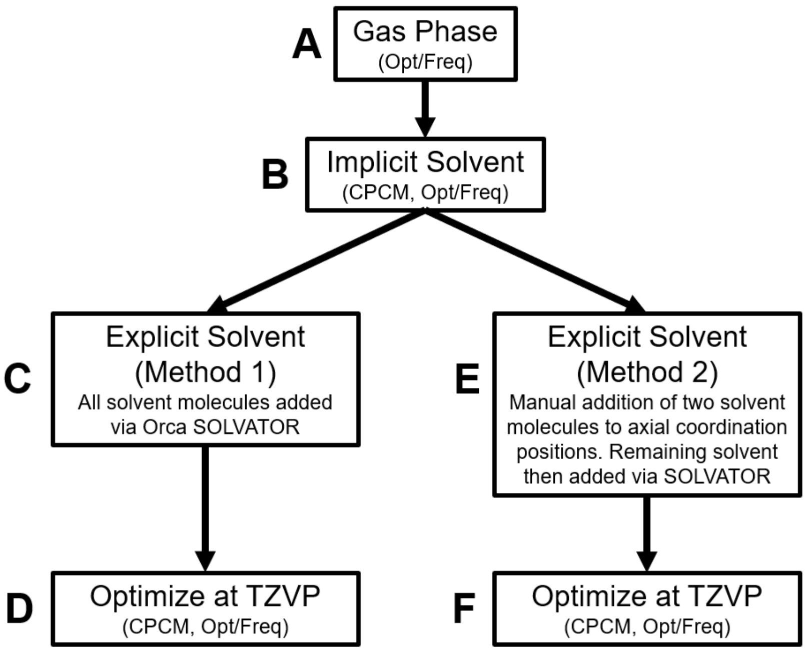

Figure S141: Sequence of DFT and SOLVATOR calculations for each copper complex.

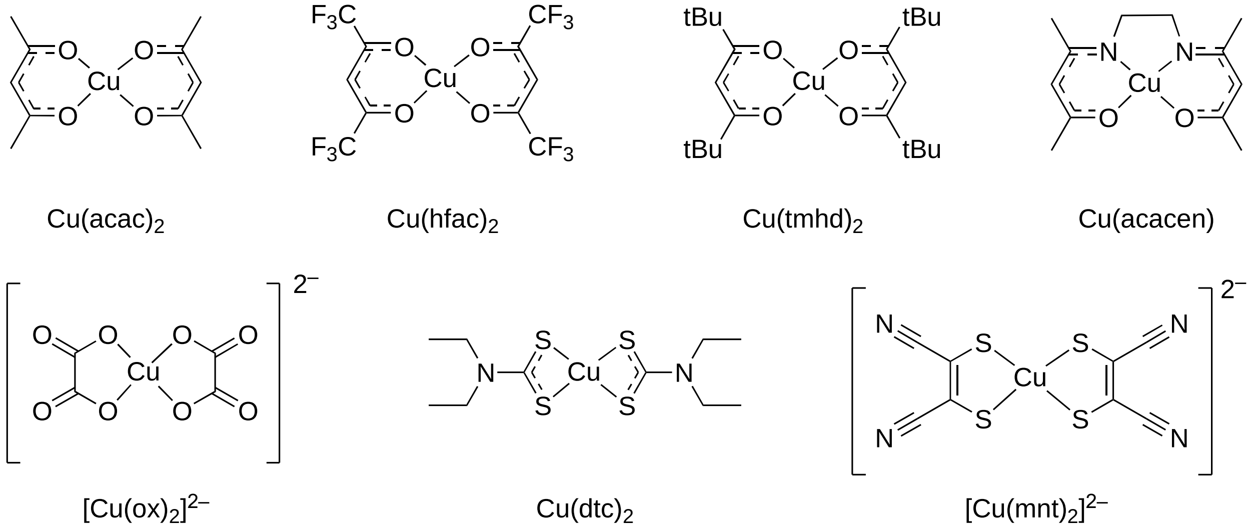

Figure S142: Set of copper complexes analyzed under explicit solvation conditions: H_2O → All seven complexes; MeOH → Cu(hfac)$_2$ and [Cu(ox)$_2$]$^{2-}$; Toluene → Cu(hfac)$_2$ and [Cu(ox)$_2$]$^{2-}$.

7.4.1 Computed g values and Free Energies

Table S70: g values, Gibbs free energies (GFE), and solvent coordination values for Cu(acac)₂.

**Cu(acac)$_2$**	g_{iso}	g_x	g_y	g_z	GFE (Ha)	# Coord
(A) Gas Phase	2.1383	2.0715	2.0730	2.2703	-2334.3690	N/A
(B) Implicit	2.1464	2.0780	2.0789	2.2823	-2334.3923	N/A
(D) – 7 H_2O	2.1577	2.0824	2.0906	2.3002	-2871.0748	1
(F) – 7 H_2O	2.1632	2.0876	2.0941	2.3079	-2871.0751	2

Table S71: g values, Gibbs free energies (GFE), and solvent coordination values for Cu(hfac)$_2$.

**Cu(hfac)$_2$**	g_{iso}	g_x	g_y	g_z	GFE (Ha)	# Coord
(A) Gas Phase	2.1432	2.0749	2.0755	2.2791	-3528.4832	N/A
(B) Implicit	2.1596	2.0872	2.0877	2.3039	-3528.5098	N/A
(D) – 7 H_2O	2.1827	2.1006	2.1110	2.3364	-4065.1951	2
(F) – 7 H_2O	2.1822	2.1030	2.1068	2.3367	-4065.1960	2
(D) – 7 MeOH	2.1834	2.0960	2.1169	2.3375	-4341.2386	2
(F) – 7 MeOH	2.1796	2.0962	2.1100	2.3327	-4341.2433	2
(D) – 4 Toluene	2.1536	2.0826	2.0829	2.2952	-4618.7436	0
(F) – 4 Toluene	2.1532	2.0824	2.0826	2.2946	-4618.7494	0

Table S72: g values, Gibbs free energies (GFE), and solvent coordination values for Cu(tmhd)$_2$.

**Cu(tmhd)$_2$**	g_{iso}	g_x	g_y	g_z	GFE (Ha)	# Coord
(A) Gas Phase	2.1364	2.0708	2.0715	2.2669	-2807.8071	N/A
(B) Implicit	2.1432	2.0761	2.0764	2.2771	-2807.8266	N/A
(D) – 7 H_2O	2.1421	2.0750	2.0762	2.2750	-3344.5049	0
(F) – 7 H_2O	2.1552	2.0823	2.0860	2.2972	-3344.5054	2

Table S73: g values, Gibbs free energies (GFE), and solvent coordination values for Cu(acacen)$_2$.

**Cu(acacen)$_2$**	g_{iso}	g_x	g_y	g_z	GFE (Ha)	# Coord
(A) Gas Phase	2.1189	2.0628	2.0633	2.2305	-2372.2059	N/A
(B) Implicit	2.1218	2.0654	2.0657	2.2342	-2372.2263	N/A
(D) – 7 H_2O	2.1237	2.0662	2.0677	2.2373	-2908.9125	0
(F) – 7 H_2O	2.1227	2.0663	2.0666	2.2353	-2908.9125	0

Table S74: g values, Gibbs free energies (GFE), and solvent coordination values for Cu(dtc)$_2$.

**Cu(dtc)$_2$**	g_{iso}	g_x	g_y	g_z	GFE (Ha)	# Coord
(A) Gas Phase	2.0887	2.0460	2.0471	2.1730	-3741.0558	N/A
(B) Implicit	2.0958	2.0504	2.0513	2.1858	-3741.0768	N/A
(D) – 7 H_2O	2.0973	2.0508	2.0526	2.1884	-4277.7599	0
(F) – 7 H_2O	2.0972	2.0512	2.0521	2.1882	-4277.7588	0

Table S75: g values, Gibbs free energies (GFE), and solvent coordination values for [Cu(mnt)$_2$]$^{2-}$.

**[Cu(mnt)$_2$]$^{2-}$**	g_{iso}	g_x	g_y	g_z	GFE (Ha)	# Coord
(A) Gas Phase	2.1050	2.0556	2.0588	2.2006	-3762.4122	N/A
(B) Implicit	2.1048	2.0560	2.0588	2.1996	-3762.6273	N/A
(D) – 7 H_2O	2.1037	2.0551	2.0580	2.1979	-4299.3151	0
(F) – 7 H_2O	2.1028	2.0548	2.0571	2.1966	-4299.3115	0

Table S76: g values, Gibbs free energies (GFE), and solvent coordination values for [Cu(ox)$_2$]$^{2-}$.

**[Cu(ox)$_2$]$^{2-}$**	g_{iso}	g_x	g_y	g_z	GFE (Ha)	# Coord
(A) Gas Phase	2.1356	2.0700	2.0718	2.2648	-2398.3340	N/A
(B) Implicit	2.1434	2.0766	2.0782	2.2755	-2398.6140	N/A
(D) – 7 H_2O	2.1603	2.0842	2.0958	2.3011	-2935.3005	1
(F) – 7 H_2O	2.1588	2.0878	2.0890	2.2998	-2935.2982	1
(D) – 7 MeOH	2.1594	2.0851	2.0929	2.3003	-3211.3405	2
(F) – 7 MeOH	2.1596	2.0821	2.0960	2.3006	-3211.3391	2
(D) – 4 Toluene	2.1396	2.0736	2.0751	2.2702	-3488.7447	0
(F) – 4 Toluene	2.1400	2.0738	2.0754	2.2707	-3488.7449	0

7.4.2 Comparison of Methods of Explicit Solvation

Table S77: Solvent coordination comparison for methods 1 (D) and 2 (F).

	# Coordinated Method 1 (D)	# Coordinated Method 2 (F)	Methods Agree?
$Cu(acac)_2$ – 7 H_2O	1	2	No
$Cu(hfac)_2$ – 7 H_2O	2	2	Yes
$Cu(hfac)_2$ – 7 MeOH	2	2	Yes
$Cu(hfac)_2$ – 7 Toluene	0	0	Yes
$Cu(tmhd)_2$ – 7 H_2O	0	2	No
$Cu(acacen)_2$ – 7 H_2O	0	0	Yes
$Cu(dtc)_2$ – 7 H_2O	0	0	Yes
$[Cu(mnt)_2]^{2-}$ – 7 H_2O	0	0	Yes
$[Cu(ox)_2]^{2-}$ – 7 H_2O	1	1	Yes
$[Cu(ox)_2]^{2-}$ – 7 MeOH	2	2	Yes
$[Cu(ox)_2]^{2-}$ – 7 Toluene	0	0	Yes

7.4.3 Changes in g_z vs. Solvent Coordination

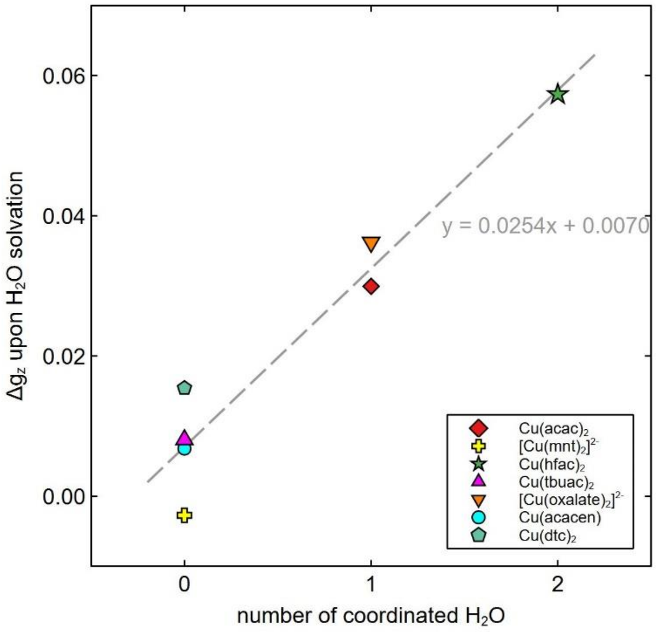

Figure S143: Change in g_z vs. number of coordinated water molecules.
Δg_z for zero coordinated water molecules was computed by subtracting the g_z value computed in the gas phase from the g_z value computed with explicit solvation (method 1), with no water molecules axially bound.

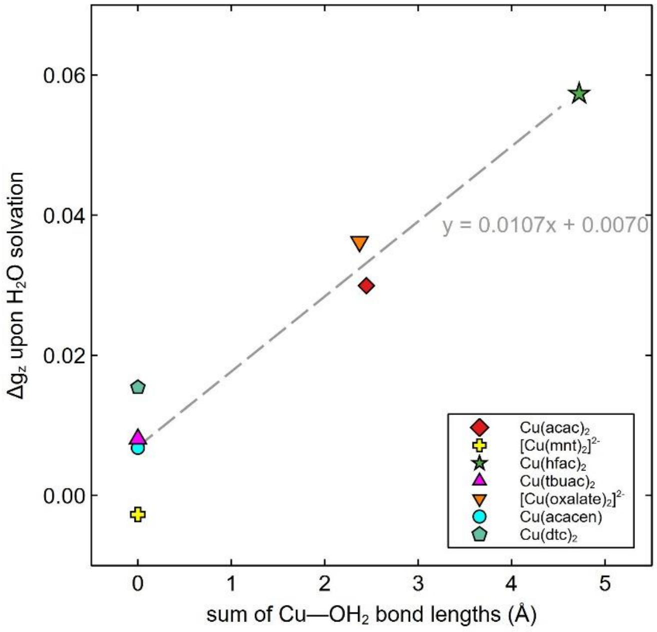

Figure S144: Change in g_z vs. sum of Cu–OH_2 bond lengths.

7.5 Computed Excited State Energies and Absorbance Spectra

7.5.1 $Cu(acac)_2 + 7H_2O$

Table S78: TDDFT-calculated [$Cu(acac)_2 + 7H_2O$] excited states assigned to d-d transitions.

State	NTO Occupation Number	Energy (cm^{-1})	Assignment
1	0.99514543	13407.2	$z^2 \rightarrow xy$
2	0.99827748	14283.7	$xz \rightarrow xy$
3	0.99917307	17179.0	$x^2–y^2 \rightarrow xy$
4	0.99810837	17420.1	$yz \rightarrow xy$

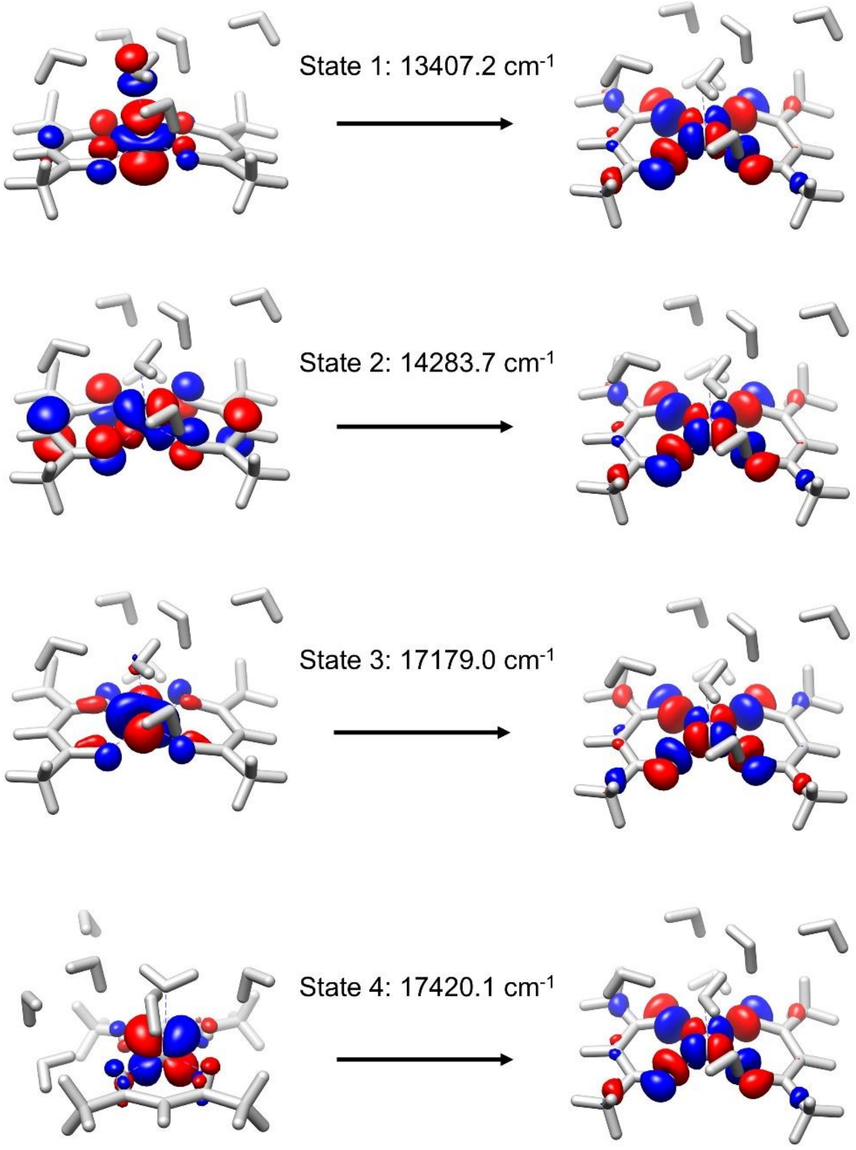

Figure S145: TDDFT natural transition orbitals for [$Cu(acac)_2 + 7H_2O$].

Table S79: TDDFT-calculated [Cu(acac)$_2$ + 7H_2O] UV-vis-NIR absorption spectrum.

Calculated via transition electric dipole moments, including oscillator strength (f_{osc}), net squared transition electric dipole moment (D^2), and transition electric dipole moment vector components.

State	Energy (cm^{-1})	Wavelength (nm)	f_{osc}	D^2 (a.u.^{2})	D_x (a.u.)	D_y (a.u.)	D_z (a.u.)
1	13407.2	745.9	3.96E-05	0.00097	-0.02713	0.01188	0.00977
2	14283.7	700.1	9.39E-05	0.00216	-0.01822	0.04279	0.00091
3	17179	582.1	2.78E-06	0.00005	0.00611	-0.00399	-0.00016
4	17420.1	574.1	0.001653	0.03124	-0.17661	-0.00356	0.00604
5	22432.3	445.8	0.000129	0.0019	0.02076	0.03818	-0.00336
6	24285.8	411.8	0.000238	0.00323	-0.02384	-0.05155	0.00258
7	26377.1	379.1	1.38E-06	0.00002	0.0041	-0.00055	-0.00029
8	26874.8	372.1	1.03E-05	0.00013	0.00203	0.01105	-0.00005
9	28313.1	353.2	0.260051	3.02376	1.73527	0.02531	-0.10926
10	31358	318.9	0.000451	0.00474	-0.06755	-0.00833	-0.0103
11	32063.2	311.9	0.0015	0.0154	0.11568	0.00879	0.04405
12	32821.9	304.7	0.004989	0.05004	-0.04839	-0.21823	0.00879
13	35026.2	285.5	0.001429	0.01343	0.00575	0.11542	-0.00898
14	35577.6	281.1	0.000127	0.00118	0.00087	0.03432	-0.00001
15	36829.5	271.5	0.000932	0.00833	0.06878	0.00536	0.05974
16	37066.8	269.8	0.002208	0.01961	-0.07652	0.03473	0.11203
17	37312.7	268	0.00344	0.03035	-0.06466	-0.16104	0.01533
18	37923.3	263.7	0.001055	0.00916	-0.04335	-0.01961	0.08305
19	38419.5	260.3	0.002196	0.01881	-0.01887	0.13347	0.02539
20	38718.9	258.3	0.00283	0.02406	0.00831	-0.15486	-0.00356
21	39216.6	255	4.96E-05	0.00042	0.01183	0.01656	-0.00144
22	40612.6	246.2	0.000148	0.0012	0.00541	0.03336	-0.00783
23	40892.5	244.5	0.02115	0.17027	0.00577	0.40979	-0.04806
24	40938.1	244.3	0.005025	0.04041	-0.03402	0.18497	0.07098
25	41359.3	241.8	0.000185	0.00147	-0.02644	0.02272	0.01599

7.5.2 $Cu(hfac)_2 + 7H_2O$

Table S80: TDDFT-calculated [$Cu(hfac)_2 + 7H_2O$] excited states assigned to d-d transitions.

State	NTO Occupation Number	Energy (cm^{-1})	Assignment
1	0.99555201	7103.6	$z^2 \rightarrow xy$
2	0.99881041	12199.9	$xz \rightarrow xy$
3	0.99843160	14772.5	$yz \rightarrow xy$
4	0.99901798	15360.7	$x^2–y^2 \rightarrow xy$

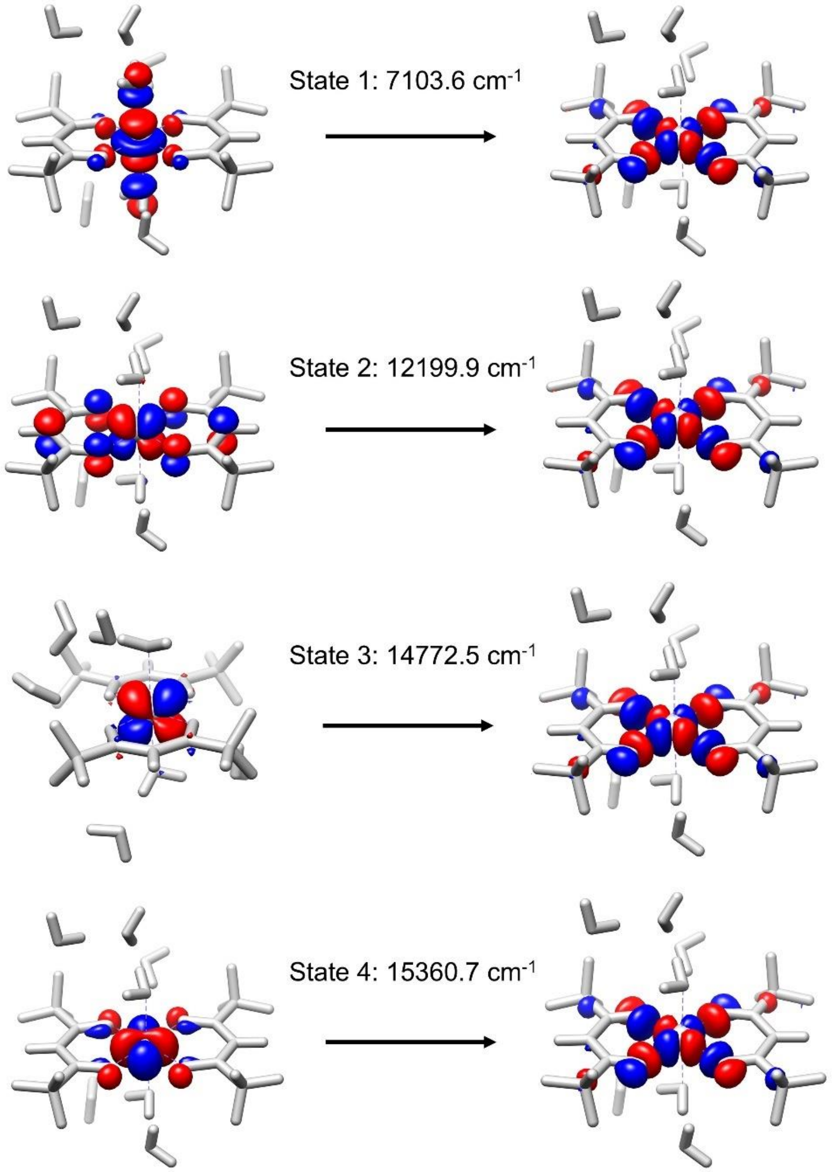

Figure S146: TDDFT natural transition orbitals for [$Cu(hfac)_2 + 7H_2O$].

Table S81: TDDFT-calculated [Cu(hfac)₂ + 7H₂O] UV-vis-NIR absorption spectrum.

Calculated via transition electric dipole moments, including oscillator strength (f_{osc}), net squared transition electric dipole moment (D^2), and transition electric dipole moment vector components.

State	Energy (cm^{-1})	Wavelength (nm)	f_{osc}	D^2 (a.u.^{2})	D_x (a.u.)	D_y (a.u.)	D_z (a.u.)
1	7103.6	1407.7	4.08E-06	0.00019	0.00835	0.00359	-0.01032
2	12199.9	819.7	1.09E-05	0.00029	-0.01625	-0.00539	0.00057
3	14772.5	676.9	3.06E-06	0.00007	0.0074	0.00366	-0.00032
4	15360.7	651	3.34E-06	0.00007	-0.00776	-0.00258	0.00216
5	21880	457	2.48E-05	0.00037	-0.01834	0.00231	0.00562
6	22930.2	436.1	4E-07	0.00001	0.00166	0.00162	-0.0006
7	23000.6	434.8	1.23E-05	0.00018	-0.00673	0.01142	0.00023
8	23498.4	425.6	0.0002	0.00281	-0.05269	0.00543	0.00089
9	29508.7	338.9	0.011505	0.12835	0.35796	0.01484	-0.0011
10	29633.1	337.5	0.242291	2.69175	-1.63989	-0.04874	0.01159
11	29947.2	333.9	0.00022	0.00242	0.01485	0.00078	0.04685
12	32473.5	307.9	0.000303	0.00307	0.01206	-0.05388	0.00475
13	32927.9	303.7	0.018432	0.18428	-0.0235	0.42863	-0.00295
14	33134.6	301.8	0.003635	0.03611	-0.00439	-0.18996	0.00317
15	33332.6	300	1.57E-05	0.00016	-0.00502	-0.00889	0.00715
16	33501.6	298.5	9.98E-06	0.0001	0.00347	-0.0092	0.0012
17	34028.2	293.9	0.000394	0.00382	0.01226	-0.05936	-0.01194
18	34281.1	291.7	5.51E-05	0.00053	0.00752	-0.02147	-0.0034
19	34907.9	286.5	0.001923	0.01814	0.05495	-0.12173	0.01726
20	35086.5	285	0.000338	0.00318	0.02258	-0.05098	0.00815
21	35255.4	283.6	0.00144	0.01345	-0.00155	0.00573	0.1158
22	35465.6	282	9.46E-05	0.00088	0.01672	0.02431	0.00273
23	35509	281.6	0.000137	0.00127	-0.03136	0.01209	0.01202
24	36093.5	277.1	0.001752	0.01598	-0.01101	0.12435	0.01996
25	36321.3	275.3	0.002538	0.023	-0.0211	0.14996	0.00819
26	37088.6	269.6	2.58E-05	0.00023	-0.01057	-0.01023	0.00351
27	37292.7	268.1	0.002587	0.02284	0.0046	-0.14755	0.03236
28	37780.7	264.7	0.00312	0.02718	0.02197	-0.16333	-0.00517
29	38180.4	261.9	0.002252	0.01942	-0.05894	-0.12624	0.00181
30	39002.7	256.4	0.000333	0.00281	0.01113	-0.05101	-0.00929
31	39538.1	252.9	0.001121	0.00933	0.02586	-0.09308	-0.00102
32	39845	251	0.000667	0.00551	-0.00872	0.01321	-0.07255
33	40583.5	246.4	0.000276	0.00224	-0.01565	0.02505	0.03698
34	41395.1	241.6	0.001263	0.01004	0.0108	-0.09949	-0.00526
35	42128.7	237.4	0.007595	0.05935	-0.00568	0.24341	-0.00839

7.5.3 Cu(tmhd)$_2$ + 7H$_2$O

Table S82: TDDFT-calculated [Cu(tmhd)$_2$ + 7H$_2$O] excited states assigned to d-d transitions.

State	NTO Occupation Number	Energy (cm^{-1})	Assignment
1	0.99826903	15367.6	xz → xy
2	0.99361441	17140.1	z^2 / x^2–y^2 → xy
3	0.99863145	19184.8	z^2 / x^2–y^2 → xy
4	0.99707246	19549.7	yz → xy

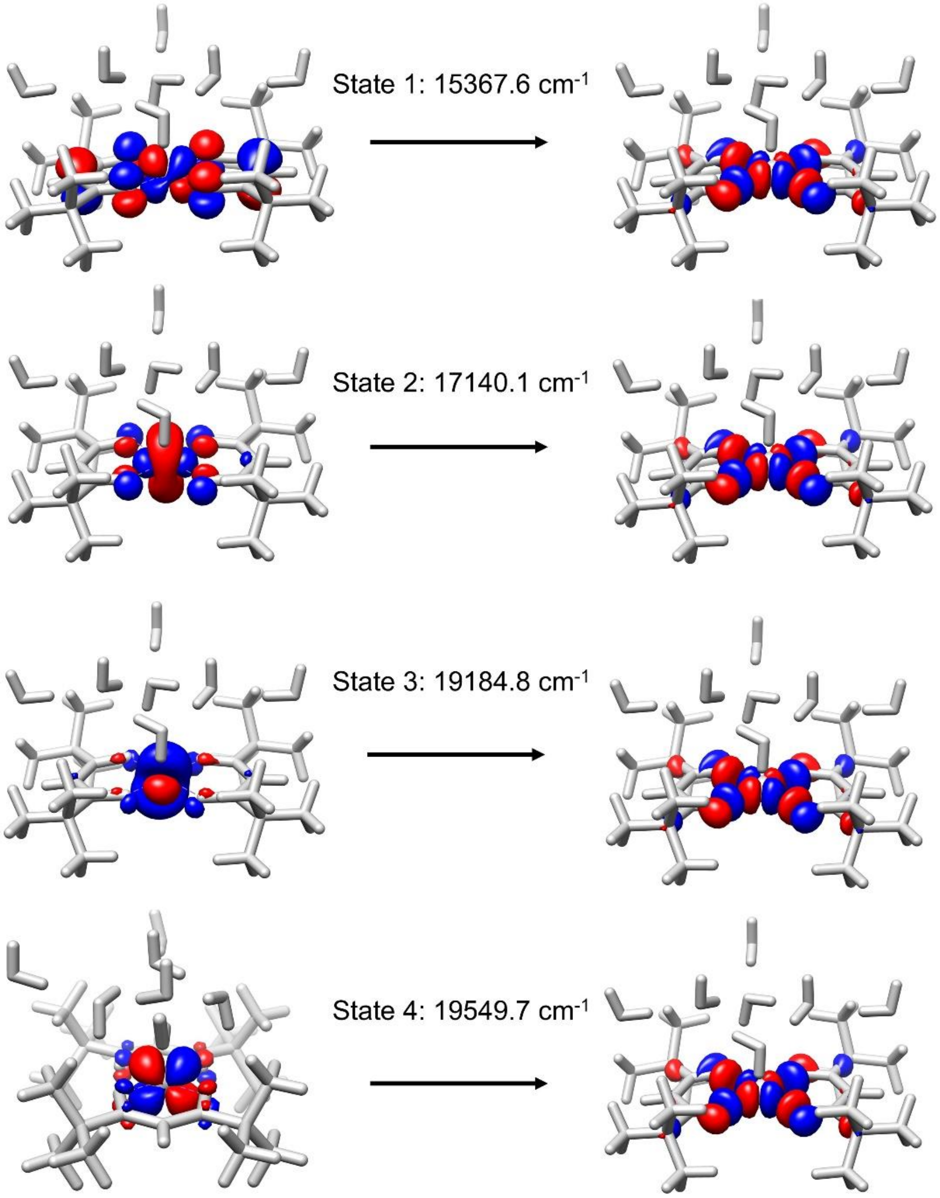

Figure S147: TDDFT natural transition orbitals for [Cu(tmhd)$_2$ + 7H$_2$O].

Table S83: TDDFT-calculated [Cu(tmhd)$_2$ + 7H_2O] UV-vis-NIR absorption spectrum.

Calculated via transition electric dipole moments, including oscillator strength (f_{osc}), net squared transition electric dipole moment (D^2), and transition electric dipole moment vector components.

State	Energy (cm^{-1})	Wavelength (nm)	f_{osc}	D^2 (a.u.^{2})	D_x (a.u.)	D_y (a.u.)	D_z (a.u.)
1	15367.6	650.7	3.35E-05	0.00072	-0.00854	-0.01962	-0.01613
2	17140.1	583.4	2.24E-05	0.00043	0.01538	-0.00721	-0.01187
3	19184.8	521.2	5.19E-06	0.00009	-0.00873	-0.00308	-0.00184
4	19549.7	511.5	0.000103	0.00173	-0.04075	-0.00409	0.00749
5	23064.7	433.6	0.000219	0.00312	0.03145	0.03801	0.02623
6	25456.1	392.8	0.000109	0.00141	-0.0073	-0.02822	-0.02359
7	26257	380.9	3.32E-05	0.00042	-0.00222	0.01475	0.0139
8	26416.5	378.6	1.55E-05	0.00019	-0.01283	0.00204	0.00494
9	27403.2	364.9	0.296584	3.56305	1.86532	0.20502	-0.20394
10	29672	337	0.002671	0.02964	-0.17207	-0.00498	-0.00209
11	30429.9	328.6	0.001468	0.01588	0.10353	-0.04451	0.05637
12	32256.9	310	0.001796	0.01833	0.03719	0.10062	0.08262
13	35210.4	284	0.001066	0.00997	-0.01658	-0.09284	-0.03273
14	35394.7	282.5	0.003659	0.03403	0.09258	0.07925	-0.13849
15	35873.3	278.8	0.002216	0.02033	0.07458	0.09389	-0.07717
16	36218.4	276.1	0.000128	0.00116	0.00331	-0.00393	-0.03366
17	36350.4	275.1	0.001148	0.01039	0.08275	-0.04513	0.03887
18	36868.2	271.2	0.003052	0.02725	-0.01729	0.11256	0.11952
19	37625.7	265.8	8.85E-05	0.00077	0.00457	0.01555	0.02261
20	38073.2	262.7	0.0022	0.01902	-0.00504	-0.10233	-0.09232
21	39835.4	251	0.003292	0.0272	-0.07977	-0.10058	0.10356
22	40761.8	245.3	0.008896	0.07185	-0.00668	-0.19928	-0.17914
23	40957.3	244.2	0.001748	0.01405	0.00034	-0.08956	-0.07763
24	41140.2	243.1	0.000821	0.00657	0.04177	-0.03616	0.05928
25	42133.3	237.3	0.002603	0.02034	-0.04708	-0.11996	-0.06108

7.5.4 Cu(acacen) + 7H_2O

Table S84: TDDFT-calculated [Cu(acacen) + 7H_2O] excited states assigned to d-d transitions.

State	NTO Occupation Number	Energy (cm^{-1})	Assignment
1	0.99761920	15235.6	xz → xy
2	0.99345098	19634.8	z^2 → xy
3	0.99428377	20471.0	yz → xy
4	0.99767971	21012.9	$x^2–y^2$ → xy

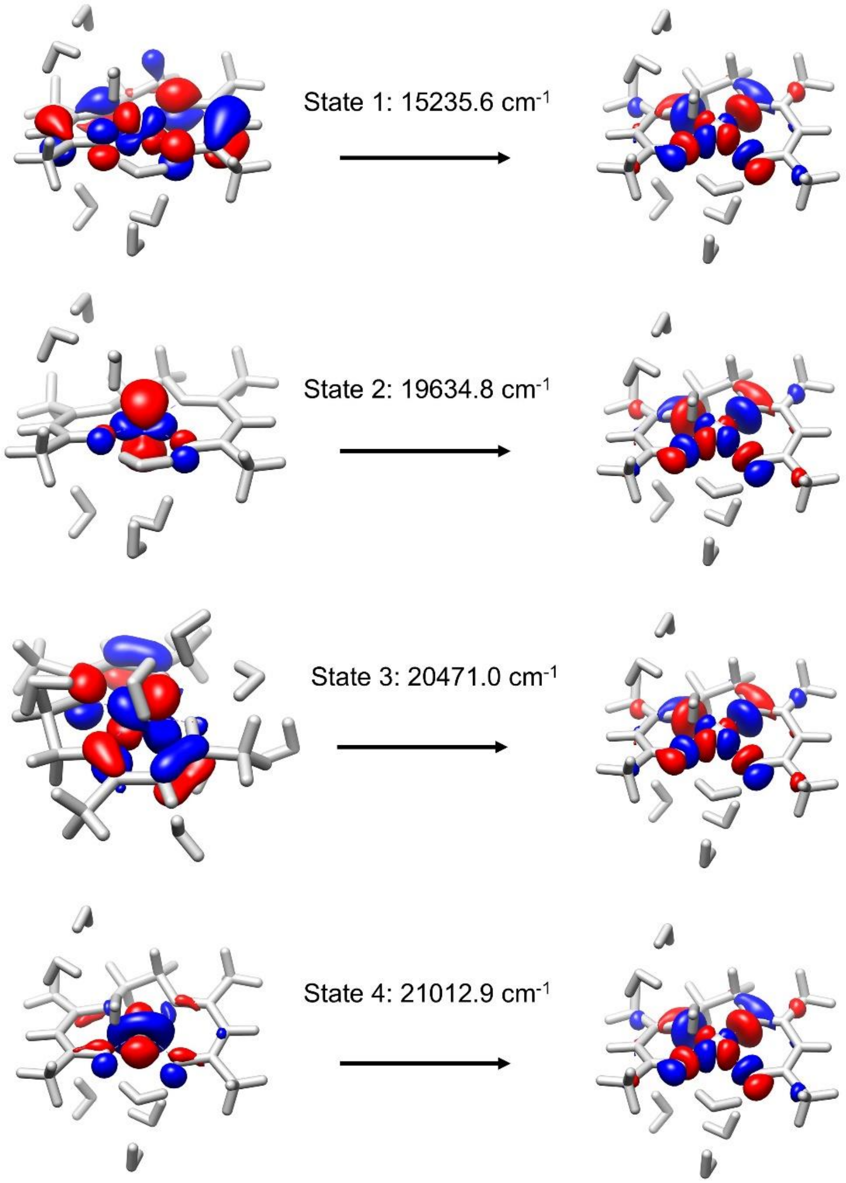

Figure S148: TDDFT natural transition orbitals for [Cu(acacen) + 7H_2O].

Table S85: TDDFT-calculated [Cu(acacen) + 7H_2O] UV-vis-NIR absorption spectrum.

Calculated via transition electric dipole moments, including oscillator strength (f_{osc}), net squared transition electric dipole moment (D^2), and transition electric dipole moment vector components.

State	Energy (cm^{-1})	Wavelength (nm)	f_{osc}	D^2 (a.u.^{2})	D_x (a.u.)	D_y (a.u.)	D_z (a.u.)
1	15235.6	656.4	0.00041	0.00887	0.09408	-0.00052	0.0038
2	19634.8	509.3	0.00096	0.0161	0.11906	-0.0109	-0.04248
3	20471	488.5	0.000524	0.00843	-0.09127	0.00865	-0.00459
4	21012.9	475.9	0.006857	0.10743	0.3275	-0.00684	0.01156
5	22712.3	440.3	0.000244	0.00354	0.03945	-0.04376	0.00816
6	25033.8	399.5	0.000709	0.00933	-0.09642	0.00548	-0.00197
7	25490.7	392.3	0.002197	0.02838	0.16511	-0.03326	-0.00293
8	26091	383.3	0.00347	0.04379	-0.20848	0.01788	0.0017
9	30354.4	329.4	0.000173	0.00187	-0.04192	-0.00997	-0.00408
10	31743	315	0.000332	0.00344	0.04603	-0.03628	-0.00265
11	32890.9	304	0.002698	0.027	0.04404	-0.08414	0.1341
12	33562	298	0.144039	1.41289	1.13992	0.33351	0.04729
13	33966	294.4	0.008218	0.07965	-0.27596	-0.05857	0.00828
14	34877.4	286.7	0.153905	1.45272	-0.89639	0.79587	0.12569
15	36179.8	276.4	0.023503	0.21386	-0.38177	-0.22614	0.13028
16	36979.5	270.4	0.001156	0.01029	0.09582	0.00649	-0.03264
17	38680.1	258.5	0.040737	0.34672	-0.52041	-0.10164	0.25605
18	40533.3	246.7	0.013272	0.1078	0.3011	-0.12844	-0.02521
19	40549	246.6	0.019502	0.15833	-0.27299	0.27218	0.09864
20	41132.2	243.1	0.03933	0.31479	0.09631	0.55086	-0.04539
21	41294.7	242.2	0.062165	0.4956	-0.11576	-0.69313	-0.04207
22	41686.5	239.9	0.042764	0.33772	-0.08226	-0.56953	0.0812
23	41929.8	238.5	0.072654	0.57044	0.03859	0.74104	0.14074
24	42280.7	236.5	0.003116	0.02426	0.00057	-0.15561	-0.00669
25	42878.1	233.2	0.013919	0.10687	-0.07018	0.30552	0.09276

7.5.5 $Cu(dtc)_2 + 7H_2O$

Table S86: TDDFT-calculated [$Cu(dtc)_2 + 7H_2O$] excited states assigned to d-d transitions.

State	NTO Occupation Number	Energy (cm^{-1})	Assignment
1	0.99749848	15627.0	yz → xy
2	0.99660720	17156.3	xz → xy
3	0.99558547	19164.9	z^2 → xy
6	0.99860276	22026.7	x^2–y^2 → xy

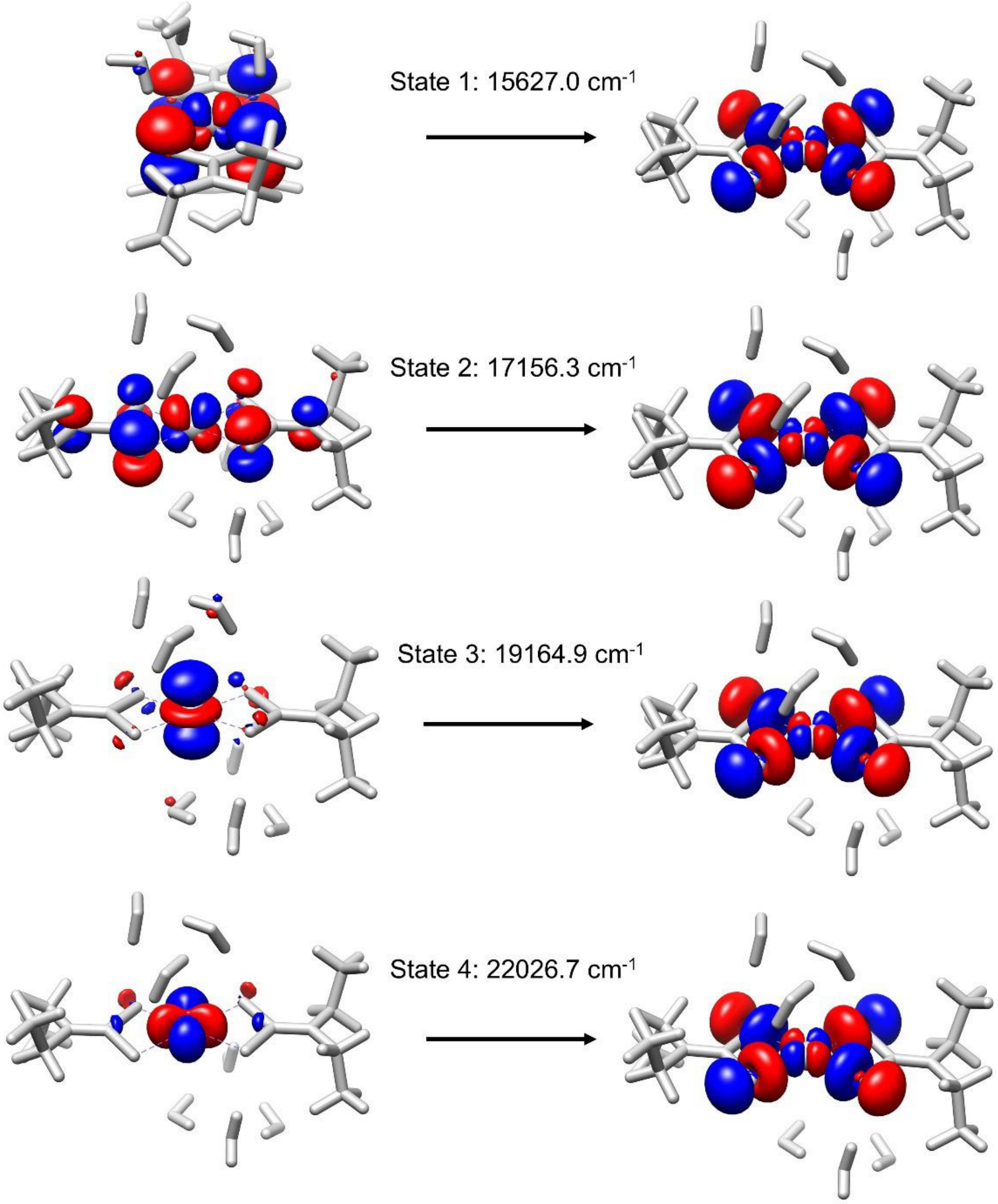

Figure S149: TDDFT natural transition orbitals for [$Cu(dtc)_2 + 7H_2O$].

Table S87: TDDFT-calculated [Cu(dtc)$_2$ + 7H_2O] UV-vis-NIR absorption spectrum.

Calculated via transition electric dipole moments, including oscillator strength (f_{osc}), net squared transition electric dipole moment (D^2), and transition electric dipole moment vector components.

State	Energy (cm^{-1})	Wavelength (nm)	f_{osc}	D^2 (a.u.^{2})	D_x (a.u.)	D_y (a.u.)	D_z (a.u.)
1	15627	639.9	2.69E-05	0.00057	0.00984	-0.01952	0.0094
2	17156.3	582.9	0.001954	0.03749	0.19357	-0.00388	-0.00321
3	19164.9	521.8	0.000219	0.00377	-0.06029	-0.01085	-0.00367
4	19280	518.7	0.01012	0.1728	0.41502	-0.00008	0.02368
5	21652.3	461.8	0.390991	5.94481	2.43667	-0.02611	-0.08234
6	22026.7	454	0.003248	0.04854	0.22004	0.00159	-0.01089
7	24530.4	407.7	9.67E-05	0.0013	0.0045	-0.03573	-0.00098
8	25164.4	397.4	0.000189	0.00248	0.02933	-0.03826	0.01241
9	25977.6	384.9	0.000275	0.00348	-0.03203	0.04931	-0.00508
10	27048.6	369.7	0.001429	0.0174	0.09214	0.09279	-0.01719
11	27283.9	366.5	0.029883	0.36058	-0.00346	-0.59749	0.05975
12	27850.9	359.1	0.001176	0.0139	0.06989	-0.09452	0.00899
13	31317.5	319.3	0.000622	0.00654	0.08073	0.00102	-0.00448
14	31462.6	317.8	0.000624	0.00653	0.01485	-0.07925	0.00532
15	32028.4	312.2	0.000423	0.00434	0.06449	0.01346	-0.0023
16	32512.7	307.6	0.000129	0.00131	0.00731	0.03526	-0.00311
17	32904	303.9	5E-05	0.0005	-0.01482	-0.00744	-0.01499
18	33490.1	298.6	0.001898	0.01866	0.13613	-0.01078	-0.0034
19	34305.1	291.5	1.64E-05	0.00016	0.0003	0.0123	-0.00235
20	35793.2	279.4	0.001207	0.0111	0.05987	0.07877	-0.03621
21	35909.3	278.5	0.002281	0.02091	-0.02602	-0.14223	0.00299
22	36056.1	277.3	0.003942	0.036	0.06026	0.17335	-0.04811
23	36200.4	276.2	0.001627	0.0148	-0.05399	-0.10593	0.02572
24	36874.9	271.2	0.111649	0.99678	-0.05763	0.9946	-0.06499
25	37298.5	268.1	0.000795	0.00702	-0.03591	0.07564	0.00225
26	37744.2	264.9	0.008998	0.07848	0.26796	0.08165	-0.0036
27	38304.3	261.1	0.00079	0.00679	-0.08146	0.00213	0.01212
28	38328.1	260.9	0.012479	0.10718	0.07553	-0.31788	0.02085
29	38515.4	259.6	0.000391	0.00334	0.05681	-0.00043	0.01058
30	39234.3	254.9	0.014532	0.12193	0.10851	-0.33162	0.01367
31	39795.9	251.3	0.003196	0.02644	0.09965	-0.12847	0.00186
32	39990.3	250.1	0.028604	0.23547	0.22285	0.42967	-0.03465
33	40081.1	249.5	0.421973	3.46593	1.86003	-0.00503	-0.07879
34	40684.8	245.8	4.79E-05	0.00039	-0.01505	-0.01202	0.00403
35	41060.5	243.5	0.006828	0.05475	-0.03408	-0.23136	0.0075

7.5.6 $[Cu(mnt)_2]^{2-}$ + $7H_2O$

Table S88: TDDFT-calculated [$[Cu(mnt)_2]^{2-}$ + $7H_2O$] excited states assigned to d-d transitions.

State	NTO Occupation Number	Energy (cm^{-1})	Assignment
3	0.98659666	17538.0	yz → xy
5	0.98599073	18092.0	z^2 → xy
7	0.99858112	21425.3	x^2–y^2 → xy
9	0.99531665	22288.9	xz → xy

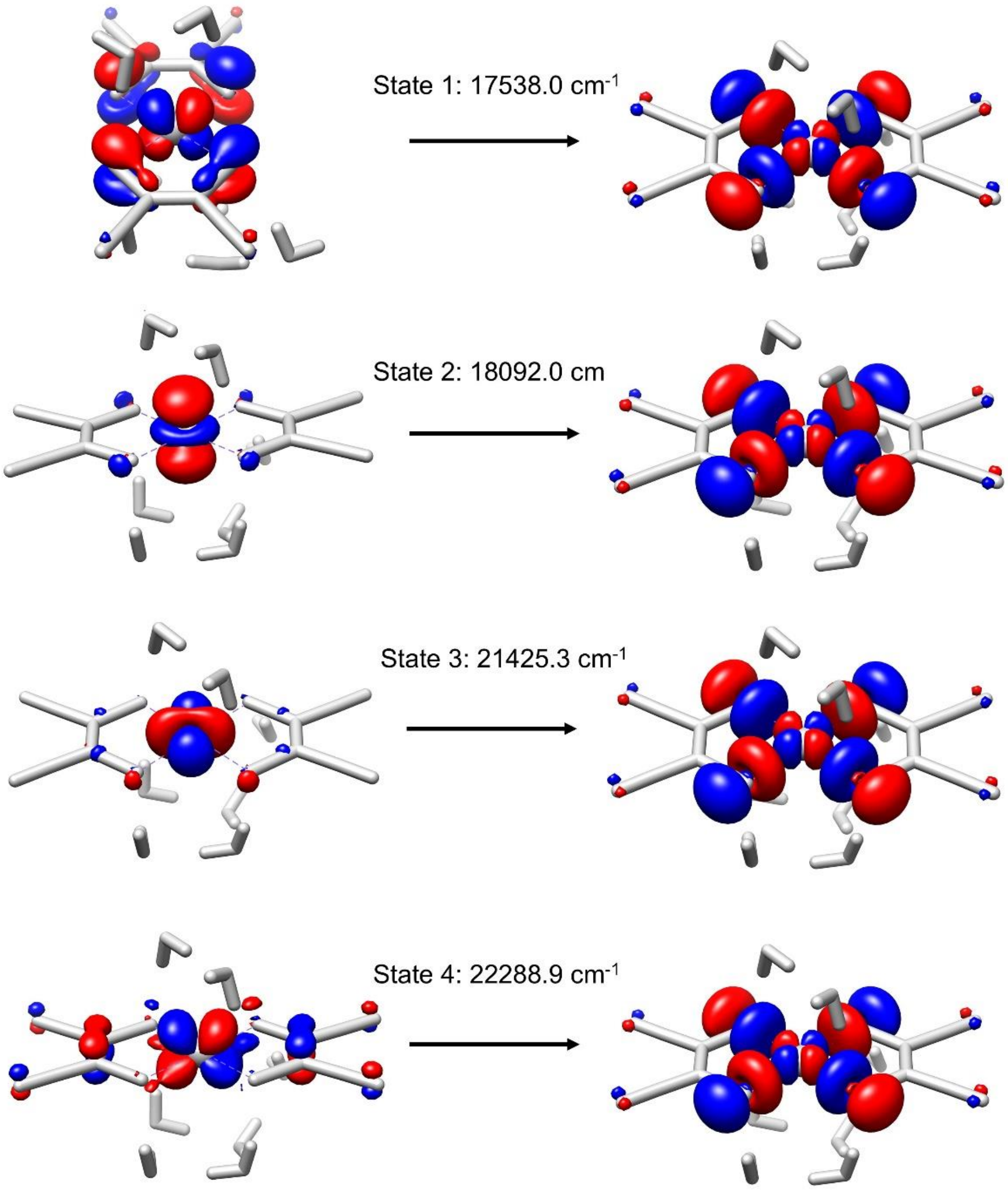

Figure S150: TDDFT natural transition orbitals for [$[Cu(mnt)_2]^{2-}$ + $7H_2O$].

Table S89: TDDFT-calculated [[Cu(mnt)$_2$]$^{2-}$ + 7H_2O] UV-vis-NIR absorption spectrum.

Calculated via transition electric dipole moments, including oscillator strength (f_{osc}), net squared transition electric dipole moment (D^2), and transition electric dipole moment vector components.

State	Energy (cm^{-1})	Wavelength (nm)	f_{osc}	D^2 (a.u.^{2})	D_x (a.u.)	D_y (a.u.)	D_z (a.u.)
1	8352	1197.3	9.14E-06	0.00036	-0.0165	0.0021	0.00915
2	14454	691.8	0.000159	0.00363	0.05736	0.00348	-0.01813
3	17538	570.2	0.000127	0.00238	-0.0488	0.00053	0.00098
4	17688.2	565.4	7.03E-07	0.00001	0.00279	0.00004	-0.0023
5	18092	552.7	1.85E-05	0.00034	-0.01694	0.00508	0.0049
6	18329.8	545.6	0.000111	0.002	0.00174	-0.04468	0.00152
7	21425.3	466.7	0.000396	0.00609	0.07797	0.00338	-0.00014
8	21741.1	460	0.000163	0.00247	-0.04948	0.00145	-0.0045
9	22288.9	448.7	7.51E-05	0.00111	-0.03311	0.00281	0.00231
10	22925.1	436.2	0.096302	1.38293	-1.17564	-0.00841	-0.0273
11	23052.5	433.8	0.183599	2.62198	1.61848	-0.01498	0.04786
12	23136.3	432.2	0.077014	1.09585	-1.04555	0.04545	-0.02464
13	23892.7	418.5	0.001637	0.02256	-0.14858	-0.0076	-0.02056
14	24533.2	407.6	0.00025	0.00336	-0.01688	-0.02794	0.04791
15	26733.8	374.1	0.026378	0.32483	-0.00602	-0.56968	0.01625
16	27188.9	367.8	0.005803	0.07027	0.00349	0.26493	-0.00826
17	27763.2	360.2	2.86E-05	0.00034	0.00089	-0.0181	-0.00319
18	28496.7	350.9	0.000105	0.00122	0.0341	-0.00133	0.00722
19	31092.6	321.6	0.000198	0.0021	-0.0021	0.02244	-0.03988
20	31118.4	321.4	0.000206	0.00218	-0.00303	-0.02461	0.03954
21	31679.6	315.7	3.61E-05	0.00038	0.01559	-0.01141	-0.00158
22	31720.9	315.2	0.001257	0.01305	-0.11067	-0.02755	-0.00615
23	32552.8	307.2	0.006826	0.06903	-0.01199	-0.26052	-0.03188
24	32566.2	307.1	0.000678	0.00685	0.00762	0.07854	0.025
25	32706.4	305.8	0.000506	0.00509	0.00531	-0.07041	-0.01035
26	33052.7	302.5	0.764678	7.61634	-0.07091	-2.75661	0.11143
27	33893.3	295	0.000396	0.00384	-0.00032	0.06114	-0.01021
28	33916.6	294.8	0.00059	0.00573	0.01125	-0.0747	0.00498
29	34428.4	290.5	2.06E-05	0.0002	0.00044	0.01402	0.00054
30	34710.6	288.1	0.002406	0.02282	0.0016	0.15016	-0.01637
31	35219.7	283.9	0.000175	0.00164	0.01009	0.00495	0.03885
32	35260.9	283.6	0.000152	0.00142	0.03056	-0.01106	0.01897
33	35971.3	278	0.000702	0.00642	-0.03434	0.03064	-0.0656
34	36089.6	277.1	0.000281	0.00257	-0.0051	0.01997	-0.04629
35	36344.7	275.1	0.000368	0.00333	-0.01556	-0.00409	0.05546
36	36435.4	274.5	0.000119	0.00107	-0.01797	0.02632	-0.00762
37	37242.1	268.5	0.000452	0.004	-0.04883	-0.0359	-0.01796
38	37371.1	267.6	0.000143	0.00126	0.02948	-0.01965	0.00041
39	37599	266	0.000633	0.00554	0.03027	0.06746	-0.00872

40	37825.7	264.4	0.000341	0.00297	-0.05338	0.0081	-0.00727
41	37900.2	263.9	9.35E-05	0.00081	0.0248	-0.00783	0.01167
42	37977.4	263.3	0.051106	0.44302	0.66289	-0.0236	0.05511
43	38124.6	262.3	0.153221	1.32309	1.14971	-0.03493	0.00647
44	38481.1	259.9	0.001271	0.01087	0.10379	-0.0099	-0.00051
45	38522.5	259.6	0.077987	0.66647	0.81491	-0.02336	0.04298
46	38595.6	259.1	0.000339	0.00289	-0.03265	-0.0201	0.03769
47	38720.1	258.3	0.019851	0.16878	0.39544	-0.11115	-0.00715
48	38721.7	258.3	0.007195	0.06117	-0.16286	-0.18557	0.01446
49	38739.4	258.1	0.004847	0.04119	-0.16678	-0.11561	0.00291
50	38976.3	256.6	7.82E-05	0.00066	0.01386	0.01717	0.01317

7.5.7 $[Cu(ox)_2]^{2-}$ + $7H_2O$

Table S90: TDDFT-calculated $[[Cu(ox)_2]^{2-} + 7H_2O]$ excited states assigned to d-d transitions.

State	NTO Occupation Number	Energy (cm^{-1})	Assignment
1	0.99763757	12269.4	$z^2 \rightarrow xy$
2	0.99880213	15078.4	$xz \rightarrow xy$
3	0.99896099	15485.4	$yz \rightarrow xy$
4	0.99928557	16483.6	$x^2–y^2 \rightarrow xy$

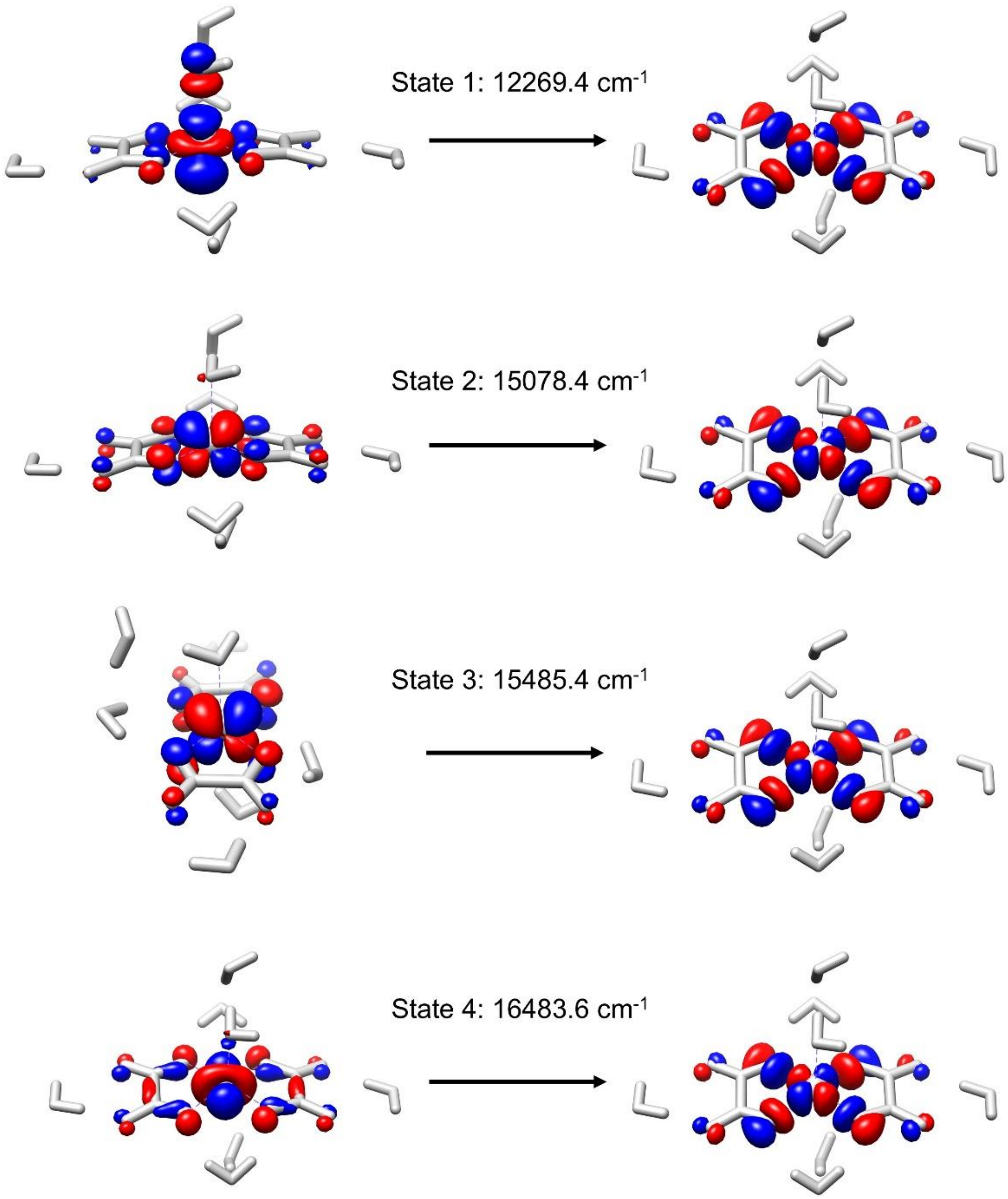

Figure S151: TDDFT natural transition orbitals for $[[Cu(ox)_2]^{2-} + 7H_2O]$.

Table S91: TDDFT-calculated [[Cu(ox)$_2$]$^{2-}$ + 7H_2O] UV-vis-NIR absorption spectrum

Calculated via transition electric dipole moments, including oscillator strength (f_{osc}), net squared transition electric dipole moment (D^2), and transition electric dipole moment vector components.

State	Energy (cm^{-1})	Wavelength (nm)	f_{osc}	D^2 (a.u.^{2})	D_x (a.u.)	D_y (a.u.)	D_z (a.u.)
1	12269.4	815	1.6E-05	0.00043	0.0041	-0.00345	0.02001
2	15078.4	663.2	0.000352	0.00769	-0.04983	-0.07212	-0.00323
3	15485.4	645.8	0.001155	0.02456	-0.15666	-0.00391	0.00201
4	16483.6	606.7	2.58E-06	0.00005	-0.00714	0.00068	0.00031
5	25447.4	393	0.002248	0.02908	0.17003	0.01284	0.00255
6	27598.3	362.3	3.95E-06	0.00005	-0.00679	0.00083	0.0006
7	29769.8	335.9	0.25494	2.81929	-1.67894	-0.01871	0.00959
8	32306	309.5	5.9E-05	0.0006	-0.00365	0.00519	0.02368
9	33048.9	302.6	4.52E-05	0.00045	0.02038	-0.00579	0.00117
10	33112.1	302	1.59E-05	0.00016	-0.01221	0.00111	0.00278
11	34506	289.8	0.000311	0.00296	-0.04194	0.0347	0.00019
12	35468.2	281.9	7.97E-05	0.00074	-0.01724	-0.01999	0.00654
13	35915.1	278.4	0.000683	0.00626	0.03303	0.07125	0.00982
14	36085.8	277.1	0.000242	0.00221	-0.01028	-0.04055	-0.02145
15	36303.8	275.5	0.001197	0.01085	0.00231	0.06282	0.08309
16	36630.7	273	0.006356	0.05713	-0.01771	-0.23756	0.01949
17	37477.8	266.8	0.000225	0.00197	0.00033	0.0168	-0.04111
18	37525.7	266.5	0.000387	0.0034	-0.01031	0.02217	-0.0529
19	37976.9	263.3	0.052557	0.45561	-0.04825	-0.6723	-0.03589
20	39748.7	251.6	4.54E-05	0.00038	0.00134	-0.01935	-0.00016
21	39752.9	251.6	0.000251	0.00208	0.0048	0.0453	0.00171
22	39844.3	251	3.11E-06	0.00003	0.00501	0.00016	-0.00079
23	40468	247.1	6.38E-06	0.00005	-0.0043	-0.00575	-0.00061
24	40514.5	246.8	0.000206	0.00167	0.03446	0.02112	0.00608
25	40535.3	246.7	0.000084	0.00068	0.01494	-0.02063	-0.00579

7.5.8 Cu(hfac)₂ + 7MeOH

Table S92: TDDFT-calculated [Cu(hfac)₂ + 7MeOH] excited states assigned to d-d transitions.

State	NTO Occupation Number	Energy (cm^{-1})	Assignment
1	0.99555171	6951.3	$z^2 \rightarrow xy$
2	0.99874199	12047.9	$xz \rightarrow xy$
3	0.99841405	14836.0	$yz \rightarrow xy$
4	0.99900189	15333.0	$x^2–y^2 \rightarrow xy$

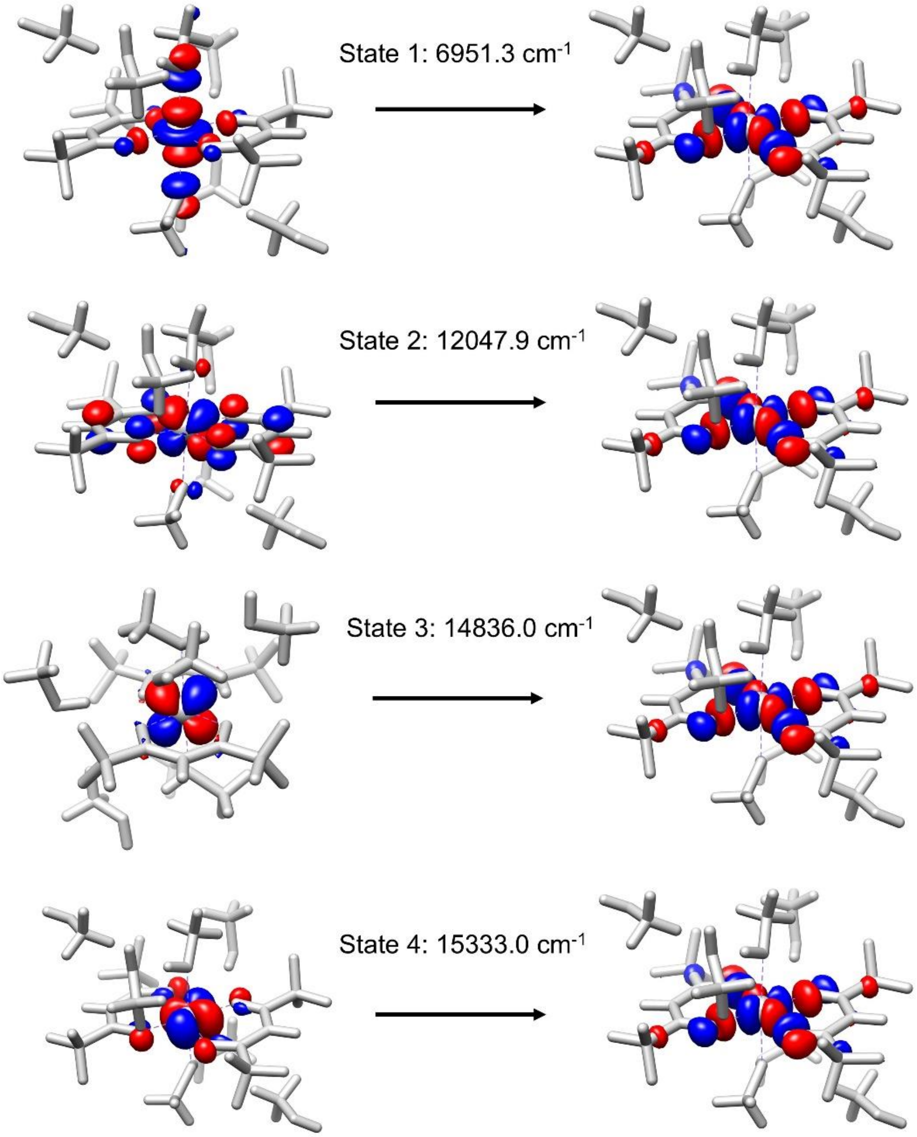

Figure S152: TDDFT natural transition orbitals for [Cu(hfac)₂ + 7MeOH].

Table S93: TDDFT-calculated [Cu(hfac)$_2$ + 7MeOH] UV-vis-NIR absorption spectrum.

Calculated via transition electric dipole moments, including oscillator strength (f_{osc}), net squared transition electric dipole moment (D^2), and transition electric dipole moment vector components.

State	Energy (cm^{-1})	Wavelength (nm)	f_{osc}	D^2 (a.u.^{2})	D_x (a.u.)	D_y (a.u.)	D_z (a.u.)
1	6951.3	1438.6	1.63E-06	0.00008	0.00789	0.00311	0.00232
2	12047.9	830	1.5E-06	0.00004	-0.00583	-0.00048	-0.00261
3	14836	674	7.64E-06	0.00017	0.01298	-0.00084	0.00056
4	15333	652.2	6.95E-06	0.00015	-0.01131	-0.00446	-0.00116
5	21926.9	456.1	0.000228	0.00342	0.0455	-0.034	0.0139
6	22759.1	439.4	6.68E-06	0.0001	-0.00133	-0.00966	-0.00125
7	23055.7	433.7	1.01E-05	0.00014	-0.01106	-0.00235	-0.00397
8	23187.1	431.3	8.63E-06	0.00012	-0.00929	-0.00474	-0.0037
9	27119.9	368.7	0.001114	0.01352	0.10377	-0.04994	0.01621
10	27576.9	362.6	0.000824	0.00984	-0.09064	0.03435	-0.02099
11	28235.5	354.2	0.001066	0.01243	0.10334	0.03304	0.02559
12	29017.4	344.6	0.000778	0.00882	-0.09264	-0.00637	-0.01417
13	29229.6	342.1	0.004218	0.0475	-0.21607	-0.01467	-0.02449
14	29350.9	340.7	0.017872	0.20046	0.44478	0.03815	0.0342
15	29506.9	338.9	0.16538	1.84517	-1.35095	-0.10385	-0.09652
16	29840.8	335.1	0.047398	0.5229	0.72159	0.04708	0.00171
17	31075.2	321.8	2.57E-05	0.00027	0.00819	0.01371	-0.00416
18	31327.1	319.2	0.000371	0.0039	-0.06128	0.01036	0.00601
19	31526.1	317.2	7.94E-06	0.00008	0.00606	0.00661	0.00158
20	32246.3	310.1	0.000401	0.00409	-0.00572	0.06372	0.001
21	33173.2	301.4	0.004084	0.04053	0.02578	-0.19958	-0.00546
22	33430.5	299.1	0.00986	0.0971	0.00454	-0.31155	-0.00382
23	33526.4	298.3	0.001199	0.01178	-0.00545	0.10813	0.00738
24	33586.7	297.7	0.00952	0.09331	-0.01645	0.30424	0.02181
25	33946.2	294.6	0.005236	0.05078	-0.01411	0.22448	0.01382
26	34142.2	292.9	0.001668	0.01608	-0.01156	0.12627	0.00169
27	34375	290.9	4.52E-05	0.00043	-0.01692	0.00323	-0.01166
28	35008.7	285.6	0.002133	0.02005	-0.09911	-0.00394	-0.10107
29	35210.1	284	0.000673	0.0063	-0.05417	-0.00459	-0.0578
30	35537.9	281.4	3.34E-05	0.00031	0.00179	0.01506	0.00894
31	35691.1	280.2	0.001136	0.01048	-0.02331	0.02474	-0.09655
32	35725	279.9	0.000174	0.0016	0.03381	-0.02084	-0.00527
33	35904.3	278.5	0.001587	0.01455	-0.01746	0.01697	-0.11815
34	36052.3	277.4	0.000754	0.00688	-0.02237	0.00336	-0.07982
35	36161.7	276.5	0.000203	0.00185	0.01976	-0.0232	0.0303
36	36264.8	275.7	0.000406	0.00369	0.00653	0.01265	0.05902
37	36296.3	275.5	0.00107	0.0097	-0.03885	0.02252	-0.08768
38	36406.5	274.7	0.000262	0.00237	0.02377	0.01673	0.03905
39	36617.7	273.1	9.98E-05	0.0009	0.01245	-0.02565	0.00918

40	36637.1	272.9	0.001842	0.01655	-0.03489	0.12288	-0.0154
41	37186.1	268.9	0.000105	0.00093	-0.02117	0.02162	-0.00364
42	37219.2	268.7	5.39E-05	0.00048	-0.00794	0.02004	0.00339
43	37481.3	266.8	0.000258	0.00226	-0.03338	-0.0336	0.00454
44	38021.2	263	0.000593	0.00514	0.02028	-0.06855	0.00498
45	38290	261.2	3.05E-05	0.00026	-0.00647	0.00036	-0.01484
46	38316.4	261	6.47E-05	0.00056	0.01177	-0.01181	0.01666
47	38475.1	259.9	2.13E-05	0.00018	-0.00699	-0.00317	-0.01112
48	38491.6	259.8	2.12E-05	0.00018	-0.00756	-0.00138	-0.01107
49	38754.3	258	0.001939	0.01647	-0.02695	0.12502	0.01076
50	39388.1	253.9	0.000189	0.00158	-0.00482	0.03719	0.01306

7.5.9 $[Cu(ox)_2]^{2-}$ + 7MeOH

Table S94: TDDFT-calculated $[[Cu(ox)_2]^{2-}$ + 7MeOH] excited states assigned to d-d transitions.

State	NTO Occupation Number	Energy (cm^{-1})	Assignment
1	0.99774061	11286.7	$z^2 \rightarrow xy$
2	0.99902547	15262.4	$xz \rightarrow xy$
3	0.99909779	15604.8	$yz \rightarrow xy$
4	0.99930789	16523.2	$x^2–y^2 \rightarrow xy$

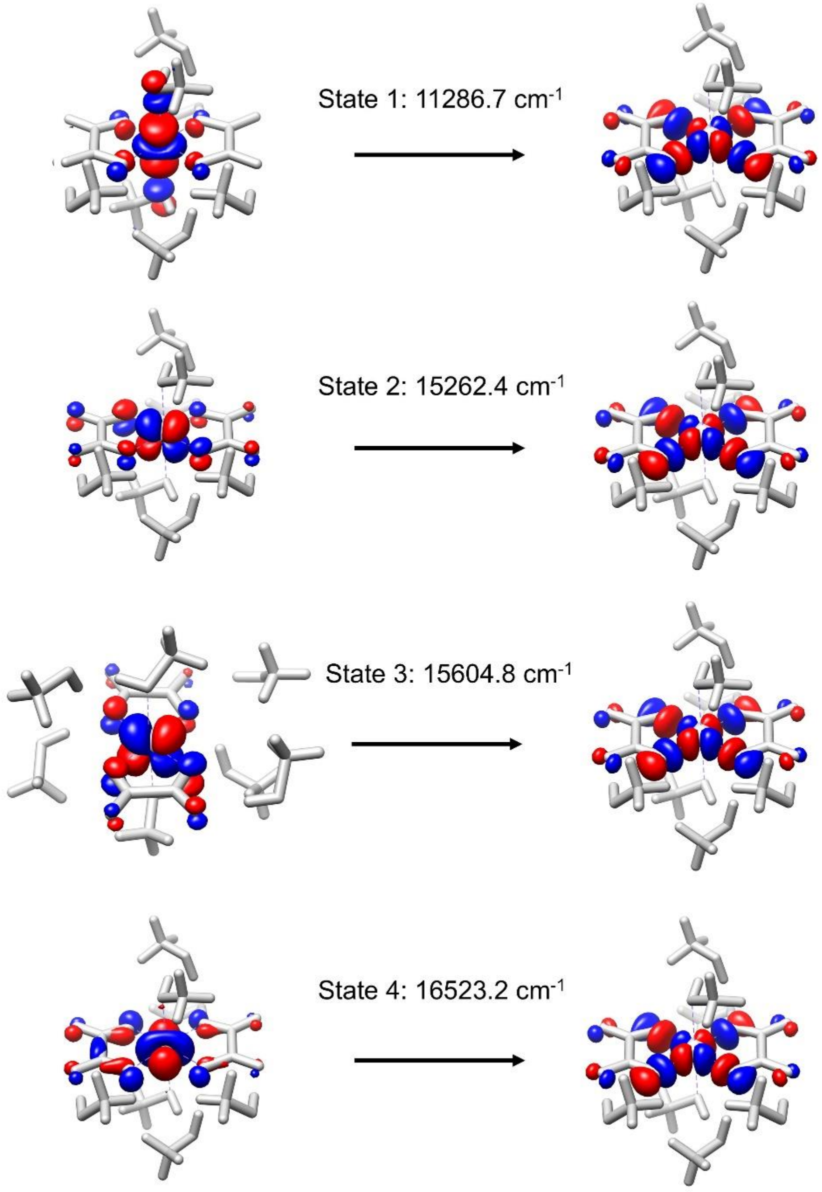

Figure S153: TDDFT natural transition orbitals for $[[Cu(ox)_2]^{2-}$ + 7MeOH].

Table S95: TDDFT-calculated [[Cu(ox)$_2$]$^{2-}$ + 7MeOH] UV-vis-NIR absorption spectrum.

Calculated via transition electric dipole moments, including oscillator strength (f_{osc}), net squared transition electric dipole moment (D^2), and transition electric dipole moment vector components.

State	Energy (cm^{-1})	Wavelength (nm)	f_{osc}	D^2 (a.u.^{2})	D_x (a.u.)	D_y (a.u.)	D_z (a.u.)
1	11286.7	886	2.36E-05	0.00069	0.01325	0.01909	-0.01217
2	15262.4	655.2	2.07E-05	0.00045	-0.0192	-0.0088	0.00024
3	15604.8	640.8	4.75E-06	0.0001	0.00884	0.00374	0.00282
4	16523.2	605.2	2.06E-05	0.00041	0.01582	0.01264	-0.00031
5	26195.2	381.7	0.000466	0.00586	0.06861	0.03384	0.00285
6	29158.4	343	0.036222	0.40896	0.63747	0.04638	0.02116
7	30596.1	326.8	0.188527	2.02855	-1.41768	-0.13269	-0.03353
8	32688.6	305.9	0.000181	0.00182	0.04211	-0.0043	-0.00544
9	32788.9	305	0.000678	0.00681	-0.08208	0.00524	0.00684
10	33136.4	301.8	0.000406	0.00403	0.0631	-0.00674	0.00203
11	33306	300.2	0.001326	0.01311	-0.10029	0.04915	-0.02518
12	33910.1	294.9	1.53E-05	0.00015	0.00994	0.00087	-0.00699
13	34094.6	293.3	0.00016	0.00155	0.00469	-0.03512	0.01702
14	34353	291.1	0.000271	0.00259	-0.03016	0.0396	-0.01076
15	34634.4	288.7	0.000885	0.00841	0.08551	0.03318	0.00043
16	34665.8	288.5	1.48E-05	0.00014	0.00598	-0.0089	0.00508
17	35606.2	280.8	8.65E-05	0.0008	0.00711	0.02519	0.01072
18	35720.8	279.9	0.000427	0.00393	0.0042	-0.06237	0.00502
19	35863.3	278.8	0.000392	0.0036	0.05792	0.01541	0.00252
20	36991.7	270.3	0.001716	0.01527	0.08765	0.08669	0.00862
21	37120.9	269.4	0.001074	0.00952	-0.06857	-0.06806	0.01381
22	37216.4	268.7	0.00074	0.00654	0.07101	0.02913	0.02551
23	37426	267.2	0.001623	0.01427	0.00083	0.11895	0.01103
24	37465	266.9	0.000932	0.00819	-0.00812	-0.06827	0.05887
25	38655.1	258.7	0.062836	0.53515	0.04812	-0.72672	0.06869
26	39450.6	253.5	0.000201	0.00168	0.01056	-0.03941	0.00361
27	39558.8	252.8	1.24E-05	0.0001	0.00164	0.01001	0.0003
28	39594	252.6	0.001017	0.00846	-0.00629	0.09044	-0.01552
29	39729.4	251.7	5.29E-05	0.00044	-0.00721	0.0187	0.00607
30	40668.2	245.9	3.18E-05	0.00026	0.00317	-0.00065	0.01572

7.5.10 Cu(hfac)$_2$ + 4Toluene

Table S96: TDDFT-calculated [Cu(hfac)$_2$ +4Toluene] excited states assigned to d-d transitions.

State	NTO Occupation Number	Energy (cm^{-1})	Assignment
1	0.99600069	13785.4	z^2 / xz → xy
2	0.99732322	14199.0	z^2 / xz → xy
3	0.99925815	17184.7	yz → xy
4	0.99855567	17217.7	x^2–y^2 → xy

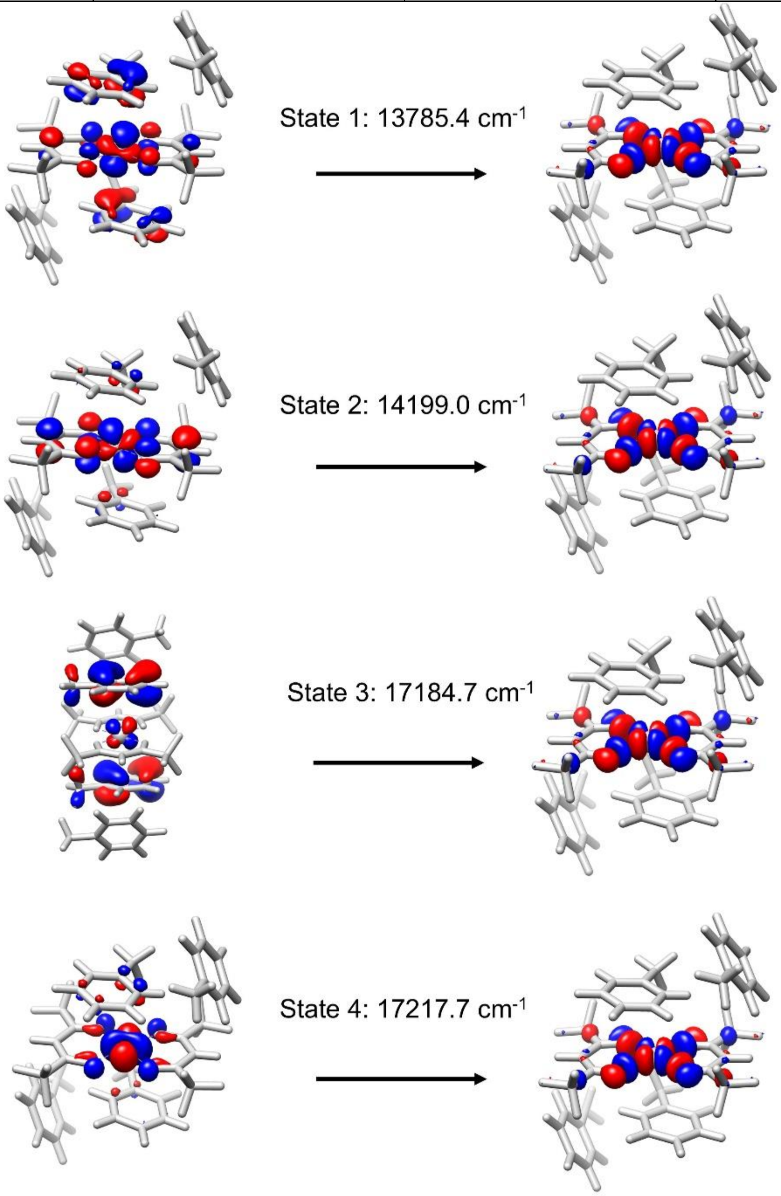

Figure S154: TDDFT natural transition orbitals for [Cu(hfac)$_2$ + 4Toluene].

Table S97: TDDFT-calculated [Cu(hfac)$_2$ + 4Toluene] UV-vis-NIR absorption spectrum.

Calculated via transition electric dipole moments, including oscillator strength (f_{osc}), net squared transition electric dipole moment (D^2), and transition electric dipole moment vector components.

State	Energy (cm^{-1})	Wavelength (nm)	f_{osc}	D^2 (a.u.^{2})	D_x (a.u.)	D_y (a.u.)	D_z (a.u.)
1	13785.4	725.4	1.3E-05	0.00031	-0.01757	0.00114	0.00098
2	14199	704.3	2.36E-06	0.00005	-0.00704	0.00094	-0.00203
3	17184.7	581.9	1.52E-06	0.00003	0.00497	-0.00202	-0.00051
4	17217.7	580.8	3.92E-05	0.00075	0.02663	0.00005	0.00634
5	17881.7	559.2	1.24E-05	0.00023	0.0013	0.01497	0.0015
6	18084.7	553	8.58E-05	0.00156	-0.03335	0.00104	-0.02116
7	18765.6	532.9	1.21E-06	0.00002	-0.00122	-0.00426	-0.00127
8	19882.7	502.9	3.25E-05	0.00054	-0.02031	-0.0024	-0.01094
9	20054.1	498.7	6.49E-06	0.00011	-0.00865	0.00059	-0.00561
10	20854.8	479.5	4.38E-06	0.00007	0.00448	-0.00046	-0.00699
11	22306.4	448.3	7.39E-07	0.00001	-0.00299	-0.00007	0.00139
12	22382.9	446.8	5.37E-06	0.00008	0.00039	-0.0088	-0.00116
13	22778.1	439	8E-08	0	0.00053	0.00073	0.00059
14	22814.8	438.3	5.85E-06	0.00008	-0.00003	-0.00918	-0.00016
15	23588.7	423.9	7.27E-07	0.00001	0.00168	0.00246	0.00113
16	25032.2	399.5	9.54E-05	0.00125	-0.0353	-0.00191	-0.00216
17	26454.9	378	0.000259	0.00322	-0.01101	0.00275	-0.05561
18	26581	376.2	2.04E-05	0.00025	-0.00629	-0.00184	-0.01448
19	26667	375	0.001254	0.01548	-0.00283	-0.00635	0.12422
20	26834.9	372.6	4.63E-05	0.00057	0.0097	-0.00017	0.02177
21	27445.6	364.4	0.000642	0.0077	-0.04365	0.02444	-0.0721
22	27584.3	362.5	0.000676	0.00807	0.05306	-0.02816	0.06682
23	27611.2	362.2	0.000147	0.00175	0.04031	0.01126	-0.00149
24	27763.8	360.2	0.000345	0.00409	0.05312	0.03258	0.01432
25	27801.3	359.7	0.002442	0.02891	-0.12057	-0.05716	-0.10538
26	27934.3	358	0.000902	0.01063	0.07241	-0.07135	0.01723
27	28021.5	356.9	0.005744	0.06748	-0.22729	-0.03694	-0.12022
28	28140.1	355.4	0.004754	0.05562	-0.20857	0.03996	-0.10256
29	28358.9	352.6	0.000719	0.00834	0.02495	-0.04924	0.07277
30	28399	352.1	0.012127	0.14059	-0.35195	-0.0496	-0.11941
31	28493.7	351	0.169315	1.95624	1.38695	0.01298	0.18009
32	28554.8	350.2	0.032222	0.37149	0.59996	0.00813	-0.10709
33	28636	349.2	0.000109	0.00126	0.01473	-0.02163	-0.0239
34	28849.4	346.6	6E-05	0.00068	0.02615	-0.00024	0.00062
35	29042.8	344.3	0.006842	0.07756	-0.25215	0.00354	0.11819
36	29189.6	342.6	0.00078	0.0088	-0.07063	0.03147	0.05311
37	29272.4	341.6	0.000186	0.00209	0.0397	0.01995	-0.01094
38	29377.3	340.4	0.000734	0.00823	-0.06328	0.01421	0.06343
39	29386.8	340.3	5.24E-05	0.00059	0.00106	-0.00728	0.02309

40	29846.8	335	0.000112	0.00123	0.0239	-0.00365	-0.02549
41	29980.4	333.6	8.16E-05	0.0009	-0.01693	-0.00873	0.0231
42	30027.3	333	5.27E-05	0.00058	0.01678	-0.00562	-0.01626
43	30149.8	331.7	2.63E-05	0.00029	-0.00497	-0.00984	0.01289
44	30346.3	329.5	0.001016	0.01102	-0.06942	0.00116	0.07874
45	30540.3	327.4	0.000393	0.00423	0.04294	-0.00248	-0.04883
46	30572.8	327.1	0.000149	0.00161	-0.00483	-0.03887	0.00867
47	30747.3	325.2	0.000367	0.00393	-0.00167	-0.06238	0.00614
48	31785.5	314.6	0.002872	0.02975	0.00002	-0.17244	0.00291
49	32053.8	312	0.000465	0.00477	-0.00345	0.06899	0.00186
50	32108	311.4	0.000174	0.00179	0.03254	-0.00409	-0.02668

7.5.11 $[Cu(ox)_2]^{2-}$ + 4Toluene

Table S98: TDDFT-calculated [$[Cu(ox)_2]^{2-}$ + 4Toluene] excited states assigned to d-d transitions.

State	NTO Occupation Number	Energy (cm^{-1})	Assignment
1	0.99719392	16353.0	$x^2–y^2$ / z^2 → xy
2	0.99865143	17686.5	xz → xy
3	0.99883946	17889.0	yz → xy
4	0.99866631	19062.6	$x^2–y^2$ / z^2 → xy

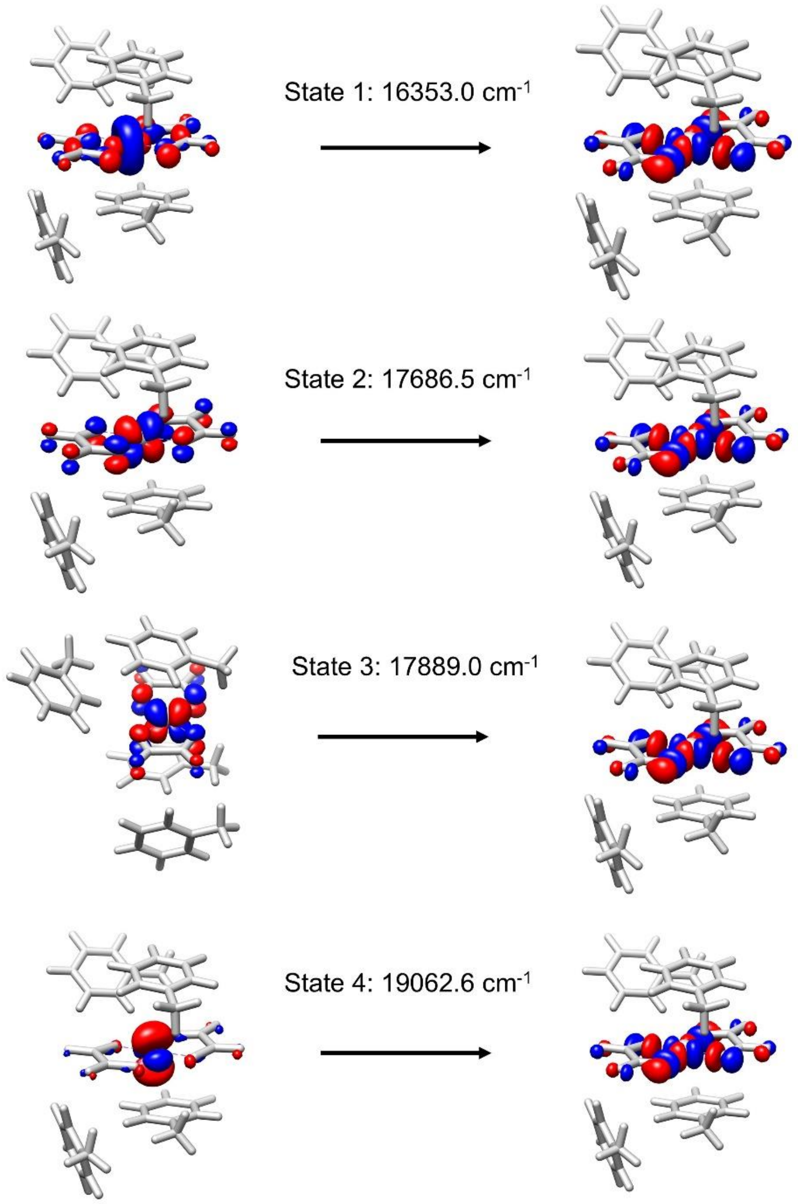

Figure S155: TDDFT natural transition orbitals for [$[Cu(ox)_2]^{2-}$ + 4Toluene].

Table S99: TDDFT-calculated [[Cu(ox)$_2$]$^{2-}$ + 4Toluene] UV-vis-NIR absorption spectrum.

Calculated via transition electric dipole moments, including oscillator strength (f_{osc}), net squared transition electric dipole moment (D^2), and transition electric dipole moment vector components.

State	Energy (cm^{-1})	Wavelength (nm)	f_{osc}	D^2 (a.u.^{2})	D_x (a.u.)	D_y (a.u.)	D_z (a.u.)
1	16353	611.5	8.21E-06	0.00017	-0.00826	-0.00401	-0.00899
2	17686.5	565.4	4.11E-05	0.00077	-0.02529	-0.01121	-0.00017
3	17889	559	1.18E-05	0.00022	-0.01471	0.00094	-0.00009
4	19062.6	524.6	1.07E-05	0.00018	0.01226	-0.00416	-0.00409
5	24941.1	400.9	1.46E-06	0.00002	0.0021	0.00256	0.00289
6	28071.9	356.2	0.000188	0.0022	0.04545	0.01047	0.00506
7	29122.7	343.4	0.218758	2.47291	-1.564	0.07726	-0.14435
8	31767.2	314.8	0.000119	0.00123	0.03268	-0.00268	-0.01239
9	31909.9	313.4	8.56E-06	0.00009	-0.00915	-0.00142	-0.00157
10	32051.9	312	8.4E-05	0.00086	-0.029	0.00448	0.00113
11	32920.8	303.8	0.000251	0.00251	0.04552	0.01088	0.01783
12	33216.8	301.1	0.000446	0.00442	-0.06488	-0.01396	-0.00364
13	33400.9	299.4	6.76E-06	0.00007	0.00711	-0.00176	0.00359
14	33726.6	296.5	5.78E-07	0.00001	0.00202	0.00064	0.00107
15	33728.2	296.5	2.83E-06	0.00003	0.00482	0.00204	0.00052
16	33847.2	295.4	4E-09	0	0.00011	-0.00003	0.00016
17	33922.8	294.8	2E-09	0	0.00004	-0.00003	-0.00011
18	34319.8	291.4	5.51E-05	0.00053	0.02162	-0.00771	-0.0013
19	34404.8	290.7	2.38E-05	0.00023	-0.01284	0.0031	-0.00731
20	35145.2	284.5	0.00045	0.00421	0.01765	0.06238	-0.00288
21	35680.8	280.3	0.000464	0.00428	0.03074	-0.05734	0.00696
22	36005.2	277.7	0.001045	0.00955	0.00196	0.03226	-0.09224
23	36026.7	277.6	0.000275	0.00251	-0.0101	-0.01169	-0.04771
24	36215.4	276.1	0.000717	0.00652	0.07852	0.00905	-0.01644
25	36340.9	275.2	2.37E-05	0.00021	0.0063	-0.00323	-0.01282
26	36367.5	275	0.00045	0.00407	0.06342	-0.00653	0.00262
27	36721.6	272.3	5.99E-05	0.00054	-0.00804	0.02158	0.00248
28	37028.2	270.1	2.84E-05	0.00025	0.01251	-0.00218	0.00957
29	37241	268.5	2.8E-05	0.00025	-0.01351	-0.00461	0.00659
30	37565.4	266.2	5.92E-06	0.00005	-0.00098	-0.00705	-0.00112
31	37671	265.5	0.000252	0.0022	-0.00097	0.04622	0.00786
32	37685.8	265.4	0.004673	0.04083	-0.00745	-0.20177	-0.00773
33	37746.3	264.9	1.04E-07	0	-0.00033	-0.00053	-0.00072
34	37833.4	264.3	0.000519	0.00451	0.0008	0.06709	0.00326
35	37851.9	264.2	0.045489	0.39563	0.03699	0.62769	0.01643
36	37931.7	263.6	0.000699	0.00607	-0.00077	0.07775	0.00492
37	38013.5	263.1	0.001502	0.01301	0.02471	0.11134	0.002
38	38082.1	262.6	2.7E-05	0.00023	-0.00083	-0.01388	-0.00636
39	38145.6	262.2	1.26E-06	0.00001	0.00002	0.00147	-0.00295

40	38167.2	262	0.000956	0.00824	0.00444	0.08931	0.01571
41	38241.8	261.5	1.87E-06	0.00002	0.00011	-0.00327	0.00232
42	38294.8	261.1	1.59E-05	0.00014	-0.00341	-0.01114	0.00098
43	38555.6	259.4	0.00073	0.00623	-0.01032	0.07826	0.00099
44	38702.1	258.4	0.000231	0.00197	0.0102	0.03852	-0.01953
45	38777.4	257.9	0.000274	0.00232	-0.0326	0.0348	-0.00708
46	39016.9	256.3	0.000129	0.00109	-0.02248	0.0215	-0.0111
47	39104.9	255.7	0.000403	0.00339	0.01191	-0.05517	0.01426
48	39137.7	255.5	0.00015	0.00126	-0.01199	0.00203	0.03336
49	39147.6	255.4	0.000208	0.00175	0.01663	0.03386	0.01798
50	39210	255	2.16E-05	0.00018	0.00432	0.00549	-0.01153

7.6 Optimized Geometries

*Table S100: Optimized DFT structures for copper complexes, from methods 1 (**D**) and 2 (**F**)*

	Method 1 (**D**)	Method 2 (**F**)
Cu(acac)$_2$ + 7H_2O	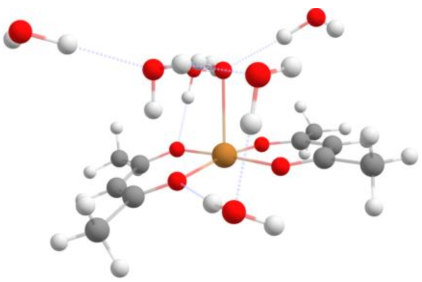	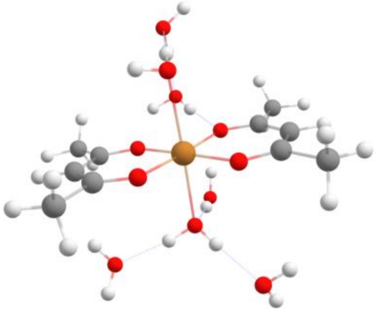
Cu(hfac)$_2$ + 7H_2O	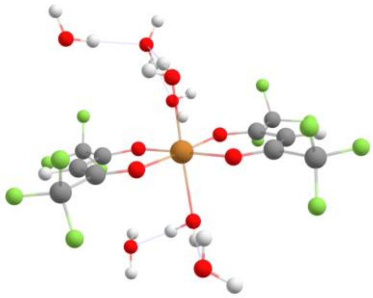	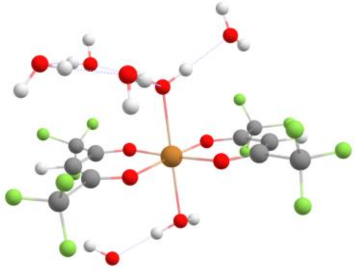
Cu(tmhd)$_2$ + 7H_2O	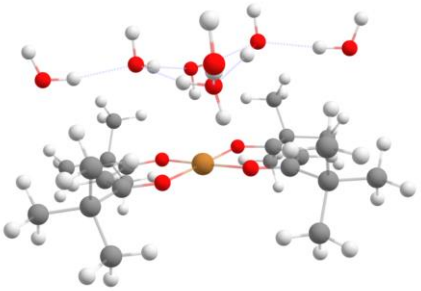	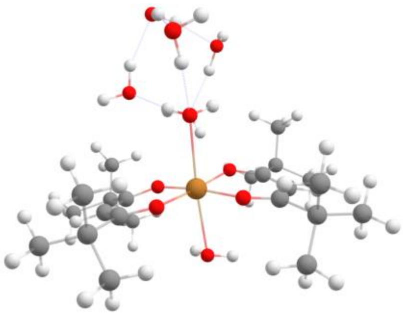
Cu(acacen)$_2$ + 7H_2O	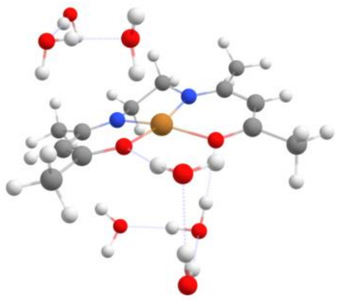	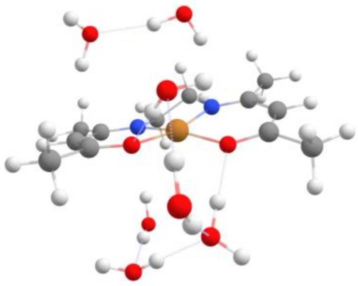

$Cu(dtc)_2$ + $7H_2O$	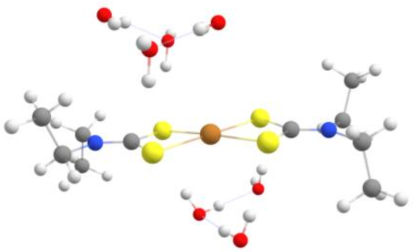	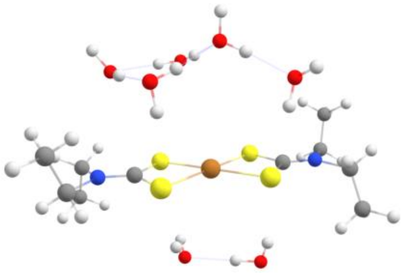
$[Cu(mnt)_2]^{2-}$ + $7H_2O$	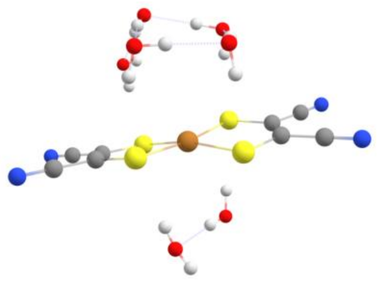	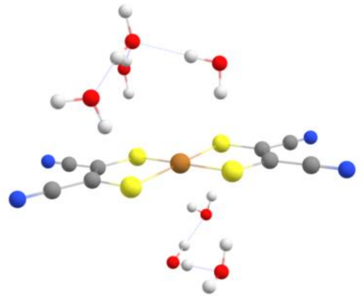
$[Cu(ox)_2]^{2-}$ + $7H_2O$	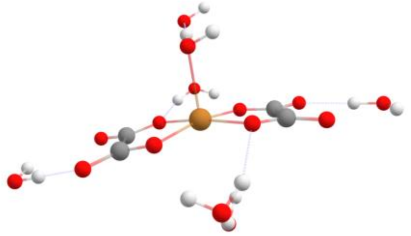	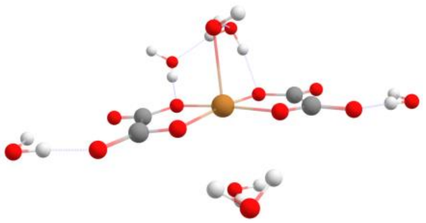
$Cu(hfac)_2$ + 7MeOH	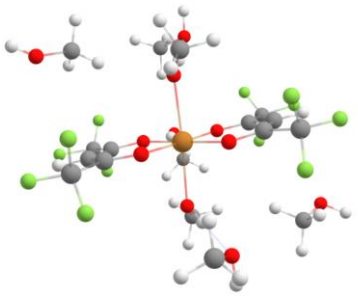	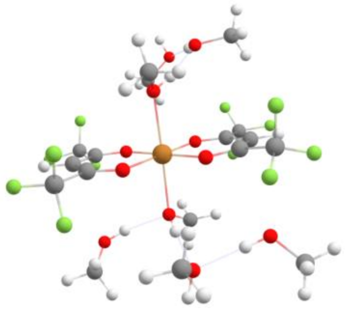

$[Cu(ox)_2]^{2-}$ + 7MeOH	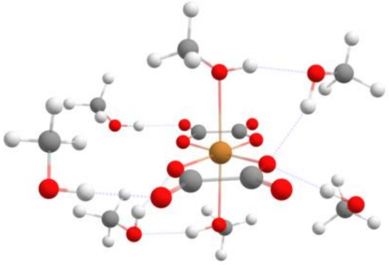	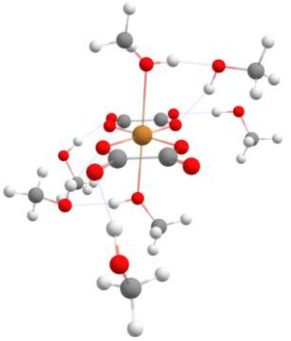
$Cu(hfac)_2$ + 7Toluene	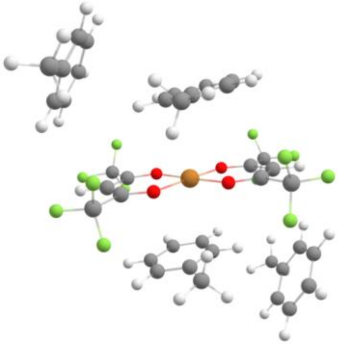	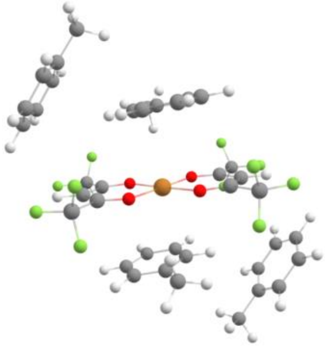
$[Cu(ox)_2]^{2-}$ + 7Toluene	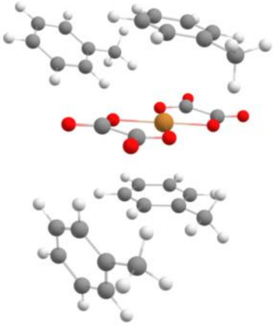	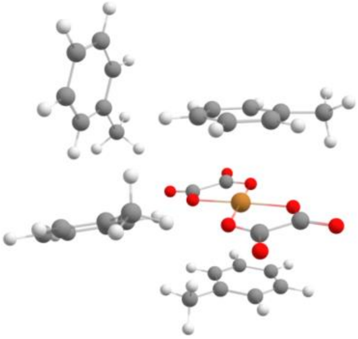

8. References

(1) Edwards, H. G. M.; Farwell, D. W.; Rose, S. J.; Smith, D. N. Vibrational Spectra of Copper (II) Oxalate Dihydrate, $CuC_2O_4 \cdot 2H_2O$, and Dipotassium Bis-Oxalato Copper (II) Tetrahydrate, $K_2Cu(C_2O_4)_2 \cdot 4H_2O$. *J Mol Struct* **1991**, *249* (2–4), 233–243. https://doi.org/10.1016/0022-2860(91)85070-J.

(2) Darensbourg, D. J.; Frantz, E. B. Manganese(III) Schiff Base Complexes: Chemistry Relevant to the Copolymerization of Epoxides and Carbon Dioxide. *Inorg Chem* **2007**, *46* (15), 5967–5978. https://doi.org/10.1021/ic7003968.

(3) Amdur, M. J.; Mullin, K. R.; Waters, M. J.; Puggioni, D.; Wojnar, M. K.; Gu, M.; Sun, L.; Oyala, P. H.; Rondinelli, J. M.; Freedman, D. E. Chemical Control of Spin–Lattice Relaxation to Discover a Room Temperature Molecular Qubit. *Chem Sci* **2022**, *13* (23), 7034–7045. https://doi.org/10.1039/D1SC06130E.

(4) Wansapura, C. M.; Juyoung, C.; Simpson, J. L.; Szymanski, D.; Eaton, G. R.; Eaton, S. S.; Fox, S. From Planar Toward Tetrahedral Copper(Il) Complexes: Structural and Electron Paramagnetic Resonance Studies of Substituent Steric Effects in an Extended Class of Pyrrolate-Imine Ligands. *J Coord Chem* **2003**, *56* (11), 975–993. https://doi.org/10.1080/00958970310001607752.

(5) Davison, A.; Holm, R. H.; Benson, R. E.; Mahler, W. Metal Complexes Derived from *Cis* -1,2-dicyano-1,2-ethylenedithiolate and Bis(Trifluoromethyl)-1,2-dithiete; 1967; pp 8–26. https://doi.org/10.1002/9780470132418.ch3.

(6) Bader, K.; Dengler, D.; Lenz, S.; Endeward, B.; Jiang, S.-D.; Neugebauer, P.; van Slageren, J. Room Temperature Quantum Coherence in a Potential Molecular Qubit. *Nat Commun* **2014**, *5* (1), 5304. https://doi.org/10.1038/ncomms6304.

(7) Alharbi, Y. T.; Alam, F.; Parvez, K.; Missous, M.; Lewis, D. J. Molecular Precursor Route to Bournonite ($CuPbSbS_3$) Thin Films and Powders. *Inorg Chem* **2021**, *60* (17), 13691–13698. https://doi.org/10.1021/acs.inorgchem.1c02001.

(8) Fataftah, M. S.; Krzyaniak, M. D.; Vlaisavljevich, B.; Wasielewski, M. R.; Zadrozny, J. M.; Freedman, D. E. Metal–Ligand Covalency Enables Room Temperature Molecular Qubit Candidates. *Chem Sci* **2019**, *10* (27), 6707–6714. https://doi.org/10.1039/C9SC00074G.

(9) Toby, B. H. *EXPGUI* , a Graphical User Interface for *GSAS*. *J Appl Crystallogr* **2001**, *34* (2), 210–213. https://doi.org/10.1107/S0021889801002242.

(10) Toby, B. H.; Von Dreele, R. B. *GSAS-II* : The Genesis of a Modern Open-Source All Purpose Crystallography Software Package. *J Appl Crystallogr* **2013**, *46* (2), 544–549. https://doi.org/10.1107/S0021889813003531.

(11) Upton, A. H. P.; Williamson, B. E. Magnetic Circular Dichroism and Absorption Spectra of Hexacyanoferrate(III) in a Poly(Vinyl Alcohol) Film. *J Phys Chem* **1994**, *98* (1), 71–76. https://doi.org/10.1021/j100052a013.

(12) Solomon, E. I.; Bell III, C. B. Inorganic and Bioinorganic Spectroscopy. In *Physical Inorganic Chemistry: Principles, Methods, and Models*; Bakac, A., Ed.; John Wiley & Sons, 2010; p 13.

(13) Stoll, S.; Schweiger, A. EasySpin, a Comprehensive Software Package for Spectral Simulation and Analysis in EPR. *Journal of Magnetic Resonance* **2006**, *178* (1), 42–55. https://doi.org/10.1016/j.jmr.2005.08.013.

(14) Du, J.-L.; Eaton, G. R.; Eaton, S. S. Temperature and Orientation Dependence of Electron-Spin Relaxation Rates for Bis(Diethyldithiocarbamato)Copper(II). *J Magn Reson A* **1995**, *117* (1), 67–72. https://doi.org/10.1006/jmra.1995.9971.
(15) McMillin, D. R.; Drago, R. S.; Nusz, J. A. New Insights into the Lewis Acidity of Bis(Hexafluoroacetylacetonato)Copper(II) from EPR and Calorimetry Studies. *J Am Chem Soc* **1976**, *98* (11), 3120–3126. https://doi.org/10.1021/ja00427a010.
(16) Eachus, R. S.; McDugle, W. G. Electron Paramagnetic Resonance Investigations of Photosensitive Transition Metal Oxalates. Copper-Doped Potassium Bis(Oxalato)Palladium(II) Dihydrate. *Inorg Chem* **1973**, *12* (7), 1561–1566. https://doi.org/10.1021/ic50125a017.
(17) Kazmierczak, N. P.; Hadt, R. G. Illuminating Ligand Field Contributions to Molecular Qubit Spin Relaxation via T_1Anisotropy. *J Am Chem Soc* **2022**, *144* (45), 20804–20814. https://doi.org/10.1021/jacs.2c08729.
(18) Mirzoyan, R.; Hadt, R. G. The Dynamic Ligand Field of a Molecular Qubit: Decoherence through Spin-Phonon Coupling. *Physical Chemistry Chemical Physics* **2020**, *22* (20), 11249–11265. https://doi.org/10.1039/d0cp00852d.
(19) Neese, F. Software Update: The ORCA Program System—Version 5.0. *WIREs Computational Molecular Science* **2022**, *12* (5). https://doi.org/10.1002/wcms.1606.
(20) DiMucci, I. M.; Lukens, J. T.; Chatterjee, S.; Carsch, K. M.; Titus, C. J.; Lee, S. J.; Nordlund, D.; Betley, T. A.; MacMillan, S. N.; Lancaster, K. M. The Myth of d ^{8} Copper(III). *J Am Chem Soc* **2019**, *141* (46), 18508–18520. https://doi.org/10.1021/jacs.9b09016.
(21) Bannwarth, C.; Ehlert, S.; Grimme, S. GFN2-XTB—An Accurate and Broadly Parametrized Self-Consistent Tight-Binding Quantum Chemical Method with Multipole Electrostatics and Density-Dependent Dispersion Contributions. *J Chem Theory Comput* **2019**, *15* (3), 1652–1671. https://doi.org/10.1021/acs.jctc.8b01176.
